# Comparative Study of Click Handle Stability in Common Ligation Conditions

**DOI:** 10.1021/acs.bioconjchem.5c00095

**Published:** 2025-04-27

**Authors:** Caitlin Fawcett, Joe Watson, Stephen Richards, Alfred E. Doherty, Hikaru Seki, Elizabeth A. Love, Charlotte H. Coles, Diane M. Coe, Craig Jamieson

**Affiliations:** † Pure and Applied Chemistry, 3527 University of Strathclyde, Thomas Graham Building, 295 Cathedral Street, Glasgow G1 1XL, United Kingdom; ‡ Research Technologies, GSK, Gunnels Wood Road, Stevenage SG1 2NY, United Kingdom

## Abstract

Click chemistry efficiently ligates molecular building blocks in a robust and high-yielding manner and has found major application in the rapid modification of important molecular actors in biological systems. However, the high reactivity of click handles often correlates with decreased stability, which presents a significant challenge in the practical application of these systems. In the current study, we describe a survey of the stability of commonly deployed click manifolds across a range of widely used ligation conditions. Incompatible click handle and ligation condition combinations are identified, with kinetic half-lives and side products of each undesired reaction determined, including the assessment of stability over extended periods and in a protein environment. This data set provides researchers with a roadmap to expediently determine the most appropriate click reaction conditions for any given bioorthogonal application, thus elevating the probability of success of procedures that utilize click chemistry.

## Graphical abstract

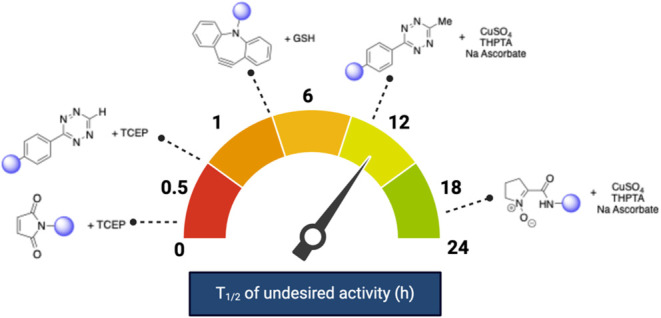

## Introduction

The term click chemistry was first coined by Sharpless in 2001 as a reaction that must be wide in scope, give very high yields, generate only inoffensive byproducts, and be stereospecific.^1^ In the past two decades, click chemistry has revolutionized a range of disciplines, from chemical biology through to radiochemistry, protein engineering, and materials science.^2,3^ The copper-catalyzed azide–alkyne cycloaddition (CuAAC) has enabled notable progress in combinatorial chemical synthesis as it offers mild, reliable, and high-yielding reaction conditions and has also inspired novel approaches within polymer science, including the development of rapid and modular dendrimer syntheses.^4^ The development of click chemistry has underpinned innovation in chemical biology through its ability to avoid cross-reactivity with the diverse range of functionalities present in a biological milieu. This bioorthogonal approach provides a valuable means of selectively derivatizing biological systems, from the synthesis of protein conjugates to profiling enzymatic activities within whole cells and animals.^5–8^

The CuAAC reaction is the most commonly used click reaction;^4^ however, its dependence on the use of metal catalysts presents a key limitation, as these can promote the generation of toxic reactive oxygen species, which often precludes its applications in a cellular context.^2^ This limitation of the CuAAC process prompted the development of strain-promoted variants, which remove the requirement for toxic metal catalysis. Bertozzi and co-workers described the strain-promoted azide–alkyne cycloaddition (SPAAC) in 2004, in which a strained alkyne rapidly ligates an azide, affording a triazole.^9^ The group demonstrated the applicability of SPAAC reactions by the selective, covalent modification of biomolecules in cellular systems with no observed toxicity.^9^

Further click reaction classes have been developed, which have broadened our toolbox to study biological processes in their native settings,^10^ including, but not limited to, the strain-promoted alkyne–nitrone cycloaddition (SPANC), ketone condensation, and the strain-promoted inverse electron demand Diels–Alder (IEDDA) reactions (Figure 1). Each of these reaction classes offers unique strengths within specific applications; notably, the bioorthogonal nature of these reactions has underpinned significant developments in chemical biology. For example, the CuAAC reaction enables the incorporation of small, relatively stable reactive handles into chemical probes, with limited disruption of target–ligand interactions and physicochemical properties of the parent molecule.^11,12^ Meanwhile, the major advantage of the IEDDA reaction is its rapid kinetics of around 10^6^ M^–1^ s^–1^, and thus it can be used to monitor and image reactions or processes in a biological setting.^2^ As such, Sharpless, Bertozzi, and Meldal were awarded the Nobel Prize for Chemistry in 2022 for the “development of click chemistry and bioorthogonal chemistry”.^13^ Numerous excellent reviews detailing each of these reactions and their extensive application are available, including Oliveira et al.,^2^ Meldal et al.,^14^ Moses et al.,^15^ Mackenzie et al.,^16^ Kaur et al.,^17^ and Nair et al.^18^

**Figure 1:**
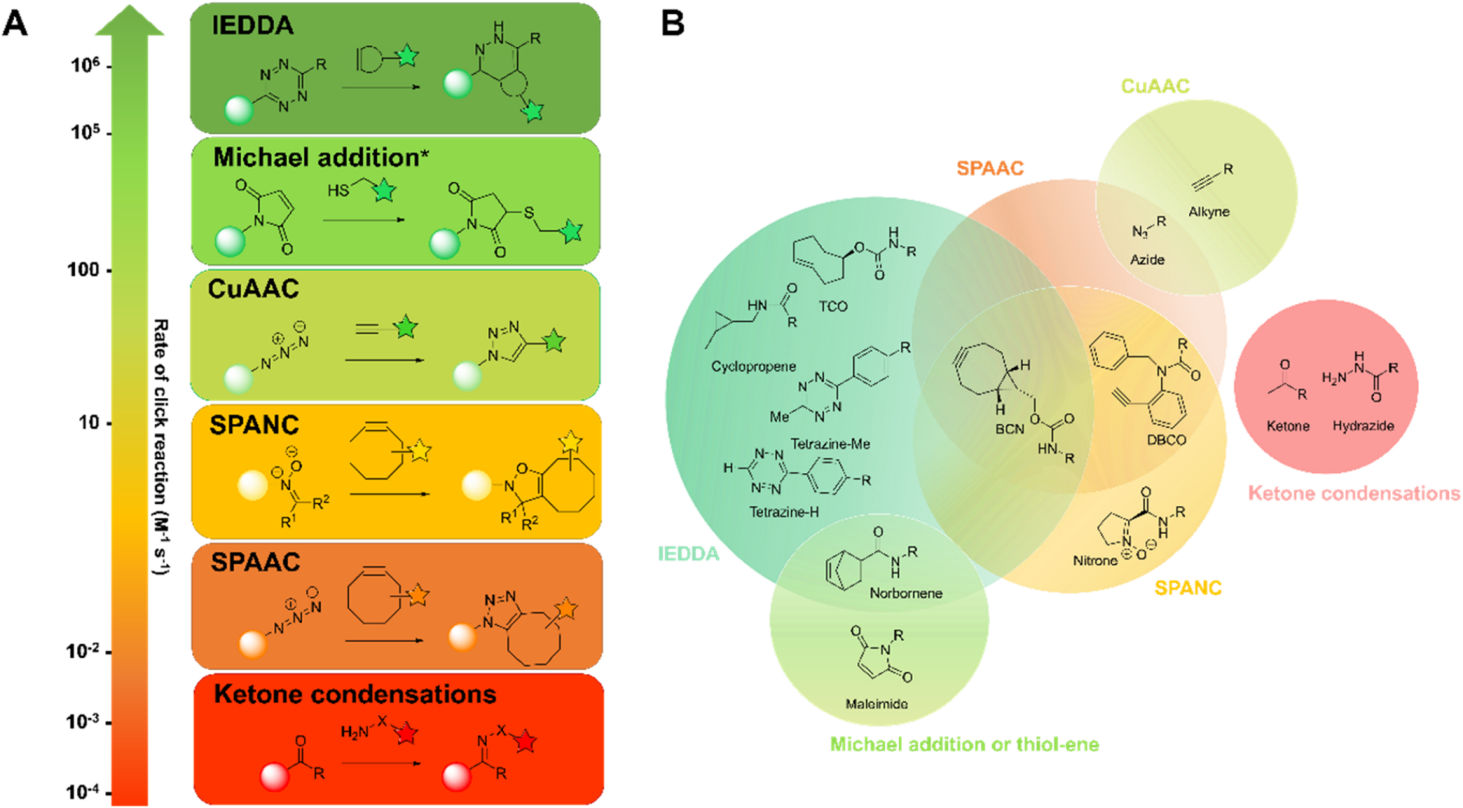
Relative reaction rates and representative functional groups for selected click and bioorthogonal reactions. (A) The relative rates of the inverse electron demand Diels–Alder (IEDDA), Michael addition, copper-catalyzed azide–alkyne cycloaddition (CuAAC), strain-promoted alkyne–nitrone cycloaddition (SPANC), strain-promoted alkyne–azide cycloaddition (SPAAC), and ketone condensation reactions. (B) Representative functional groups, which can be used as click handles within the listed click reactions. Michael addition and thiol–ene reactions are included within the figure, as they are not formally click processes but are often considered as bioorthogonal click reactions due to their rapid kinetics and innocuous byproducts.^19^ Furthermore, there is known reactivity associated with maleimides; therefore, we deemed it suitable for assessment in this comparative study. Norbornenes are only discussed in the context of IEDDA reactions herein due to the requirement for photoactivation of their thiol–ene reaction. Photoactivated click reactions are not discussed herein.^20^

Due to the inherently high reactivity of most click handles, they can be susceptible to undesirable cross-reactivity. In many applications, this is not an appreciable concern, as the rate of the click reaction generally surpasses that of any competing reaction pathways. Click reactions can be compromised, however, when the competing reaction occurs prior to initiation of the desired click reaction. For example, when generating antibody drug conjugates (ADCs) or chemically linked bispecific antibodies, ligation of a chemical linker containing a click handle to a monoclonal antibody (mAb) or antibody fragment (Fab) is often carried out prior to a click reaction.^8,21,22^ During this ligation, the click handles may be exposed to conditions that promote the undesired cross-reactivity. For example, cysteine conjugation reactions require reducing agents such as tris­(2-carboxyethyl)­phosphine (TCEP) to reduce the mAb interchain disulfide bonds to liberate free thiols under aqueous conditions.^23^ Therefore, the click handle employed must be stable to TCEP, free thiols, aqueous conditions, and other chemical groups on the conjugation warhead. Conditions used for the subsequent click reaction must also not interfere with the mAbs themselves; for example, CuAAC conditions may cause oxidation of mAb binding regions, which may potentially decrease target binding.^24^

Click reactions are a final stage of many multistep workflows,^25^ and many researchers are focused on attaching two reactive groups to proteins, which can be done chemically or via genetic code expansion.^26^ Therefore, it is imperative during experimental design to understand how all experimental parameters could impact the selected click reaction and whether the click handles are compatible with reaction conditions throughout. As there is no universal click reaction class suitable for all applications, the benefits and limitations of each click reaction should be carefully considered before the selection of a reaction for a specific application.^27^ There are several excellent reviews addressing the challenges and limitations of click reactions, which enable practitioners to consider the choice of click reaction suitable for a given application.^27–30^ However, to our knowledge, no authoritative comparative study has been performed to assess the stability and general applicability of common click handles across a range of standard reaction conditions in order to inform click handle selection for a specific application.

Accordingly, in the current study, we describe a comprehensive assessment of the compatibility between commonly used click handles and bioorthogonal reaction conditions. Incompatible click handles and ligation condition combinations are identified, kinetic data of the undesired reaction between each incompatible combination are discerned, and a ranking of click handle stability within each ligation condition is compiled. Competing reaction products were isolated and characterized where possible, and long-term click handle stability studies for each combination (at room temperature and 4 °C over 4 weeks) were performed. Example click handles were also conjugated onto a model protein to assess their stability within a protein environment, which was compared with their small molecule stability.

## Results and Discussion

### Generation of Test Molecules Containing Common Click Handles

To investigate the stability and general properties of click handle functional groups, a literature survey was carried out to identify 14 representative click handles, which have extensive application in chemical biology and provide broad coverage of commonly employed click and bioorthogonal reaction classes (Figure 1). There are a large number of functional handles that are available for use in click reactions; therefore, a subset were selected for profiling in this work, which are representative of click handles commonly used in bioorthogonal applications. Fourteen click handle-containing constructs were subsequently generated, attached to a solubilizing linker consisting of a PEG-3 species, and a UV active pyrimidine core to aid liquid chromatography-mass spectrometry (LCMS) analysis (Figure 2; see the Supporting Information (SI) for synthesis details). The solubility of each construct was determined using a charged aerosol detector (CAD) solubility assay (SI Table S4).^31^ This confirmed that all constructs were sufficiently soluble for use in aqueous conditions, with measured solubilities between 123 and 694 μM in phosphate-buffered saline (PBS) at pH 7.4.

**Figure 2:**
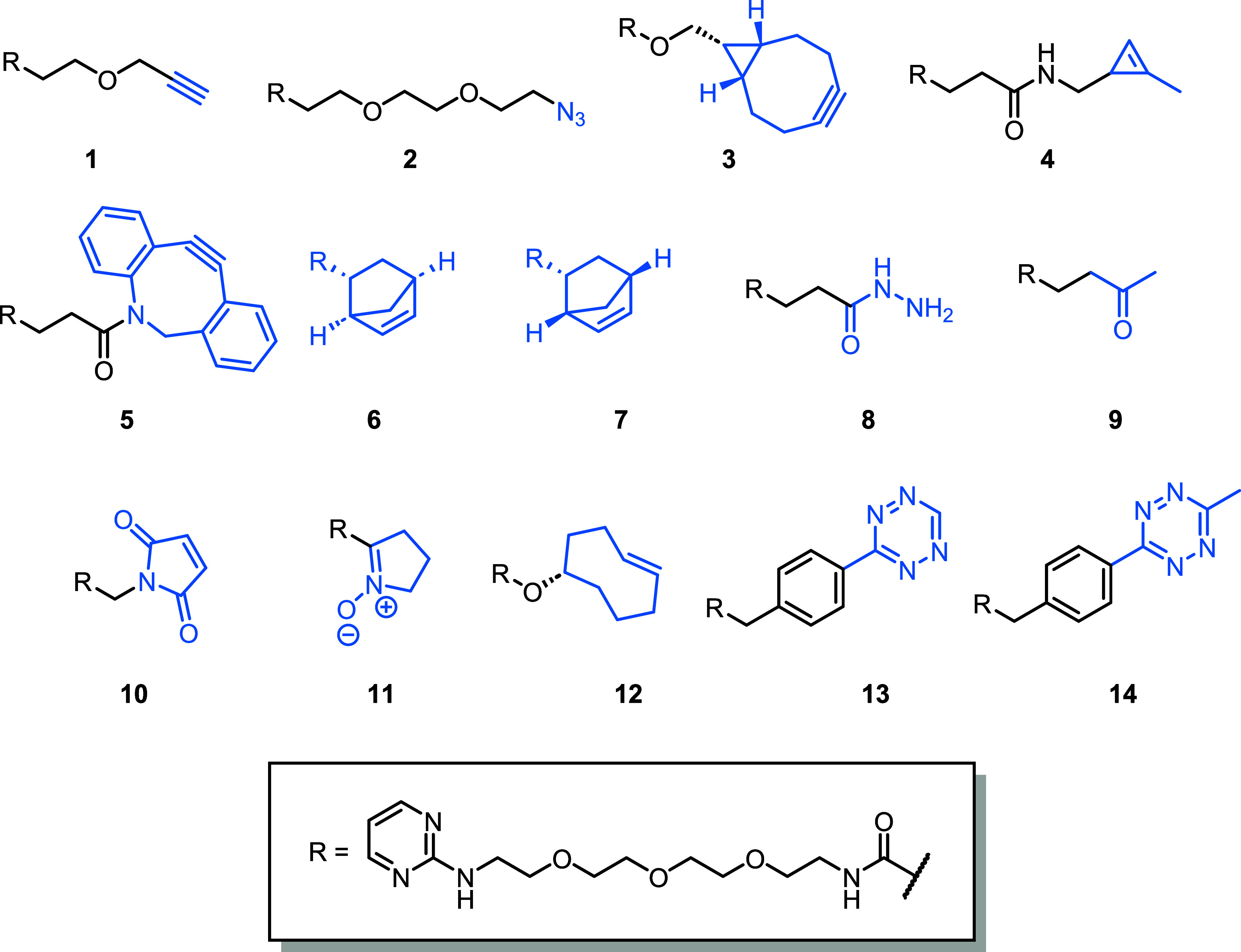
Structures of the 14 click handle-containing constructs profiled within this work. Click handles are highlighted in blue.

### Compatibility of Common Click Handles with Ligation Conditions

We initially assessed the stability of each click handle-containing compound in 12 ligation conditions: a range of six pH values, redox active glutathione (GSH), two common disulfide reducing agents (TCEP and dithiothreitol (DTT)), a CuAAC cocktail consisting of copper­(II) sulfate, tris­(benzyltriazolylmethyl)­amine (THPTA), and sodium ascorbate, the oxidizing agent dehydroascorbic acid (DHA), and the protein-denaturing reagent urea. These conditions were selected to span a range of potential conditions to which click handles may be exposed during frequently executed chemical biology workflows. Each combination of click handle substrate and ligation condition, in a 5 equiv excess if applicable, was incubated at 37 °C for 24 h prior to LCMS analysis, in which the ratio of each compound (P) relative to the internal standard (STD) was calculated.^32^ Combinations that showed statistically significant instability over a 24 h period were determined using Tukey’s honestly significant difference (HSD) multiple comparison procedure, applied to Box–Cox transformed linear regression models fit to the ratios. Models were fit to each compound separately with the 12 treatments and plate IDs included as categorical factors. Conditions were judged to be those with significantly lower mean ratios than the highest mean ratio, under the assumption that at least 1 of the 12 conditions are stable for each compound (Figure 3).^33,34^ Maleimide **10**, a handle commonly used in Michael addition reactions, was observed to be largely incompatible with the conditions examined here (Figure 3A). The CuAAC reaction mixture was also incompatible with a range of functional groups. Both norbornene isomers (**6** and **7**) and the terminal alkyne **1**, however, showed excellent stability across all ligation conditions assessed.

**Figure 3:**
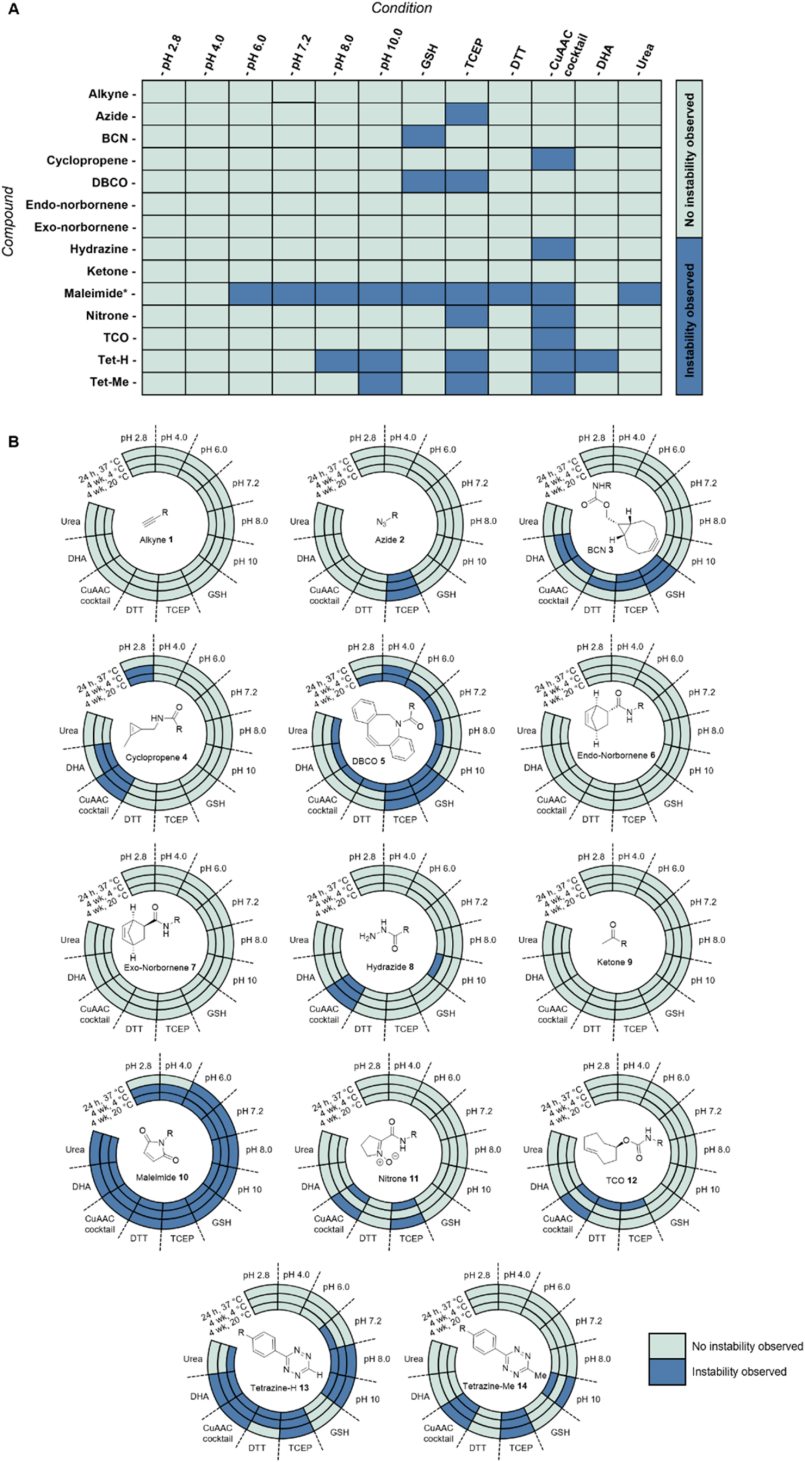
Compatibility of 14 click handles in 12 ligation conditions. (A) Compatibility of 14 click handles with 12 ligation conditions following a 24 h incubation at 37 °C in a 5 equiv excess of additive, if applicable. Click handle abundance was measured and interpreted by LCMS at 24 h time points, and the extent of material loss compared to a constant concentration of internal standard (across three sample replicates) was used as a measure of instability. Significance was calculated using the HSD procedure described in the text. Combinations that showed statistically significant instability are highlighted in dark blue. (B) Long-term stability of 14 click handles in 12 ligation conditions. The outer ring signifies 24 h stability at 37 °C (data from panel (A)), the middle ring 4 week stability at 4 °C, and the inner ring 4 week stability at 20 °C. 4 week stability data is a summary of two reaction replicates. *Maleimide **10** experiments were conducted after thiol capping due to the instability of maleimide **10** to the LCMS conditions; the capped maleimide species was analyzed as stable, except in the case of maleimide **10** and GSH, in which capping with GSH demonstrates instability of the maleimide to the condition. Maleimide **10** additive results were confirmed by kinetic studies carried out at pH 2.8, which demonstrated that maleimide **10** was unstable to each of these additives except for DHA, as is reflected in this figure. Azide **2** pH 7.2 showed instability in this initial study; however, follow-up studies showed that this was anomalous and therefore is shown as stable here.

We next examined long-term stability by assessing whether combinations in which the click handle showed no instability over 24 h remained stable over a 4 week period (Figure 3B). The majority of compounds that exhibited instability after 4 weeks do so at both room temperature and 4 °C. However, a group of combinations tested were stable for 4 weeks at subambient temperatures; however, they were unstable at room temperature, such as hydrazide **8** and base. Compounds that showed substantial instability over the 4 week study, including DBCO **5**, maleimide **10**, and tetrazine-H **13**, should therefore always be made immediately prior to use and stored accordingly.

The IEDDA reaction is reported to exhibit the most rapid click reaction kinetics;^2^ therefore, IEDDA click handles are often highly reactive species and may be anticipated to exhibit a degree of instability. Two heterocycles that can be used within IEDDA reactions were profiled in this study: tetrazine-H **13** and tetrazine-Me **14**. Tetrazine-H **13** provides faster IEDDA kinetics than tetrazine-Me **14** ^35^ and was unstable under more ligation conditions. A balance must therefore be developed between stability and click reactivity when selecting the optimal system for IEDDA reactions.^36,37^

The IEDDA dienophile partners profiled in this study include BCN **3**, cyclopropene **4**, norbornenes **6** and **7**, and TCO **12**. Within this substrate class, it was pleasing to observe a differentiation in the ligation conditions, which resulted in instability, offering a choice of click handle based upon stability in the selected reaction conditions. The norbornene species provide a more stable alternative to other strained species within IEDDA reactions, while BCN **3** displays poor long-term stability across several reaction conditions. However, norbornenes demonstrate much slower IEDDA kinetics than other strained species, further highlighting the compromise required between stability and the rate of click reaction.^2^

Compatibility studies of TCO **12** provided more variable data than the other compounds screened; however, the data were still statistically valid. It was demonstrated that this was not solubility driven (solubility ≥ 661 μM) and is potentially due to light-mediated isomerization of TCO to the *cis* isomer. It has been reported previously that copper and thiols can promote TCO isomerization, likely via a radical pathway, which can result in unusual kinetic profiles.^38,39^ However, a surprising finding here was the stability of TCO **12** to thiols GSH and DTT over a 24 h period. All of this should therefore be considered when selecting TCO as a click handle, and due to this variability in TCO **12** data compared with the other data collected, this substrate was omitted from further investigation.

Many of the strained species examined can also be used as dipolarophiles within SPAAC and SPANC reactions in addition to IEDDA, and therefore their differential stability can be harnessed within experimental design across a breadth of click reactions and applications. For example, DBCO **5** showed instability to TCEP over a 24 h period; therefore, if reducing conditions are required, BCN **3** would be a more appropriate strained alkyne choice, or an alternative reducing agent such as DTT should be employed.

SPAAC and SPANC 1,3-dipoles demonstrated reasonable stability across the ligation conditions explored, for example, azide **2** instability was only observed in the presence of TCEP, resulting in a Staudinger reduction, which is well documented in the literature.^40^ While both azide **2** and nitrone **11** were unstable in TCEP, they were stable to DTT, which is, therefore, the preferable reducing agent prior to SPAAC or SPANC reactions.

Interestingly, standard conditions used for CuAAC reactions showed incompatibility with around half of the click handles within 24 h, suggesting that experiments that carry out orthogonal click chemistries post-CuAAC reaction may not be successful. For example, dual-payload ADC synthesis often requires two or more orthogonal click chemistries, of which CuAAC is commonly used;^41^ therefore, the selection pool of available click chemistry pairs is limited by the stabilities of many click handles in CuAAC conditions.

### Kinetic Studies of Click Handle Instability

The click handle and ligation condition combinations, which demonstrated statistically significant undesired reactivity, were progressed to kinetic studies to confirm observations from the initial compatibility studies. The kinetic half-lives of each construct were determined and used to compare relative rates of click handle undesired cross-reactivity under specific ligation conditions. Each combination under investigation was incubated at 37 °C, and then the relative abundance of the intact click handle compound was monitored by LCMS, at 10 time points over a time frame appropriate for the kinetics of each combination. The first-order kinetics of each reaction were plotted, from which the half-life of the reaction was calculated. For compounds with half-lives of >24 h, an accurate half-life could not be calculated due to the duration of the kinetic time course. The kinetic half-lives of exemplar combinations are presented in Figure 4, calculated from three replicate kinetic experiments (kinetic plots for all combinations are provided in SI Figure S43).

**Figure 4:**
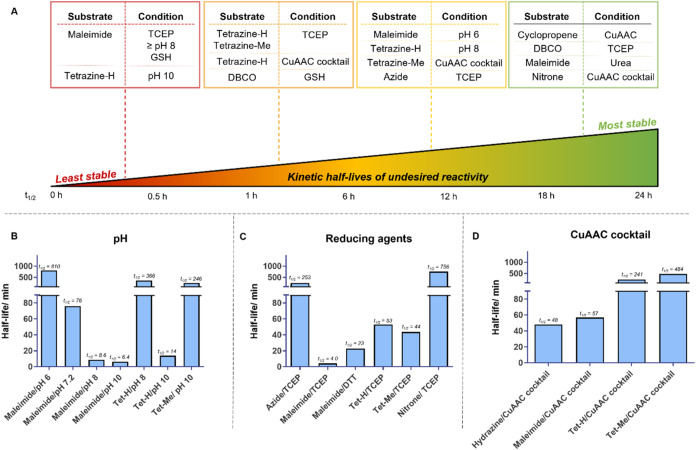
Kinetic half-lives measured for incompatible click handle and ligation condition combinations. (A) Approximate kinetic half-lives of undesired side reactions for exemplar click handle and ligation condition combinations. All kinetic experiments were carried out at pH 7.2 except maleimide **10** and tetrazine-H **13** reactions with additives, which were conducted at pH 2.8, due to instability of the click handle at pH 7.2. Kinetic half-lives measured for all click handles, which demonstrated instability over (B) the range of pH buffers tested, (C) the reducing agents tested, and (D) in the CuAAC cocktail conditions. Triplicate measurements performed for all data points; half-life values are shown above each bar in minutes.

Kinetic analyses identified one false positive in the initial compatibility studies, which could perhaps be expected with a data set of this size using a two-way analysis of variance (ANOVA) statistical model. This HSD procedure controls the experiment-wise error rate at 5%, resulting in an expected two incorrect declarations over the 42 HSD procedures carried out.^33,34^ Azide **2** did not in fact exhibit instability at pH 7.2, with a kinetic half-life of much greater than 24 h (Figure S43A), and therefore was omitted from further investigation.

We identified a robust corroboration between pH and rate of instability of maleimide **10**, as exemplified in Figure 4B, where the kinetic half-lives of hydrolysis of maleimide **10** in buffers ranging from pH 6 to 10 were plotted against pH. Therefore, maleimides should be used in mildly acidic conditions, unless confident that the desired reaction kinetics are more rapid than the undesired base-mediated hydrolysis. Due to the instability of maleimide **10** at pH 7.2 (Figure 4B), in contrast with its stability in pH 2.8 (Figure 3A), each kinetic analysis of reactions between maleimide **10** and all of the additives were conducted at pH 2.8. Maleimide **10** and DHA showed no reaction at pH 2.8 (SI Figure S43N), indicating that the instability previously observed was caused by the buffer of the reaction (PBS pH 7.2) and was not caused by the DHA itself. Maleimide **10** instability was observed in the presence of all other additives tested, however, at pH 2.8; therefore, this instability was indeed caused by the additives rather than the buffer conditions.

Tetrazine-H **13** also showed instability under basic conditions; therefore, kinetic studies were also carried out at pH 2.8, at which it is stable. This revealed that conditions found to induce tetrazine-H **13** cross-reactivity were indeed due to the additive conditions rather than the buffer conditions. It should also be noted that tetrazine-H **13** indicated much greater instability in basic conditions than tetrazine-Me **14**, which showed instability only in strongly basic conditions (pH 10), with a half-life of around 4 h (Figure 4A), which is potentially due to the increased substitution around the tetrazine ring.

Three of the constructs examined reacted with GSH: BCN **3**, DBCO **5**, and maleimide **10** (Figure 3A). Maleimide **10** rapidly reacted with the thiol with a measured 4 min half-life, as anticipated.^42^ Of the two strained alkynes assessed, which can be used in IEDDA (BCN only), SPAAC and SPANC click reactions, BCN **3** was significantly more stable to GSH than DBCO **5**, with a stark difference between their half-lives (∼6 h vs 71 min, respectively). DBCO **5** was also reactive to TCEP, as noted in the initial incompatibility studies; however, kinetic analyses demonstrated a half-life of greater than 24 h, suggesting that this combination may still be suitable for applications that take less than a day, provided the buffer does not contain any nucleophilic species in which the click handle is unstable to.

Three strained alkyne reactive species were also identified to show instability in the TCEP: both tetrazines **13** and **14** and azide **2** (Figure 4C). Interestingly, the kinetics of the undesired reactions of both tetrazines **13** and **14** were more rapid than that of azide **2**, despite the well-documented reaction between azides and phosphines.^40^ Maleimide **10** also demonstrated instability toward TCEP, with a kinetic half-life comparable to its undesired reactivity in GSH. Maleimide **10** also showed instability when using the second reducing agent tested, DTT, with a half-life of less than an hour. Therefore, if using an excess of either of these reducing agents prior to maleimide conjugation, a rigorous buffer exchange step is required to minimize the extent of the undesired side reaction. Ethylenediaminetetraacetic acid (EDTA) should ideally also be used within the reducing agent containing buffer solutions to limit reoxidation of the reduced disulfide bonds during these buffer exchanges.^43^

Of the species that were not stable to CuAAC conditions, maleimide **10** and hydrazide **8** reacted most rapidly, with half-lives of less than an hour (Figure 4D). Tetrazine-H **13** reacted at a slower rate, with a half-life of around 4 h, and tetrazine-Me **14** reacted with a half-life of around 8 h. The kinetics of these processes are slower than a typical CuAAC click reaction; however, residual CuAAC reagents may interfere with subsequent click reactions if these click handles are utilized.

### Identification and Characterization of Products from Undesired Click Handle Reactions

We subsequently performed reactions between incompatible click handles and ligation conditions on a larger scale in an effort to isolate and identify the undesired products. Details for all scaled-up reactions are included within the SI synthesis section, with key examples highlighted in Scheme 1. Expected products from the Staudinger reaction and thiol–yne reactions were isolated (Scheme 1A,B).

**Scheme 1:**
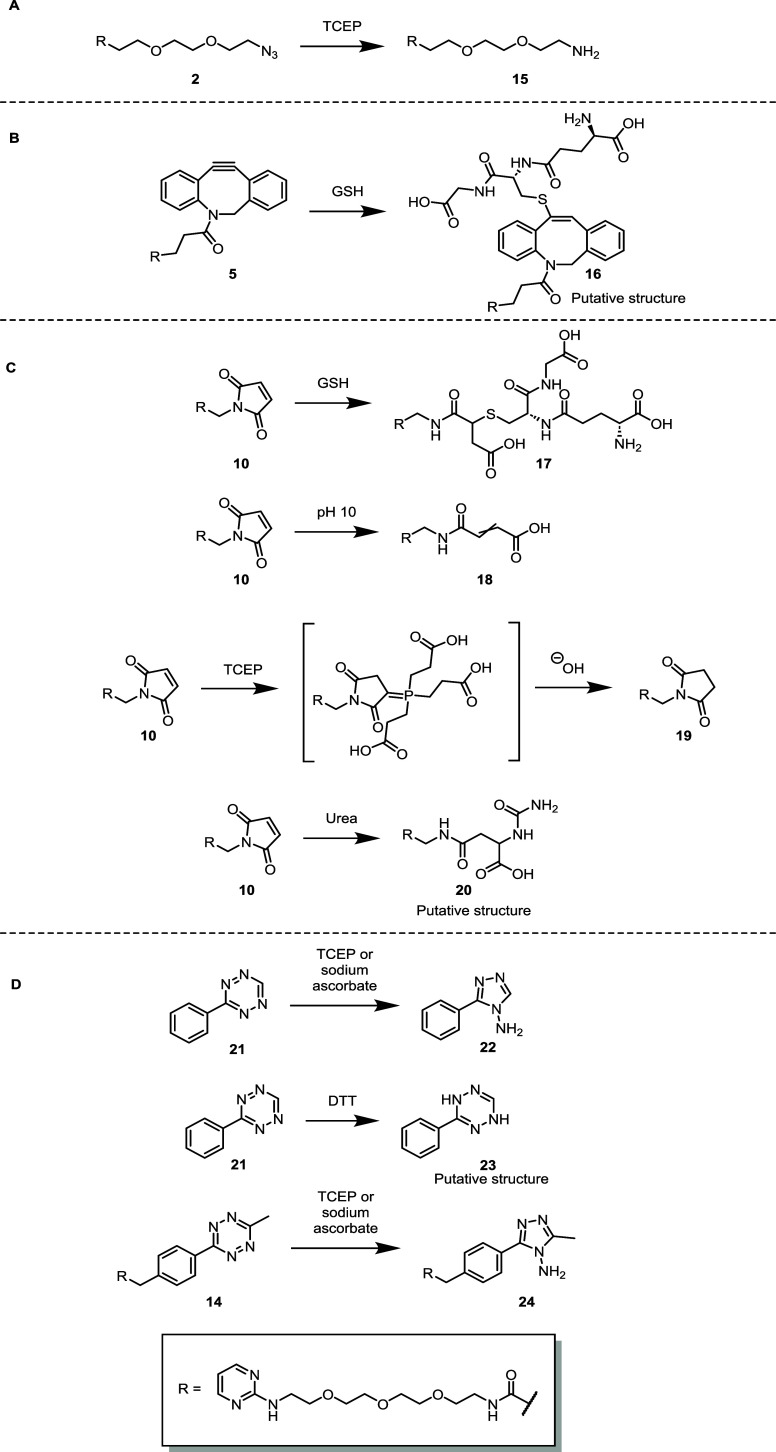
Isolated Products from a Selection of Larger-Scale Stability Studies^a^

a (A) Reaction of azide **2** with TCEP. (B) Reaction of BCN **3** and DBCO **5** with GSH. (C) Reaction of maleimide **10** with GSH, pH 10, TCEP, and urea. (D) Reaction of tetrazines **14** and **21** with TCEP, sodium ascorbate, and DTT.

We observed that following the addition of soft nucleophiles into maleimide **10**, the nucleophilic attack of water onto the imide carbonyl species resulted in ring opening, as is typically observed during maleimide conjugation reactions (Scheme 1C).^44^ Maleimide **10** also reacted with TCEP, as previously discussed in a study by Kantner et al.^45^ Both a species containing TCEP connected to the maleimide via a carbon–phosphorus double bond adjacent to a carbonyl and the hydrolyzed form were observed throughout our study. The phosphorus-containing species was detected during kinetic analysis; however, the hydrolyzed species was isolated upon scale-up, suggesting that the purification conditions used triggered the release of phosphorus from the construct. Both LCMS and NMR analyses of the reaction product when maleimide **10** was incubated in the presence of urea suggested a potential addition of urea into the enone, followed by hydrolysis of the ring forming **20**.

Due to the large number of conditions under which tetrazine-H **13** was shown to be unstable, larger-scale reactions were carried out instead using tetrazine **21**, which contained the click handle alone, as a more significant quantity of material was available for experimentation (Scheme 1D). Tetrazine **21** was subjected sequentially to different CuAAC cocktail components to determine which component caused the cross-reactivity. This was determined to be sodium ascorbate, after the addition of which a 2 Da mass increase was reported via LCMS analysis, corresponding to a reduction. Earlier compatibility studies suggested that reaction with TCEP also provided a compound with the same mass with varying degrees of conversion over a 24 h period (Figure 4). NMR analysis revealed that the product structure of the reaction between tetrazine-H **21** and both reducing agents was triazole **22** (Scheme 1D). It is known that tetrazines can rearrange in this manner;^46^ however, to our knowledge, this is the first report of the triazole product formed under these conditions.

Tetrazine-Me **14** showed a similar reactivity, undergoing ring contraction to the corresponding methyl triazole **24**. However, reaction kinetics varied depending on the reducing agent used, when compared with tetrazine-H **13**. Tetrazine-H **13** reacted most rapidly in sodium ascorbate compared with TCEP, with the respective half-lives differing by around 3 h (241 min vs 53 min, respectively), while tetrazine-Me **14** reacted much more rapidly in TCEP than sodium ascorbate, with half-lives differing by over 7 h (44 min vs ∼8 h, respectively). This suggests that if reducing agents are required prior to an IEDDA reaction, they should be chosen according to the substituents present on the tetrazine ring.

Although neither tetrazine analogues **13** or **14** reacted with DTT over the time frame of the compatibility experiments (Figure 3A), a large-scale reaction between tetrazine **21** and DTT was carried out to assess whether DTT also causes a ring contraction observed previously with TCEP, but on a much slower time scale. LCMS and NMR analyses suggested that the ring contraction previously observed did not occur using DTT. Instead, the data suggested a simple reduction of the tetrazine to form dihydrotetrazine **23** (Scheme 1D); however, isolation was difficult, potentially due to further degradation or reoxidation to the tetrazine occurring.

Tetrazines **13**, **14**, and **21** also showed instability under basic conditions, resulting in the production of at least seven compounds in the case of tetrazine-H **21** (reaction details can be found in the SI in the synthesis of click substrates section).

### On-Protein Stability Studies

Given the potential influence of protein environment upon the reactivity of small molecules and that a major application of click chemistry is within biological settings, we further assessed the stability of two example substrates, which can be used in IEDDA reactions, following protein conjugation (Figure 5A). Click handles that were previously identified to show instability under different conditions were selected (BCN, unstable to GSH over 24 h and tetrazine-Me, unstable to pH 10, TCEP and CuAAC cocktail over 24 h) to assess whether similar reactivity is observed when the click handles are ligated to a model protein.

**Figure 5:**
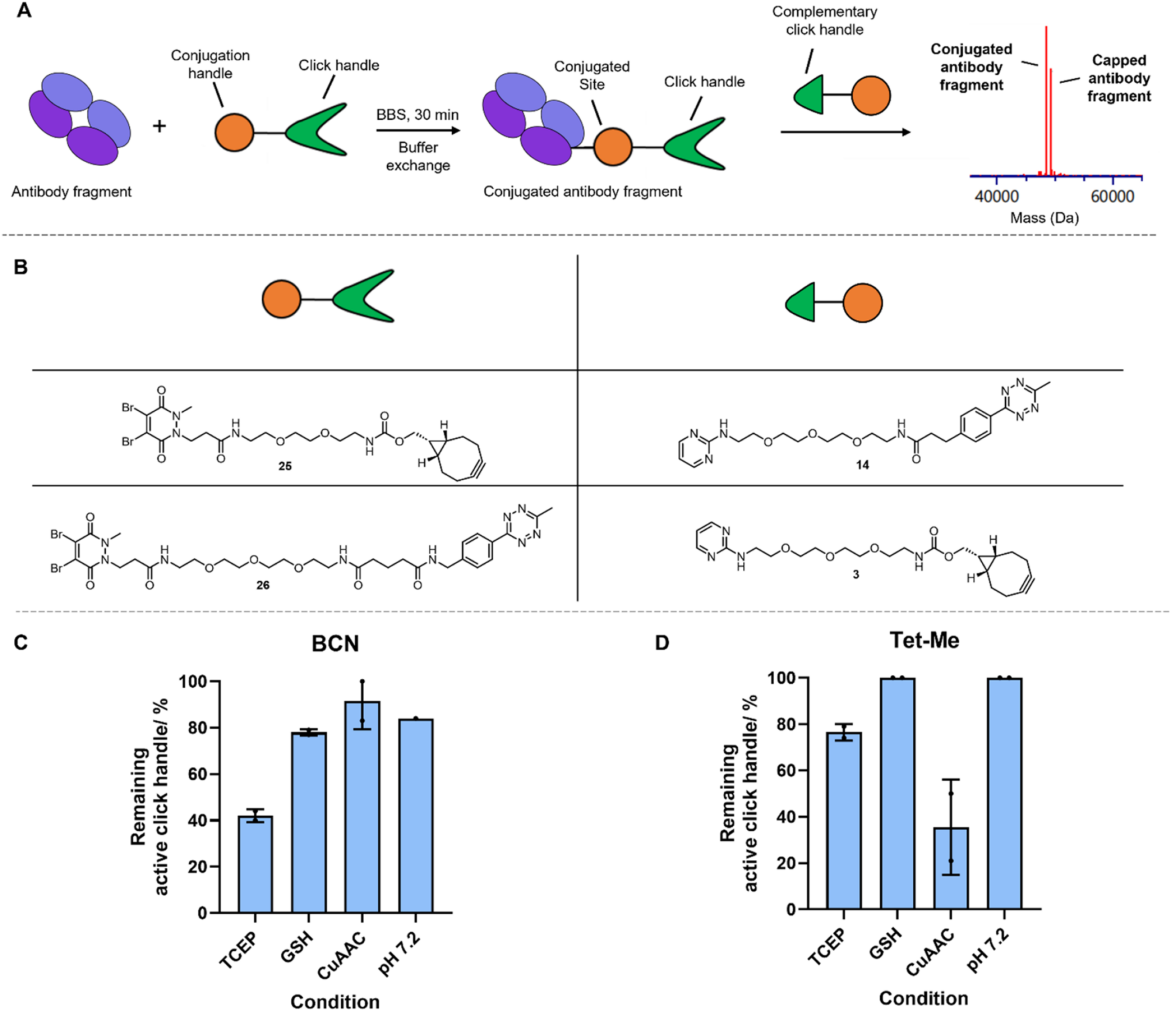
On-protein click handle stability study. (A) Procedure to determine the stability of exemplar click handles in a protein environment. (B) Structures of conjugation and click handle-containing species, alongside the complementary click handle-containing species used in the final step of the workflow. Percentage remaining BCN (C) and Tet-Me (D) active click handle following a 24 h incubation under selected bioorthogonal conditions. Error bars are equal to the standard deviation from *n* = 2. BCN in pH 7.2 was reported with no replicate due to low intensity of the LCMS spectrum.

BCN **25** and tetrazine-Me **26** substrates were synthesized (Figure 5B), containing a dibromopyridazinedione handle, which is a cysteine targeting group that can “rebridge” a reduced disulfide bond.^47^ An antibody fragment (Fab) was reduced using TCEP, buffer-exchanged to remove any residual reducing agent and then “rebridged” using the pyridazinedione compounds to generate two click handle-containing Fab conjugates. Each of these conjugates was subjected to four different ligation conditions overnight (Figure 5C,D). These conditions were selected to cover a range of stable and unstable combinations, as seen under small molecule conditions (Figure 3). Following a 24 h incubation at 37 °C, each Fab conjugate was capped with an excess of its corresponding click handle and then analyzed by intact protein mass spectrometry to determine conversion to capped Fab conjugate. Any uncapped Fab conjugate was associated with the instability of the click handle, and an estimate of percent cross-reactivity was calculated from the relative signal intensities of the Fab conjugate, containing inactive click handle, versus the capped Fab conjugate species.

As expected from the small molecule stability studies, tetrazine **26** was stable at pH 7.2, and to GSH, and showed full conversion to capped Fab conjugate (Figure 5D). Additionally, BCN **25** showed instability to GSH, as observed in a small molecule setting (Figure 5C vs Figure 3A). However, BCN **25** also showed instability at pH 7.2, CuAAC conditions, and TCEP. In the case of TCEP, this was to a greater extent than to GSH, whereas it had been stable in this condition previously, except for over a 4 week period at room temperature (Figure 3B). This suggests that a protein environment may sometimes encourage the instability of click handles to reagents they are stable to over a comparable time frame in a small molecule setting.

Tetrazine **26** was unstable under TCEP and CuAAC conditions in a protein environment, as well as in a small molecule setting (Figures 5D and 3). However, a greater degree of instability was observed under CuAAC conditions than TCEP in a protein environment, contrasting with the kinetic rankings of tetrazine **14** as a small molecule (Figures 5D and 4). Similar levels of remaining active click handle were observed for both tetrazines **14** and **26** under CuAAC conditions; however, tetrazine **26** showed much greater stability to TCEP than tetrazine **14**. This could potentially suggest that the increased steric bulk around the click handle in some protein environments may protect the click handle from undesired cross-reactivity. The lack of an IEDDA reaction observed following incubation of conjugated tetrazine **26** with TCEP and CuAAC conditions suggests that the aminotriazole product formed under these conditions is unable to react within IEDDA reactions.

### Decision Trees and Recommendations

After reviewing primarily click handle stability within the reaction conditions tested in this work but also reported click reaction kinetics (compiled in Luu et al.),^27^ in addition to click handle solubility (SI Table S7), we constructed decision trees to inform on recommended click handles within each click reaction class for any given application (Figure 6). These decision trees, in conjunction with Figure 3, can be used to assess the compatibility of each of the click handles used within this study with commonly used reagents within bioorthogonal workflows. We employed the decision trees in Figure 6 to determine our recommended click handles for a selection of different exemplar applications, which are outlined in greater detail in the SI, for example, to produce chemically linked bispecific antibodies or label cell surface biomolecules.^9,10,48,49^ Pleasingly, our recommendations aligned well with the click reaction classes and click handles used within the exemplar click chemistry applications, and in some cases, additional substrates were also highlighted as suitable for use within these applications. This suggests that the application of this guide can result in the rapid and facile selection of appropriate click reactions and click handles for a given purpose, which therefore expedites experimental design within the field, obviating the need for additional experimental activities.

**Figure 6:**
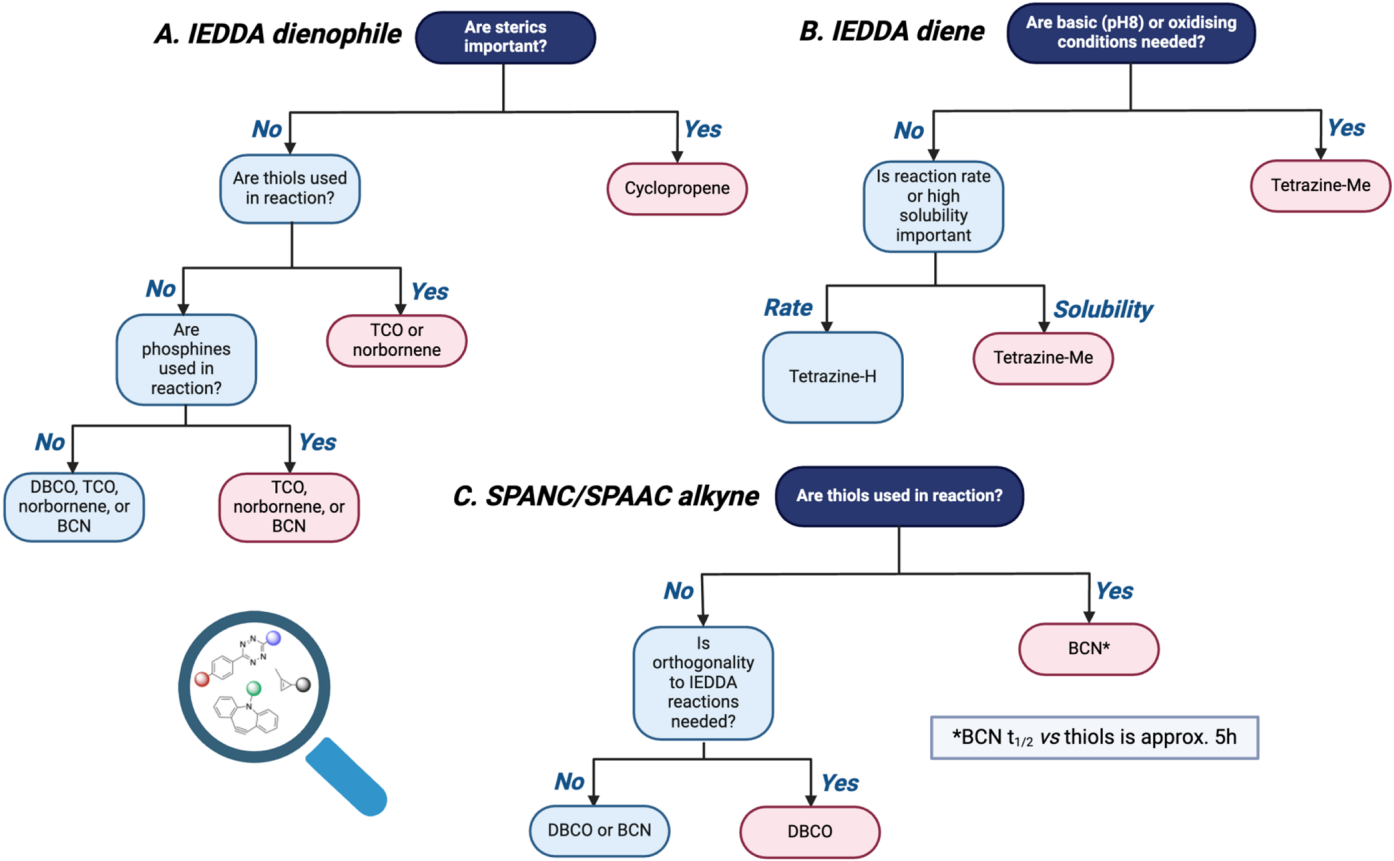
Decision trees to guide the selection of the most suitable: (A) dienophile for an IEDDA application. (B) Diene for an IEDDA application. (C) Strained alkyne for an SPAAC or SPANC application.

Prior to using the decision trees in Figure 6, users should consider the following questions using the stability data in Figures 3 and 4, alongside reaction kinetics data^27^ and solubility data (Table S7) to determine the most suitable click reaction for an application.

Are the click handles required to be present in any of the following conditions: pH ≥ 8, cell media, GSH-containing buffer, oxidizing conditions, or protein-denaturing conditions?

If any of these conditions are required, Michael additions are not a suitable choice for the click reaction.

Is CuAAC planned as an additional click reaction within the workflow?

All of the click reactions tested in this study use at least one click handle that is incompatible with the CuAAC conditions. If such conditions are required, we recommend carrying out the CuAAC reaction as the final step in the workflow, and that Figure 1 should be considered to assess orthogonality between click handles.

Is a reduction step required in the workflow?

Due to the incompatibility of many click handles with TCEP, we recommend carrying out a buffer exchange prior to the click reaction to enable the use of click handles, which are incompatible to TCEP, as shown in Figure 3A.

## Conclusions

Challenges that may limit the utility of click chemistry have been identified, and key parameters to consider when selecting appropriate click handles are presented through profiling 14 commonly used handles. Although the individual reagents are considered to be bioorthogonal, this work has shown that many are not orthogonal to each other, placing limitations on their use in sequential click processes, e.g., in the preparation of ADCs and related constructs. Incompatible combinations of click handles and common ligation conditions have been identified, the kinetic half-life of each of these undesired side reactions was determined, and where possible, the undesired side products were identified. Exemplar click handles were also conjugated onto an antibody fragment to explore their stability within a protein environment. We envisage that this comparative study will provide a roadmap to enable the expedient selection of the most appropriate click handles for any bioorthogonal application to maximize experimental success and ultimately benefit research in the vast range of disciplines that utilize click chemistry.

## Supplementary Material



## Notes

Supplementary figures; synthetic procedures of all compounds; experimental methods; statistical analysis; spectroscopic data; and additional cited references (PDF)

## Notes

The authors have cited additional references within the Supporting Information.^50–53^

## Abbreviations

ADC: antibody drug conjugate
BBS: borate-buffered saline
BCN: bicyclononyne
CAD: charged aerosol detector
CuAAC: copper-catalyzed azide–alkyne cycloaddition
DBCO: dibenzocyclooctyne
DHA: dehydroascorbic acid
DTT: dithiothreitol
Fab: antibody fragment
GSH: glutathione
HSD: honestly significant difference
IEDDA: inverse electron demand Diels–Alder
LCMS: liquid chromatography-mass spectrometry
mAb: monoclonal antibody
PBS: phosphate-buffered saline
SPAAC: strain-promoted azide–alkyne cycloaddition
SPANC: strain-promoted alkyne–nitrone cycloaddition
TCEP: tris­(2-carboxyethyl)­phosphine
TCO: *trans*-cyclooctene
THPTA: tris­(benzyltriazolylmethyl)­amine
