# Supplementary material for: Comparative Study of Click Handle Stability in Common Ligation Conditions
Source: Bioconjug Chem. 2025 Apr 27;36(5):1054–65. doi: 10.1021/acs.bioconjchem.5c00095 (PMC12100641; doi:10.1021/acs.bioconjchem.5c00095)
Supplement: Supplementary file 1 [file bc5c00095_si_001.pdf]

Supporting Information

A Comparative Study of Click Handle Stability in Common Ligation Conditions

Caitlin Fawcett, [a], [b] *Joe Watson,*[b] *Stephen Richards,*[b] *Alfred E. Doherty,*[a], [b] *Hikaru Seki,*[b] *Elizabeth A. Love,*[b] *Charlotte H. Coles,*[b] *Diane M. Coe,*[b] *and Craig Jamieson.**[a]

[a] C. Fawcett, A. E. Doherty and C. Jamieson

Pure and Applied Chemistry, University of Strathclyde, Thomas Graham Building, 295 Cathedral St, Glasgow G1 1XL

E-mail: craig.jamieson@strath.ac.uk

[b] C. Fawcett, H. Seki, A. E. Doherty, J. Watson, S. Richards, E. A. Love, C. H. Coles, D. M. Coe

Research Technologies, GSK, Gunnels Wood Rd, Stevenage SG1 2NY, United Kingdom, Stevenage SG1 2NY

Table of Contents

1. Materials and methods 2

1.1 General 2

1.2 Buffer details 2

1.3 Chrom LogD7.4 3

1.4 CAD Solubility Experimental Conditions 3

1.5 Protein analytics 3

2. Experimental 4

2.1 Synthesis of click substrates 5

2.2 Synthesis of products of reactions between incompatible substrates 15

2.3 Synthesis of substrates for on-protein stability studies 25
2.4 Compatibility studies general procedure 28
2.5 Compatibility studies with capping general procedure 28
2.6 Statistics for compatibility studies 29
2.7 Long term stability studies statistical analysis 34
2.8 Kinetic studies 44
2.9 Solubility assessment 50
2.10 Fab generation 52
2.11 On protein stability assessment procedure 52
2.12 Exemplar applications of click chemistry in which decision trees are used to select optimal click handles 53
2.13 Copies of NMR spectra 55
2.14 Copies of protein analysis data 90

Materials and methods

General

All reagents and solvents were purchased from commercial suppliers including Sigma Aldrich, Enamine and BroadPharm, and were used without further purification. All chemical reactions were carried out under air unless stated otherwise. Reactions which were heated kept the temperature maintained at the desired temperature using a thermometer-controlled heating mantle. Rf values were reported when LCMS data was not available due to a lack of chromophore or instability of products to LCMS conditions.

Flash column chromatography was carried out using the Teledyne ISCO CombiFlash® Rf+ apparatus with RediSep® silica cartridges (normal-phase), Biotage® SNAP KP-C18 cartridges (reverse-phase) or an EZ Prep® column (preparatory HPLC). Eluent conditions are stated in a form describing a gradient of the minor solvent (e.g. EtOAc) in the major solvent (e.g. cyclohexane).

Mass Directed Auto Preparative HPLC (MDAP) was performed on Xselect C_{18} columns (150 mm x 30 mm, 5 µm packing diameter) with either formic acid, ammonium bicarbonate or TFA modifiers at ambient temperature. The gradient employed was selected from one of five pre-set methods, with a gradient between 0.1 % v/v solution of modifier in water and 0.1 % v/v solution of modifier in acetonitrile. The UV wavelength detection range was between 210 nm to 350 nm. Mass spectrometry was performed by Waters ZQ ionisation, by alternate-scan positive and negative electrospray.

NMR spectra were recorded on a Bruker AV 500 MHz or a Bruker AV 400 MHz. Chemical shifts (δ) are reported in ppm and coupling constants (*J*) are in Hz. The following abbreviations are used for multiplicities: s = singlet; d = doublet; t = triplet; q = quartet; quint. = quintet; dd = doublet of doublets; dt = doublet of triplets; m = multiplet; br = broad. Residual solvent signals not reported.

Liquid Chromatography Mass Spectrometry (LCMS) methods used for reaction monitoring and final purity analysis are referred to by the modifier used (formic acid or high pH). The analysis was conducted on a Waters AcquityTM UPLCTM I-Class Plus instrument equipped with a BEH column (50 × 2.1 mm, 1.7 µm packing diameter), using alternate-scan positive and negative electrospray ionisation. Analytes were detected by UV absorption in the range 210 – 350 nm. Two methods were used: Formic – 40 °C, 1 mL min-1 flow rate, linear gradient 1 → 97% v/v MeCN (containing 0.1% v/v formic acid) in water (containing 0.1% v/v formic acid) over 1.5 minutes, then remaining at 97% of the MeCN mixture for 0.4 minutes, before increasing to 100% over 0.1 minutes. High pH (HpH) – 40 °C, 1 mL min-1 flow rate, linear gradient 1 → 97% v/v MeCN in aqueous ammonium bicarbonate (10 mM, adjusted to pH 10 with aqueous ammonia) over 1.5 minutes, then remaining at 97% MeCN for 0.4 minutes, before increasing to 100% over 0.1 minutes.

IR spectra were obtained on a Perkin Elmer Spectrum One spectrometer. Absorption frequencies (ν_{max}) are reported in wavenumbers (cm^{-1}).

TLC was performed using pre-coated silica on aluminium backed plates. TLC plates were visualised by $KMnO_4$ dissolved in H_2O, ninhydrin dissolved in *n*-butanol with acetic acid or UV.

HRMS was carried out by the internal service at GSK Stevenage, using a Waters Xevo® G2-XS QToF instrument under conditions of positive electrospray ionisation.

Buffer details

**Buffer name**	**Buffer composition**
Acetate buffer pH 2.8	300 mM acetic acid @ pH 2.8
Citric buffer pH 4.0	34 mM sodium citrate, 66 mM citric acid @ pH 4.0
Acetate buffer pH 6.0	100 mM sodium acetate, 6 mM acetic acid @ pH 6.0
PBS pH 7.2	Dulbecco's phosphate buffered saline @ pH 7.2
BBS pH 8.0	100 mM boric acid, 25 mM sodium borate, 75 mM sodium chloride @ pH 8.0
Carbonate buffer pH 10.0	8.2 mM sodium carbonate, 1.8 mM sodium bicarbonate @ pH 10.0

Table S1. *Composition of buffers used within click handle stability studies*

Chrom LogD*7.4*

The Chromatographic Hydrophobicity Index (CHI) values were measured using a Waters Acquity UPLC System equipped with a Acquity PDA detector, using reversed phase HPLC column (50 x 2 mm, 3 µM Gemini NX C18, Phenomenex, UK) with fast acetonitrile gradient at starting mobile phase of 50

mM ammonium acetate at pH 7.4. The sample was run at a flow rate of 0.75 mL/min, with an injection volume of 0.75 µL. The obtained chromatograms were analysed using Masslynx Version 4.2. CHI values are derived directly from the gradient retention times using calibration parameters for standard compounds. The CHI value approximates to the volume % organic concentration when the compound elutes. CHI is linearly transformed into Chrom LogD by least-square fitting of experimental CHI values to calculated ClogP values for over 20 k research compounds using the following formula: Chrom LogD = 0.0857CHI-2.00. The average error of the assay is ±3 CHI unit or ±0.25 Chrom LogD.

CAD Solubility Experimental Conditions

The kinetic solubility assay was measured by dilution of reagent (5 µL of 10 mM DMSO stock solution) with phosphate buffered saline (95 µL, pH 7.4) and equilibrating for 1 hour at room temperature. After which time, the mixture was filtered through Millipore Multiscreen HTS-PCF filter plates (MSSL BPC). The filtrate is quantified using a suitably calibrated Thermo Scientific Dionex Ultimate 3000 UHPLC connected with a Corona VEO Charged Aerosol Detector. A UPLC column is used (Acquity UPLC CSH C18 1.7 µm, 2.1 x 50 mm), with a flow rate of 0.75 mL/min. The injection volumes are 0.5 µL for DMSO samples and 2.5 µL for the aqueous filtrates. The obtained chromatograms were analysed using Chromeleon Version 7.2.10.ES. The standard error of the CAD solubility determination is ±30 µM, the upper limit of the solubility is 500 µM when working from 10 mM DMSO stock solution.

Protein analytics

Protein concentration was determined by measuring the absorbance at λ = 280 nm using a NanoDrop 1000 spectrophotometer.

aSEC data was collected using a 1260 Infinity II LC system (Agilent), fitted with an Acquity UPLC BEH SEC 4.6 x 150 mm column (Waters). 2 µg of protein was used per injection. Absorbance at 214 nm was measured and used to quantify the relative abundance of Fab monomer and other species. Observed peak integrations were manually re-adjusted where necessary, then exported manually via ChromView for Chemstation v2.4.3 (MindGarden, Inc.) to generate a compiled data table. Data analysed was the wavelength data using the A214 peptide backbone.

Intact MS was performed using a Waters Acquity UPLC pump system connected with a TUV detector with Acquity RDa Waters Mass Spectrometer and using column: Waters BioResolve 2.1x50mm column. Mobile phase A was Water + 0.1% Formic acid , mobile phase B was MeCN + 0.1% Formic acid. The sample was run at a flow rate of 0.5 ml/min. The obtained m/z spectra was deconvoluted and analysed using the Unifi Version 1.9.4.053. 10 µL of protein sample was prepared at 1 mg/mL. Bondbreaker TCEP (1 µL) solution was added to the samples if a reduced Fab spectrum was required, and the mixture was incubated for 15 min.

Centrifugation was carried out on a 1.5 mL Eppendorf tube scale using a 5415C Eppendorf centrifuge.

SDS-PAGE was carried out using Invitrogen NuPage 4-12% Bis-Tris gels. Samples were mixed with SDS non-reducing loading buffer (NuPAGE LDS sample buffer 4x) or reducing loading buffer (NuPAGE LDS sample buffer + 0.5 uL bondbreaker TCEP). Samples were run at a constant current (120 mA) and voltage (180 V) for 40 min in Novex NuPage MES SDS running buffer (20x). Gels were stained with InstantBlue® Coomassie protein stain and de-stained with H_2O. The molecular ladder used was either the SeeBlue Plus 2 prestained protein standard or Novex Sharp pre-stained protein standard.

Gel imagery was obtained using a BioRad GeldocTM EZ Imager (White Light Sample Tray) and processed using Image Lab: Exposure Time (sec) 0.273 (Auto - Intense Bands), Application Instant Blue, 33 Dark Type Referenced, Ref. Bkgd. Time (sec) 10, Flat Field Applied, Serial Number 735BR07211, Software Version 6.1.0.07, Illumination Mode White Transillumination.

Experimental

Synthesis of click substrates

Synthesis of carbamate S1[35]

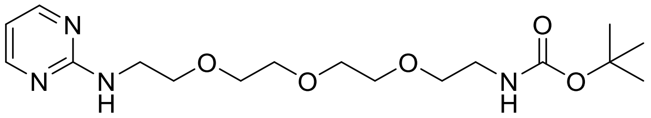

Cesium carbonate (3.1 g, 9.6 mmol) was added to 2-chloropyrimidine (518 mg, 4.52 mmol) and *tert*-butyl (2-(2-(2-(2-aminoethoxy)ethoxy)ethoxy)ethyl)carbamate (1.28 g, 4.38 mmol) in 1,4-dioxane (22 mL) and the reaction mixture was stirred at 100 degC for 30 h. The reaction mixture was filtered over celite, washed with 3:1 EtOAc/EtOH and the filtrate was concentrated under reduced pressure. The crude was dissolved in the minimum amount of DCM and MeOH then was filtered, redissolved in the same solvents, and purified by normal phase column chromatography with a gradient of 0-100% EtOAc in cyclohexane immediately followed by a gradient of 0-100% 3:1 EtOAc:EtOH in EtOAc. Appropriate fractions were combined and concentrated under reduced pressure to afford *tert*-butyl (2-(2-(2-(2-(pyrimidin-2-ylamino)ethoxy)ethoxy)ethoxy)ethyl)carbamate (1.07 g, 66%) as an orange oil.

LCMS HpH retention time = 0.84 min, [M+H] = 371.17 (98%)

^{1}H NMR (400 MHz, CHLOROFORM-d) δ ppm 8.22 - 8.32 (m, 2 H) 6.47 - 6.55 (m, 1 H) 5.67 - 5.83 (m, 1 H) 5.24 - 5.44 (m, 1 H) 3.66 (s, 12 H) 3.52 - 3.57 (m, 2 H) 3.26 - 3.35 (m, 2 H) 1.44 (s, 9 H)

Synthesis of amine S2

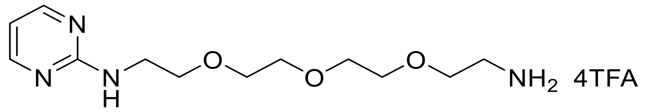

Tert-butyl (2-(2-(2-(2-(pyrimidin-2-ylamino)ethoxy)ethoxy)ethoxy)ethyl)carbamate (1.4 g, 3.89 mmol) was dissolved in TFA (6 mL, 80 mmol) and the reaction mixture was stirred at 1 h at rt. The reaction mixture was concentrated under a stream of nitrogen then dried under high vacuum overnight to afford N-(2-(2-(2-(2-aminoethoxy)ethoxy)ethoxy)ethyl)pyrimidin-2-amine, 4 trifluoroacetic acid salt (3.0 g, quant.) as an orange oil.

LCMS HpH retention time = 0.52 min, [M+H] = 271.15 (99%)

^{1}H NMR (400 MHz, DMSO-d6) δ ppm 8.33 - 8.43 (m, 2 H) 7.55 - 7.99 (m, 4 H) 6.65 - 6.75 (m, 1 H) 3.57 - 3.63 (m, 2 H) 3.51 - 3.57 (m, 10 H) 3.44 - 3.50 (m, 2 H) 2.92 - 3.02 (m, 2 H)

^{13}C NMR (101 MHz, DMSO-d6) δ ppm 159.8, 157.7, 109.9, 69.7 (2C), 69.6, 68.6, 66.6 (2C), 40.5, 38.6

HRMS (ESI+) Calculated for $C_{12}H_{23}N_4O_3^+$ $[M+H]^+$, 271.1765, found 271.1770 Da.

IR: 3400 (N-H), 1674 (C=O), 1182 (C-N), 1126 (C-O) cm^{-1}.

Synthesis of alkyne 1

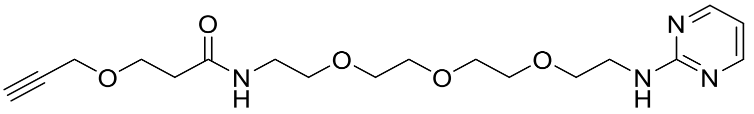

DIPEA (255 µL, 1.48 mmol) was added to a solution of 3-(prop-2-yn-1-yloxy)propanoic acid (52.1 mg, 407 µmol) and HATU (211 mg, 555 µmol) in DMF (1.8 mL) and the reaction mixture was stirred at rt for 5 min. *N*-(2-(2-(2-(2-aminoethoxy)ethoxy)ethoxy)ethyl)pyrimidin-2-amine (100 mg, 370 µmol) was added and the reaction mixture was stirred at rt for a further 1.5 h. The reaction mixture was directly purified by MDAP HpH method A and appropriate fractions were combined and concentrated under reduced pressure. The product was further purified by MDAP method A HpH and appropriate fractions were combined and concentrated under reduced pressure to afford 3-(prop-2-yn-1-yloxy)-*N*-(2-(2-(2-(2-(pyrimidin-2-ylamino)ethoxy)ethoxy)ethoxy)ethyl)propenamide (21.3 mg, 15%) as an orange gum.

LCMS HpH retention time = 0.67 min, [M+H] = 381.30 (95%)

^{1}H NMR (400 MHz, $CDCl_3$) δ ppm 8.27 (d, *J* = 4.9 Hz, 2 H) 6.71 - 6.81 (m, 1 H) 6.50 - 6.56 (m, 1 H) 5.80 - 5.96 (m, 1 H) 4.15 - 4.19 (m, 2 H) 3.77 - 3.83 (m, 2 H) 3.66 (s, 12 H) 3.55 - 3.60 (m, 2 H) 3.43 - 3.49 (m, 2 H) 2.47 - 2.51 (m, 2 H) 2.45 - 2.47 (m, 1 H)

^{13}C NMR (101 MHz, $CDCl_3$) δ ppm 171.2, 162.2, 158.2, 110.8, 79.8, 75.0, 70.9 (2C), 70.7, 70.6, 70.2, 70.1, 66.4, 58.6, 41.5, 39.5, 37.2

HRMS (ESI+) Calculated for $C_{18}H_{29}N_4O_5^+$ $[M+H]^+$, 381.2121, found 381.2146 Da.

IR: 3278 (N-H), 2140 (C≡C), 1645 (C=O), 1281 (C-N), 1088 (C-O) cm^{-1}.

Synthesis of azide 2

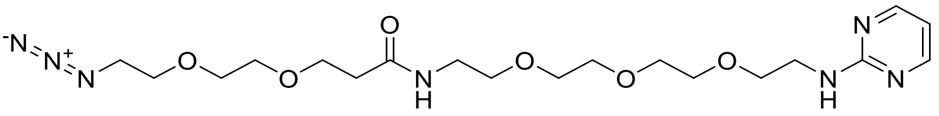

DIPEA (127 µL, 740 µmol) was added to a solution of *N*-(2-(2-(2-(2-aminoethoxy)ethoxy)ethoxy)ethyl)pyrimidin-2-amine (100 mg, 370 µmol) and 2,5-dioxopyrrolidin-1-yl 3-(2-(2-azidoethoxy)ethoxy)propanoate (111 mg, 370 µmol) in DMF (1.8 mL) and the reaction mixture was stirred at rt for 40 min. The reaction mixture was directly purified by MDAP HpH method B. Appropriate fractions were combined and concentrated under a stream of nitrogen to afford 3-(2-(2-azidoethoxy)ethoxy)-*N*-(2-(2-(2-(2-(pyrimidin-2-ylamino)ethoxy)ethoxy)ethoxy)ethyl)propenamide (70.8 mg, 42%) as a colourless gum.

LCMS HpH retention time = 0.73 min, [M+H] = 456.35 (98%)

^{1}H NMR (400 MHz, $CDCl_3$) δ ppm 8.21 - 8.30 (m, 2 H) 6.73 - 6.81 (m, 1 H) 6.48 - 6.54 (m, 1 H) 5.70 - 5.81 (m, 1 H) 3.71 - 3.75 (m, 2 H) 3.63 (m, 18 H) 3.53 - 3.57 (m, 2 H) 3.41 - 3.46 (m, 2 H) 3.34 - 3.39 (m, 2 H) 2.40 - 2.49 (m, 2 H)

^{13}C NMR (101 MHz, $CDCl_3$) δ ppm 171.2, 162.0, 157.8, 110.4, 70.5, 70.5, 70.4, 70.3, 70.2 (2C), 69.9, 69.8, 69.7, 67.3, 50.6, 41.1, 39.1, 36.9

HRMS (ESI+) Calculated for $C_{19}H_{34}N_7O_6^+$ $[M+H]^+$, 456.2565, found 456.2591 Da.

IR: 3352 (N-H), 2102 (N=N=N), 1644 (C=O), 1350 (C-N), 1095 (C-O) cm^{-1}.

Synthesis of BCN 3

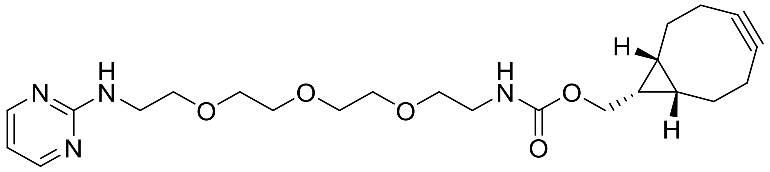

DIPEA (88.6 µL, 515 µmol) was added to a solution of *N*-(2-(2-(2-(2-aminoethoxy)ethoxy)ethoxy)ethyl)pyrimidin-2-amine (100 mg, 370 µmol) and *N*-[((1*R*,8*S*,9s)-bicyclo[6.1.0]non-4-yn-9-yl)methyloxycarbonyloxy]succinimide (100 mg, 343 µmol) in DMF (1.8 mL) and the reaction mixture was stirred at rt for 1 h. The reaction mixture was directly purified by EZ prep method B HpH modified gradient of 20-60% MeCN in HpH water. UV was changed to detect 300 nm only. Appropriate fractions were combined and concentrated under reduced pressure to afford ((1*R*,8*S*,9s)-bicyclo[6.1.0]non-4-yn-9-yl)methyl (2-(2-(2-(2-(pyrimidin-2-ylamino)ethoxy)ethoxy)ethoxy)ethyl)carbamate (35.2 mg, 23%) as a colourless oil.

LCMS HpH retention time = 0.98 min, [M+H] = 447.36 (97%)

^{1}H NMR (400 MHz, $CDCl_3$) δ ppm 8.26 - 8.39 (m, 2 H) 6.53 - 6.64 (m, 1 H) 5.49 - 5.62 (m, 1 H) 4.09 - 4.24 (m, 2 H) 3.62 - 3.72 (m, 14 H) 3.55 - 3.61 (m, 2 H) 3.34 - 3.43 (m, 2 H) 2.20 - 2.27 (m, 4 H) 1.52 - 1.66 (m, 2 H) 1.31 - 1.42 (m, 1 H) 0.88 - 1.00 (m, 2 H) One exchangeable proton not observed

^{13}C NMR (101 MHz, $CDCl_3$) δ ppm 157.6, 110.1, 98.5, 76.8, 76.7, 70.4, 70.23, 70.2, 70.1, 70.0, 69.9, 69.5, 40.8, 28.7, 21.1, 19.8, 17.5

HRMS (ESI+) Calculated for $C_{23}H_{35}N_4O_5^+$ $[M+H]^+$, 447.2602, found 447.2604 Da.

IR: 3332 (N-H), 1698 (C=O), 1247 (C-N), 1096 (C-O) cm^{-1}.

Synthesis of TMS cyclopropene S3

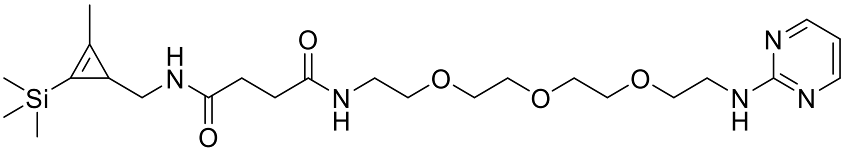

DIPEA (224 µL, 1.29 mmol) was added to a stirred solution of *N*-(2-(2-(2-(2-aminoethoxy)ethoxy)ethoxy)ethyl)pyrimidin-2-amine (182 mg, 673 µmol) and succinic anhydride (41.8 µL, 644 µmol) in DCM (2 mL) and the reaction was stirred at room temperature for 1.5 h. To the previous reaction mixture was added bis(2,5-dioxopyrrolidin-1-yl) carbonate (165 mg, 644 µmol) and the reaction was stirred at room temperature for 1 h. To the previous reaction mixture was added a solution of (2-methyl-3-(trimethylsilyl)cycloprop-2-en-1-yl)methanamine (100 mg, 644 µmol) in DCM (1.7 mL) and the reaction was stirred at room temperature for 1.5 h. The reaction was diluted with DCM and water and extracted. The aqueous layer was extracted with DCM x 3, then the organics were dried over a hydrophobic frit and concentrated under reduced pressure. The crude was purified by normal phase silica column chromatography with a gradient of 0-100% EtOAc in cyclohexane then 0-100% 3:1 EtOAc/EtOH in EtOAc. Appropriate fractions were combined and concentrated under reduced pressure to afford *N*1-((2-methyl-3-(trimethylsilyl)cycloprop-2-en-1-yl)methyl)-*N*4-(2-(2-(2-(2-(pyrimidin-2-ylamino)ethoxy)ethoxy)ethoxy)ethyl)succinimide (85.1 mg, 26%) as a colourless film.

LCMS HpH retention time = 1.00 min, [M+H] = 508.32 (98%)

^{1}H NMR (400 MHz, CHLOROFORM-*d*) δ ppm 8.22 - 8.31 (m, 2 H) 6.67 - 6.81 (m, 1 H) 6.49 - 6.56 (m, 1 H) 5.91 - 6.02 (m, 1 H) 5.76 - 5.88 (m, 1 H) 3.59 - 3.71 (m, 12 H) 3.54 - 3.59 (m, 2 H) 3.39 - 3.48 (m, 2 H) 3.15 - 3.24 (m, 1 H) 2.99 - 3.09 (m, 1 H) 2.45 - 2.55 (m, 4 H) 2.18 (s, 3 H) 1.39 - 1.46 (m, 1 H) 0.16 (s, 9 H)

^{13}C NMR (101 MHz, $CDCl_3$) δ ppm 172.6, 172.1, 162.5, 158.3, 136.1, 112.1, 110.8, 70.8 (2C), 70.6 (2C) 70.1, 46.7, 41.5, 39.7, 32.3, 32.1, 25.8, 19.5, 13.4, 1.4.

HRMS (ESI+) Calculated for $C_{24}H_{42}N_5O_5Si^+$ $[M+H]^+$, 508.2950, found 508.2962 Da.

IR: 1658 (C=O), 1644 (C=C), 1247 (C-N), 1098 (C-O), 696 (C-Si) cm^{-1}.

Synthesis of cyclopropene 4

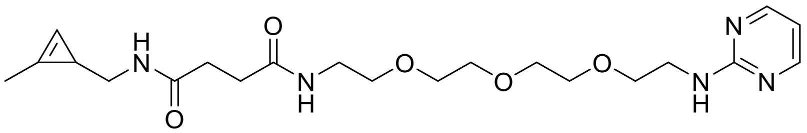

1 M TBAF in THF (335 µL, 335 µmol) was added to a solution of *N*1-((2-methyl-3-(trimethylsilyl)cycloprop-2-en-1-yl)methyl)-*N*4-(2-(2-(2-(2-(pyrimidin-2-ylamino)ethoxy)ethoxy)ethoxy)ethyl)succinamide (85.1 mg, 168 µmol) in THF (838 µL) and the reaction mixture was stirred at rt for 1.5 h. Additional TBAF (335 µL, 335 µmol) was added and the reaction mixture was stirred for 1 h. The reaction mixture was concentrated under reduced pressure then was purified by normal phase silica column chromatography with a gradient of 0-100% EtOAc in cyclohexane. Appropriate fractions were combined and concentrated under reduced pressure then dissolved in DMSO and purified by EZ prep method B HpH modified to 20-50% MeCN in HpH water. Appropriate fractions were combined and concentrated under reduced pressure to afford *N*1-((2-methylcycloprop-2-en-1-yl)methyl)-*N*4-(2-(2-(2-(2-(pyrimidin-2-ylamino)ethoxy)ethoxy)ethoxy)ethyl)succinamide (35.1 mg, 48%) as a colourless gum.

LCMS HpH retention time = 0.66 min, [M+H] = 436.38 (95%)

^1H NMR (400 MHz, $CDCl_3$) δ ppm 8.23 - 8.29 (m, 2 H) 6.55 - 6.59 (m, 1 H) 6.50 - 6.54 (m, 1 H) 3.66 (m, 12 H) 3.55 - 3.59 (m, 2 H) 3.42 - 3.47 (m, 2 H) 3.34 - 3.40 (m, 2 H) 2.50 (s, 4 H) 2.11 (d, J = 1.0 Hz, 3 H) 1.51 - 1.55 (m, 1 H). Two exchangeable protons not observed.

^{13}C NMR (101 MHz, $CDCl_3$) δ ppm 172.5, 172.2, 162.6, 158.3, 121.8, 110.9, 103.2, 70.9, 70.8, 70.6 (2C), 70.1 (2C), 45.5, 41.4, 39.5, 32.2 (2C), 20.1, 11.9

HRMS (ESI+) Calculated for $C_{21}H_{34}N_5O_5^+$ $[M+H]^+$, 436.2554, found 436.2566 Da.

IR: 3411 (N-H), 1688 (C=O), 1646 (C=C), 1123 (C-O) cm^{-1}.

Synthesis of DBCO 5

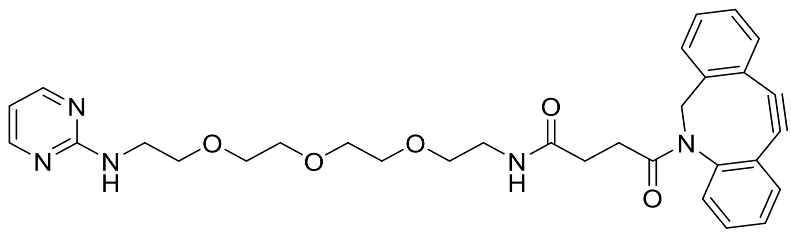

DIPEA (42.8 µL, 249 µmol) was added to a solution of *N*-(2-(2-(2-(2-aminoethoxy)ethoxy)ethoxy)ethyl)pyrimidin-2-amine (67.2 mg, 249 µmol) and dibenzocyclooctyne-*N*-hydroxysuccinimidyl ester (100 mg, 249 µmol) in DMF (1.8 mL) and the reaction mixture was stirred at rt for 2.5 h. Further DIPEA (21.6 µL, 124 µmol) was added and the reaction was stirred for 2 h. The reaction mixture was directly purified by EZprep method C modified to 25-80% MeCN in HpH water, with the UV changed to 300 nm only. Appropriate fractions were combined and concentrated under reduced pressure to afford dibenzocyclooctyne-*N*-(2-(2-(2-(2-(pyrimidin-2-ylamino)ethoxy)ethoxy)ethoxy)ethyl)succinamide (71.8 mg, 52%) as a milky gum.

LCMS HpH retention time = 1.03 min, [M+H] = 558.22 (96%)

^{1}H NMR (400 MHz, $CDCl_3$) δ ppm 8.23 - 8.30 (m, 2 H) 7.66 - 7.72 (m, 1 H) 7.51 - 7.57 (m, 1 H) 7.23 - 7.44 (m, 6 H) 6.49 - 6.54 (m, 1 H) 6.38 - 6.46 (m, 1 H) 5.92 - 6.01 (m, 1 H) 5.14 - 5.21 (m, 1 H) 3.64 (s, 12 H) 3.43 - 3.54 (m, 3 H) 3.31 - 3.39 (m, 2 H) 2.76 - 2.89 (m, 1 H) 2.41 - 2.56 (m, 1 H) 2.13 - 2.23 (m, 1 H) 1.90 - 2.04 (m, 1 H)

^{13}C NMR (101 MHz, $CDCl_3$) δ ppm 172.2, 172.0, 161.7, 157.8, 151.5, 148.1, 132.2, 129.4, 128.6, 128.1, 128.0, 127.6, 127.0, 125.4, 123.2, 122.5, 114.7, 110.3, 107.9, 70.5 (2C), 70.3, 70.2, 69.7, 69.7, 55.5, 41.2, 39.2, 31.2, 30.1

HRMS (ESI+) Calculated for $C_{31}H_{36}N_5O_5^+$ $[M+H]^+$, 558.2711, found 558.2704 Da.

IR: 3316 (N-H), 1655 (C=O), 1253 (C-N), 1108 (C-O) cm^{-1}.

Synthesis of norbornenes 6 and 7

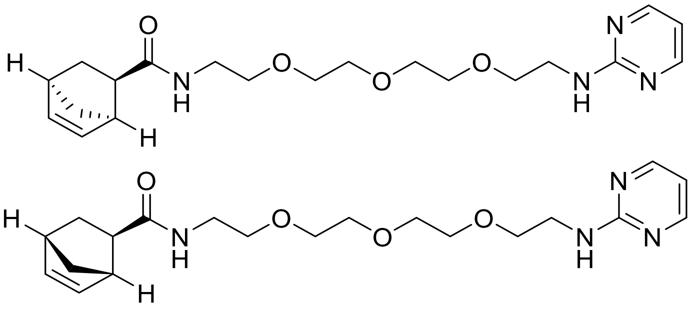

DIPEA (255 µL, 1.48 mmol) was added to a solution of bicyclo[2.2.1]hept-5-ene-2-carboxylic acid (56.2 mg, 407 µmol) and HATU (211 mg, 555 µmol) in DMF (1.8 mL) and the reaction mixture was stirred at rt for 5 min. *N*-(2-(2-(2-(2-aminoethoxy)ethoxy)ethoxy)ethyl)pyrimidin-2-amine (100 mg, 370 µmol) was added and the reaction mixture was stirred at rt for a further 1.5 h. The reaction mixture was directly purified by MDAP HpH method B and appropriate fractions were combined and concentrated under reduced pressure to afford (1*R*,2*R*,4*R*)-*N*-(2-(2-(2-(2-(pyrimidin-2-ylamino)ethoxy)ethoxy)ethoxy)ethyl)bicyclo[2.2.1]hept-5-ene-2-carboxamide (35.3 mg, 24%) and (1*S*,2*R*,4*S*)-*N*-(2-(2-(2-(2-(pyrimidin-2-ylamino)ethoxy)ethoxy)ethoxy)ethyl)bicyclo[2.2.1]hept-5-ene-2-carboxamide (14.7 mg, 10%) as colourless oils.

Endo-isomer

LCMS retention time = 0.79 min, [M+H] = 391.35

^{1}H NMR (400 MHz, $CDCl_3$) δ ppm 8.21 - 8.32 (m, 2 H) 6.47 - 6.57 (m, 1 H) 6.18 - 6.22 (m, 1 H) 5.94 - 5.99 (m, 1 H) 5.72 - 5.82 (m, 1 H) 3.65 (s, 12 H) 3.50 - 3.54 (m, 2 H) 3.37 - 3.42 (m, 2 H) 3.08 - 3.13 (m, 1 H) 2.86 - 2.89 (m, 1 H) 2.81 - 2.86 (m, 1 H) 2.61 - 2.68 (m, 1 H) 1.85 - 1.92 (m, 1 H) 1.38 - 1.43 (m, 1 H) 1.31 - 1.37 (m, 1 H) 1.23 - 1.27 (m, 1 H)

^{13}C NMR (151 MHz, $CDCl_3$) δ ppm 174.1, 161.9, 157.7, 137.3, 132.0, 110.3, 70.3 (2C), 70.1, 69.9, 70.1, 69.6, 49.7, 45.9, 44.5, 42.5, 40.9, 38.9, 29.5.

HRMS (ESI+) Calculated for $C_{20}H_{31}N_4O_4^+$ $[M+H]^+$, 391.2340, found 391.2357 Da.

IR: 3318 (N-H), 1644 (C=O), 1587 (C=C), 1243 (C-N), 1095 (C-O) cm^{-1}.

Exo-isomer

LCMS retention time = 0.83 min, [M+H] = 391.36 (95%)

^{1}H NMR (400 MHz, $CDCl_3$) δ ppm 8.23 - 8.29 (m, 2 H) 6.51 - 6.55 (m, 1 H) 6.08 - 6.13 (m, 1 H) 6.03 - 6.08 (m, 1 H) 5.76 - 5.87 (m, 1 H) 3.65 (s, 12 H) 3.56 - 3.61 (m, 2 H) 3.44 - 3.49 (m, 2 H) 2.86 - 2.92 (m, 2 H) 2.34 - 2.41 (m, 1 H) 1.97 - 2.02 (m, 1 H) 1.87 - 1.93 (m, 1 H) 1.69 - 1.74 (m, 1 H) 1.29 - 1.35 (m, 1 H) 1.24 - 1.29 (m, 1 H)

^{13}C NMR (151 MHz, $CDCl_3$) δ ppm 175.5, 161.8, 157.7, 137.9, 135.8, 110.3, 70.3 (2C), 70.1, 70.0, 69.8, 69.6, 47.0, 46.1, 44.3, 41.3, 40.9, 39.0, 30.2.

HRMS (ESI+) Calculated for $C_{20}H_{31}N_4O_4^+$ $[M+H]^+$, 391.2340, found 391.2345 Da.

IR: 3294 (N-H), 1645 (C=O), 1588 (C=C), 1246 (C-N), 1098 (C-O) cm^{-1}.

Synthesis of Boc-hydrazine acid S4

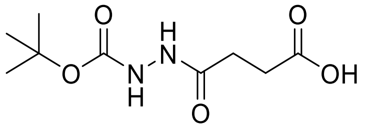

Succinic anhydride (649 µL, 9.99 mmol) and *N*-boc-hydrazine (1.14 mL, 9.99 mmol) were dissolved in water (20 mL) and the reaction mixture was stirred for 1 h, during this time the solution became colourless. The reaction mixture was lyophilised, transferred to a vial in MeOH and concentrated under a stream of nitrogen to afford 4-(2-(*tert*-butoxycarbonyl)hydrazineyl)-4-oxobutanoic acid (2.25 g, 97%) as a colourless gum.

^{1}H NMR (400 MHz, DMSO-*d6*) δ ppm 11.77 - 12.09 (m, 1 H) 9.51 (s, 1 H) 8.58 - 8.78 (m, 1 H) 2.44 (s, 2 H) 2.29 - 2.36 (m, 2 H) 1.40 (s, 9 H)

^{13}C NMR (101 MHz, DMSO-*d6*) δ ppm 174.0, 171.2, 155.7, 79.5, 29.2, 28.5 (2C).

HRMS (ESI+) Calculated for $C_{18}H_{32}N_4NaO_{10}^+$ $[2M+Na]^+$, 487.2016, found 487.2011 Da.

IR: 3274 (N-H), 2981 (O-H), 1712 (C=O), 1679 (C=O), 1152 (C-O) cm^{-1}.

Synthesis of Boc-hydrazine S5

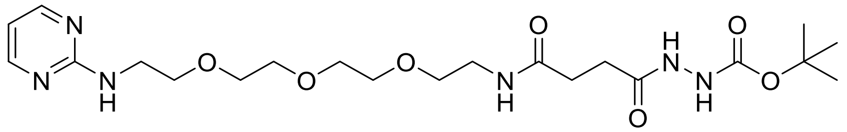

DIPEA (168 µL, 978 µmol) was added to a solution of 4-(2-(*tert*-butoxycarbonyl)hydrazineyl)-4-oxobutanoic acid (125 mg, 538 µmol) and 4-(4,6-dimethoxy-1,3,5-triazin-2-yl)-4-methylmorpholinium chloride (241 mg, 733 µmol) in DMF (2.4 mL) and the reaction mixture was stirred at rt for 5 min. *N*-(2-(2-(2-(2-aminoethoxy)ethoxy)ethoxy)ethyl)pyrimidin-2-amine, hydrochloride (150 mg, 489 µmol) was added and the reaction mixture was stirred at rt for a further 18 h. The reaction mixture was directly purified by EZprep HpH method B and a second batch by MDAP HpH method B. Appropriate fractions were combined and concentrated under reduced pressure. The crude was repurified by EZprep method B and appropriate fractions were combined and concentrated under reduced pressure to afford *tert*-butyl 4,7-dioxo-19-(pyrimidin-2-ylamino)-11,14,17-trioxa-2,3,8-triazanonadecanoate (12.3 mg, 5%) as a colourless gum.

LCMS HpH retention time = 0.71 min, [M+H] = 485.35 (91%)

^1H NMR (400 MHz, $CDCl_3$) δ ppm 8.59 - 8.72 (m, 1 H) 8.22 - 8.34 (m, 2 H) 7.20 - 7.26 (m, 1 H) 6.99 - 7.09 (m, 1 H) 6.49 - 6.57 (m, 1 H) 6.18 - 6.28 (m, 1 H) 3.60 - 3.73 (m, 12 H) 3.54 - 3.60 (m, 2 H) 3.39 - 3.46 (m, 2 H) 2.56 (s, 4 H) 1.45 (s, 9 H)

^{13}C NMR (101 MHz, $CDCl_3$) δ ppm 172.2, 161.8, 157.9 (2C), 155.6, 110.3, 81.2, 70.6, 70.4, 70.2, 70.1, 69.7, 69.6, 41.2, 39.4, 31.6, 29.9, 28.2.

HRMS (ESI+) Calculated for $C_{21}H_{37}N_6O_7^+$ $[M+H]^+$ 485.2718, found 485.2722 Da.

IR: 3269 (N-H), 1722 (C=O), 1672 (C=O), 1365 (C-N), 1157 (C-O) cm^{-1}.

Synthesis of hydrazide 8

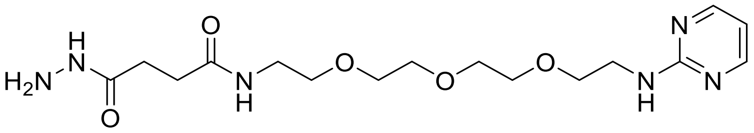

TFA (47.7 µL, 619 µmol) was added to *tert*-butyl 4,7-dioxo-19-(pyrimidin-2-ylamino)-11,14,17-trioxa-2,3,8-triazanonadecanoate (30 mg, 62 µmol) and the reaction mixture was stirred for 7 h. The reaction mixture was concentrated under a stream of nitrogen then dissolved in DMSO and purified using EZ prep HpH method A. Appropriate fractions were combined and concentrated under reduced pressure to afford 4-hydrazineyl-4-oxo-*N*-(2-(2-(2-(2-(pyrimidin-2-ylamino)ethoxy)ethoxy)ethoxy)ethyl)butanamide (7.5 mg, 33%) as a colourless gum.

LCMS HpH retention time = 0.51 min, [M+H] = 385.32 (76%)

^1H NMR (400 MHz, CHLOROFORM-*d*) δ ppm 8.18 - 8.28 (m, 2 H) 6.56 - 6.74 (m, 1 H) 6.43 - 6.54 (m, 1 H) 5.71 - 5.87 (m, 1 H) 3.83 - 3.92 (m, 1 H) 3.57 - 3.68 (m, 12 H) 3.50 - 3.57 (m, 2 H) 3.36 - 3.45 (m, 1 H) 3.09 - 3.20 (m, 1 H) 2.71 - 2.78 (m, 1 H) 2.56 - 2.68 (m, 1 H) 2.36 - 2.43 (m, 1 H) 2.19 - 2.30 (m, 1 H) 2.14 (s, 1 H) 1.84 - 1.97 (m, 1 H)

^{13}C NMR (101 MHz, $CDCl_3$) δ ppm 172.6, 171.7, 162.0, 157.67, 110.1, 70.3, 70.1, 70.0, 69.9, 69.5, 69.4, 40.8, 39.1, 31.2, 29.8.

HRMS (ESI+) Calculated for $C_{16}H_{29}N_6O_5^+$ $[M+H]^+$, 385.2194, found 385.2192 Da.

IR: 3339 (N-H), 1688 (C=O), 1683 (C=O), 1198 (N-N), 1142 (C-O) cm^{-1}.

Synthesis of ketone 9

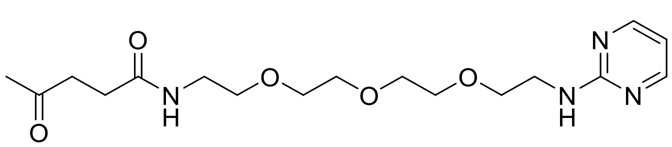

DIPEA (337 µL, 1.96 mmol) was added to a solution of levulinic acid (55 µL, 540 µmol) and HATU (279 mg, 733 µmol) in DMF (2.4 mL) and the reaction mixture was stirred at rt for 5 min. *N*-(2-(2-(2-(2-aminoethoxy)ethoxy)ethoxy)ethyl)pyrimidin-2-amine, hydrochloride (150 mg, 489 µmol) was added and the reaction mixture was stirred at rt for a further 45 min. The reaction mixture was directly purified by MDAP HpH method A and appropriate fractions were combined and concentrated under a stream of nitrogen to afford 4-oxo-*N*-(2-(2-(2-(2-(pyrimidin-2-ylamino)ethoxy)ethoxy)ethoxy)ethyl)pentanamide (93.6 mg, 52%) as a pale orange oil.

LCMS HpH in retention time = 0.62 min, [M+H]+ = 369.34 (98%)

^{1}H NMR (400 MHz, $CDCl_3$) δ ppm 8.19 - 8.31 (m, 2 H) 6.45 - 6.53 (m, 1 H) 5.75 - 5.86 (m, 1 H) 4.64 - 4.88 (m, 1 H) 3.84 - 3.92 (m, 1 H) 3.51 - 3.68 (m, 15 H) 2.62 (d, J = 17.1 Hz, 1 H) 2.20 - 2.29 (m, 1 H) 2.10 - 2.18 (m, 1 H) 1.90 (d, J = 12.7 Hz, 1 H) 1.47 - 1.51 (m, 3 H).

^{13}C NMR (101 MHz, $CDCl_3$) δ ppm 207.8, 175.6, 162.5, 158.2, 110.7, 70.7, 70.5, 70.0, 69.8, 41.34, 39.5, 39.2, 38.7, 34.8, 29.7, 26.6.

HRMS (ESI+) Calculated for $C_{17}H_{29}N_4O_5^+$ [M+H]$^+$, 369.2132, found 369.2138 Da.

IR: 3344 (N-H), 1673 (C=O), 1668 (C=O), 1227 (C-N), 1097 (C-O) cm^{-1}.

Synthesis of maleimide 10

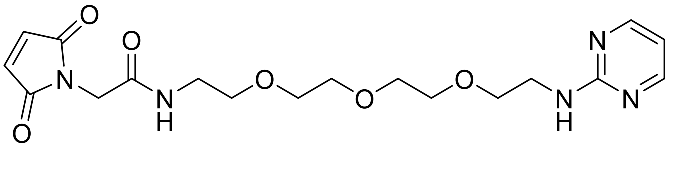

DIPEA (255 µL, 1.48 mmol) was added to a solution of 2-(2,5-dioxo-2,5-dihydro-1*H*-pyrrol-1-yl)acetic acid (40.0 µL, 407 µmol) and HATU (211 mg, 555 µmol) in DMF (1.8 mL) and the reaction mixture was stirred at rt for 5 min. *N*-(2-(2-(2-(2-aminoethoxy)ethoxy)ethoxy)ethyl)pyrimidin-2-amine (100 mg, 370 µmol) was added and the reaction mixture was stirred at rt for a further 1 h. The reaction mixture was diluted with EtOAc and water and extracted. The aqueous layer was extracted with EtOAc x 3 then the combined organic layers were washed with 5% LiCl solution. The aqueous layer was extracted with DCM x 3 then the combined organic layers were dried over a hydrophobic frit and concentrated under reduced pressure. The crude was purified by normal phase silica column chromatography with a gradient of 0-100% EtOAc in cyclohexane then 0-100% 3:1 EtOAc:EtOH in EtOAc. Appropriate fractions were combined and concentrated under reduced pressure to afford 2-(2,5-dioxo-2,5-dihydro-1*H*-pyrrol-1-yl)-N-(2-(2-(2-(2-(pyrimidin-2-ylamino)ethoxy)ethoxy)ethoxy)ethyl)acetamide (43.3 mg, 29%) as a colourless gum.

LCMS HpH retention time = 0.62 min, [M+H]+ = 408.20 (73%) (Product not stable to LCMS conditions)

^{1}H NMR (400 MHz, $CDCl_3$) δ ppm 8.22 - 8.31 (m, 2 H) 7.10 - 7.21 (m, 1 H) 6.72 - 6.77 (m, 2 H) 6.50 - 6.56 (m, 1 H) 5.82 - 5.92 (m, 1 H) 4.14 - 4.23 (m, 2 H) 3.63 - 3.73 (m, 12 H) 3.57 - 3.61 (m, 2 H) 3.44 - 3.48 (m, 2 H)

^{13}C NMR (101 MHz, $CDCl_3$) δ ppm 170.5, 166.4, 162.5, 158.3, 134.7, 110.9, 70.8 (2C), 70.6, 70.1, 69.9, 51.2, 41.5, 40.6, 39.9

HRMS (ESI+) Calculated for $C_{18}H_{26}N_5O_6^+$ [M+H]$^+$, 408.1878, found 408.1881 Da.

IR: 3207 (N-H), 1714 (C=O), 1642 (C=C), 1233 (C-O), 937 (C=C) cm^{-1}.

Synthesis of Boc-proline amide S6

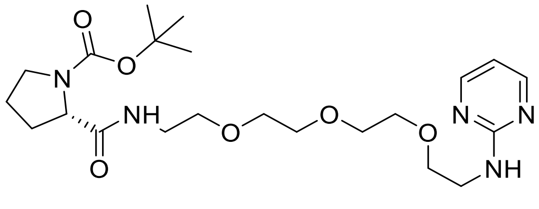

DIPEA (0.29 mL, 1.7 mmol) was added to a solution of HATU (243 mg, 640 µmol) and (2*S*)-1-[(*tert*-butoxy)carbonyl]pyrrolidine-2-carboxylic acid (101 mg, 469 µmol) in DMF (2.1 mL) and the reaction mixture was stirred for 10 min under nitrogen. 14-(Pyrimidin-2-yloxy)-3,6,9,12-tetraoxatetradecan-1-

amine, hydrochloride (150 mg, 426 μmol) was added and the reaction mixture was stirred for a further 1 h. The reaction mixture was directly purified by MDAP method B HpH and appropriate fractions were combined and concentrated under reduced pressure to afford *tert*-butyl (*S*)-2-((14-(pyrimidin-2-yloxy)-3,6,9,12-tetraoxatetradecyl)carbamoyl)pyrrolidine-1-carboxylate (76.5 mg, 35%) as a colourless gum.

LCMS HpH retention time = 0.82 min, [M+H]+ = 513.26 (99%)

^{1}H NMR (400 MHz, CHLOROFORM-*d*) δ ppm 8.41 - 8.53 (m, 2 H) 6.95 - 7.10 (m, 1 H) 6.84 - 6.93 (m, 1 H) 6.43 - 6.63 (m, 1 H) 4.44 - 4.52 (m, 2 H) 4.06 - 4.26 (m, 1 H) 3.80 - 3.87 (m, 2 H) 3.53 - 3.64 (m, 10 H) 3.47 - 3.52 (m, 2 H) 3.38 - 3.43 (m, 2 H) 1.96 - 2.24 (m, 2 H) 1.76 - 1.86 (m, 2 H) 1.40 (s, 9 H)

^{13}C NMR (101 MHz, $CDCl_3$) δ ppm 165.1, 159.2, 115.0, 80.2, 70.8, 70.6 (2C), 70.5, 70.3, 69.9, 69.3, 66.7, 47.0 (2C), 39.2, 28.5, 28.3. Reported as observed.

HRMS (ESI+) Calculated for $C_{22}H_{38}N_5O_6^+$ $[M+H]^+$, 468.2817, found 468.2828 Da.

IR: 3326 (N-H), 1673 (C=O), 1246 (C-N), 1118 (C-O) cm^{-1}.

Synthesis of proline amide S7

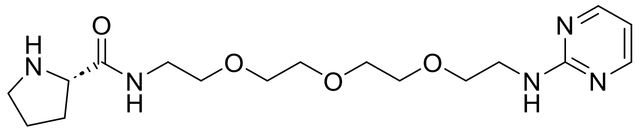

Hydrogen chloride 4 M in dioxane (504 µL, 2.02 mmol) was added to *tert*-butyl (*S*)-2-((2-(2-(2-(2-(pyrimidin-2-ylamino)ethoxy)ethoxy)ethoxy)ethyl)carbamoyl)pyrrolidine-1-carboxylate (94.3 mg, 202 µmol) and the reaction mixture was stirred at rt for 30 min. The reaction mixture was concentrated under a stream of nitrogen then dissolved in MeOH and eluted through an SCX column and washed with 4 M ammonia in methanol. The filtrate was concentrated under reduced pressure to afford (*S*)-*N*-(2-(2-(2-(2-(pyrimidin-2-ylamino)ethoxy)ethoxy)ethoxy)ethyl)pyrrolidine-2-carboxamide (78.4 mg, quantitative yield) as a white solid.

LCMS HpH broad signal retention time = 0.61 min, [M+H] = 368.35

^{1}H NMR (400 MHz, DMSO-*d*$_6$) δ ppm 8.21 - 8.29 (m, 2 H) 7.88 - 8.00 (m, 1 H) 6.92 - 7.04 (m, 2 H) 6.55 (s, 1 H) 3.48 - 3.55 (m, 11 H) 3.38 - 3.44 (m, 4 H) 3.18 - 3.24 (m, 2 H) 2.80 - 2.88 (m, 1 H) 2.71 - 2.78 (m, 1 H) 1.90 - 1.97 (m, 1 H) 1.60 - 1.69 (m, 1 H) 1.52 - 1.60 (m, 2 H)

^{13}C NMR (101 MHz, DMSO-*d*$_6$) δ ppm 171.0, , 109.5, 69.3 (2C), 69.1 (2C) 68.6, 68.4, 59.7, 46.2, 40.6, 37.7, 29.9, 25.3.

HRMS (ESI+) Calculated for $C_{17}H_{30}N_5O_4^+$ $[M+H]^+$, 368.2292, found 368.2309 Da.

IR: 3325 (N-H), 1649 (C=O), 1246 (C-N), 1098 (C-O) cm^{-1}.

Synthesis of nitrone 11

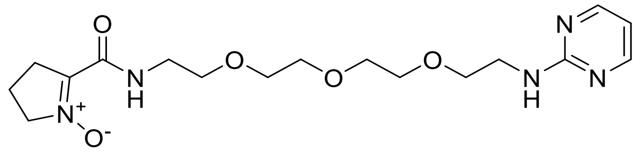

Sodium bicarbonate (89.6 mg, 1.07 mmol) was added to a stirred solution of (*S*)-*N*-(2-(2-(2-(2-(pyrimidin-2-ylamino)ethoxy)ethoxy)ethoxy)ethyl)pyrrolidine-2-carboxamide (78.4 mg, 213 µmol) and EDTA (622 µg, 2.13 µmol) in a mixture of acetonitrile (850 µL) and THF (213 µL) at 5 °C. While cooling to maintain the temperature at 5 °C, oxone (138 mg, 224 µmol) was added over 2 h, then the reaction mixture was stirred for a further 4 h. EtOAc and water were added then the layers were separated and the aqueous layer was extracted with EtOAc x 2. The combined organic layers were dried over a hydrophobic frit, concentrated under reduced pressure and purified by normal phase silica column chromatography with a gradient of 0-100% EtOAc in cyclohexane, then 0-100% 3:1 EtOAc:EtOH in EtOAc followed by a methanol flush. Appropriate fractions were combined and concentrated under reduced pressure to afford 5-((2-(2-(2-(2-(pyrimidin-2-ylamino)ethoxy)ethoxy)ethoxy)ethyl)carbamoyl)-3,4-dihydro-2*H*-pyrrole 1-oxide (7.5 mg, 9%) as a colourless gum.

LCMS HpH retention time = 0.58 min, [M+H] = 382.32 (68%)

^{1}H NMR (400 MHz, $CDCl_3$) δ ppm 10.02 - 10.23 (m, 1 H) 8.18 - 8.38 (m, 2 H) 6.44 - 6.63 (m, 1 H) 5.59 - 5.80 (m, 1 H) 4.14 - 4.28 (m, 1 H) 3.53 - 3.72 (m, 16 H) 3.02 - 3.15 (m, 1 H) 2.06 - 2.21 (m, 2 H) 0.75 - 0.98 (m, 2 H)

^{13}C NMR (151 MHz, DMSO-d_6) δ ppm 165.1, 162.2, 158.8, 138.4, 110.0, 69.8, 69.7, 69.6, 68.9, 68.8, 65.6, 48.6, 40.3, 38.0, 29.0, 15.6

HRMS (ESI+) Calculated for $C_{17}H_{28}N_5O_5^+$ $[M+H]^+$, 382.2085, found 382.2102 Da.

IR: 3370 (N-H), 1649 (C=O), 1588 (N-O), 1204 (C-N), 1096 (C-O) cm^{-1}.

Synthesis of TCO 12

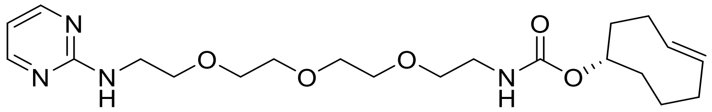

DIPEA (96.6 µL, 561 µmol) was added to a solution of (*S*,*E*)-cyclooct-4-en-1-yl (2,5-dioxopyrrolidin-1-yl) carbonate (100 mg, 374 µmol) and *N*-(2-(2-(2-(2-aminoethoxy)ethoxy)ethoxy)ethyl)pyrimidin-2-amine (101 mg, 374 µmol) in DMF (1.8 mL) and the reaction mixture was stirred at rt for 1 h in a flask wrapped with tin foil. The reaction mixture was diluted with DCM and water and extracted. The aqueous layer was extracted with DCM x 3 then the combined organic layers were washed with 5% LiCl solution, dried over a hydrophobic frit and concentrated under reduced pressure. The crude was purified by normal phase silica chromatography with a gradient of 0-100% EtOAc in cyclohexane with the UV detection at 300 nm only. All fractions were combined and concentrated under reduced pressure. The column was flushed with methanol, appropriate fractions were combined and concentrated under reduced pressure to afford (*S*,*E*)-cyclooct-4-en-1-yl (2-(2-(2-(2-(pyrimidin-2-ylamino)ethoxy)ethoxy)ethoxy)ethyl)carbamate (49.3 mg, 31%) as a colourless film.

LCMS HpH retention time = 0.99 min, [M+H]+ = 423.30 (90%)

^{1}H NMR (400 MHz, $CDCl_3$) δ ppm 8.16 - 8.29 (m, 2 H) 6.42 - 6.52 (m, 1 H) 5.57 - 5.66 (m, 1 H) 5.48 (ddd, *J* = 15.8, 6.9, 3.5 Hz, 2 H) 5.36 - 5.43 (m, 1 H) 4.26 - 4.33 (m, 1 H) 3.55 - 3.66 (m, 12 H) 3.48 - 3.54 (m, 2 H) 3.26 - 3.34 (m, 2 H) 2.27 - 2.34 (m, 2 H) 1.94 - 2.01 (m, 1 H) 1.88 (br d, *J* = 9.4 Hz, 3 H) 1.71 - 1.77 (m, 1 H) 1.67 - 1.71 (m, 1 H) 1.63 - 1.67 (m, 1 H) 1.42 - 1.54 (m, 1 H).

^{13}C NMR (101 MHz, $CDCl_3$) δ ppm 162.3, 158.0, 134.9, 133.0, 110.0 (2C), 80.4, 70.6, 70.4 (2C), 70.3, 69.9, 41.2, 41.2, 41.1, 40.8, 38.7, 34.3, 32.5, 31.0

HRMS (ESI+) Calculated for $C_{21}H_{35}N_4O_5^+$ $[M+H]^+$, 423.2602, found 423.2611 Da.

IR: 3327 (N-H), 1704 (C=O), 1631 (C=C), 1247 (C-N), 1095 (C-O) cm^{-1}.

Synthesis of tetrazine-H 13

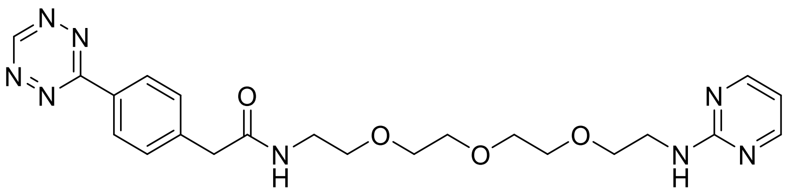

DIPEA (82.4 µL, 479 µmol) was added to a solution of 2,5-dioxopyrrolidin-1-yl 2-(4-(1,2,4,5-tetrazin-3-yl)phenyl)acetate (100 mg, 319 µmol) and *N*-(2-(2-(2-(2-aminoethoxy)ethoxy)ethoxy)ethyl)pyrimidin-2-amine (86.3 mg, 319 µmol) in DMF (1.6 mL) and the reaction mixture was stirred at rt for 2 h. The reaction mixture was directly purified by EZ prep method B HpH with an extended gradient of 45 min. Appropriate fractions were combined and concentrated under reduced pressure to afford 2-(4-(1,2,4,5-tetrazin-3-yl)phenyl)-*N*-(2-(2-(2-(2-(pyrimidin-2-ylamino)ethoxy)ethoxy)ethoxy)ethyl)acetamide (51.1 mg, 34%) as a pink solid.

LCMS HpH retention time = 0.79 min, [M+H] = 469.35 (91%)

^{1}H NMR (400 MHz, $CDCl_3$) δ ppm 10.18 - 10.24 (m, 1 H) 8.54 - 8.60 (m, 2 H) 8.25 - 8.45 (m, 2 H) 7.96 - 8.20 (m, 1 H) 7.53 - 7.64 (m, 2 H) 6.95 - 7.04 (m, 1 H) 6.64 - 6.73 (m, 1 H) 3.58 - 3.74 (m, 16 H) 3.46 - 3.52 (m, 2 H)

^{13}C NMR (101 MHz, $CDCl_3$) δ ppm 170.2, 166.4, 157.7, 141.3, 130.4, 128.5, 109.3, 77.2, 73.2, 70.6, 70.6 (2C), 70.2, 69.7, 68.9, 43.5, 41.6, 41.1, 39.6

HRMS (ESI+) Calculated for $C_{22}H_{29}N_8O_4^+$ $[M+H]^+$, 469.2306, found 469.2312 Da.

IR: 3331 (N-H), 1631 (C=O), 1224 (C-N), 1122 (N-N), 1094 (C-O) cm^{-1}.

Synthesis of tetrazine-Me 14

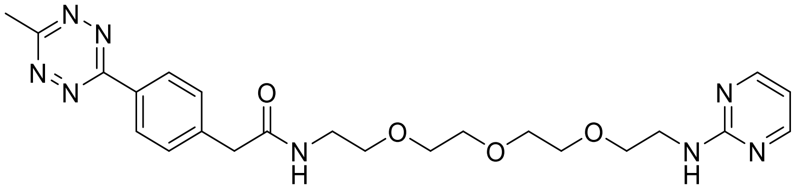

DIPEA (52.6 µL, 306 µmol) was added to a solution of *N*-(2-(2-(2-(2-aminoethoxy)ethoxy)ethoxy)ethyl)pyrimidin-2-amine (82.6 mg, 1 Eq, 306 µmol) and 2,5-dioxopyrrolidin-1-yl 2-(4-(6-methyl-1,2,4,5-tetrazin-3-yl)phenyl)acetate (100 mg, 306 µmol) in DMF (1.8 mL) and the reaction mixture was stirred at rt for 2.5 h. Further DIPEA (26.6 µL, 153 µmol) was added and the reaction was stirred for 2 h. The reaction mixture was directly purified by EZprep method B HpH. Appropriate fractions were combined and concentrated under reduced pressure to afford 2-(4-(6-methyl-1,2,4,5-tetrazin-3-yl)phenyl)-*N*-(2-(2-(2-(2-(pyrimidin-2-ylamino)ethoxy)ethoxy)ethoxy)ethyl)acetamide (81.5 mg, 55%) as a pink solid

LCMS HpH retention time = 0.83 min, [M+H] = 483.33 (98%)

^{1}H NMR (400 MHz, $CDCl_3$) δ ppm 8.48 - 8.55 (m, 2 H) 8.20 - 8.27 (m, 2 H) 7.46 - 7.53 (m, 2 H) 6.67 - 6.73 (m, 1 H) 6.48 - 6.55 (m, 1 H) 5.86 - 5.95 (m, 1 H) 3.61 (s, 14 H) 3.55 - 3.59 (m, 2 H) 3.44 - 3.49 (m, 2 H) 3.09 (s, 3 H)

^{13}C NMR (101 MHz, $CDCl_3$) δ ppm 170.1, 167.2, 163.8, 161.8, 157.8 (2C), 140.2, 130.5, 130.1, 128.2, 110.4, 70.5 (2C), 70.0, 53.4, 43.5, 41.1, 39.5, 29.7, 21.1

HRMS (ESI+) Calculated for $C_{23}H_{31}N_8O_4^+$ $[M+H]^+$, 483.2463, found 483.2464 Da.

IR: 3294 (N-H), 1648 (C=O), 1244 (C-N), 1138 (N-N), 1089 (C-O) cm^{-1}.

Synthesis of products of reactions between incompatible substrates

Reaction between azide 2 and TCEP; synthesis of 15

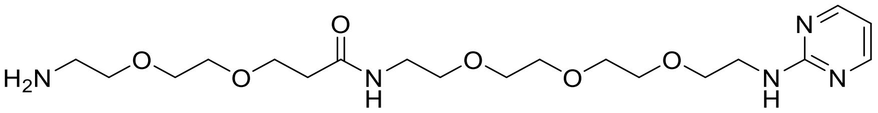

A 10 mmol solution of 3-(2-(2-azidoethoxy)ethoxy)-*N*-(2-(2-(2-(2-(pyrimidin-2-ylamino)ethoxy)ethoxy)ethoxy)ethyl)propanamide (11.4 mg, 25.0 µmol) in DMSO (2.5 mL) was produced. To the solution was added TCEP solution in PBS (2 mL, 50 mmolar, 100 µmol) and the reaction mixture was stirred at rt for 120 h. The reaction was filtered then purified by EZ prep HpH method A. Appropriate fractions were combined and concentrated under reduced pressure to afford 3-(2-(2-aminoethoxy)ethoxy)-N-(2-(2-(2-(2-(pyrimidin-2-ylamino)ethoxy)ethoxy)ethoxy)ethyl)propenamide (0.6 mg, 6%). Insufficient material was isolated for ^{13}C NMR.

LCMS HpH retention time = 0.56 min, [M+H] = 430.32 (100%)

^{1}H NMR (400 MHz, DMSO-d6) δ ppm 8.20 - 8.28 (m, 2 H) 7.79 - 7.90 (m, 1 H) 6.92 - 7.03 (m, 1 H) 6.50 - 6.58 (m, 1 H) 3.55 - 3.62 (m, 3 H) 3.44 - 3.53 (m, 13 H) 3.36 - 3.44 (m, 6 H) 3.14 - 3.20 (m, 4 H) 2.60 - 2.69 (m, 1 H) 2.25 - 2.34 (m, 3 H)

HRMS (ESI+) Calculated for $C1_9H_{36}N_5O_6^+$ $[M+H]^+$, 430.2660, found 430.2664 Da.

IR: 3294 (N-H), 3085 (N-H), 2921 (C-H), 1647 (C=O), 1360 (C-O) cm^{-1}.

Reaction between DBCO 5 and GSH; synthesis of 16

DBCO (9.0 mg, 16 µmol) was dissolved in DMSO (160 µL). To this was added glutathione (24.8 mg, 80.7 µmol) in PBS (807 µL) and the mixture was left to stand for 3 days, stirred at rt for 8 h then left to stand for 16 h. Further glutathione (24.8 mg, 80.7 µmol) in PBS (807 µL) was added and the reaction mixture was stirred for 2 h then left to stand for a further 18 h. The reaction mixture was directly purified by EZprep method A HpH. Appropriate fractions were combined and concentrated under reduced pressure to afford a mixture of compounds. LCMS data and suggested structures of the two major species are shown below, however insufficient material was isolated for NMR analysis or structural elucidation.

Product A:

LCMS HpH retention time = 0.57 min, [M+H] = 881.33 (99%)

Putative structure:

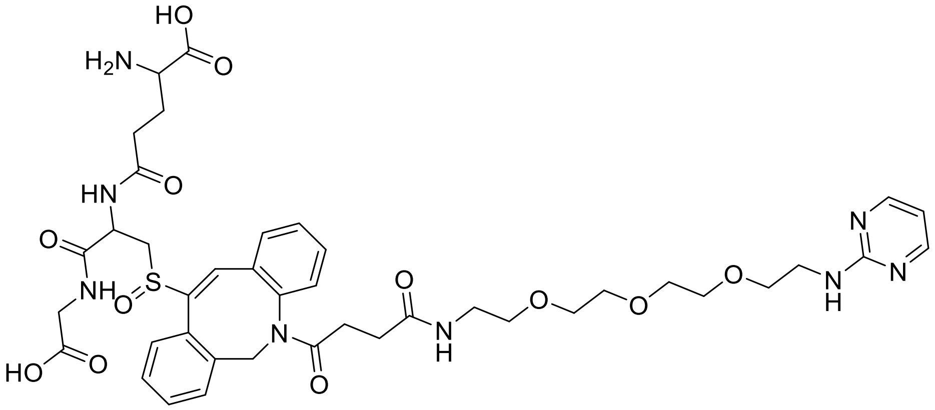

Product B:

LCMS HpH retention time = 0.60 min, [M+H] = 865.35 (100%)

Putative structure:

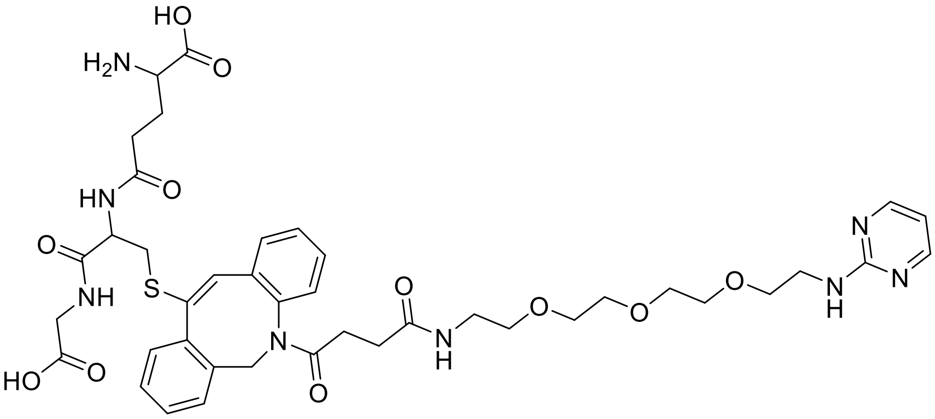

Reaction between maleimide 10 and GSH; synthesis of 17

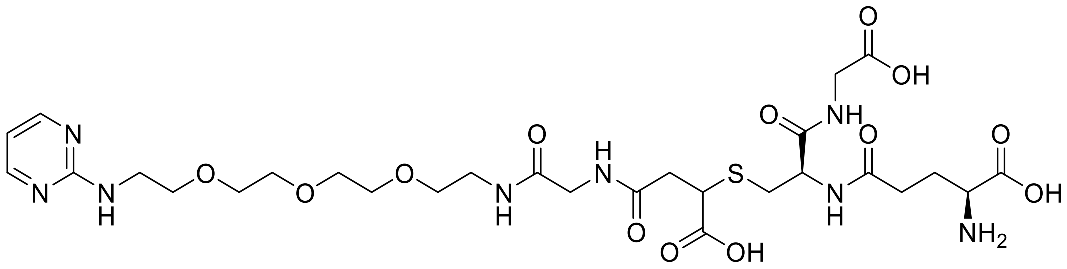

Glutathione (13.4 mg, 43.4 µmol) was added to a solution of 2-(2,5-dioxo-2,5-dihydro-1*H*-pyrrol-1-yl)-*N*-(2-(2-(2-(2-(pyrimidin-2-ylamino)ethoxy)ethoxy)ethoxy)ethyl)acetamide (17.7 mg, 43.4 µmol) in DMSO (220 µL) and PBS (220 µL) for 5 days. The reaction was diluted with 4:1 DCM:MeOH and 5% aq LiCl solution and extracted. The aqueous layer was extracted with 4:1 DCM:MeOH x 3 then the combined organic layers were discarded and the combined aqueous layers were concentrated under reduced pressure. The crude was purified by EZprep HpH method A modified to start at 0% HpH water, first UV absorbance changed to 230 nm and Appropriate fractions were combined and concentrated under reduced pressure to afford *N*5-((2*R*)-3-((1-carboxy-3-oxo-3-((2-oxo-14-(pyrimidin-2-ylamino)-6,9,12-trioxa-3-azatetradecyl)amino)propyl)thio)-1-((carboxymethyl)amino)-1-oxopropan-2-yl)-L-glutamine as a colourless gum (12.4 mg, 9%). Insufficient quantity to acquire ^{13}C spectrum.

LCMS HpH retention time = 0.39 min, [M+H] = 733.23 (90%)

^1H NMR (400 MHz, DMSO-d_6) δ ppm 9.66 - 9.79 (m, 1 H) 8.74 - 8.90 (m, 1 H) 8.29 - 8.37 (m, 1 H) 8.21 - 8.28 (m, 2 H) 7.88 - 7.99 (m, 1 H) 7.70 - 7.81 (m, 1 H) 6.96 - 7.11 (m, 2 H) 6.49 - 6.61 (m, 1 H) 4.23 - 4.40 (m, 1 H) 3.62 - 3.72 (m, 2 H) 3.46 - 3.57 (m, 12 H) 3.38 - 3.45 (m, 5 H) 3.25 - 3.34 (m, 1 H) 2.76 - 2.86 (m, 1 H) 2.56 - 2.68 (m, 1 H) 2.53 - 2.55 (m, 3 H) 2.21 - 2.45 (m, 3 H) 1.85 - 2.13 (m, 2 H). Three exchangeable protons not observed

HRMS (ESI+) Calculated for $C_{28}H_{45}N_8O_{13}S^+$ $[M+H]^+$, 733.2821, found 733.2799 Da.

IR: 3365 (O-H), 3279 (N-H), 1651 (C=O), 1231 (C-O) cm^{-1}.

Reaction between maleimide 10 and pH 10; synthesis of 18

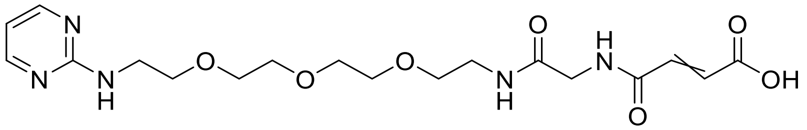

Carbonate buffer pH 10 (320 µL) was added to a solution of 2-(2,5-dioxo-2,5-dihydro-1*H*-pyrrol-1-yl)-*N*-(2-(2-(2-(2-(pyrimidin-2-ylamino)ethoxy)ethoxy)ethoxy)ethyl)acetamide (28.9 mg, 70.9 µmol) in DMSO (32 µL) and the reaction mixture was stirred for 6 hours then left to stand for 4 days. Further carbonate buffer pH 10 (320 µL) was added and the reaction mixture was left to stand for 6 days. The reaction mixture was directly purified by EZ prep HpH method A and appropriate fractions were combined and concentrated under a stream of nitrogen to afford 13,16-dioxo-1-(pyrimidin-2-ylamino)-3,6,9-trioxa-12,15-diazanonadec-17-en-19-oic acid as a white solid (4 mg, 6.4%).

LCMS HpH retention time = 0.45 min, [M+H] = 426.25 (100%)

^1H NMR (400 MHz, DMSO-d_6) δ ppm 9.26 - 9.42 (m, 1 H) 8.21 - 8.32 (m, 2 H) 7.97 - 8.11 (m, 1 H) 6.92 - 7.05 (m, 1 H) 6.52 - 6.62 (m, 1 H) 6.39 - 6.48 (m, 1 H) 6.17 - 6.31 (m, 1 H) 3.78 - 3.83 (m, 2 H) 3.51 - 3.55 (m, 6 H) 3.43 (s, 4 H) 3.27 - 3.37 (m, 4 H) 3.21 - 3.26 (m, 2 H). One exchangeable proton not observed.

^{13}C NMR (101 MHz, DMSO-d_6) δ ppm 168.0, 166.0, 165.5, 162.2, 157.9, 132.0, 131.7, 110.0, 69.6, 69.3, 42.3. Overlapping PEG signals observed

HRMS (ESI+) Calculated for $C_{18}H_{28}N_5O_7^+$ $[M+H]^+$, 426.1983, found 426.1993 Da.

IR: 3276 (N-H), 1660, (C=O), 1633 (C=C), 1243 (C-O) cm^{-1}.

Reaction between maleimide 10 and TCEP; synthesis of 19

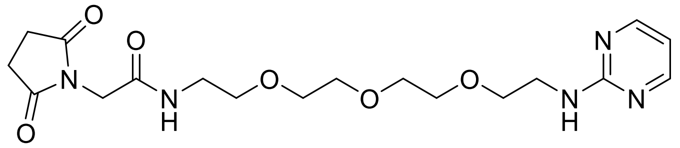

2-(2,5-Dioxo-2,5-dihydro-1H-pyrrol-1-yl)-*N*-(2-(2-(2-(2-(pyrimidin-2-ylamino)ethoxy)ethoxy)ethoxy)ethyl)acetamide (26.4 mg, 64.8 µmol) was dissolved in DMSO (200 µL) before addition of TCEP (92.9 mg, 324 µmol) and PBS (200 µL). The reaction mixture was stirred at rt for 1 h, left to stand overnight then concentrated under a stream of nitrogen. The reaction was purified by EZ prep method A UV absorbance changed to 239 nm and appropriate fractions were combined and concentrated under reduced pressure to afford 2-(2,5-dioxopyrrolidin-1-yl)-N-(2-(2-(2-(2-(pyrimidin-2-ylamino)ethoxy)ethoxy)ethoxy)ethyl)acetamide (2.6 mg, 10%) as a white solid.

LCMS retention time = 0.57 min, [M+H] = 410.24 (70%)

^{1}H NMR (400 MHz, $CDCl_3$) δ ppm 8.19 - 8.32 (m, 2 H) 7.18 - 7.25 (m, 1 H) 6.48 - 6.58 (m, 1 H) 5.74 - 5.90 (m, 1 H) 4.12 - 4.23 (m, 2 H) 3.67 (br dd, *J* = 5.8, 1.1 Hz, 14 H) 3.42 - 3.51 (m, 2 H) 2.70 - 2.84 (m, 4 H)

^{13}C NMR (101 MHz, $CDCl_3$) δ ppm 176.7, 165.4, 162.2, 158.0, 110.6, 70.5, 70.0, 41.2, 41.1, 39.6, 28.3. 4C not observed.

HRMS (ESI+) Calculated for $C_{18}H_{28}N_5O_6^+$ $[M+H]^+$, 410.2034, found 410.2039 Da.

IR: 2924 (N-H), 1708 (C=O), 1670 C=O), 1200 (C-N), 1172 (C-O) cm^{-1}.

Reaction between maleimide 10 and urea; synthesis of 20

Putative structure:

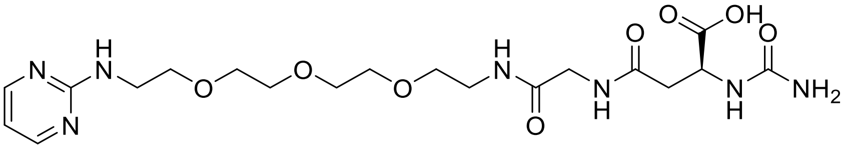

Urea (19.7 mg, 328 µmol) was added to a solution of 2-(2,5-dioxo-2,5-dihydro-1*H*-pyrrol-1-yl)-*N*-(2-(2-(2-(2-(pyrimidin-2-ylamino)ethoxy)ethoxy)ethoxy)ethyl)acetamide (27.8 mg, 68.2 µmol) in DMSO (220 µL) and acetate buffer pH 2.8 (220 µL) for 6 days. The reaction mixture was directly purified by EZ prep method A HpH, first UV detection changed to 229 nm. Appropriate fractions were combined and concentrated under a stream of nitrogen to afford a colourless film (2.5 mg, 8%). Insufficient quantity to acquire a ^{13}C spectrum.

LCMS HpH retention time = 0.44 min, [M+H] = 486.19 (91%)

^{1}H NMR (400 MHz, DMSO-d_6) δ ppm 8.50 - 8.62 (m, 1 H) 8.29 - 8.35 (m, 1 H) 8.26 (d, *J* = 4.7 Hz, 2 H) 6.99 - 7.07 (m, 1 H) 6.53 - 6.59 (m, 1 H) 6.07 - 6.15 (m, 1 H) 5.48 - 5.63 (m, 2 H) 3.98 - 4.08 (m, 1 H) 3.62 - 3.73 (m, 2 H) 3.45 - 3.56 (m, 12 H) 3.40 - 3.44 (m, 4 H) 3.15 - 3.21 (m, 2 H). One exchangeable proton not observed

HRMS (ESI+) Calculated for $C_{19}H_{32}N_7O_8^+$ $[M+H]^+$, 486.2307, found 486.2311 Da.

IR: 3349 (N-H), 1655 (C=O), 1364 (C-N), 1280 (C-O) cm^{-1}.

Reaction between maleimide 10 and DTT; synthesis of S8

DTT (24 mg, 160 µmol) was added to a solution of 2-(2,5-dioxo-2,5-dihydro-1*H*-pyrrol-1-yl)-*N*-(2-(2-(2-(2-(pyrimidin-2-ylamino)ethoxy)ethoxy)ethoxy)ethyl)acetamide (24.0 mg, 31.3 µmol) in DMSO (400 µL) and the reaction mixture was stirred at rt for 6 days before further addition of DTT (24 mg, 160 µmol) and the reaction mixture was stirred for 24 h. Further DTT (24 mg, 160 µmol) and PBS (200 µL) were added and the reaction mixture was stirred for a further 4 days. The reaction mixture was diluted with 4:1 DCM:MeOH and water then extracted. The aqueous layer was extracted with 4:1 DCM/MeOH x 2 then the aqueous and organic layers were combined and concentrated under a stream of nitrogen. The crude was dissolved in the minimum amount of DMSO and purified by EZprep method A first absorbance modified to 222 nm. Appropriate fractions were combined and concentrated under a stream of nitrogen to afford isolated DTT. Reaction mixture LCMS spectra suggested conversion to hydrolysed maleimide, suggesting the DTT addition adduct is not stable and hydrolyses.

Reaction between Tetrazine-H 21 and TCEP/ sodium ascorbate; synthesis of 22

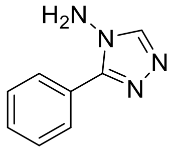

- 3-Phenyl-1,2,4,5-tetrazine (50.0 mg, 316 μmol) was dissolved in DMSO (263 μL) and PBS (1.3 mL) and sodium ascorbate (523 mg, 2.64 mmol) was added. The reaction mixture was stirred at rt for 1.5 h, during this time the colour changed from pink to yellow. The reaction mixture was filtered then purified by MDAP formic method A. Appropriate fractions were combined and concentrated under a stream of nitrogen to afford 3-phenyl-4*H*-1,2,4-triazol-4-amine (7.9 mg, 16%) as a pale orange solid.

- A solution of 3-phenyl-1,2,4,5-tetrazine (20.2 mg, 128 μmol) in DMSO (2.6 mL) was produced. To the solution was added TCEP (305 mg, 1.06 mmol) in PBS (5.3 mL), the reaction was stirred for 4 days, during which the solution changed colour from pink to yellow. The reaction mixture was directly purified by EZ prep method A HpH. Appropriate fractions were combined and concentrated under reduced pressure to afford 3-phenyl-4*H*-1,2,4-triazol-4-amine (7 mg, 34%).

LCMS retention time = 0.49 min, [M+H] = 161.05 (100%)

^{1}H NMR (400 MHz, DMSO-d$_6$) δ ppm 8.45 - 8.49 (m, 1 H) 7.98 - 8.06 (m, 2 H) 7.45 - 7.55 (m, 3 H) 6.27 - 6.35 (m, 2 H)

^{13}C NMR (101 MHz, DMSO-d$_6$) δ ppm 152.3, 146.9, 129.9, 128.9, 128.4, 127.4

^{15}N^{1}H HMBC NMR key correlations: 8.48/ 313.3, 8.48/180.3, 6.39/67.6, 6.33/180.3, 6.27/67.6

HRMS (ESI+) Calculated for $C_{16}H_{16}N_8Na^+$ [2M+Na]$^+$, 343.1390, found 343.1394 Da.

IR: 3373 (N-H), 3123 (N-H), 1475 (C-H), 1186 (N-N), 1007 (C-N) cm^{-1}.

Identification of component of CuAAC cocktail which causes Tetrazine-H 21 reaction

- 3-Phenyl-1,2,4,5-tetrazine (100 mg, 632 μmol) was dissolved in DMSO (527 μL) and PBS (2.6 mL). To the reaction was added copper(II) sulfate anhydrous (169 mg, 1.06 mmol) and an LCMS was taken: LCMS formic retention time = 0.86 min, [M+H] = 159.08 (93%) suggested no conversion from starting material. THPTA (43 mg, 99 μmol) was added, in which the colour changed from bright pink to a deeper pink, then an LCMS was taken: LCMS formic retention time = 0.86 min, [M+H] = 158.95 (97%) suggested no conversion from starting material. Sodium ascorbate (1.0 g, 5.3 mmol) was added and the reaction mixture was stirred for 1 min. The reaction mixture changed from pink to brown.

  LCMS retention time = 0.52 min, [M+H] = 161.11 (50%), suggested sodium ascorbate was the component of the CuAAC cocktail which resulted in Tetrazine-H instability.

- 3-Phenyl-1,2,4,5-tetrazine (50 mg, 316 μmol) was dissolved in DMSO (263 μL) and PBS (1.3 mL). Sodium ascorbate (523 mg, 2.64 mmol) was added and the reaction mixture was stirred for 1.5 h. The reaction mixture changed from pink to yellow.

  LCMS retention time = 0.52 min, [M+H] = 161.12 (90%), confirmed sodium ascorbate was the component of the CuAAC cocktail which resulted in Tetrazine-H instability.

Reaction between Tetrazine-H 22 and DTT; synthesis of 23

Putative structure:

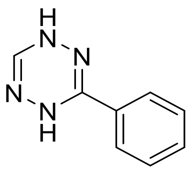

DTT (416 mg, 2.70 mmol) was added to 3-phenyl-1,2,4,5-tetrazine (88.1 mg, 557 μmol) in DMSO (1.4 mL) and PBS (1.4 mL) and the reaction mixture was stirred for 5 days. The reaction was directly purified by EZprep method A with the first UV absorbance changed to 235 nm. Appropriate fractions were combined and concentrated under a stream of nitrogen to afford an orange solid. Insufficient purity for structural elucidation.

LCMS retention time = 0.59 min, [M+H] = 161.03

^{1}H NMR (400 MHz, DMSO-d_6) δ ppm 8.64 - 8.78 (m, 1 H) 8.31 - 8.44 (m, 1 H) 7.69 - 7.82 (m, 2 H) 7.42 (m, 3 H) 6.77 - 6.87 (m, 1 H)

^{13}C NMR (101 MHz, DMSO-d_6) δ ppm 146.6, 140.1, 130.3, 129.7, 128.2, 125.7.

IR: 3219 (N-H), 2964 (C-H), 2121 (C=N), 1133 (N-N) cm^{-1}.

Reaction between Tetrazine-Me 14 and TCEP/ sodium ascorbate; synthesis of 24

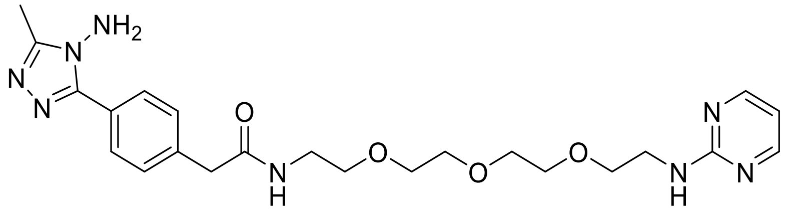

- A solution of 2-(4-(6-methyl-1,2,4,5-tetrazin-3-yl)phenyl)-*N*-(2-(2-(2-(2-(pyrimidin-2-ylamino)ethoxy)ethoxy)ethoxy)ethyl)acetamide (10.9 mg, 22.6 μmol) in DMSO (451 μL) was produced. To the solution was added TCEP (53.9 mg, 188μmol) in PBS (941 μL), the reaction was stirred for 4 days, during this time the reaction mixture changed colour from pink to yellow. The reaction was directly purified by EZ prep method A HpH and appropriate fractions were combined and concentrated under reduced pressure to afford 2-(4-(4-amino-5-methyl-4*H*-1,2,4-triazol-3-yl)phenyl)-N-(2-(2-(2-(2-(pyrimidin-2-ylamino)ethoxy)ethoxy)ethoxy)ethyl)acetamide (6.6 mg, 60%). Insufficient quantity to acquire a ^{13}C NMR spectrum.

LCMS HpH retention time = 0.58 min, [M+H] = 485.22 (98%)

^{1}H NMR (400 MHz, DMSO-d_6) δ ppm 8.17 - 8.30 (m, 2 H) 8.07 - 8.16 (m, 1 H) 7.86 - 8.00 (m, 2 H) 7.31 - 7.41 (m, 2 H) 6.90 - 7.04 (m, 1 H) 6.52 - 6.55 (m, 1 H) 5.92 - 6.02 (m, 2 H) 3.48 - 3.53 (m, 7 H) 3.45 - 3.47 (m, 1 H) 3.38 - 3.44 (m, 3 H) 3.33 - 3.35 (m, 1 H) 3.27 (s, 7 H) 3.18 - 3.23 (m, 2 H)

^{13}C from HMBC: 170.6, 163.0, 153.8, 153.0, 138.4, 129.7, 128.3, 126.4, 110.8, 70.8, 70.0, 69.6, 42.8, 41.1, 40.4, 39.5. Reported as observed.

^{15}N^{1}H HMBC NMR key correlations: 8.24/114.8, 8.09/114.8, 7.09/85.5, 6.94/86.4, 6.05/64.6, 5.93/64.6

HRMS (ESI+) Calculated for $C_{23}H_{33}N_8O_4^+$ $[M+H]^+$, 485.2619, found 485.2623 Da.

IR: 1652 (C=O), 1362 (C-N), 1315, (C-O), 1096 (N-N) cm^{-1}.

- A solution of 2-(4-(6-methyl-1,2,4,5-tetrazin-3-yl)phenyl)-N-(2-(2-(2-(2-(pyrimidin-2-ylamino)ethoxy)ethoxy)ethoxy)ethyl)acetamide (18.7 mg, 38.8 μmol) in DMSO (3.9 mL) was produced and an LCMS was taken. To the solution was added copper(II) sulfate anhydrous (6.2 mg, 39 μmol) in PBS (775 μL), the reaction was stirred for 1.5 h. To the reaction was added sodium ascorbate (61.4 mg, 310 μmol) in PBS (6.2 mL) and the reaction mixture was stirred for 4 h, left to stand for 17 h then stirred for 3 h. The reaction mixture was filtered and purified by EZ prep formic method A, however only starting material could be isolated.

  Reaction mixture LCMS suggested formation of 2-(4-(4-amino-5-methyl-4*H*-1,2,4-triazol-3-yl)phenyl)-N-(2-(2-(2-(2-(pyrimidin-2-ylamino)ethoxy)ethoxy)ethoxy)ethyl)acetamide:
  LCMS formic retention time = 0.49 min, [M+H] = 485.28 (66%)

Reaction between cyclopropene 4 and copper sulfate and sodium ascorbate; synthesis of S9

Sodium ascorbate (195 mg, 984 μmol) was added to a mixture of *N*1-((2-methylcycloprop-2-en-1-yl)methyl)-N4-(2-(2-(2-(2-(pyrimidin-2-ylamino)ethoxy)ethoxy)ethoxy)ethyl)succinamide (54 mg, 120 μmol) and copper(II)sulfate anhydrous (27.8 mg, 174 μmol) in DMSO (311 μL) and PBS (311 μL) and the reaction mixture was stirred at rt for 21 h. The temperature was increased to 37 degC then the reaction was stirred for a further 5 days. To the reaction mixture was added copper(II)sulfate anhydrous (27.8 mg, 174 μmol) and sodium ascorbate (195 mg, 984 μmol) and PBS (311 μL) and DMSO (311 μL) and the reaction mixture was stirred at 37 degC for 24 h, the reaction mixture was left to stand for 5 days, then was heated to 55 °C and stirred for 2 days. No conversion from starting material was observed by LCMS, only increases in levels of impurity.

Reaction between hydrazide 8 and pH 10; synthesis of S10

Putative structure:

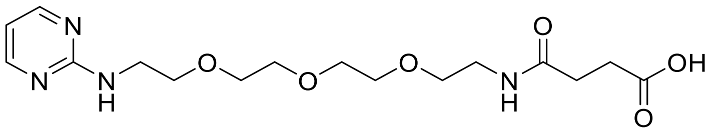

Carbonate buffer pH 10 (220 μL) was added to 4-hydrazineyl-4-oxo-*N*-(2-(2-(2-(2-(pyrimidin-2-ylamino)ethoxy)ethoxy)ethoxy)ethyl)butanamide (20.3 mg, 52.8 μmol) in DMSO (44 μL) and the reaction mixture was stirred for 6 days. The reaction mixture was directly purified by EZ prep method A UV detection changed to 240 and 305 nm. Appropriate fractions were combined and concentrated under a stream of nitrogen to produce a colourless film (3.6 mg).

LCMS suggested a mixture of two species, one of which was remaining starting material. Retention time = 0.45 min, [M+H] = 371.17 (33%)

^{1}H NMR (400 MHz, DMSO-d_6) δ ppm 8.20 - 8.38 (m, 2 H) 7.89 - 8.07 (m, 1 H) 6.90 - 7.06 (m, 1 H) 6.47 - 6.63 (m, 1 H) 3.46 - 3.57 (m, 13 H) 3.38 - 3.45 (m, 3 H) 3.15 - 3.21 (m, 1 H) 2.60 - 2.65 (m, 3 H) 2.24 - 2.40 (m, 1 H)

^{13}C NMR (101 MHz, DMSO-d_6) δ ppm 177.3 (2C), 162.0, 157.6, 109.8, 69.5, 69.4, 69.3, 69.1, 68.6, 66.0, 40.1, 37.1, 27.7, 27.6.

HRMS (ESI+) Calculated for $C_{16}H_{27}N_4O_6^+$ $[M+H]^+$, 371.1925, found 371.1941 Da.

IR: 3369 (O-H), 1695 (C=O), 1189 (C-O), 1096 (C-N) cm^{-1}.

Reaction between hydrazide 8 and dehydroascorbic acid; synthesis of S11

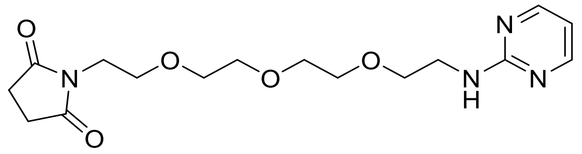

4-Hydrazineyl-4-oxo-N-(2-(2-(2-(2-(pyrimidin-2-ylamino)ethoxy)ethoxy)ethoxy)ethyl)butanamide (16.4 mg, 1 Eq, 42.7 μmol) was added to a solution of dehydroascorbic acid (51 mg, 6.9 Eq, 292.92 μmol) in DMSO (200 μL) and PBS (200 μL) and the reaction mixture was stirred at rt for 6 days. The reaction mixture was diluted with DMSO and purified by EZ prep HpH method A first absorbance changed to 235 nm. Appropriate fractions were combined and concentrated under a stream of nitrogen to afford 1-(2-(2-(2-(2-(pyrimidin-2-ylamino)ethoxy)ethoxy)ethoxy)ethyl)pyrrolidine-2,5-dione as a brown gum (3.5 mg, 23%).

LCMS retention time = 0.61 min, [M+H] = 353.16

^{1}H NMR (600 MHz, DMSO-d_6) δ ppm 8.19 - 8.30 (m, 2 H) 6.95 - 7.07 (m, 1 H) 6.50 - 6.60 (m, 1 H) 3.50 (br d, *J* = 6.8 Hz, 14 H) 3.38 - 3.43 (m, 2 H) 2.60 - 2.64 (m, 4 H)

^{13}C NMR (101 MHz, DMSO-d_6) δ ppm 177.5, 162.2, 157.8, 110.0, 69.7, 69.5 (2C), 69.3, 68.8, 66.2, 40.3, 37.3, 27.9.

HRMS (ESI+) Calculated for $C_{16}H_{25}N_4O_5^+$ $[M+H]^+$, 353.1819, found 353.1842 Da.

IR: 3348 (N-H), 1590 (C=O), 1232 (C-O), 1097 (C-N) cm^{-1}.

Reaction between hydrazide 8 and copper sulfate and sodium ascorbate; synthesis of S12

Sodium ascorbate (64 mg, 320 μmol) was added to a solution of 4-hydrazineyl-4-oxo-*N*-(2-(2-(2-(2-(pyrimidin-2-ylamino)ethoxy)ethoxy)ethoxy)ethyl)butanamide (20.7 mg, 53.8 μmol) and copper(II)sulfate anhydrous (17 mg, 110 μmol) in DMSO (100 μL) and PBS (100 μL) and the reaction mixture was stirred at rt for 6 days. The reaction mixture was directly purified by EZ prep HpH method A with the first UV detection changed to 235 nm. Appropriate fractions were combined to afford an impure mixture. The crude was purified using normal phase silica chromatography with a gradient of 0-100% MeOH in DCM. Appropriate fractions were combined and concentrated under reduced pressure to afford a yellow/ brown solid of insufficient purity for structural elucidation following two purifications.

Reaction between nitrone 11 and TCEP; synthesis of S13

TCEP (52.4 mg, 183 μmol) was added to a solution of 5-((2-(2-(2-(2-(pyrimidin-2-ylamino)ethoxy)ethoxy)ethoxy)ethyl)carbamoyl)-3,4-dihydro-2*H*-pyrrole 1-oxide (12 mg, 31 μmol) in DMSO (200 μL) and PBS (200 μL) and the reaction mixture was stirred at rt for 2 days. The reaction was directly purified by EZprep method A, gradient modified from 15-40% MeCN in HpH water, first UV detection changed to 238 nm. Appropriate fractions were combined and concentrated under a stream of nitrogen to afford two batches of insufficient yield for structural elucidation.

LCMS HpH retention time = 0.57 min.

^{1}H NMR (400 MHz, DMSO-d_6) δ ppm 8.64 - 8.76 (m, 1 H) 8.22 - 8.30 (m, 2 H) 6.94 - 7.07 (m, 1 H) 6.54 - 6.58 (m, 1 H) 6.50 - 6.53 (m, 1 H) 3.49 - 3.55 (m, 8 H) 3.39 - 3.45 (m, 4 H) 3.26 - 3.29 (m, 6 H) 2.66 - 2.70 (m, 1 H) 2.31 - 2.35 (m, 1 H) 1.89 - 1.97 (m, 3 H) 1.47 - 1.51 (m, 2 H) 1.23 - 1.27 (m, 1 H) 1.13 - 1.17 (m, 1 H).

Reaction between nitrone 11 and copper sulfate and sodium ascorbate; synthesis of S14

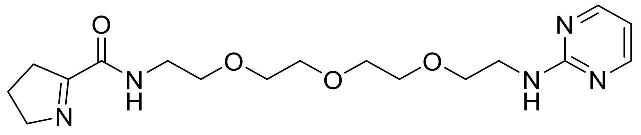

Sodium ascorbate (56.2 mg, 284 μmol) was added to a solution of 5-((2-(2-(2-(2-(pyrimidin-2-ylamino)ethoxy)ethoxy)ethoxy)ethyl)carbamoyl)-3,4-dihydro-2*H*-pyrrole 1-oxide (14 mg, 38 μmol) and copper(II) sulfate anhydrous (10 mg, 62 μmol) in DMSO (200 μL) and PBS (200 μL) and the reaction mixture was stirred at rt for 14 days, then was heated to 40 °C and stirred for 3 days. The reaction mixture was diluted with DMSO, filtered and purified by EZprep method A first UV detection 232 nm. Appropriate fractions were combined and concentrated under a stream of nitrogen to afford *N*-(2-(2-(2-(2-(pyrimidin-2-ylamino)ethoxy)ethoxy)ethoxy)ethyl)-3,4-dihydro-2*H*-pyrrole-5-carboxamide as a white solid (4 mg, 29%). Insufficient quantity to acquire a ^{13}C NMR spectrum.

LCMS HpH retention time = 0.66 min, [M+H] = 366.18 (98%)

^{1}H NMR (400 MHz, DMSO-d_6) δ ppm 8.22 - 8.31 (m, 2 H) 8.10 - 8.20 (m, 1 H) 6.94 - 7.04 (m, 1 H) 6.49 - 6.62 (m, 1 H) 3.87 - 3.99 (m, 2 H) 3.50 - 3.55 (m, 10 H) 3.45 - 3.49 (m, 2 H) 3.39 - 3.45 (m, 2 H) 3.32 (br s, 2 H) 2.60 - 2.73 (m, 2 H) 1.76 - 1.97 (m, 2 H)

HRMS (ESI+) Calculated for $C_{17}H_{28}N_5O_4^+$ $[M+H]^+$, 366.2136, found 366.2142 Da.

IR: 3288 (N-H), 1668 (C=O), 1623 (C=N), 1098 (C-N) cm^{-1}.

Reaction between Tetrazine-H 23 and pH 10; synthesis of S15

3-Phenyl-1,2,4,5-tetrazine (29.7 mg, 188 μmol) was dissolved in DMSO (94 μL) before addition of carbonate buffer pH 10 (846 μL). The reaction was left to stand over the weekend. The pH was adjusted to 10 using 2 M NaOH solution, after which the mixture immediately changed colour to a dark orange. DMSO (500 μL) was added and the reaction mixture was stirred at rt for 8 h and left to stand for 16 h. The reaction was directly purified by EZ prep HpH method A modified gradient up to 85% MeCN over 1 h and appropriate fractions were combined in batches.

Product A: 14.3 mg

LCMS retention time = 1.32 min (393.28, 72%) and 1.67 min (785.45, 28%)

^{1}H NMR (600 MHz, DMSO-d_6) δ ppm 8.12 - 8.23 (m, 1 H) 7.64 - 7.78 (m, 1 H) 4.00 - 4.24 (m, 1 H) 3.01 - 3.11 (m, 1 H) 2.67 - 2.79 (m, 1 H) 2.28 (s, 2 H) 2.12 - 2.20 (m, 2 H) 1.27 - 1.35 (m, 1 H) 1.06 - 1.13 (m, 1 H)

^{13}C NMR (151 MHz, DMSO-d_6) δ ppm 170.9, 136.5, 111.4, 45.8, 40.2, 31.3, 19.6, 13.1.

Putative structure contains amide O=C-NH-CH_2-CH_2-R.

Product B: 0.2 mg

LCMS retention time = 0.73 min, [M+H] = 307.09 (100%)

HRMS (ESI+) found 307.1311 Da, consistent with $C_{16}H_{15}N_6O$

Product C: 1 mg

LCMS retention time = 0.50 min, poor ionisation (94%)

Product D: 1.3 mg

LCMS retention time = 0.84 min, [M+H] = 225.14 (100%)

HRMS (ESI+) found 225.1033 Da, consistent with $C_{14}H_{13}N_2O$

Product E: 1.2 mg

LCMS retention time = 1.21 min, [M+H] = 209.14 (68%)Product F: 1.3 mg

LCMS retention time = 0.93 min, [M+H] = 267.13 (98%)

HRMS (ESI+) found 267.1255 Da, consistent with $C_{15}H_{15}N_4O$

Product G: 4.1 mg

LCMS retention time = 1.32 min, [M+H] = 393.27 (72%) and 1.67 min, [M+H] = 785.43 (19%).

Reaction between Tetrazine-H 15 and dehydroascorbic acid; synthesis of S16

Dehydroascorbic acid (325 mg, 1.87 mmol) was added to 3-phenyl-1,2,4,5-tetrazine (59 mg, 370 μmol) in DMSO (933 μL) and PBS (933 μL) and the reaction mixture was stirred for 5 days. The reaction mixture was directly purified by EZ prep method A first UV detection set to 245 nm and appropriate fractions were combined and concentrated under a stream of nitrogen as a yellow/ brown gum (8 mg).

LCMS HpH retention time = 0.39 min, [M+H] = 189.07 or 264.10 (100%)

NMR contained too high levels of impurity for structural confirmation

Reaction between Tetrazine-Me 14 and pH 10; synthesis of S17

Carbonate buffer pH 10 (175 μL) was added to 2-(4-(6-methyl-1,2,4,5-tetrazin-3-yl)phenyl)-*N*-(2-(2-(2-(2-(pyrimidin-2-ylamino)ethoxy)ethoxy)ethoxy)ethyl)acetamide (11 mg, 23 μmol) in DMSO (35 μL) and the reaction mixture was stirred for 5 days. The reaction mixture was directly purified by EZ prep method B first UV detection changed to 230 and appropriate fractions were combined and concentrated under reduced pressure to afford a colourless film (0.2 mg).

LCMS retention time = 0.67 min, [M+H] = 471.22 (99%).

Insufficient material isolated for NMR analysis and structural elucidation.

Reaction between DBCO 5 and TCEP; synthesis of S18

TCEP (40 mg, 140 μmol) was added to a solution of DBCO (1.6 mL, 10 mmolar, 16 μmol) in PBS (50 μL) and the reaction mixture was stirred for 2 days. Further TCEP (40 mg, 140 μmol) was added and the reaction mixture was stirred for a further 5 days. Further TCEP (40 mg, 140 μmol) was added and the reaction mixture was stirred for a further 1 day. The reaction mixture was directly purified by EZ prep method A first UV detection changed to 231 nm. Appropriate fractions were combined and concentrated under a stream of nitrogen to afford a colourless film. Insufficient sample isolated for structural elucidation.

LCMS HpH retention time = 0.49 min, [M+H] = 808.28 (100%). Mass suggests addition of TCEP into alkyne

Reaction between BCN 3 and GSH; synthesis of S19

Glutathione (298 mg, 970 μmol) was added to ((1*R*,8*S*,9s)-bicyclo[6.1.0]non-4-yn-9-yl)methyl (2-(2-(2-(2-(pyrimidin-2-ylamino)ethoxy)ethoxy)ethoxy)ethyl)carbamate (80.9 mg, 181 μmol) in DMSO (453 μL) and PBS (453 μL) and the reaction mixture was stirred for 5 days. The reaction was diluted with 4:1 DCM/MeOH and 5% LiCl solution and extracted. The aqueous layer was extracted with 4:1 DCM/MeOH x 3, the organic layers were discarded and the combined aqueous layers were concentrated under a stream of nitrogen. The crude was dissolved in DMSO and purified by EZprep HpH method A first UV detection changed to 227 nm. Appropriate fractions were combined and concentrated under a stream of nitrogen to afford a colourless gum. Insufficient sample isolated for structural elucidation; LCMS corresponds with addition of GSH and OH, potentially caused by oxidation of the sulfur.

LCMS HpH retention time = 0.51 min, [M+H] = 770.29 (98%).

Synthesis of substrates for on-protein stability studies

Synthesis of methyl Boc-hydrazine[33a] S20

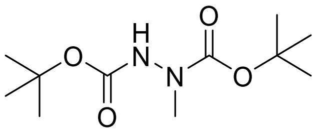

A solution of di-*tert*-butyl (*E*)-diazene-1,2-dicarboxylate (1.0 g, 4.4 mmol), in diethyl ether (21 mL) under nitrogen was cooled to -78°C before dropwise addition of 1.4 M methylmagnesium bromide solution in diethyl ether: toluene 3:1 (4.8 mL, 6.7 mmol) over 5 minutes. The reaction mixture was stirred at -78 °C for 45 min, and the suspension changed colour from yellow to white. The reaction was quenched by addition of aq. sat. NH_4Cl (25 mL) and warmed to 0 °C before further dilution with water (15 mL), where the organic solution turned pale yellow. The mixture was extracted with diethyl ether (3 x 20 mL), then the organic layers were combined, dried over a hydrophobic frit and concentrated under reduced pressure. The residue was purified on a normal phase silica column with a gradient of 0-20% EtOAc in cyclohexane and the appropriate fractions were combined and concentrated under reduced pressure to afford di-*tert*-butyl 1-methylhydrazine-1,2-dicarboxylate (825 mg, 77 % yield) as a colourless oil.
^{1}H NMR (400 MHz, $CDCl_3$) δ 6.46 - 6.39 (1H, s, br), 3.11 (3H, s), 1.49 (9H, s), 1.48 (9H, s) ppm. Rotamers observed.
Rf: 0.33 (15% EtoAc: 85% cyclohexane, visualised by ninhydrin).

Synthesis of *tert*-butyl ester[33a] S21

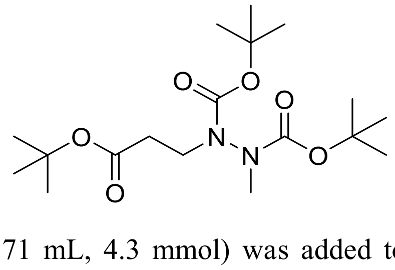

tert-Butyl 3-bromopropanoate (0.71 mL, 4.3 mmol) was added to a suspension of di-*tert*-butyl 1-methylhydrazine-1,2-dicarboxylate (693 mg, 2.82 mmol) and caesium carbonate (1.84 g, 5.64 mmol) in DMF (15 mL) and the reaction was stirred at rt for 19 h. The reaction mixture was diluted with water (20 mL) and extracted with diethyl ether (20 mL). The aqueous phase was further extracted with diethyl ether (2 x 20 mL) then the organic phases were combined, washed with 5% lithium chloride solution (2 x 20 mL), brine (20 mL), passed through a hydrophobic frit and concentrated under reduced pressure. The remaining solvent was azeotroped with toluene and concentrated under reduced pressure. The residue was purified on a normal phase silica column with a gradient of 0-15% EtOAc in cyclohexane. Appropriate fractions were combined and concentrated under reduced pressure to afford di-*tert*-butyl

1-(3-(*tert*-butoxy)-3-oxopropyl)-2-methylhydrazine-1,2-dicarboxylate (826 mg, 78% yield) as a colourless oil.
^{1}H NMR (400 MHz, $CDCl_3$) δ 3.66-3.38 (m, 2H), 2.85 (m, 3H), 2.03 (m, 2H), 1.29 (m, 27H) ppm.
Rf: 0.5 (15% EtOAc: 85% cyclohexane, visualised by potassium permanganate).

Synthesis of pyridazinedione acid[33a] S22

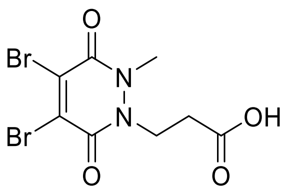

A solution of 2,3-dibromomaleic acid (450 mg, 1.6 mmol) in AcOH (4 mL) was heated to reflux and stirred for 45 min, prior to addition of di-*tert*-butyl 1-(3-(*tert*butoxy)-3-oxopropyl)-2-methylhydrazine-1,2-dicarboxylate (502 mg, 1.34 mmol) in AcOH (3 mL). The reaction mixture was left to reflux for 3.5 h, during the time the solution changed colour from colourless to yellow. The solution was azeotroped with toluene and concentrated under reduced pressure. The residue was dryloaded onto a normal phase silica column and purified with a gradient of 50-100% EtOAc [1% AcOH] in cyclohexane. Appropriate fractions were combined and concentrated under reduced pressure to afford 3-(4,5-dibromo-2-methyl-3,6-dioxo-3,6-dihydropyridazin-1(2*H*)-yl)propanoic acid (294 mg, 62% yield) as a yellow oil.

LCMS retention time = 0.58 min, [M+H] = 354.85, 356.86. 358.85 (88% purity)

^{1}H NMR (400 MHz, $CDCl_3$) δ 4.42 (t, J = 7.1 Hz, 2 H) 3.66 - 3.74 (s, 3 H) 2.75 - 2.85 (t, J = 7.2 Hz, 2 H) ppm. One exchangeable proton not observed.

Synthesis of succinic ester[33a] S23

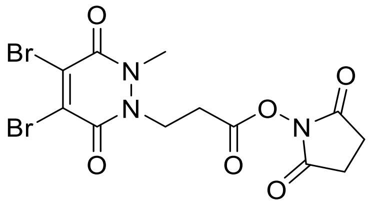

Dicyclohexylcarbodiimide (238 mg, 1.16 mmol) was added to a solution of 3-(4,5-dibromo-2-methyl-3,6-dioxo-3,6-dihydropyridazin-1(2*H*)-yl)propanoic acid (202 mg, 566 μmol in THF (10.6 mL) at 0 °C and the reaction mixture was stirred at 0 °C for 30 min. *N*-Hydroxysuccinimide (134 mg, 1.16 mmol) was added and the reaction was stirred at rt for 6 h. The reaction mixture was filtered under vacuum and the filtrate was concentrated under reduced pressure, then dryloaded onto a normal phase silica column and purified with a gradient of 0-100% EtOAc in cyclohexane. Appropriate fractions were combined and concentrated under reduced pressure then dried in the vacuum oven to afford 2,5-dioxopyrrolidin-1-yl 3-(4,5-dibromo-2-methyl-3,6-dioxo-3,6-dihydropyridazin-1(2*H*)-yl)propanoate (80.1 mg, 31% yield) as an orange/ yellow gummy solid.

LCMS retention time =0.68 min, [M+H] = 451.7, 453.85, 455.7 (81%)

^{1}H NMR (400 MHz, METHANOL-d_4) δ 4.53 (t, J = 7.0 Hz, 2 H) 3.68 (d, J = 1.0 Hz, 3 H) 3.13 - 3.19 (m, 2 H) 2.82 - 2.85 (m, 4 H) ppm.

HRMS (ESI+) Calculated for $[M+H]^+$, 451.9087, found 451.9088 Da.

IR: 1734 (C=O), 1299 (N-O), 1199 (N-N), 645 (C-Br) cm^{-1}.

Synthesis of BCN pyridazinedione 25

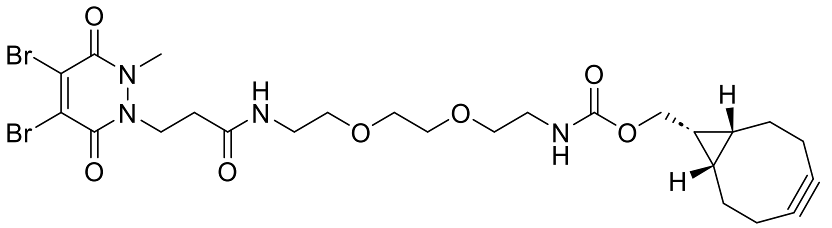

2,5-Dioxopyrrolidin-1-yl 3-(4,5-dibromo-2-methyl-3,6-dioxo-3,6-dihydropyridazin-1(2*H*)-yl)propanoate (44.5 mg, 98.2 μmol) was added to a solution of (1a,8a,9b)-bicyclo[6.1.0]non-4-yn-9-ylmethyl *N*-{2-[2-(2-aminoethoxy)ethoxy]ethyl}carbamate (39.6 mg, 122 μmol) in DMF (0.5 mL) and the reaction was stirred at room temperature for 1.5 h. The reaction mixture was directly purified by EZPrep formic method C with UV detection turned to 300 nm only and the appropriate fractions were combined and concentrated under reduced pressure. This was then dried under vacuum to afford ((1*R*,8*S*,9s)-bicyclo[6.1.0]non-4-yn-9-yl)methyl (2-(2-(2-(3-(4,5-dibromo-2-methyl-3,6-dioxo-3,6-dihydropyridazin-1(2*H*)-yl)propanamido)ethoxy)ethoxy)ethyl)carbamate (26.7 mg, 41%) as a pale yellow gum.

LCMS formic retention time = 0.96 min, [M+H] 660.92, 662.88, 664.81 (90%)

^{1}H NMR (400 MHz, DMSO-d_6) δ ppm 8.03 - 8.09 (m, 1 H) 7.01 - 7.07 (m, 1 H) 4.23 - 4.28 (m, 2 H) 4.00 - 4.04 (m, 2 H) 3.53 (s, 3 H) 3.48 (s, 3 H) 3.36 - 3.40 (m, 2 H) 3.32 - 3.36 (m, 2 H) 3.25 - 3.27 (m, 1 H) 3.13 - 3.17 (m, 2 H) 3.07 - 3.13 (m, 2 H) 2.41 - 2.47 (m, 2 H) 2.11 (br s, 6 H) 1.44 - 1.56 (m, 2 H) 1.20 - 1.29 (m, 1 H) 0.81 - 0.89 (m, 2 H).

^{13}C NMR (101 MHz, DMSO-d_6) δ ppm 169.2, 155.7, 152.6, 152.3, 135.2, 134.9, 98.9, 69.5, 69.4, 69.1, 68.9, 61.3, 43.8, 40.2, 38.5, 34.6, 33.1, 28.5, 20.8, 19.5, 17.6.

Rf: 0.13 (EtOAc, visualised under UV)

HRMS (ESI+) Calculated for $[M+Na]^+$, 683.0686, found 683.0674 Da.

IR: 3326 (N-H), 1626 (C=O), 1246 (C-N), 1095 (C-O), 557 (C-Br) cm^{-1}.

Synthesis of tetrazine pyridazinedione 26

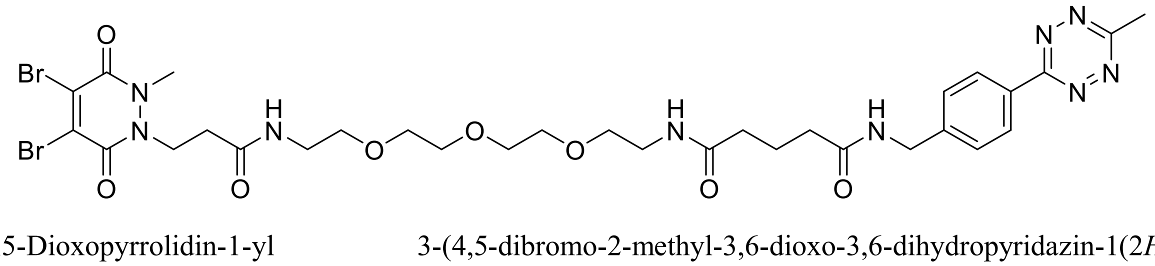

2,5-Dioxopyrrolidin-1-yl 3-(4,5-dibromo-2-methyl-3,6-dioxo-3,6-dihydropyridazin-1(2*H*)-yl)propanoate (8.4 mg, 18.5 μmol) was added to a mixture of *N*1-(2-(2-(2-(2-aminoethoxy)ethoxy)ethoxy)ethyl)-*N*5-(4-(6-methyl-1,2,4,5-tetrazin-3-yl)benzyl)glutaramide (8.8 mg, 18 μmol) in DMF (135 μL) and the reaction mixture was stirred at rt in air for 2h. The reaction mixture was directly purified using the EZ prep method B and appropriate fractions were combined and

concentrated under reduced pressure then dried under high vacuum to afford *N*1-(15-(4,5-dibromo-2-methyl-3,6-dioxo-3,6-dihydropyridazin-1(2*H*)-yl)-13-oxo-3,6,9-trioxa-12-azapentadecyl)-*N*5-(4-(6-methyl-1,2,4,5-tetrazin-3-yl)benzyl)glutaramide (5.1 mg, 34%) as a pink solid.

LCMS retention time = 0.81 min, [M+H] = 826.04, 827.99, 829.87 (96% purity)

^{1}H NMR (400 MHz, $CDCl_3$) δ ppm 8.50 - 8.58 (m, 2 H) 7.46 - 7.56 (m, 2 H) 6.97 - 7.04 (m, 1 H) 6.74 (br t, *J*=5.6 Hz, 1 H) 6.43 (br t, *J*=5.0 Hz, 1 H) 4.52 - 4.59 (m, 2 H) 4.37 - 4.44 (m, 2 H) 3.71 (s, 3 H) 3.61 (d, *J*=10.8 Hz, 8 H) 3.56 (br d, *J*=5.4 Hz, 2 H) 3.52 (s, 2 H) 3.38 - 3.45 (m, 4 H) 3.10 (s, 3 H) 2.58 - 2.67 (m, 2 H) 2.34 - 2.40 (m, 2 H) 2.25 - 2.32 (m, 2 H) 2.02 (t, *J*=7.1 Hz, 2 H).

^{13}C NMR (101 MHz, $CDCl_3$) δ ppm 172.8 (2C), 169.2, 167.3, 163.8, 152.9 (2C), 143.5, 136.3, 135.2, 130.9, 128.5, 128.2, 70.4 (2C), 70.1 (2C), 69.8 (2C), 44.5, 43.2, 39.4, 39.2, 35.4, 35.3, 35.0, 33.9, 21.9, 21.2.

Rf 0.82 (4:1 DCM:MeOH, visualised by UV)

HRMS (ESI+) Calculated for $[M+H]^+$, 826.1518, found 826.1504 Da.

IR: 3281 (N-H), 1627 (C=O) 1246 (C-N), 1110 (N-N), 1089 (C-O), 562 (C-Br) cm^{-1}.

Compatibility studies general procedure

Stock solutions:

1: 480 µL DMSO, 4.2 mL PBS, 360 µL internal standard (10 mM methyl *p*-tolyl sulfone in DMSO).

2: 50 mM each additive in PBS.

3: 10 mM click handle substrate in DMSO.

4: 120 µL DMSO, 90 µL internal standard (10 mM methyl p-tolyl sulfone in DMSO), 1.2 mL buffer.

CuAAC cocktail solution: 15 µL $CuSO_4$ solution (50 mM in PBS), 75 µL sodium ascorbate solution (50 mM in PBS) and 45 µL THPTA solution (50 mM in PBS).

A 384 well plate was loaded with: either 42 µL stock solution 1 or 47 µL stock solution 4, 5 µL stock solution 2 and 3 µL stock solution 3, into appropriate wells, before incubation at 37 °C in a thermomixer for 24 h and subsequent LCMS analysis. Three experimental replicates were performed.

Compatibility studies with capping general procedure

Given that maleimide **10** was unstable under the LCMS conditions and a major undesired reactivity product of TCO **12** is likely to be the isomerised form,[36] which is difficult to distinguish by LCMS, following the standard incubation detailed above, maleimide **10** and TCO **12** samples were capped to monitor any remaining active species. Maleimide **10** was capped using GSH due to the rapid reaction between maleimides and thiols. Note that the compatibility data indicates no instability of maleimide **10** to GSH (Figure S10), however in reality complete cross-reaction with GSH is observed. Similarly, TCO **12** was capped with an aryl-tetrazine, as the more stable *cis*-cyclooctene is much less reactive in IEDDA reactions.

Stock solutions:

1: 480 µL DMSO, 4.2 mL PBS, 360 µL internal standard (10 mM methyl *p*-tolyl sulfone in DMSO).

2: 50 mM each additive in PBS.

3: 10 mM click handle substrate in DMSO.

4: 120 μL DMSO, 90 μL internal standard (10 mM methyl *p*-tolyl sulfone in DMSO), 1.2 mL buffer.

5: 10 mM of either 3-phenyl-1,2,4,5-tetrazine or glutathione (10 mM in DMSO), for capping of TCO **12** and maleimide **10**, respectively.

CuAAC cocktail solution: 15 μL $CuSO_4$ solution (50 mM in PBS), 75 μL sodium ascorbate solution (50 mM in PBS) and 45 μL THPTA solution (50 mM in PBS).

A 384 well plate was loaded with: either 42 μL stock solution 1 or 47 μL stock solution 4, 5 μL stock solution 2 and 3 μL stock solution 3 into appropriate wells before incubation at 37 °C in a thermomixer for 24 h. 5 μL stock solution 5 was then added prior to analysis by LCMS. Three experimental replicates were performed.

Statistics for compatibility studies

Using maximum likelihood within the software platform JMP,[37] a separate linear regression model is fitted for each compound, with the raw peak area ratio used as the response and with condition number and plate ID included as categorical fixed effects. We do not include interaction of the three factors (response, condition number and plate ID) in the model. Residual diagnostic plots are made on all models to scrutinise the statistical assumptions underlying the modelling, and any necessary response transformations and/or outlier removals are performed. Residual diagnostics plots were deemed appropriate for all models, with only 3 data points out of 540 removed as gross outliers (one per compound for azide **2**, ketone **9**, and *exo*-norbornene **7**).

For each compound, pairwise comparisons of the mean peak area ratios are made between all pairs of conditions using the fitted regression model. The Tukey HSD multiplicity adjustment procedure with 5% significance level is applied to ensure that the family-wise type I error rate across all the comparisons within each compound is held below 5%. Finally, we declare that a condition causes instability to a compound if it has a significantly lower mean normalized peak area ratio compared with the condition estimated to have the highest value at the (multiplicity-adjusted) 5% significance level, assuming that at least one condition does not cause instability for each compound. Two-sided hypothesis tests are performed throughout the multiple comparisons and results are displayed in a connecting letters display table.

The plots in Figures S1-S14 come from the JMP analysis report. On the left is a Studentised Residual Plot with reference lines added. The points correspond to the 'Studentised' model residuals, each calculated as a raw residual (observed value minus the model-estimated value) divided by an estimate of the model standard error and scaled by the leverage. The chosen normalization approach for Studentised residuals enable reference lines to be drawn to assist with model residual diagnosis. The green lines correspond to pointwise 95% limits and the red lines correspond to Bonferroni simultaneous 95% limits. If the model assumptions are satisfied, we would anticipate around 1 in 20 points to fall outside of the green limits, and only 1 in 20 plots to contain one or more points outside the red bands. In the middle is a Least Squares Mean plot vs the 12 conditions. For each condition, the model-estimated mean peak area ratio is estimated, along with a 95% confidence interval for the mean. Note, these means are marginal means, in that they are averaged across the plate ID effects. Finally, on the right is a Connecting Letters Display Table, with Conditions ranked from highest to lowest according to their least squares mean estimate. Results of all pairwise statistical comparisons performed using Tukey's HSD procedure with a 5% experiment-wise Type I error rate are shown in the second column of the table with characters. The mean peak area ratios of two conditions are statistically significantly different, if and only if they do not share a common letter in the second column. We cannot say anything about two conditions sharing one or more common letter; we have neither shown such conditions to be

different, nor the same. Note: additional residual diagnostics plots, including residuals vs predicted and QQ plots, were also produced but did not show any severe departures from normality (not shown here).

Table S2 shows the condition numbers shown in this analysis and the conditions these are associated with.

Condition number	Condition name
1	pH 2.8
2	pH 4.0
3	pH 6.0
4	pH 7.2
5	pH 8.0
6	pH 10.0
7	GSH
8	TCEP
9	DTT
10	CuAAC conditions
11	DHA
12	Urea

Table S2. *Condition name correlation to condition number used in statistical analysis.*

Three outliers were removed from this analysis performed in JMP, due to unrealistic results believed to be caused by experimental run failure such as inaccurate pipetting or injection into LCMS.

This data is used in Figure 3 of the main text.

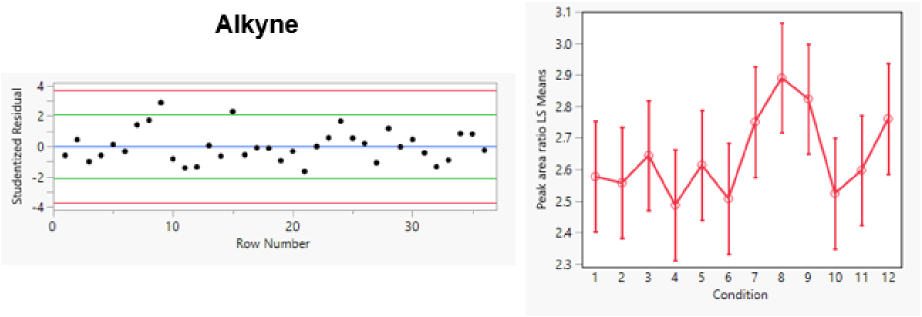

Condition		Least Squares Mean
8	A	2.8900000
9	A	2.8233333
12	A	2.7600000
7	A	2.7500000
3	A	2.6433333
5	A	2.6133333
11	A	2.5966667
1	A	2.5766667
2	A	2.5566667
10	A	2.5233333
6	A	2.5066667
4	A	2.4866667

Quantile = 3.63759, Adjusted DF = 22.0, Adjustment = Tukey

Figure S1. *Statistical analysis of alkyne* ***1*** *stability within 12 ligation conditions.*

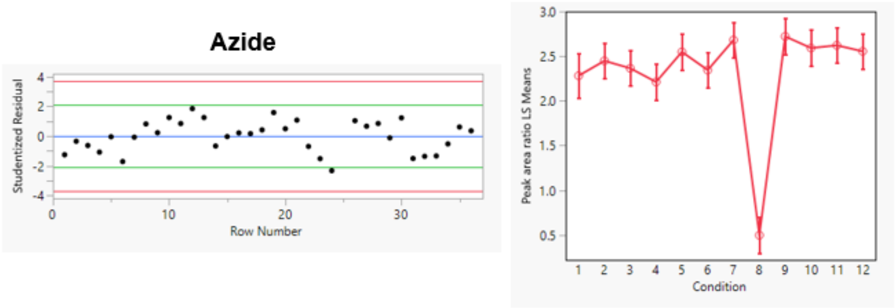

Condition				Least Squares Mean
9	A			2.7233333
7	A	B		2.6833333
11	A	B		2.6233333
10	A	B		2.5933333
12	A	B		2.5566667
5	A	B		2.5500000
2	A	B		2.4500000
3	A	B		2.3666667
6	A	B		2.3466667
1	A	B		2.2830303
4		B		2.2133333
8			C	0.4966667

Quantile = 3.65595, Adjusted DF = 21.0, Adjustment = Tukey-Kramer

Figure S2. *Statistical analysis of azide* ***2*** *stability within 12 ligation conditions.*

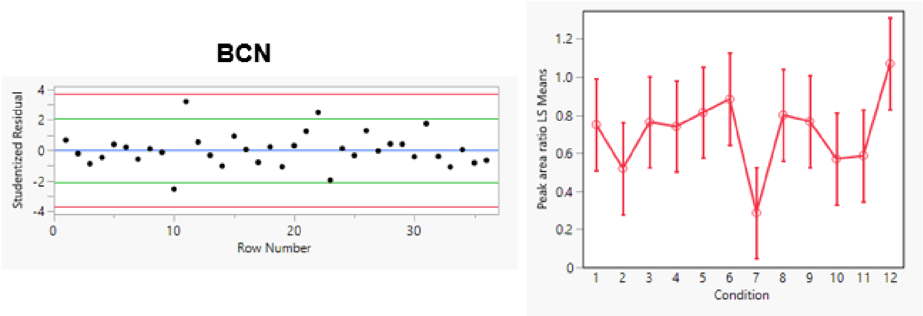

Condition		Least Squares Mean
12	A	1.0700000
6	A	0.8833333
5	A B	0.8133333
8	A B	0.8000000
9	A B	0.7666667
3	A B	0.7633333
1	A B	0.7500000
4	A B	0.7400000
11	A B	0.5866667
10	A B	0.5700000
2	A B	0.5200000
7	B	0.2866667

Quantile = 3.63759, Adjusted DF = 22.0, Adjustment = Tukey

Figure S3. *Statistical analysis of BCN* ***3*** *stability within 12 ligation conditions.*

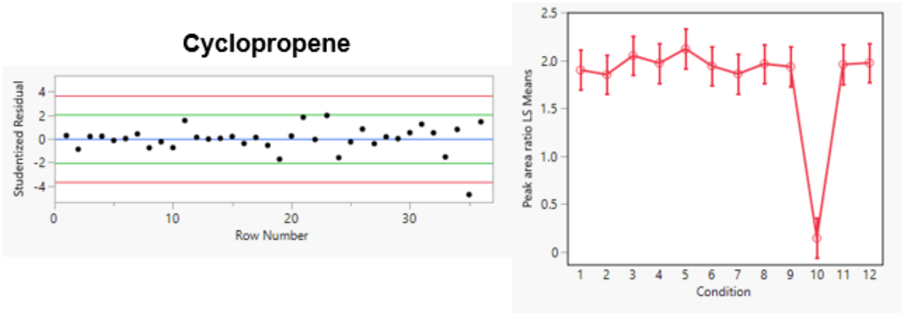

Condition		Least Squares Mean
5	A	2.1207000
3	A	2.0491945
12	A	1.9739978
4	A	1.9704573
8	A	1.9635767
11	A	1.9564584
6	A	1.9416606
9	A	1.9333599
1	A	1.8990999
7	A	1.8586862
2	A	1.8507872
10	B	0.1444943

Quantile = 3.63759, Adjusted DF = 22.0, Adjustment = Tukey

Figure S4. *Statistical analysis of cyclopropene* ***4*** *stability within 12 ligation conditions.*

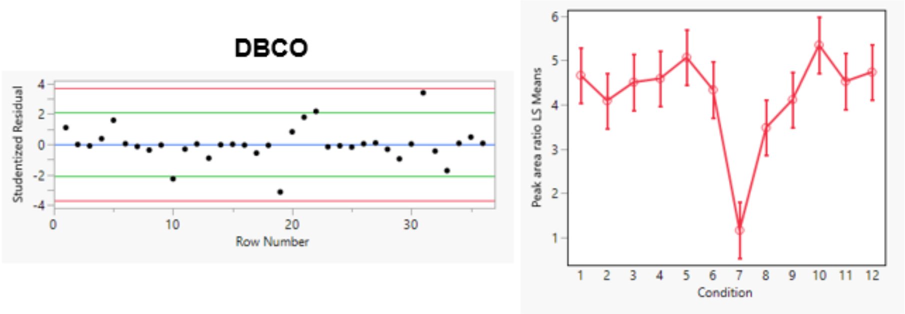

Condition		Least Squares Mean
10	A	5.3442516
5	A	5.0642450
12	A B	4.7348229
1	A B	4.6593935
4	A B	4.5880173
11	A B	4.5247126
3	A B	4.5045780
6	A B	4.3335795
9	A B	4.1105310
2	A B	4.0883724
8	B	3.4772516
7	C	1.1606101

Quantile = 3.63759, Adjusted DF = 22.0, Adjustment = Tukey

Figure S5. *Statistical analysis of DBCO* ***5*** *stability within 12 ligation conditions.*

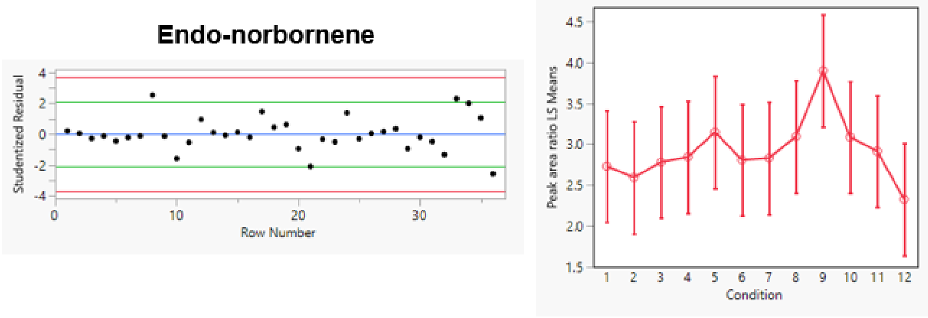

Condition		Least Squares Mean
9	A	3.8966667
5	A	3.1466667
8	A	3.0933333
10	A	3.0866667
11	A	2.9133333
4	A	2.8433333
7	A	2.8300000
6	A	2.8066667
3	A	2.7800000
1	A	2.7300000
2	A	2.5933333
12	A	2.3266667

Quantile = 3.63759, Adjusted DF = 22.0, Adjustment = Tukey

Figure S6*. Statistical analysis of endo-norbornene* ***6*** *stability within 12 ligation conditions.*

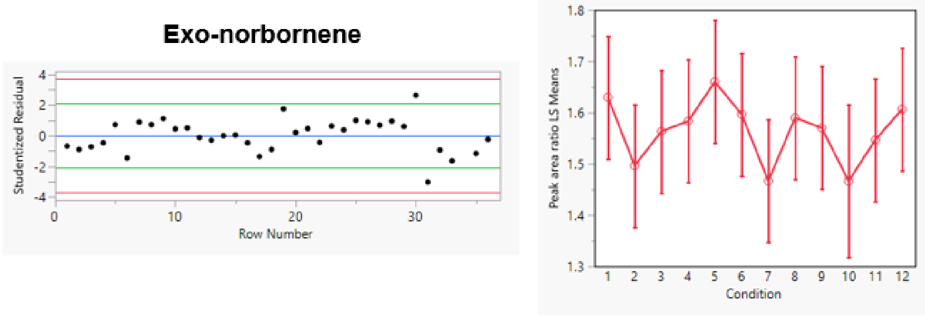

Condition		Least Squares Mean
5	A	1.6600000
1	A	1.6300000
12	A	1.6066667
6	A	1.5966667
8	A	1.5900000
4	A	1.5833333
9	A	1.5700000
3	A	1.5633333
11	A	1.5466667
2	A	1.4966667
7	A	1.4666667
10	A	1.4663636

Quantile = 3.65595, Adjusted DF = 21.0, Adjustment = Tukey-Kramer

Figure S7. Statistical analysis of exo-norbornene 7 stability within 12 ligation conditions.

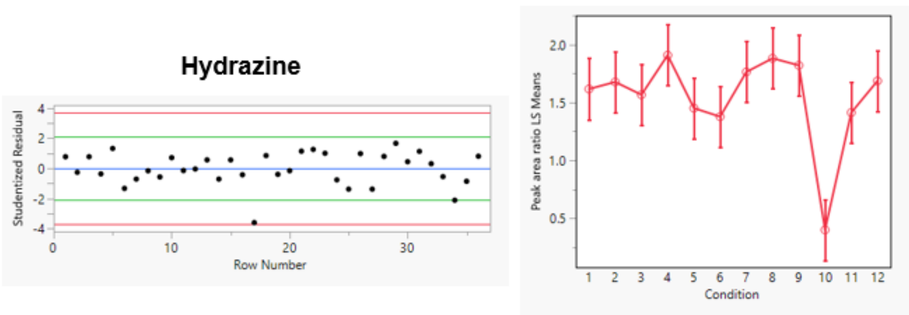

Condition			Least Squares Mean
4	A		1.9076386
8	A		1.8807673
9	A		1.8201276
7	A		1.7629507
12	A		1.6858666
2	A		1.6767455
1	A		1.6161590
3	A		1.5663372
5	A		1.4508298
11	A		1.4122470
6	A		1.3776899
10		B	0.4011286

Quantile = 3.63759, Adjusted DF = 22.0, Adjustment = Tukey

***Figure S8.** Statistical analysis of hydrazide **8** stability within 12 ligation conditions.*

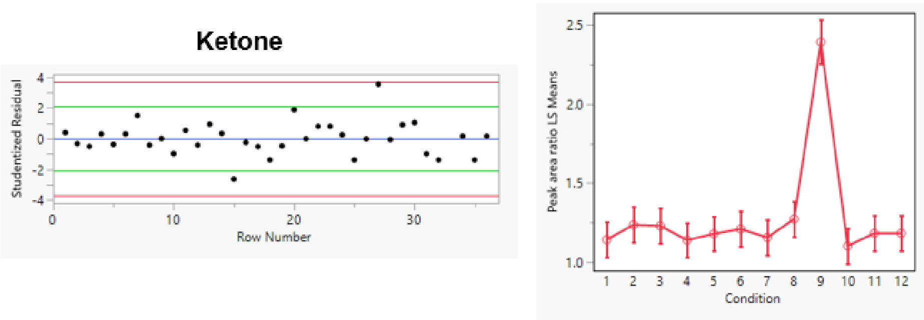

Condition			Least Squares Mean
9	A		2.3922727
8		B	1.2733333
2		B	1.2366667
3		B	1.2300000
6		B	1.2100000
11		B	1.1833333
12		B	1.1833333
5		B	1.1800000
7		B	1.1566667
1		B	1.1433333
4		B	1.1400000
10		B	1.1033333

Quantile = 3.65595, Adjusted DF = 21.0, Adjustment = Tukey-Kramer
Sample 9 exhibited overlapping LCMS signals resulting in a consistent anomaly and is therefore excluded from analysis.

***Figure S9.** Statistical analysis of ketone **9** stability within 12 ligation conditions.*

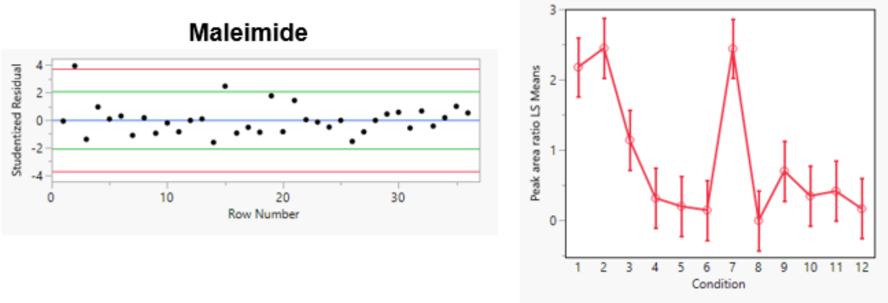

Condition		Least Squares Mean
2	A	2.4486316
7	A	2.4414847
1	A B	2.1790139
3	B C	1.1446682
9	C D	0.7046808
11	C D	0.4182639
10	C D	0.3504446
4	C D	0.3188235
5	C D	0.2016609
12	C D	0.1690098
6	C D	0.1486106
8	D	1.1102e-16

Quantile = 3.63759, Adjusted DF = 22.0, Adjustment = Tukey

Sample 7 appeared to show no degradation, however this condition is GSH and can be judged to show complete degradation due to GSH being the selected cap for maleimide experiments.

Figure S10. *Statistical analysis of maleimide **10** stability within 12 ligation conditions.*

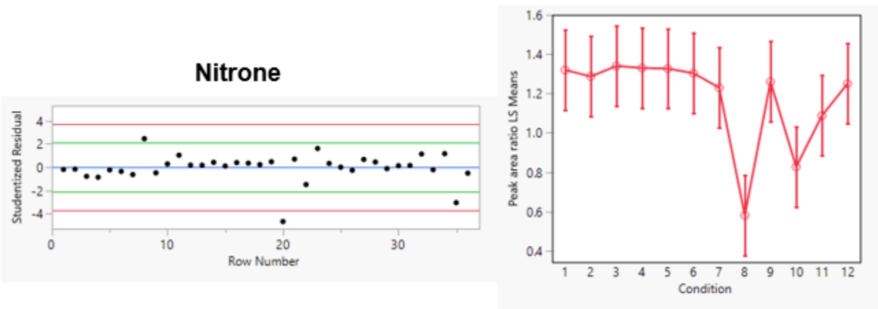

Condition		Least Squares Mean
3	A	1.3400000
4	A B	1.3300000
5	A B	1.3266667
1	A B	1.3200000
6	A B	1.3033333
2	A B	1.2866667
9	A B	1.2600000
12	A B	1.2500000
7	A B	1.2300000
11	A B	1.0866667
10	B C	0.8266667
8	C	0.5800000

Quantile = 3.63759, Adjusted DF = 22.0, Adjustment = Tukey

Figure S11. *Statistical analysis of nitrone **11** stability within 12 ligation conditions.*

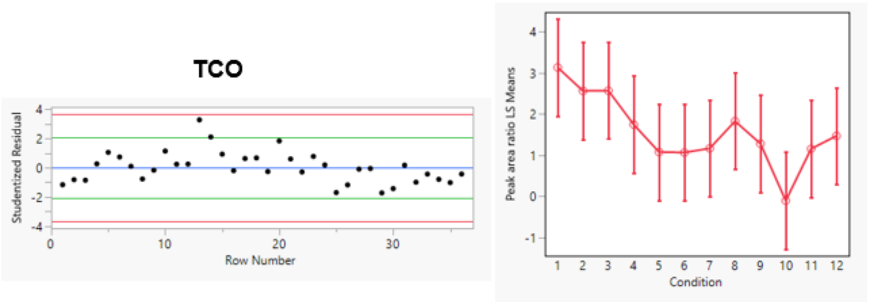

Condition		Least Squares Mean
1	A	3.135208
3	A B	2.571875
2	A B	2.565208
8	A B	1.828542
4	A B	1.748542
12	A B	1.471875
9	A B	1.285208
7	A B	1.168542
11	A B	1.158542
5	A B	1.081875
6	A B	1.068542
10	B	-0.101458

Quantile = 3.62089, Adjusted DF = 23.0, Adjustment = Tukey-Kramer

Figure S12. *Statistical analysis of TCO **12** stability within 12 ligation conditions.*

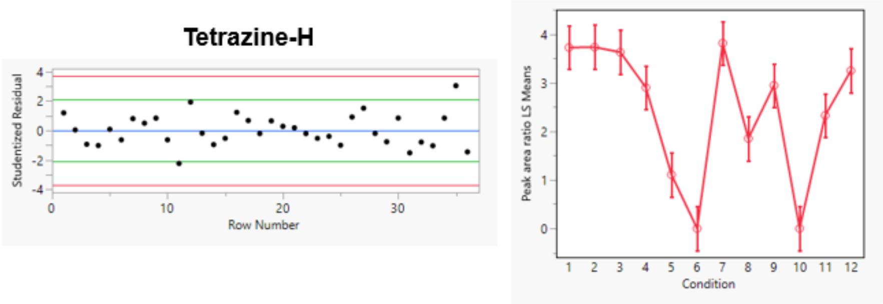

Condition		Least Squares Mean
7	A	3.8133333
2	A	3.7300000
1	A	3.7233333
3	A	3.6266667
12	A B	3.2466667
9	A B C	2.9433333
4	A B C	2.9000000
11	B C	2.3266667
8	C D	1.8533333
5	D E	1.1066667
10	E	8.8818e-16
6	E	4.4409e-16

Quantile = 3.63759, Adjusted DF = 22.0, Adjustment = Tukey

Figure S13. *Statistical analysis of tetrazine-H **13** stability within 12 ligation conditions.*

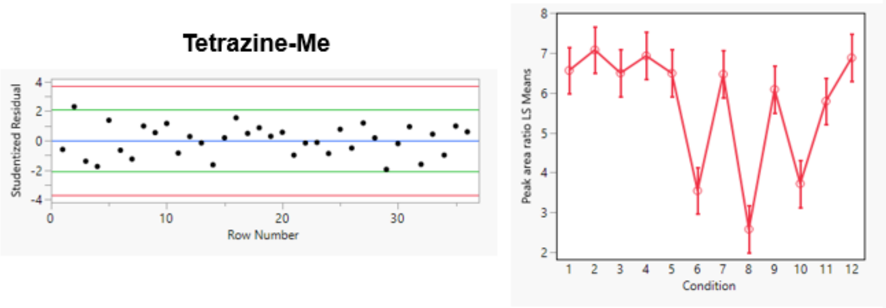

Condition			Least Squares Mean
2	A		7.0723494
4	A		6.9239434
12	A		6.8736680
1	A		6.5579820
3	A		6.4932022
5	A		6.4881606
7	A		6.4635581
9	A		6.0843692
11	A		5.7867795
10		B	3.7202706
6		B	3.5432097
8		B	2.5785566

Quantile = 3.63759, Adjusted DF = 22.0, Adjustment = Tukey

Figure S14. *Statistical analysis of tetrazine-Me **14** stability within 12 ligation conditions.*

Long term stability studies statistical analysis

Note: these analyses were carried out in the statistical software R instead of JMP. The procedure was identical, except for the use of a Box-Cox transform to sensibly choose a response transformation instead of the previous approach of a manual transformation selection.

These analyses were conducted in R using the *lm* function and these two default residual diagnostics plots are used to diagnose serious deviations from the statistical assumptions required for the regression model. On the left we see a plot of raw residuals vs model-predicted peak area ratio, with a mean smoother overlayed in red. Unmet model assumptions can be detected from this plot as a strong (nonzero) trend in the mean or variance of the residuals along the x-axis. Potentially problematic residuals, due to their size, leverage/influence, or otherwise, are numbered in the plot. Note that no outliers were removed in these automated analyses. The plot on the right is a normal QQ plot. Standardised residuals are ordered and then plotted alongside expected quantile values from a normal distribution. Unmet model assumptions here appear as strong departures from a straight-line trend. Note: residuals and model predictions appear on the box-cox transformed scale and not necessarily the peak area ratio scale.

Sample comments:

- Statistical analysis suggested no significant instability of DBCO **5** at room temperature over a four week period across any of the conditions tested. This however was not true, and in fact DBCO **5** showed instability in all of the conditions. This is an artifact of the assumptions made during the statistical analysis, where it is assumed that at least one combination of ligation condition and substrate was stable. It was found that each of these DBCO **5** combinations were in fact incompatible via manual inspection of the LCMS spectra.

Room temperature

Alkyne 1

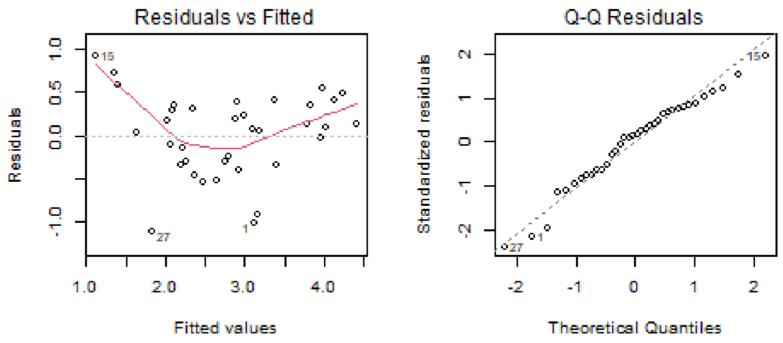

Figure S15. *Statistical analysis of alkyne* ***1*** *stability within 12 ligation conditions following a 4 week incubation at rt.*

Azide 2

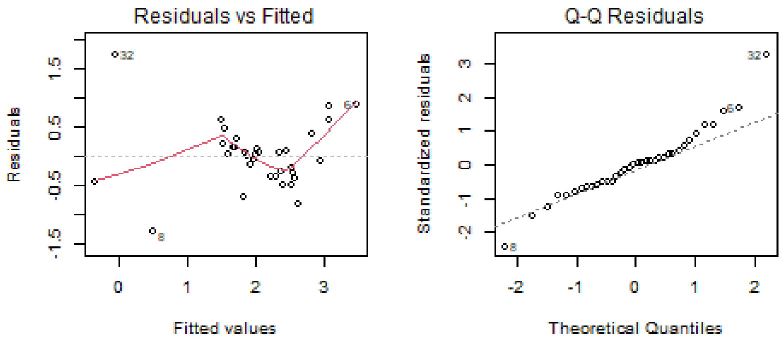

Figure S16. *Statistical analysis of azide* ***2*** *stability within 12 ligation conditions following a 4 week incubation at rt.*

BCN 3

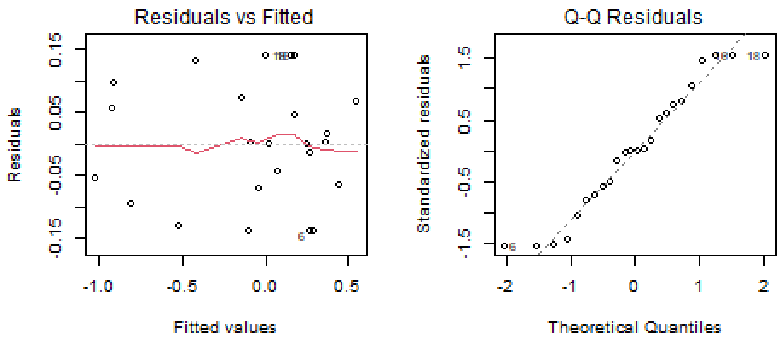

Figure S17. *Statistical analysis of BCN* ***3*** *stability within 12 ligation conditions following a 4 week incubation at rt.*

Cyclopropene 4

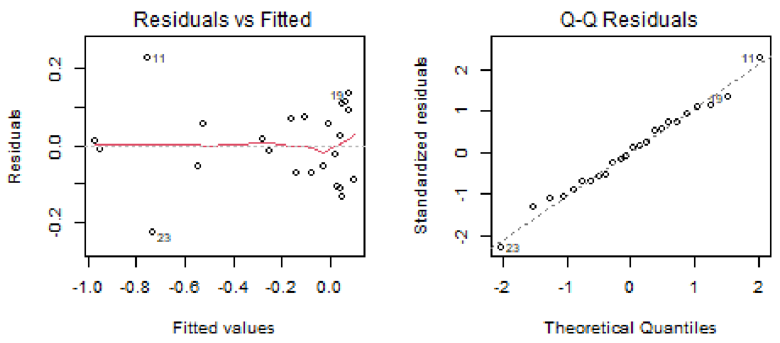

Figure S18. *Statistical analysis of cyclopropene* ***4*** *stability within 12 ligation conditions following a 4 week incubation at rt.*

DBCO 5

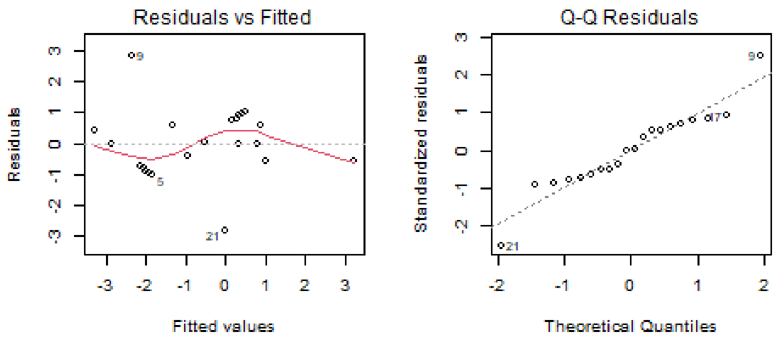

Figure S19. *Statistical analysis of DBCO* ***5*** *stability within 12 ligation conditions following a 4 week incubation at rt.*

Endo-norbornene 6

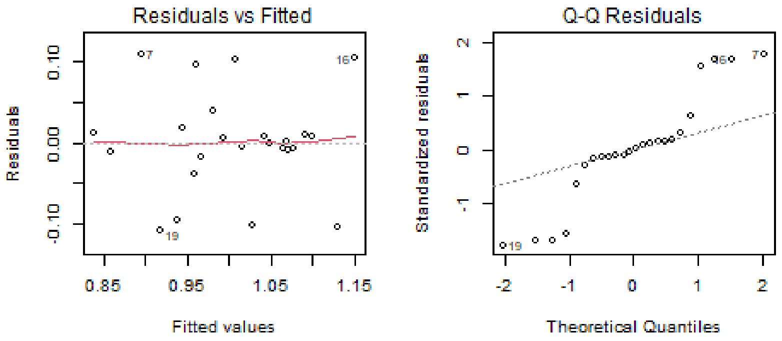

Figure S20. *Statistical analysis of endo-norbornene* ***6*** *stability within 12 ligation conditions following a 4 week incubation at rt.*

Exo-norbornene 7

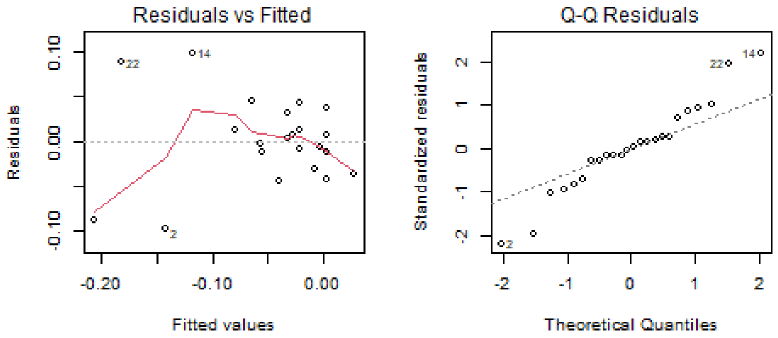

Figure S21. *Statistical analysis of exo-norbornene 7 stability within 12 ligation conditions following a 4 week incubation at rt.*

Hydrazide 8

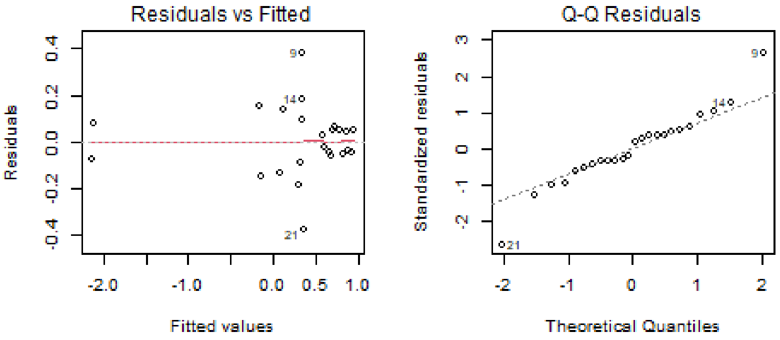

Figure S22*. Statistical analysis of hydrazide* ***8*** *stability within 12 ligation conditions following a 4 week incubation at rt.*

Ketone 9

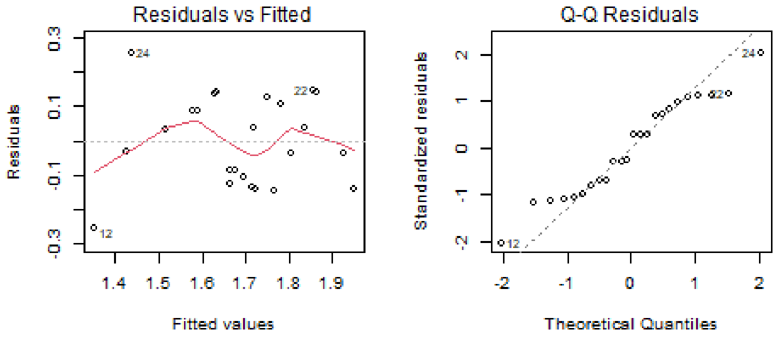

Figure S23. *Statistical analysis of ketone* ***9*** *stability within 12 ligation conditions following a 4 week incubation at rt.*

Maleimide 10

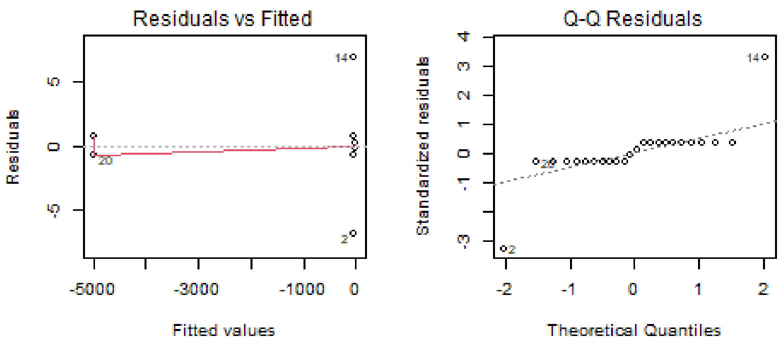

Figure S24. *Statistical analysis of maleimide **10** stability within 12 ligation conditions following a 4 week incubation at rt.*

Nitrone 11

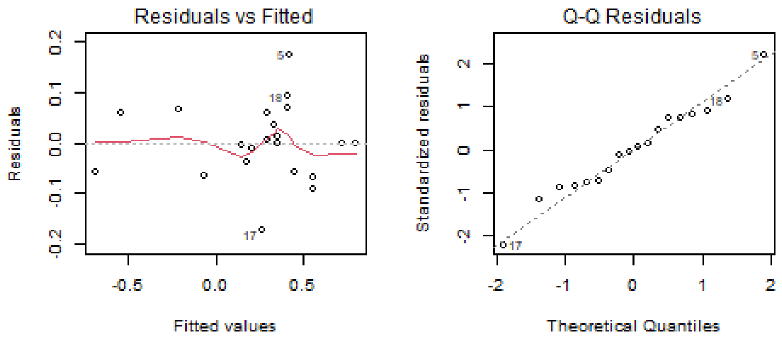

Figure S25. *Statistical analysis of nitrone **11** stability within 12 ligation conditions following a 4 week incubation at rt.*

TCO 12

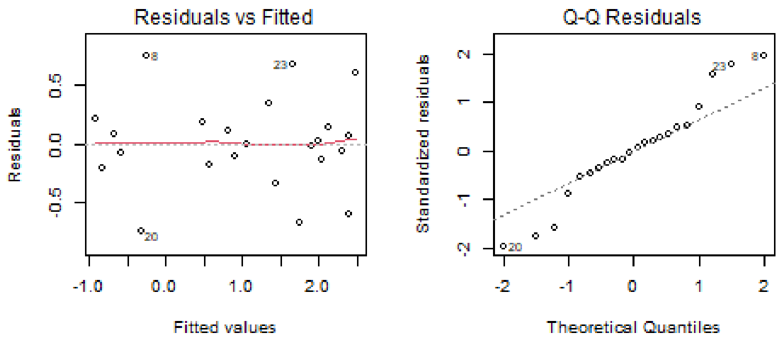

Figure S26. *Statistical analysis of TCO **12** stability within 12 ligation conditions following a 4 week incubation at rt.*

Tetrazine-H 13

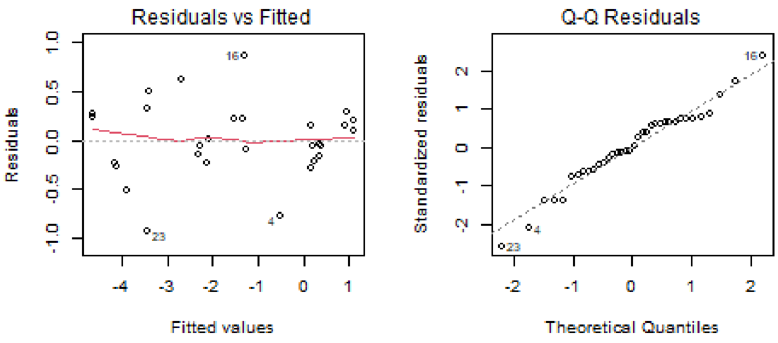

Figure S27. *Statistical analysis of tetrazine-H **13** stability within 12 ligation conditions following a 4 week incubation at rt.*

Tetrazine-Me 14

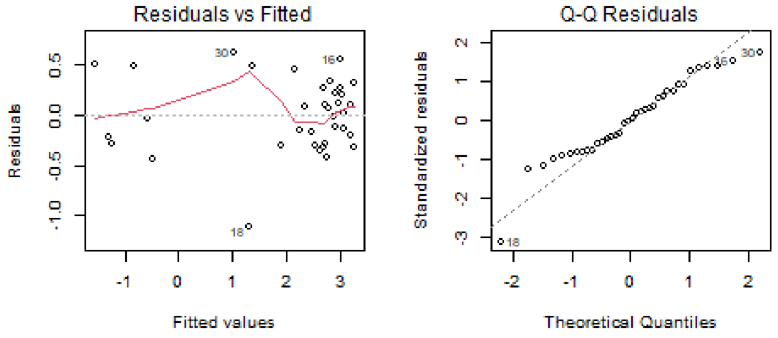

Figure S28. *Statistical analysis of tetrazine-Me **14** stability within 12 ligation conditions following a 4 week incubation at rt.*

Sub-ambient temperature (4 °C)

Alkyne 1

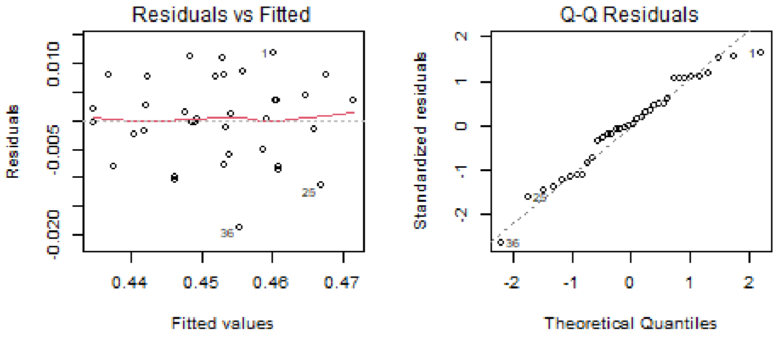

Figure S29. *Statistical analysis of alkyne **1** stability within 12 ligation conditions following a 4 week incubation at 4 °C.*

Azide 2

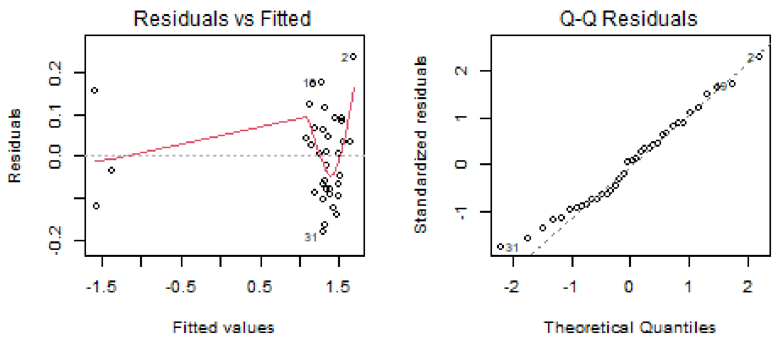

__Figure S30.__ Statistical analysis of azide __2__ stability within 12 ligation conditions following a 4 week incubation at 4 °C.

BCN 3

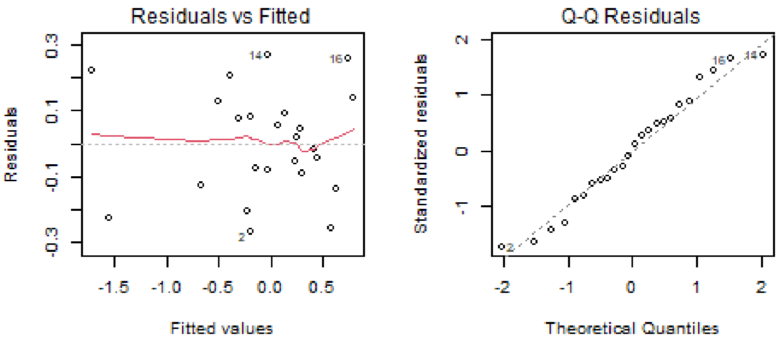

__Figure S31.__ Statistical analysis of BCN __3__ stability within 12 ligation conditions following a 4 week incubation at 4 °C.

Cyclopropene 4

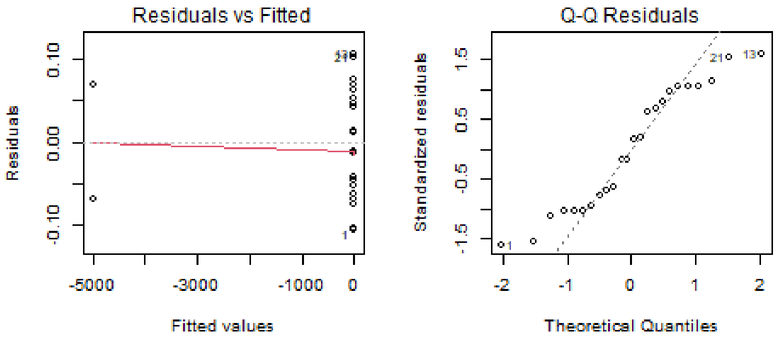

__Figure S32.__ Statistical analysis of cyclopropene __4__ stability within 12 ligation conditions following a 4 week incubation at 4 °C.

DBCO 5

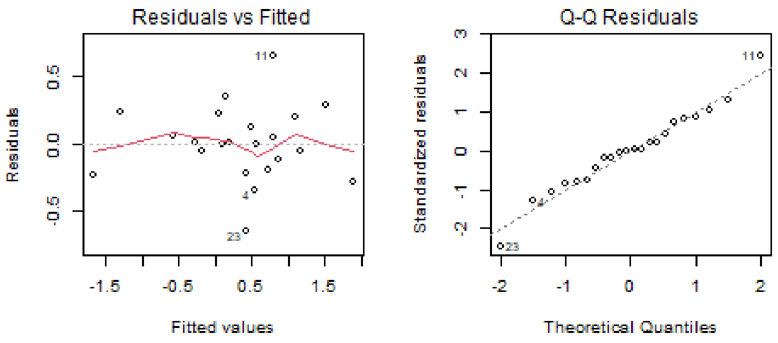

__Figure S33.__ Statistical analysis of DBCO ***5*** *stability within 12 ligation conditions following a 4 week incubation at 4 °C.*

Endo-norbornene 6

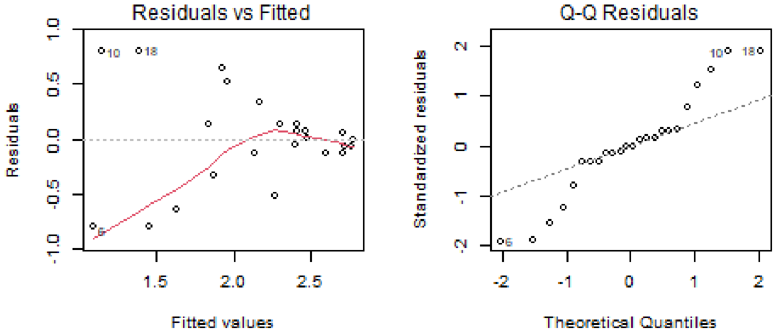

__Figure S34.__ Statistical analysis of endo-norbornene ***6*** *stability within 12 ligation conditions following a 4 week incubation at 4 °C.*

Exo-norbornene 7

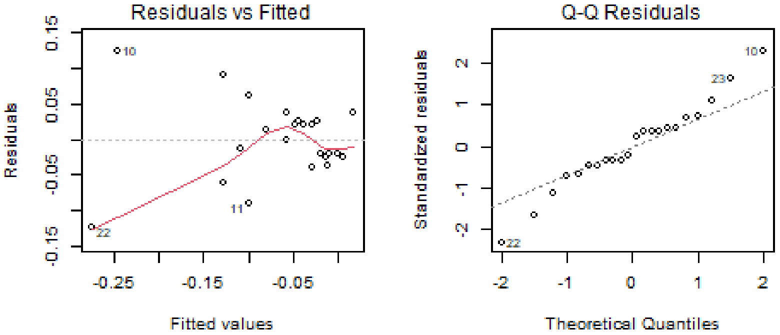

__Figure S35.__ Statistical analysis of exo-norbornene 7 stability within 12 ligation conditions following a 4 week incubation at 4 °C.

Hydrazide 8

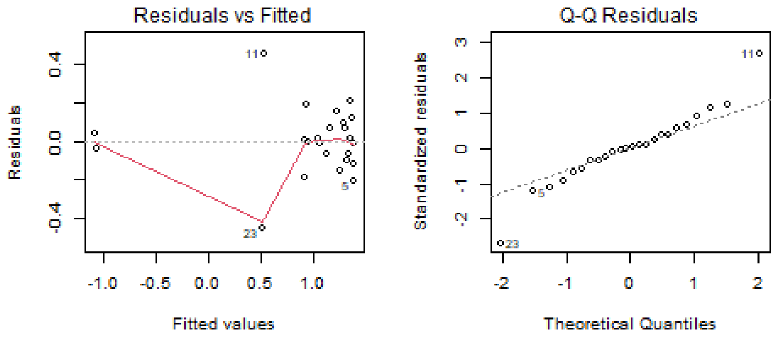

*Figure S36. Statistical analysis of hydrazide **8** stability within 12 ligation conditions following a 4 week incubation at 4 °C.*

Ketone 9

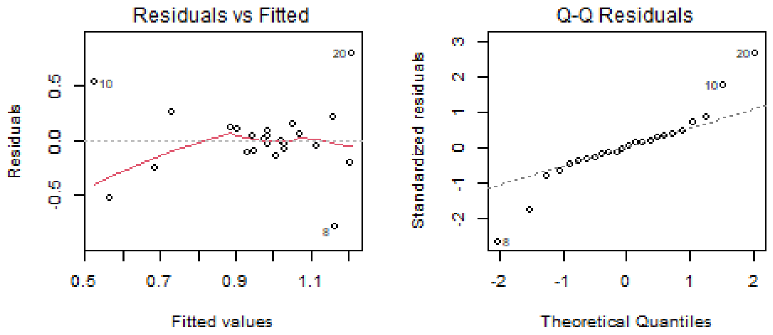

*Figure S37. Statistical analysis of ketone **9** stability within 12 ligation conditions following a 4 week incubation at 4 °C.*

Maleimide 10

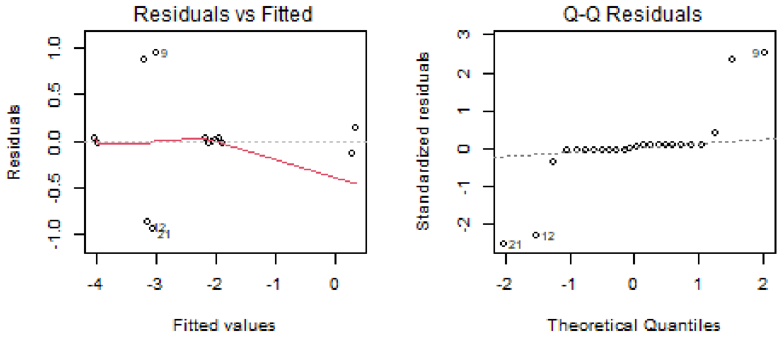

*Figure S38. Statistical analysis of maleimide **10** stability within 12 ligation conditions following a 4 week incubation at 4 °C.*

Nitrone 11

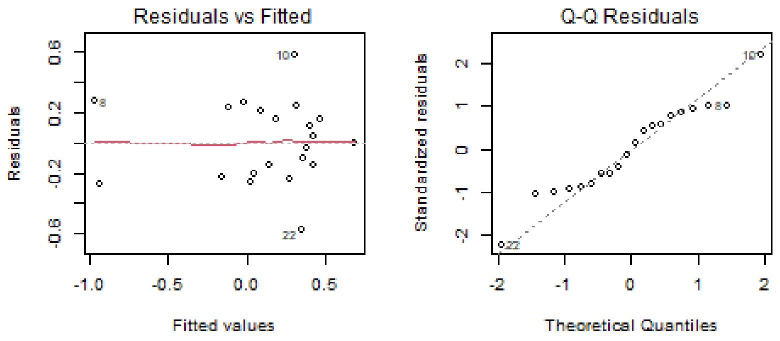

*Figure S39. Statistical analysis of nitrone **11** stability within 12 ligation conditions following a 4 week incubation at 4 °C.*

TCO 12

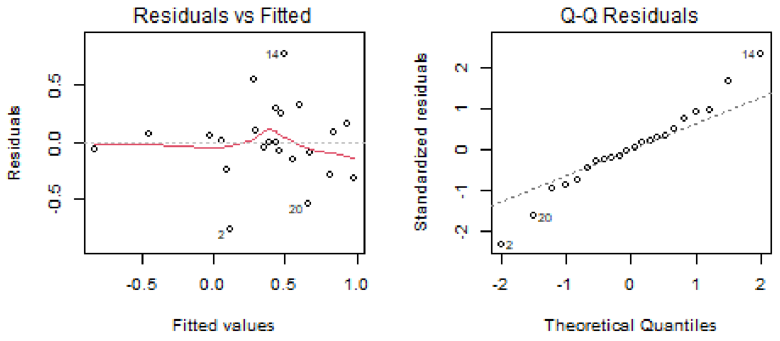

*Figure S40. Statistical analysis of TCO **12** stability within 12 ligation conditions following a 4 week incubation at 4 °C.*

Tetrazine-H 13

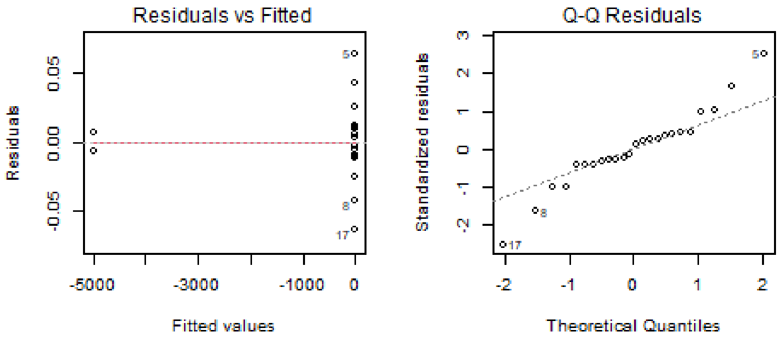

*Figure S41. Statistical analysis of tetrazine-H **13** stability within 12 ligation conditions following a 4 week incubation at 4 °C.*

Tetrazine-Me 14

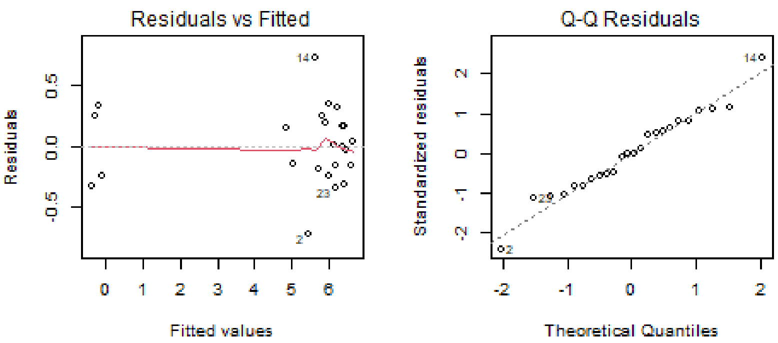

Figure S42. Statistical analysis of tetrazine-Me 14 stability within 12 ligation conditions following a 4 week incubation at 4 °C.

Kinetic studies

This data is used in Figure 4 of the main text.

Kinetic studies - general procedure

Stock solutions:

1: 96 µL DMSO, 840 µL or 960 µL buffer solution, internal standard solution (10 mM methyl *p*-tolyl sulfone in DMSO), 120 µL additive solution (50 mM of additive in DMSO) and 72 µL of click handle substrate solution (10 mM in DMSO).

50 µL of each stock solution was aliquoted into a 384 well plate before immediate analysis by LCMS (formic method, 37 °C, see general experimental for method details) and the peak area ratio between the click handle compound and internal standard was calculated.[23] The process was repeated on subsequent days until an n=3 was achieved. These values were plotted using Prism Graphpad using a one phase decay model.[38] Y(t=0) was set to 1 and the plateau was set to 0. Half-lives were determined using the software, using pseudo first order rate constants.

Kinetic studies - capping general procedure

Stock solutions:

1: 96 µL DMSO, 840 µL or 960 µL buffer solution, internal standard solution (10 mM methyl *p*-tolyl sulfone in DMSO), 120 µL additive solution (50 mM of additive in DMSO) and 72 µL of click handle substrate solution (10 mM in DMSO).

2: 10 mM 3-phenyl-1,2,4,5-tetrazine in DMSO

3: 10 mM GSH in DMSO

The first column of a 384 well plate was preloaded with 5 µL of stock solution 2 or 3. 50 µL of each stock solution was added to the plate, then was immediately transferred to a hotplate set to 37 °C. 5 uL stock solution 2 or 3 was added to subsequent columns at regular intervals until the timecourse was complete. The plate was then analysed by LCMS (formic method, 37 °C, see general experimental for method details) and the peak area ratio between the click handle compound and internal standard was calculated.[23] The process was repeated on subsequent days until an n=3 was achieved. These values were plotted using Prism Graphpad using a one phase decay model.[38] Y(t=0) was set to 1 and the plateau was set to 0. Half-lives were determined using the software, using pseudo first order rate constants.

Kinetics comments:

- No accurate half-life for the reactions between DTT or TCEP with maleimide **10** could be calculated due to kinetics more rapid than the time taken to load the samples onto the LCMS machine. T=0 min timepoints showed complete conversion from maleimide **10**.
- Very slow maleimide **10** kinetics in DHA demonstrated that initial observations of maleimide degradation in DHA were in fact due to degradation of maleimide **10** in the reaction buffer at pH 7.2 rather than the DHA additive.

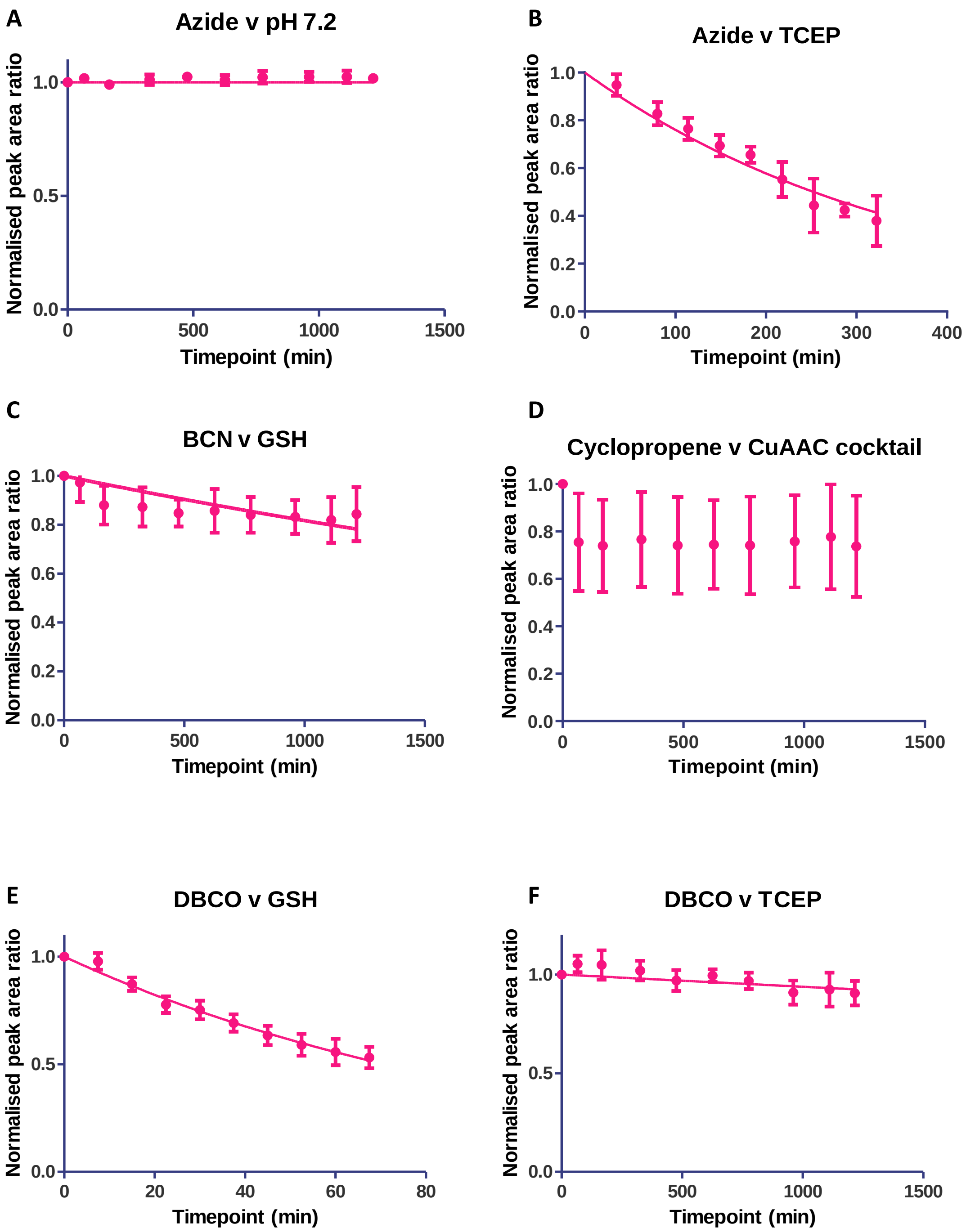

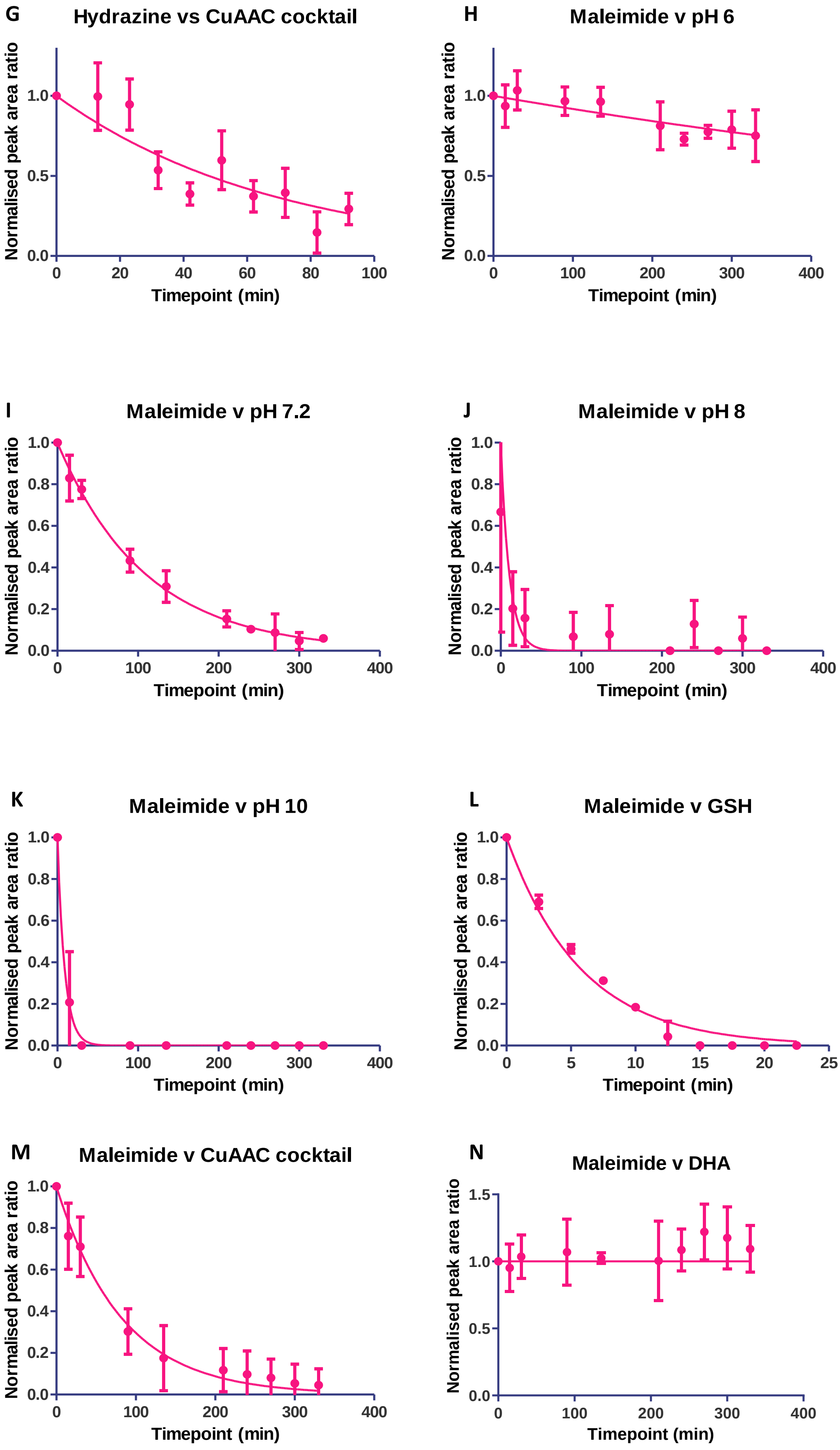
G
Hydrazine vs CuAAC cocktail
Normalised peak area ratio
Timepoint (min)
H
Maleimide v pH 6
Normalised peak area ratio
Timepoint (min)
I
Maleimide v pH 7.2
Normalised peak area ratio
Timepoint (min)
J
Maleimide v pH 8
Normalised peak area ratio
Timepoint (min)
K
Maleimide v pH 10
Normalised peak area ratio
Timepoint (min)
L
Maleimide v GSH
Normalised peak area ratio
Timepoint (min)
M
Maleimide v CuAAC cocktail
Normalised peak area ratio
Timepoint (min)
N
Maleimide v DHA
Normalised peak area ratio
Timepoint (min)

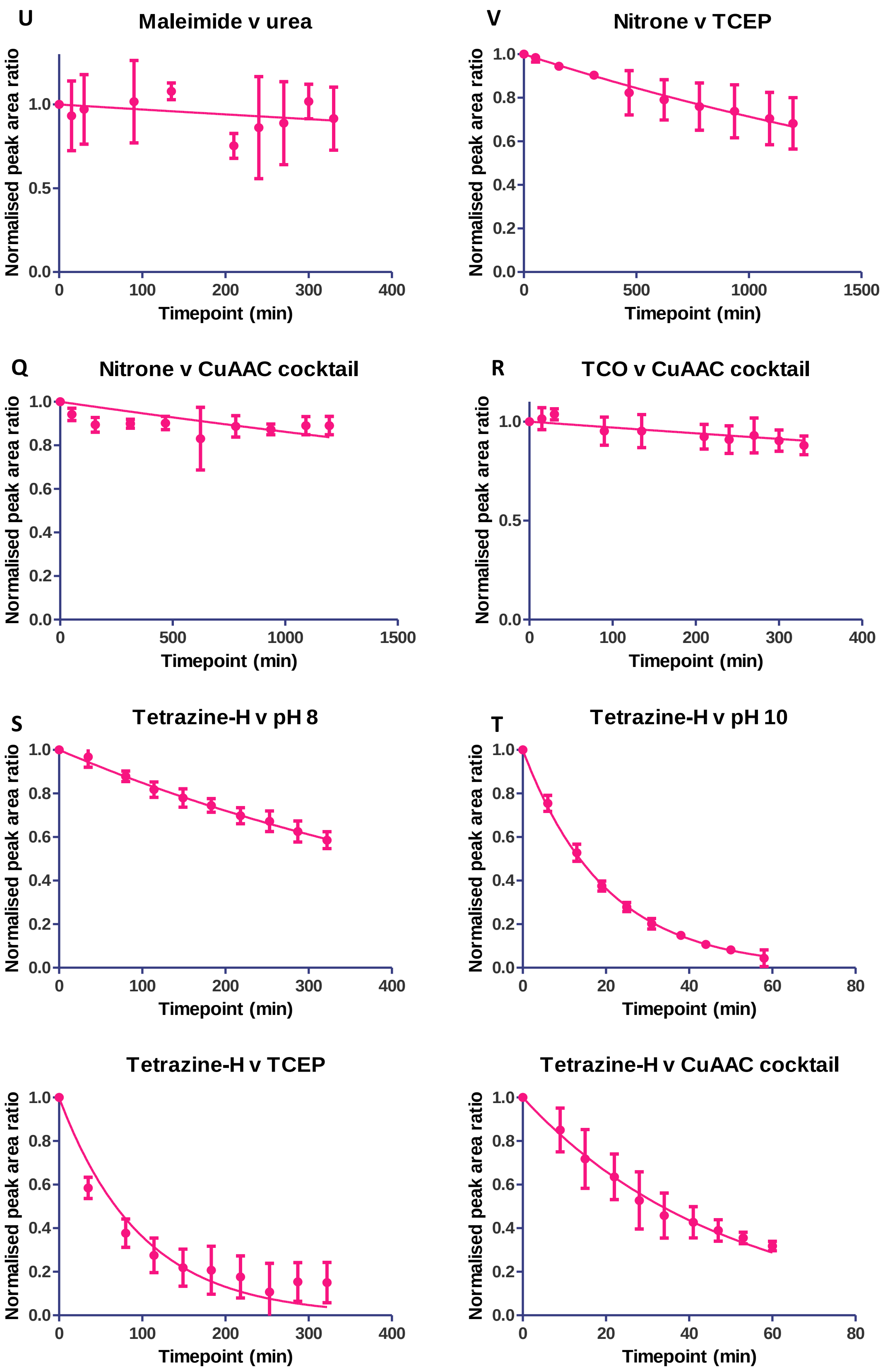
U
Maleimide v urea
Normalised peak area ratio
0.0
0.5
1.0
0
100
200
300
400
Timepoint (min)
V
Nitrone v TCEP
Normalised peak area ratio
0.0
0.2
0.4
0.6
0.8
1.0
0
500
1000
1500
Timepoint (min)
Q
Nitrone v CuAAC cocktail
Normalised peak area ratio
0.0
0.2
0.4
0.6
0.8
1.0
0
500
1000
1500
Timepoint (min)
R
TCO v CuAAC cocktail
Normalised peak area ratio
0.0
0.5
1.0
0
100
200
300
400
Timepoint (min)
S
Tetrazine-H v pH 8
Normalised peak area ratio
0.0
0.2
0.4
0.6
0.8
1.0
0
100
200
300
400
Timepoint (min)
T
Tetrazine-H v pH 10
Normalised peak area ratio
0.0
0.2
0.4
0.6
0.8
1.0
0
20
40
60
80
Timepoint (min)
Tetrazine-H v TCEP
Normalised peak area ratio
0.0
0.2
0.4
0.6
0.8
1.0
0
100
200
300
400
Timepoint (min)
Tetrazine-H v CuAAC cocktail
Normalised peak area ratio
0.0
0.2
0.4
0.6
0.8
1.0
0
20
40
60
80
Timepoint (min)

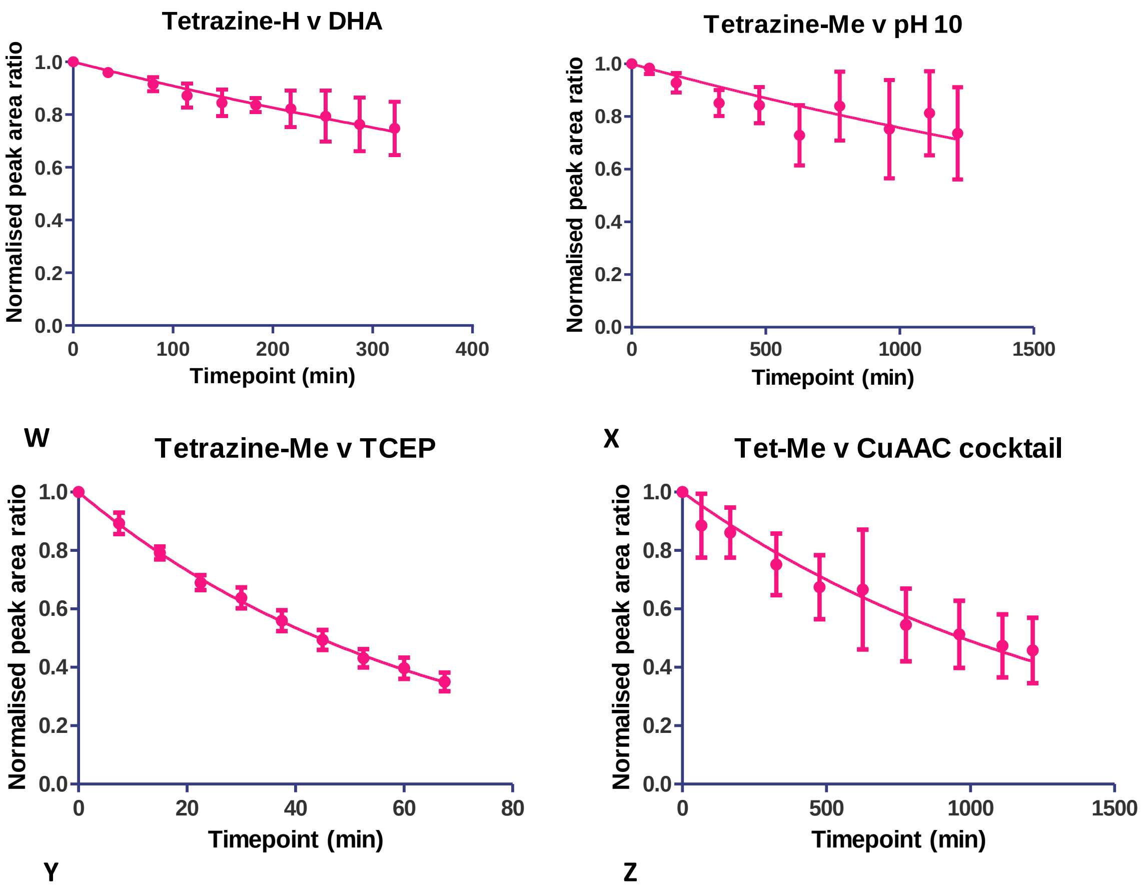

Figure S43. *Kinetic plots for click handle/ ligation condition combinations observed to be incompatible in initial experiments (Figure 3), taken in triplicate over 10 timepoints. (A) Azide **2** v pH 7.2, (B) Azide **2** v TCEP, (C) BCN **3** v GSH, (D) cyclopropene **4** v CuAAC cocktail, (E) DBCO **5** v GSH, (F) DBCO **5** v TCEP, (G) hydrazide **8** v CuAAC cocktail, (H) maleimide **10** v pH 6, (I) maleimide **10** v pH 7.2, (J) maleimide **10** v pH 8, (K) maleimide **10** v pH 10, (L) maleimide **10** v GSH, (M) maleimide **10** v CuAAC cocktail, (N) maleimide **10** v DHA, (O) maleimide **10** v urea, (P) nitrone **11** v TCEP, (Q) nitrone **11** v CuAAC cocktail, (R) TCO **12** v CuAAC cocktail, (S) tetrazine-h **13** v pH 8, (T) tetrazine-h **13** v pH 10, (U) tetrazine-h **13** v TCEP, (V) tetrazine-h **13** v CuAAC cocktail, (W) tetrazine-h **13** v DHA, (X) tetrazine-Me **14** v pH 10,(Y) tetrazine-Me **14** v TCEP and (Z) tetrazine-Me **14** v CuAAC cocktail.*

The half-life of kinetics was calculated from the plots in Figure S43, are summarised in Table S3 and the bar charts in Figure S44. An accurate average half-life could not be calculated for half-life values of over 24 hours due to the experiment timeframe, therefore half-lives are reported as ≥1440 min and are not included in the bar charts (Fig. S44). The bar charts y-axes are split into two segments at 95 min to enable visualisation of the full half-life range.

Compound/ condition combination	Average half-life (min)
Azide/TCEP	252.9
BCN/GSH	356.9
Cyclopropene/CuAAC cocktail	≥1440
DBCO/GSH	70.95
DBCO/TCEP	≥1440
Hydrazine/CuAAC cocktail	48.00
Maleimide/pH 6	810.3
Maleimide/pH 7.2	75.96
Maleimide/pH 8	8.597
Maleimide/pH 10	6.394
Maleimide/GSH	4.000
Maleimide/TCEP	4.058
Maleimide/DTT	22.80
Maleimide/CuAAC cocktail	56.98
Maleimide/DHA	≥1440
Maleimide/Urea	≥1440
Nitrone/TCEP	756.0
Nitrone/CuAAC cocktail	≥1440
Tet-H/pH 8	365.5
Tet-H/pH 10	13.88
Tet-H/TCEP	52.86
Tet-H/CuAAC cocktail	241.1
Tet-H/DHA	≥1440
Tet-Me/pH 10	245.7
Tet-Me/TCEP	43.60
Tet-Me/CuAAC cocktail	483.6
Azide/pH 7.2	≥1440

Table S3. *Summary table of half-lives determined from kinetic studies for each click handle/ ligation condition combination, taken in triplicate.*

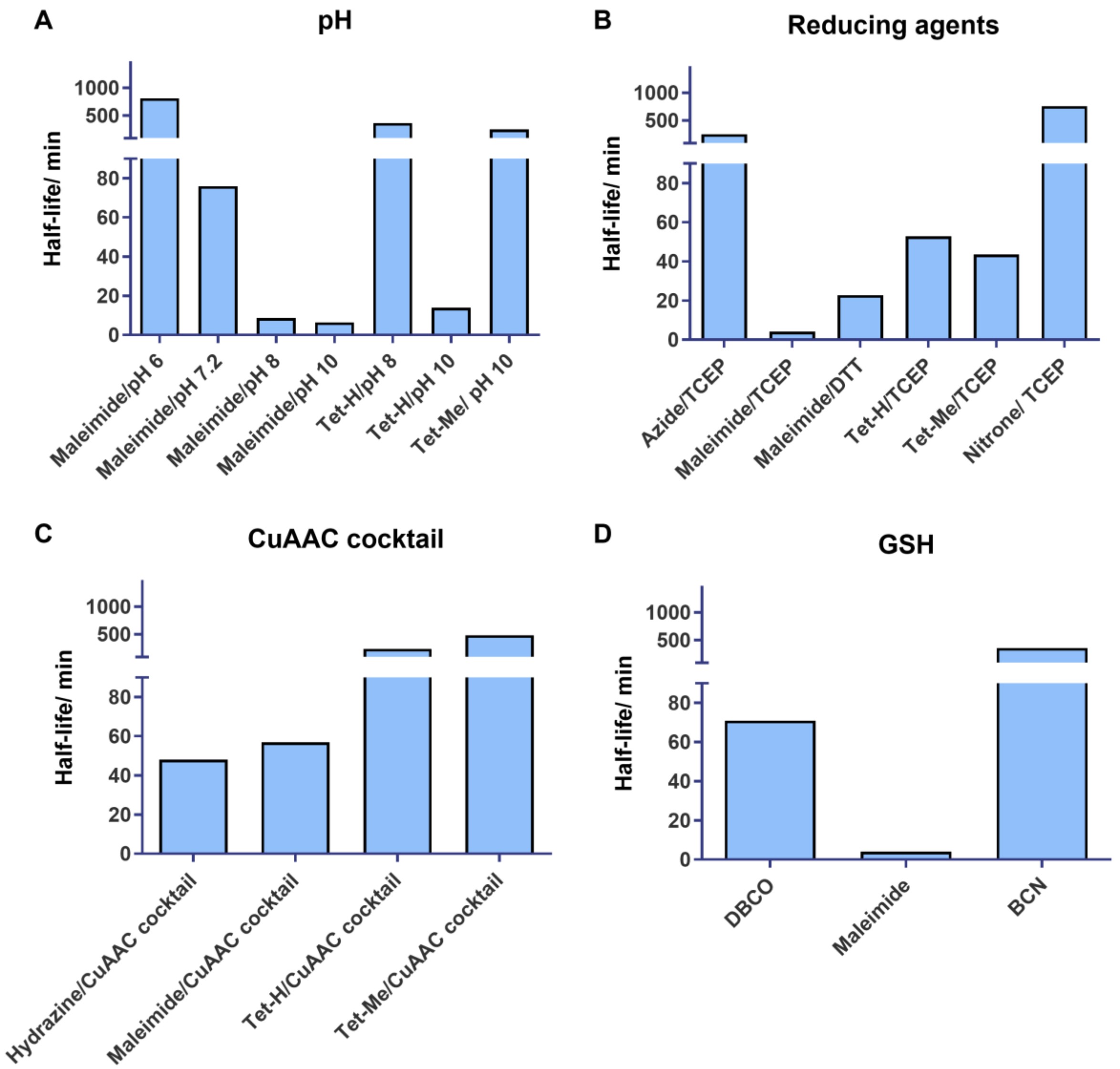

Figure S44*. Summary bar charts of half-lives determined from kinetic studies for each click handle/ ligation condition combination, calculated from triplicate experimental runs. (A) Maleimide* ***10****, tetrazine-H* ***13*** *and tetrazine-me* ***14*** *in pH buffers 6-10. (B) Azide* ***2****, maleimide* ***10****, tetrazine-h* ***13****, tetrazine-Me* ***14*** *and nitrone* ***11*** *in reducing agents: TCEP and DTT. (C) Hydrazide* ***8****, maleimide* ***10****, tetrazine-h* ***13*** *and tetrazine-Me* ***14*** *in CuAAC cocktail. (D) BCN* ***3****, DBCO* ***5*** *and maleimide* ***10*** *in GSH.*

Solubility assessment

Solubility of the constructs listed in Table S4 was measured using a Charged Aerosol Detector (CAD) solubility assay, data reported here as the mean of two experimental replicates.[39] Relative solubility of was attributed to the click handle only, as the R-group (Fig. 2) was conserved across each of the compounds. $LogD_{7.4}$ is also reported.

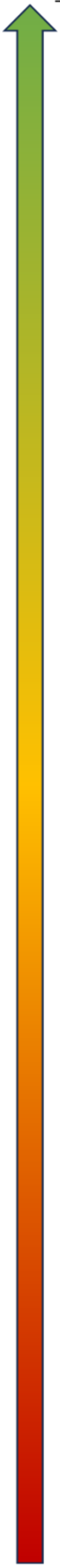

Compound	Solubility/ µM	LogD pH 7.4
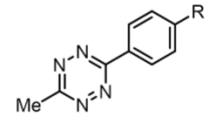 Tetrazine-Me **14**	≥694	0.825
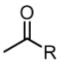 Ketone **9**	≥677	0.595
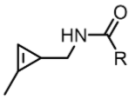 Cyclopropene **4**	≥618	0.510
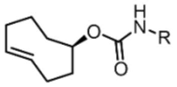 TCO **12**	≥611	2.37
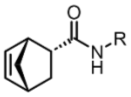 Endo-norbornene **6**	≥562	1.17
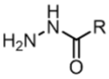 Hydrazine **8**	≥548	0.050
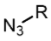 Azide **2**	≥539	0.880
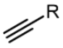 Alkyne **1**	≥393	0.600
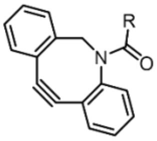 DBCO **5**	≥285	2.31
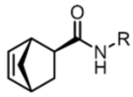 Exo-norbornene **7**	≥215	0.825
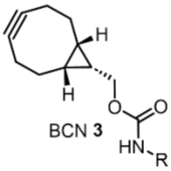 BCN **3**	≥205	
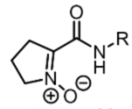 Nitrone **11**	≥169	0.710
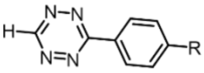 Tetrazine-H **13**	≥123	1.17

Table S4. *Solubility and LogD (see general experimental for details) ranking of click handle containing compounds. Listed from most to least soluble in PBS pH 7.4 in descending order. Note, maleimide was not included due its lack of stability in water, and BCN logD$_{7.4}$ was not measured due to apparent instability of the species during the assay.*

Fab generation

Fabs generated were used in the experiment described in Figure 5.

pTT5-based expression vectors encoding the heavy chain VH-CH1 domains and the light chain of an antibody fragment (Fab) were generated at Twist Bioscience.

The plasmids were used, together with PEI (product code R103D), to transiently express the Fab construct in HEK293-6E cells. 6 d post-transfection cell suspensions were sterile filtered prior to protein purification using Capto-L purification beads (product code 17547806). Purified protein was buffer exchanged into PBS prior to use in conjugation experiments.

On protein stability assessment procedure

Fab solution (1 mL at 5 mg/mL) was buffer exchanged into borate buffered saline (BBS) before reduction of interchain disulfide bonds with TCEP (30 µL, 100 mM in water). The solution was left at rt for 4 h before a buffer exchange into BBS without reducing agent. Either BCN **25** or tetrazine **26** (8.4 µL, 20 mM in DMF) were added to the reduced Fab solution, and samples incubated at 37 °C for 30 min with 500 rpm shaking. Small molecules were then removed by buffer exchange into PBS before splitting each sample into eight aliquots, all diluted to 100 µL in PBS. To each of the two Fab conjugates was added TCEP, GSH, CuAAC conditions or left in PBS (additives added from 400 mM stock solutions in water to produce 5 mM final concentration solutions. CuAAC conditions were pre-mixed in a 1:5:10 ratio of $CuSO_4$, THPTA and sodium ascorbate, final concentrations 1 mM, 5 mM and 10 mM respectively). Samples were incubated at 37 °C for 24 h. Small molecules were removed by buffer exchange into PBS before capping with corresponding click handles BCN **3** and tetrazine **14** (1.6 µL of 10 mM solutions in DMSO). Each reaction was incubated at rt for 21 h with 500 rpm shaking before a final buffer exchange into PBS, ahead of reduced LCMS and SDS-PAGE analysis. Two experimental runs were carried out at the same time, but the Fab conjugations and click reactions were carried out separately.

This data is used in Figure 5 of the main text.

**Repeat number**	**Click handle**	**Condition**	**Expected clicked mass/ Da**	**Measured clicked mass/ Da**	**Clicked mass intensity/ counts**	**Measured non-clicked mass/ Da**	**Non-clicked mass intensity/ counts**
N=1	BCN **25**	TCEP	48364	48364	25371945	47947	38103855
N=1	BCN **25**	GSH	48364	48364	40502296	47945	10914297
N=1	BCN **25**	CuAAC conditions	48364	48365	1360051	-	0
N=1	BCN **25**	pH 7.2	48364	48364	51810444	47944	9826598
N=1	Tetrazine **26**	TCEP	48493	48493	47753672	48076	16457254

N=1	Tetrazine **26**	GSH	48493	48493	77997343	-	0
N=1	Tetrazine **26**	CuAAC conditions	48493	48493	1923494	48081	1890327
N=1	Tetrazine **26**	pH 7.2	48493	48493	57218541	-	0
N=2	BCN **25**	TCEP	48364	48364	25004452	47950	32106784
N=2	BCN **25**	GSH	48364	48364	38216010	47946	11659875
N=2	BCN **25**	CuAAC conditions	48364	48364	5588428	47950	1176087
N=2	BCN **25**	pH 7.2					
N=2	Tetrazine **26**	TCEP	48493	48493	53473526	48076	14436135
N=2	Tetrazine **26**	GSH	48493	48493	63968779	-	0
N=2	Tetrazine **26**	CuAAC conditions	48493	48495	419057	48071	1622140
N=2	Tetrazine **26**	pH 7.2	48493	48493	75971297	-	0

Table S5. *Summary of LCMS data for on-protein stability assessment. Non-clicked species are not observed for some samples, which are denotated in the table as -. BCN in pH 7.2 n=2 not reported due to low intensity spectrum.*

Exemplar applications of click chemistry in which decision trees are used to select optimal click handles

Example Application 1: Production of dually functionalised bispecific antibodies

Context: Maruani and coworkers described a plug and play approach to produce dually functionalised chemically linked bispecific antibodies (Scheme S1).[33a] The key requirements for the two click reactions are to be stable to reducing and basic conjugation conditions and also be orthogonal to each other.

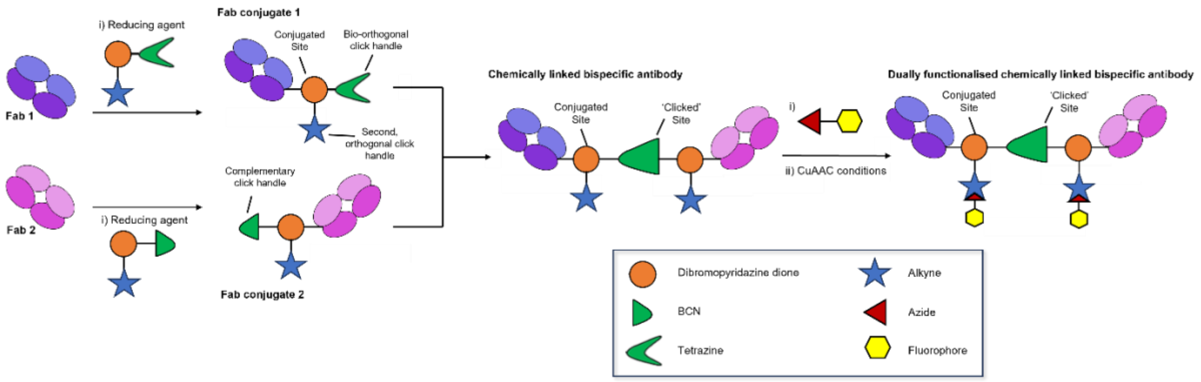

Scheme S1. *Schematic of the plug and play procedure used by Maruani and coworkers to produce chemically linked bispecific antibodies using click chemistry.*[40]

Recommendations, using this guide:

- From 'Decision Trees and Recommendations':
  1. Basic buffer used → Avoid Michael additions

2. If CuAAC is selected, use as the final step in the process.
3. Carry out a buffer exchange prior to the conjugation reaction.

- Select click reactions based on reaction kinetics and orthogonality of the reactions to each other (Fig. 1) → IEDDA and CuAAC
- Using 'Decision Trees' (Fig. 6A)
  1. Steric influence not relevant → no
  2. Thiols not present → no
  3. Previously recommended buffer exchange, phosphine reducing agent not present → no

  → TCO, norbornene or BCN appropriate
- Using 'Decision Trees' (Fig. 6B)
  1. Basic reaction conditions present → yes

  → Tetrazine-Me appropriate

Results: Following the questions and decision trees provided in the main text, for this example we would recommend using the IEDDA reaction followed by the CuAAC reaction. Suggested IEDDA dienophiles are TCO, norbornene or BCN and tetrazine-Me is suggested as the IEDDA diene.

Discussion: The click reaction classes and associated click handles which are recommended using the above process correlate well with those used by Maruani and coworkers. In addition, two further IEDDA dienophiles which may be suitable for the process are identified.

Example Application 2: Labelling of Proteins on Mammalian Cell Surfaces

Context: Lemke *et al* described a method using click chemistry to label proteins on live cell surfaces.[41] The key requirements for the click reaction are that the process is not toxic to cells and the click handles are commercially available within noncanonical amino acids.

Recommendations, using this guide:

- From 'Decision Trees and Recommendations':
  1. Presence of thiols in cell media → Avoid Michael additions
  2. Copper toxic to cells → Avoid CuAAC
  3. Not relevant
- Select click reactions based on no cell toxicity and commercial availability of click handles →IEDDA, SPAAC and SPANC suitable
- Additional note, ketone condensations not appropriate due to inefficiency at neutral pH.
- Using 'Decision Trees' (Fig. 6A)
  1. Steric influence not relevant → no
  2. Thiols present → yes

  → TCO appropriate (norbornene noncanonical amino acid not commercially available). BCN may be appropriate, depending on concentration of thiols present in cell media.
- Using 'Decision Trees' (Fig. 6B)
  1. Basic reaction conditions present → no
  2. Reaction kinetics more important

  → Tetrazine-H appropriate, Tetrazine-Me may also be appropriate
- Using 'Decision Trees' (Fig. 6C)
  1. Thiols present → yes

Results: In terms of the IEDDA diene click partner, we suggest that any tetrazine containing dye would be appropriate. If SPAAC is selected, we recommend using BCN vs DBCO due to its increased stability to thiols.

Discussion: The two click reactions which are recommended using our procedure align with the two click reactions used by Lemke *et al*. TCO was recommended for use in an IEDDA reaction in this application, however it was also suggested that BCN could be appropriate. The authors use both of these click handles, demonstrating that the suggested click handles within this guide can be used successfully within this application.

Example Application 3: Activity Based Protein Profiling (ABPP)

Context: Activity based chemical probes can be used to interrogate enzyme activities to understand their role in physiological processes.[33b] The key requirements for the click reaction are that the click handles are sterically inert, so that the proteome can be profiled in a sterically unbiased manner.

Recommendations, using this guide:

- From 'Decision Trees and Recommendations':
  1. Presence of thiols in cell media → Avoid Michael additions
  2. Not relevant
  3. Not relevant
- Select click reactions based on sterics of click handles →IEDDA and CuAAC suitable
- Using 'Decision Trees' (Fig. 6A)
  1. Steric influence relevant → yes

  → Cyclopropene appropriate
- Using 'Decision Trees' (Fig. 6B)
  1. Basic reaction conditions present → no
  2. Reaction kinetics more important

  → Tetrazine-H appropriate, Tetrazine-Me may also be appropriate

Results: Click reaction used following cell lysis, so CuAAC appropriate. IEDDA using a cyclopropene 'mini-tag' would also be suitable.

Discussion: The click reaction used within this application was an azide alkyne cycloaddition, however using rhodamine alkyne reagents as an alternative to the CuAAC reaction. In addition, we also recommended an alternative set of click handles from a different click reaction class that may be appropriate for the application.

Copies of NMR spectra

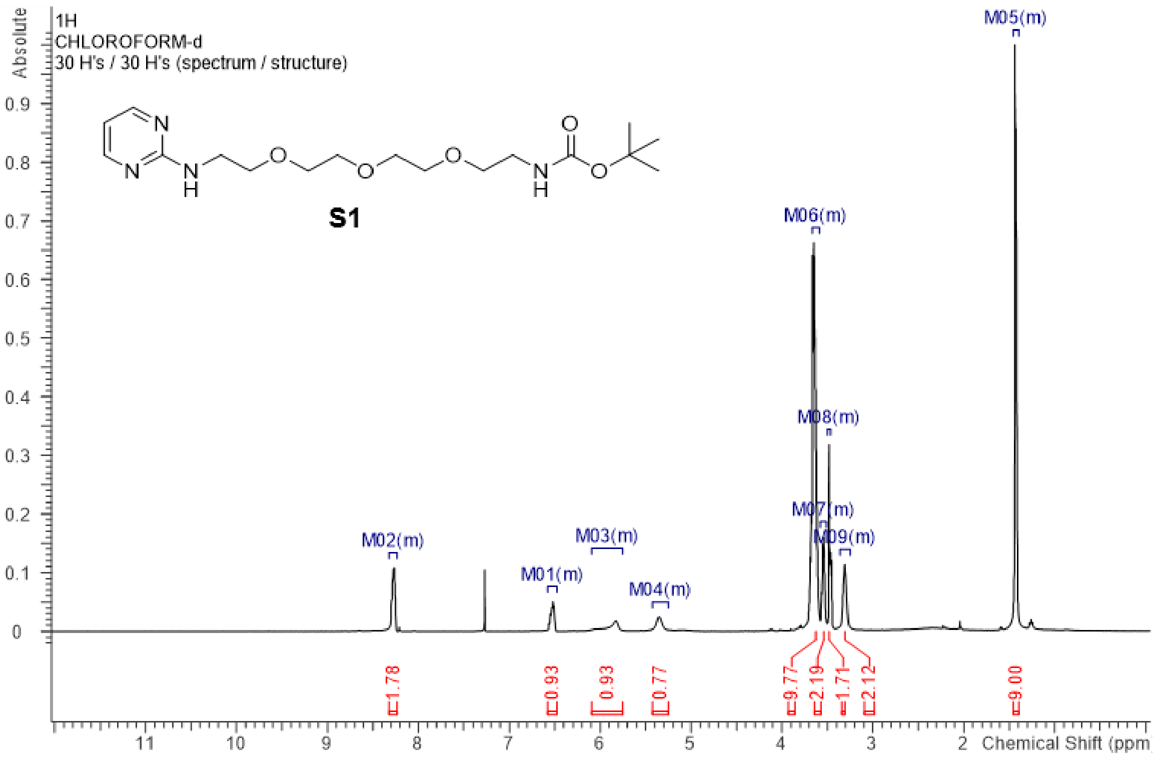

Figure S45. *¹H NMR spectrum of compound* ***S1***

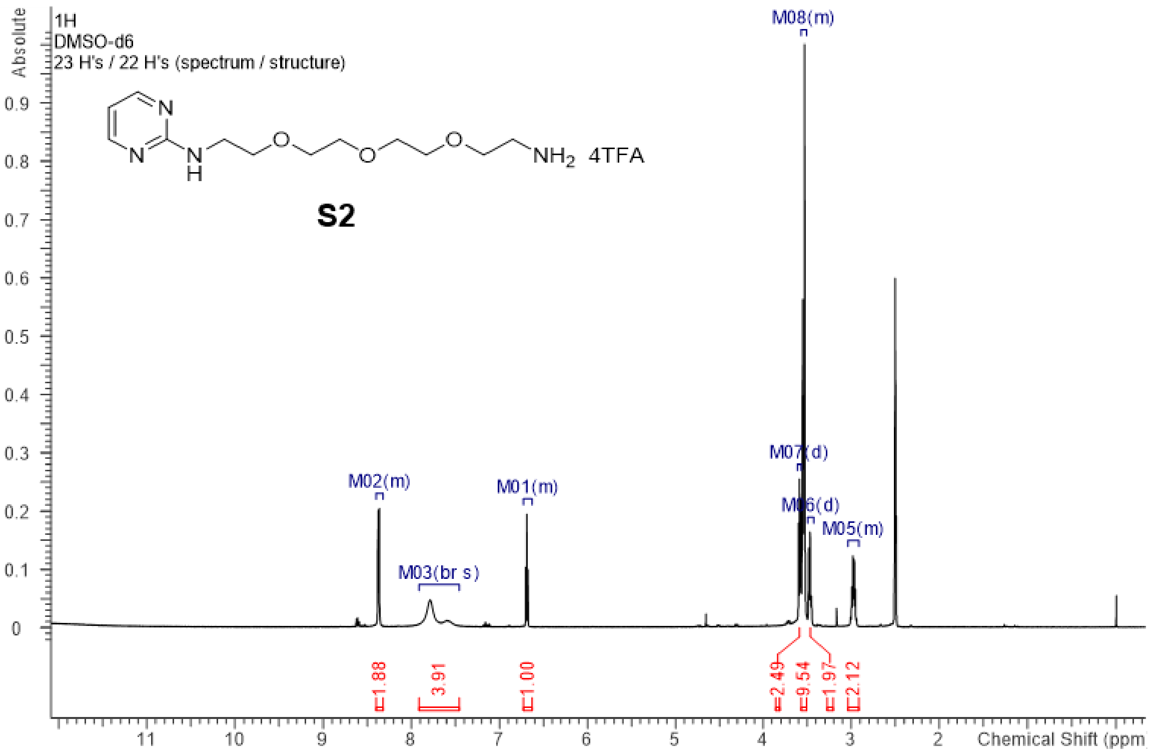

Figure S46. *¹H NMR spectrum of compound* ***S2***

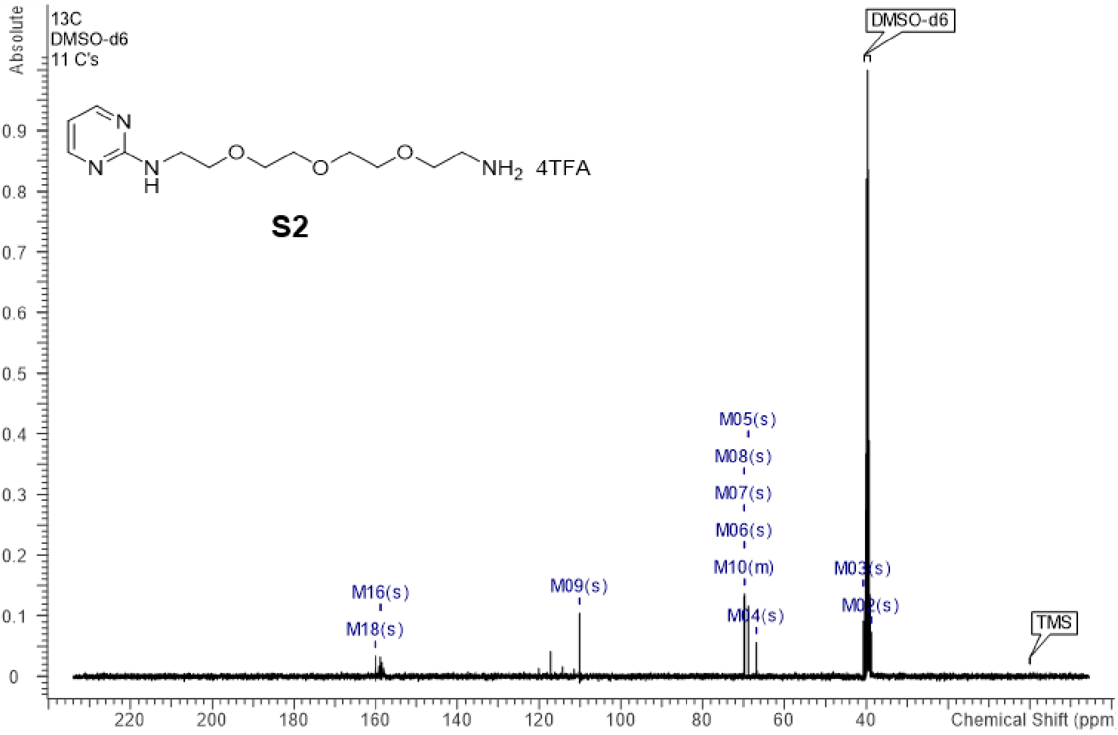

Figure S47. *^{13}C NMR spectrum of compound* ***S2***

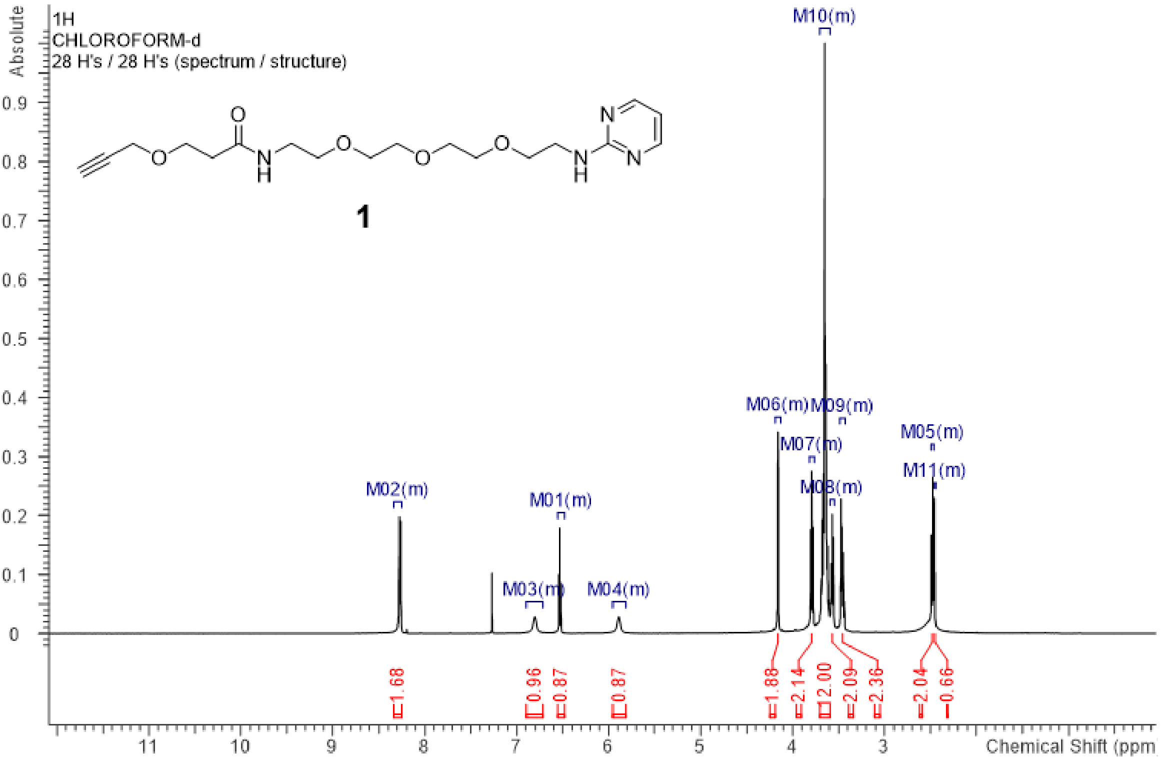

Figure S48. *^{1}H NMR spectrum of compound* ***1***

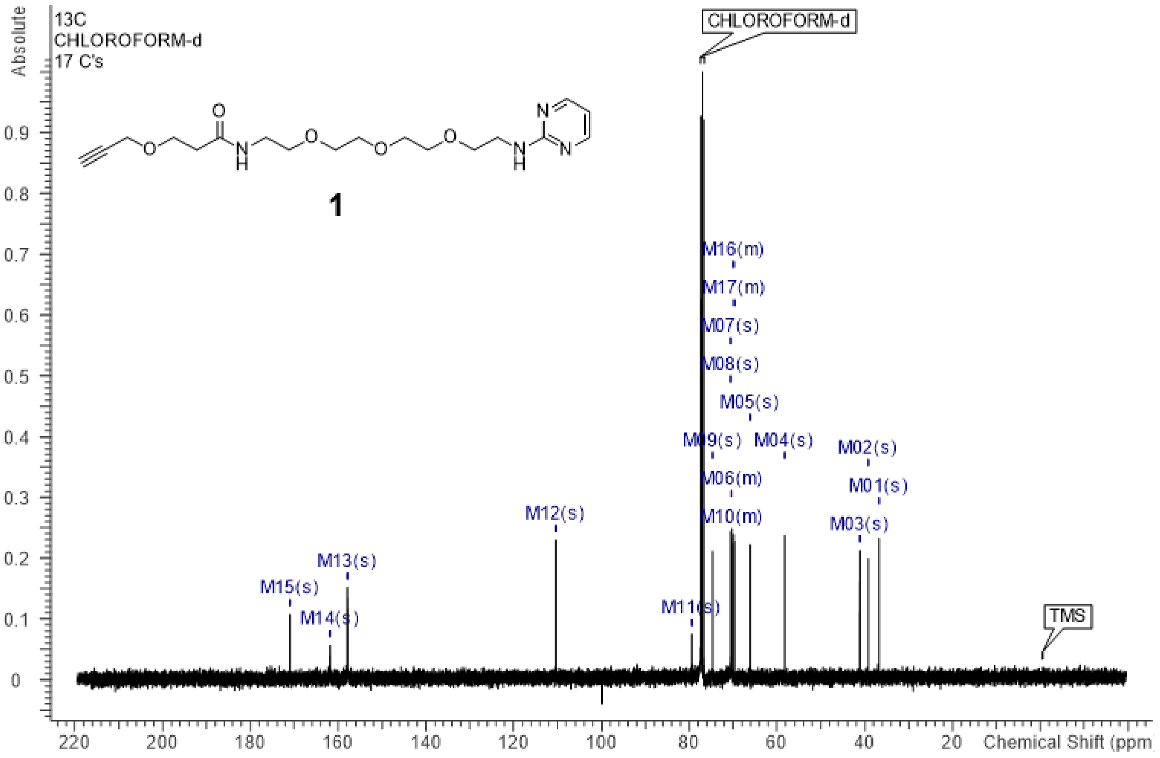

Figure S49. *^{13}C NMR spectrum of compound **1***

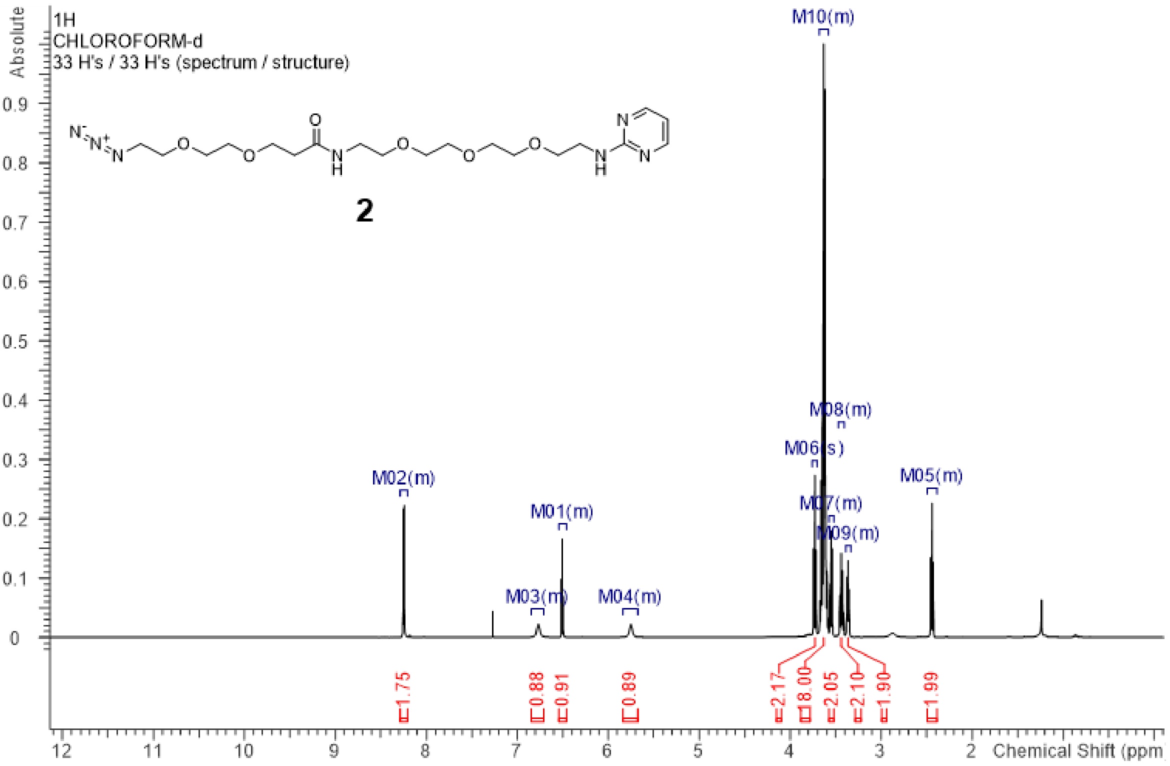

Figure S50. *^{1}H NMR spectrum of compound **2***

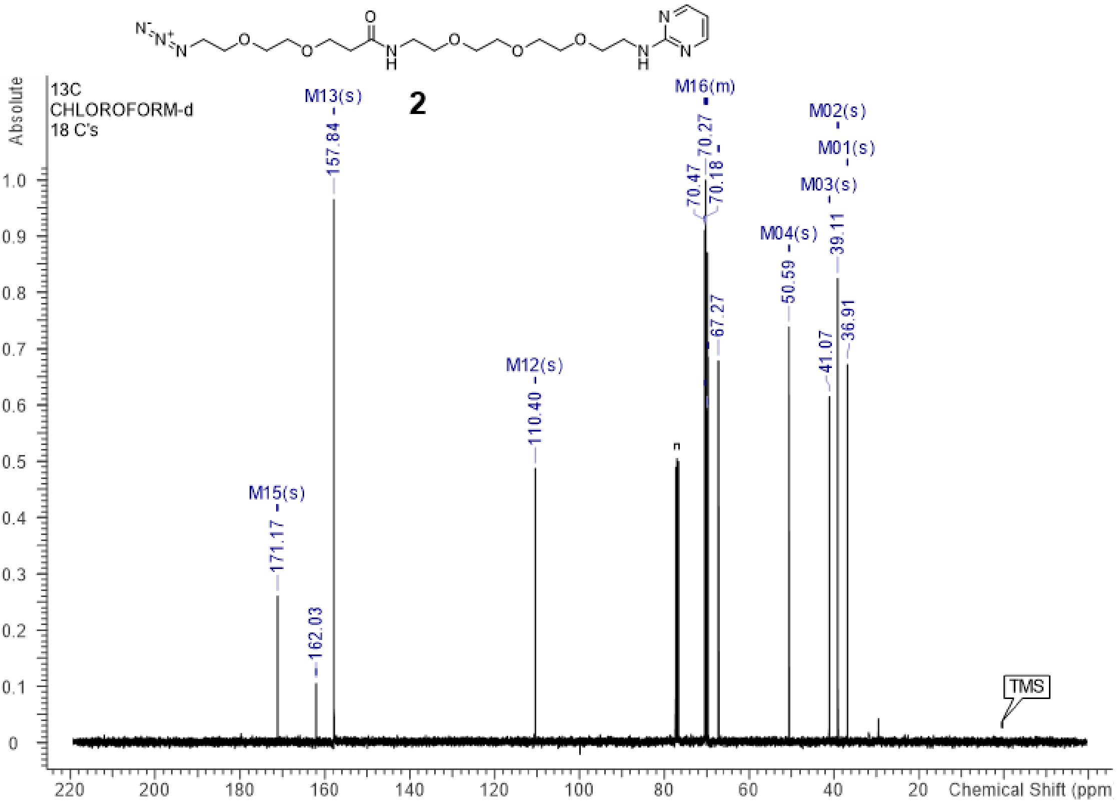

Figure S51. *^{13}C NMR spectrum of compound **2***

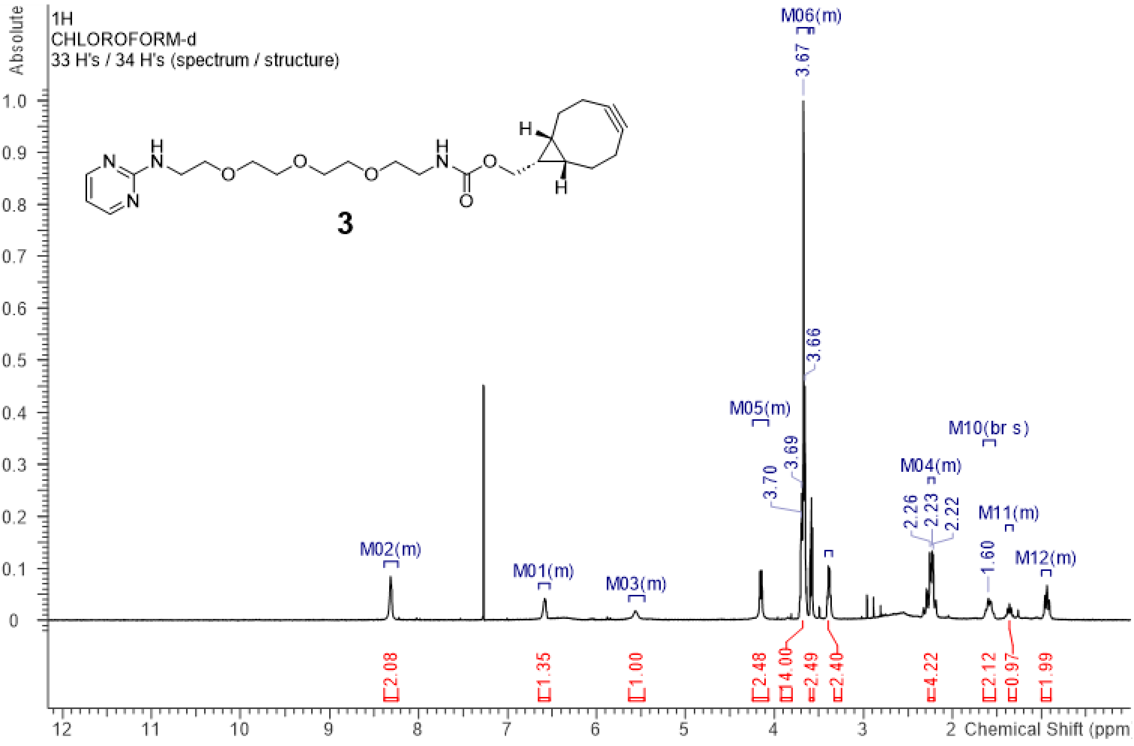

Figure S52. *^{1}H NMR spectrum of compound **3***

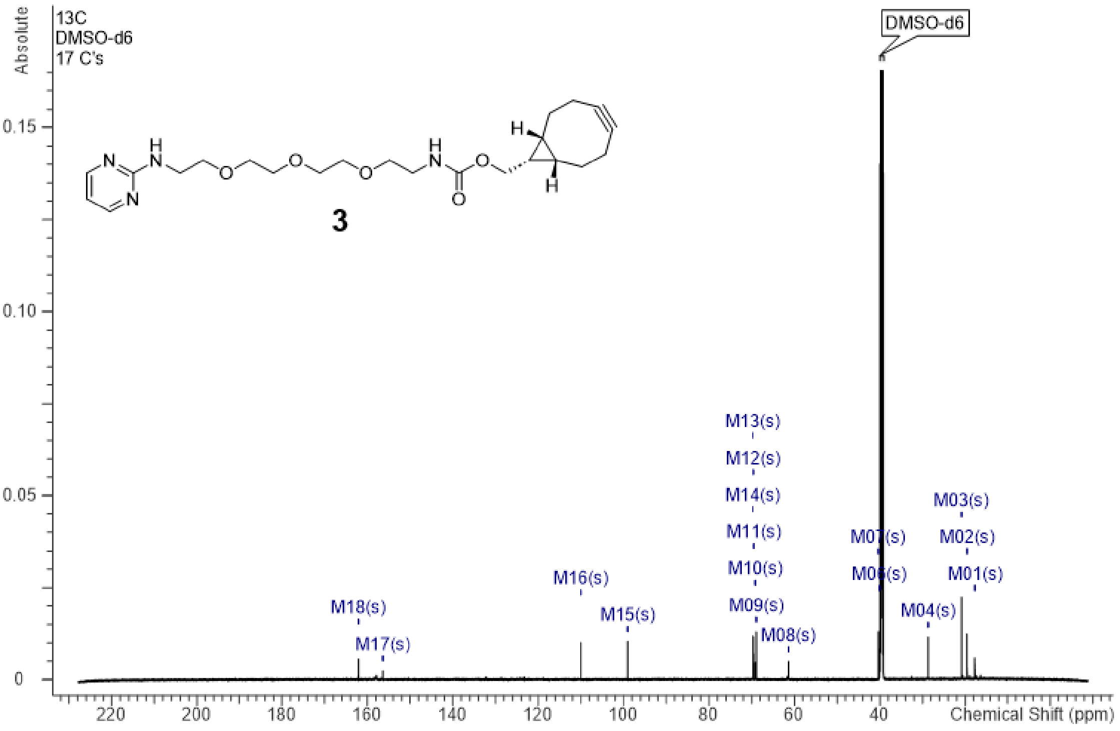

Figure S53. *^{13}C NMR spectrum of compound **3***

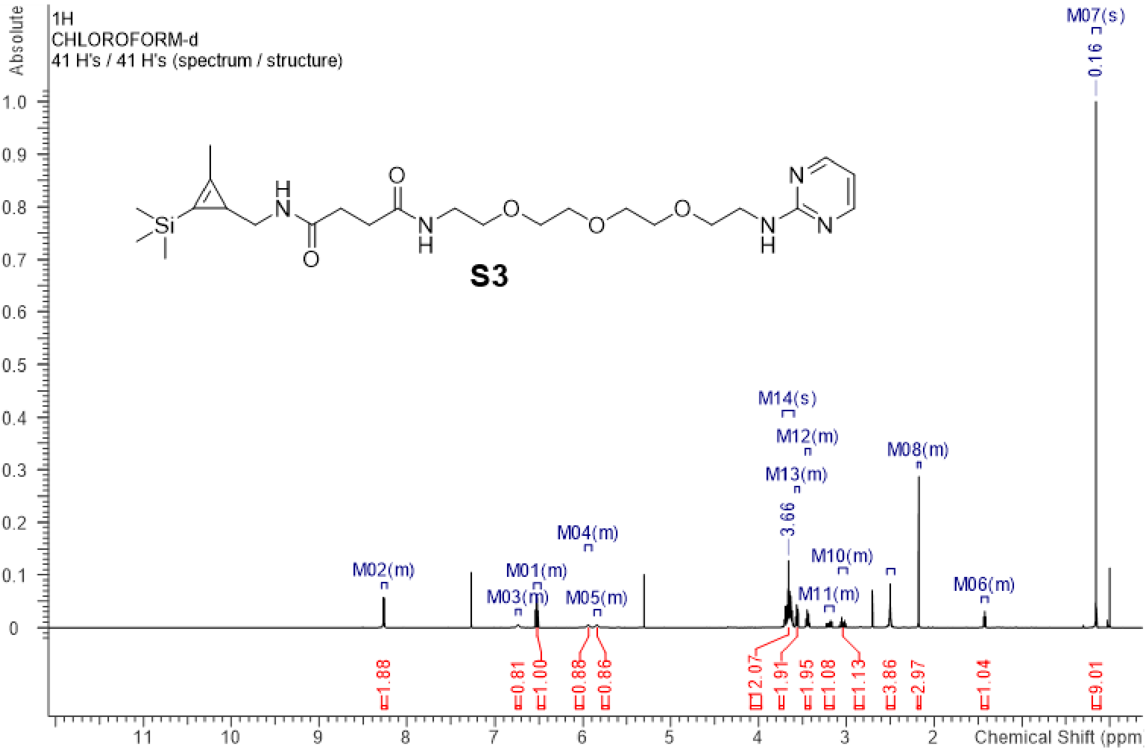

Figure S54. *^{1}H NMR spectrum of compound **S3***

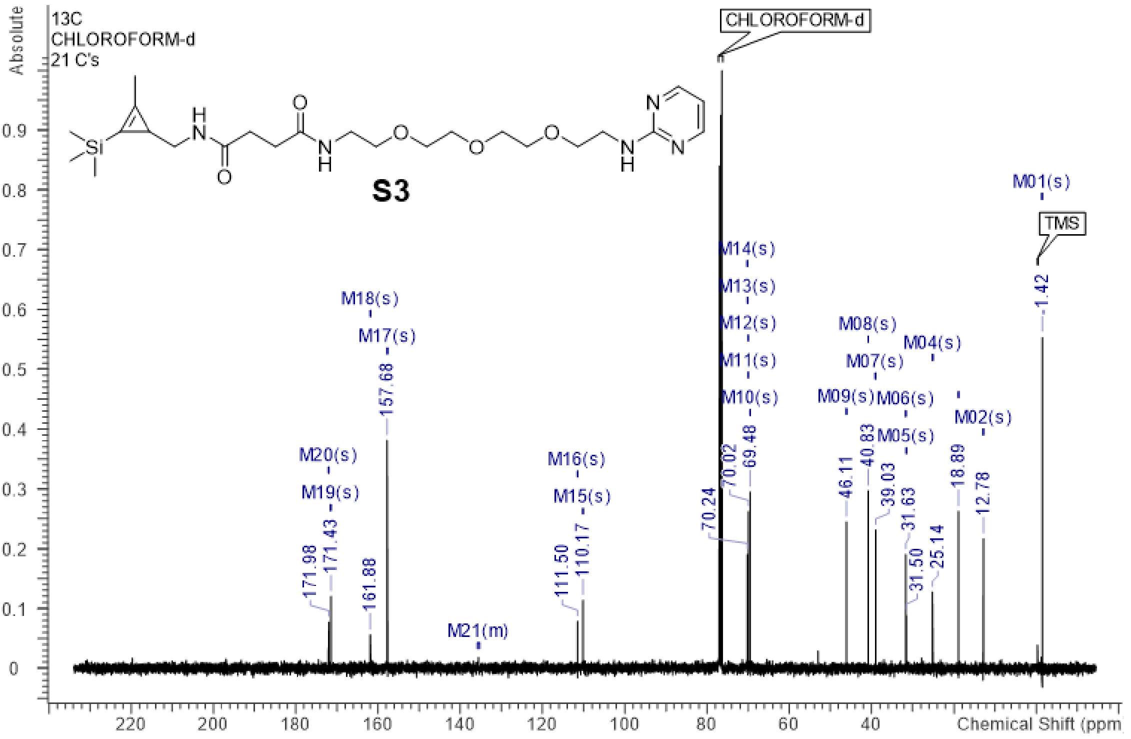

Figure S55. ^{13}C *NMR spectrum of compound* ***S3***

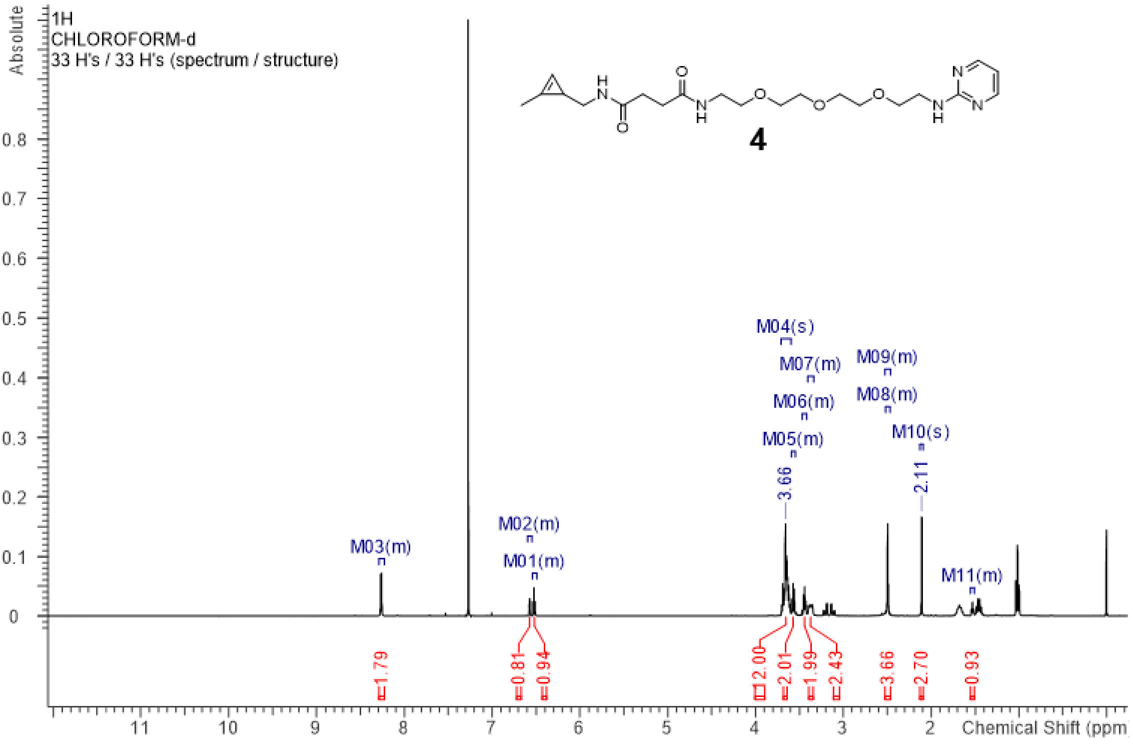

Figure S56. ^{1}H *NMR spectrum of compound* ***4***

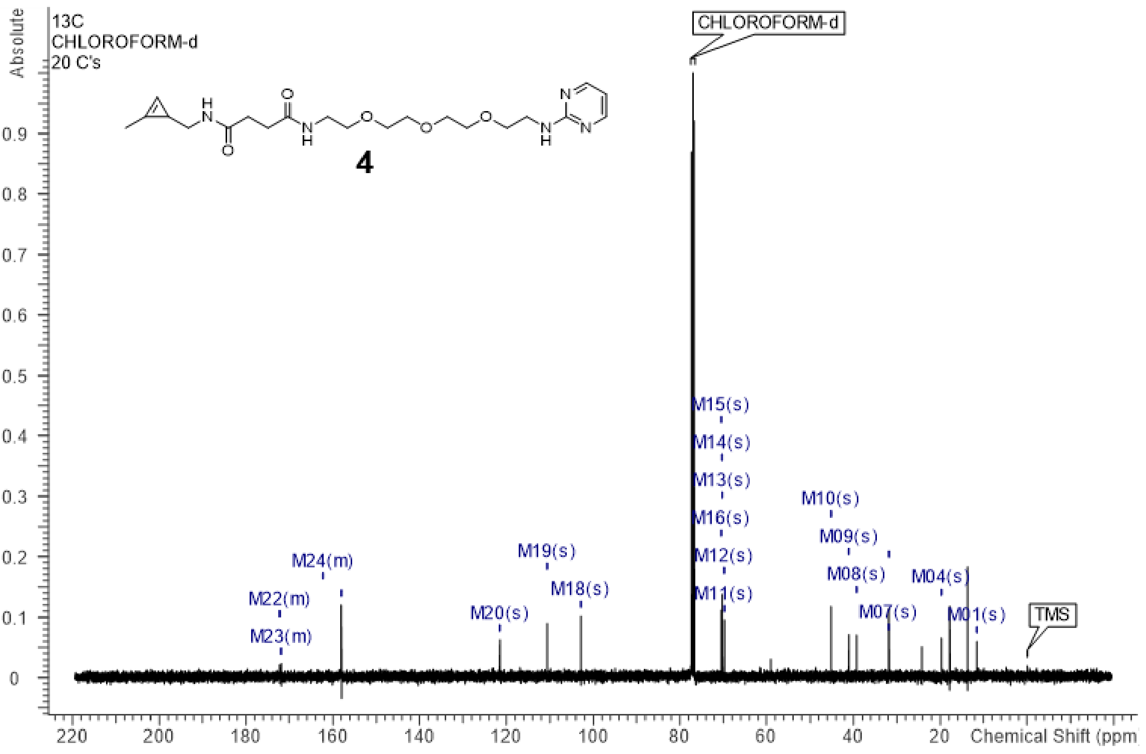

Figure S57. ^{13}C NMR spectrum of compound 4

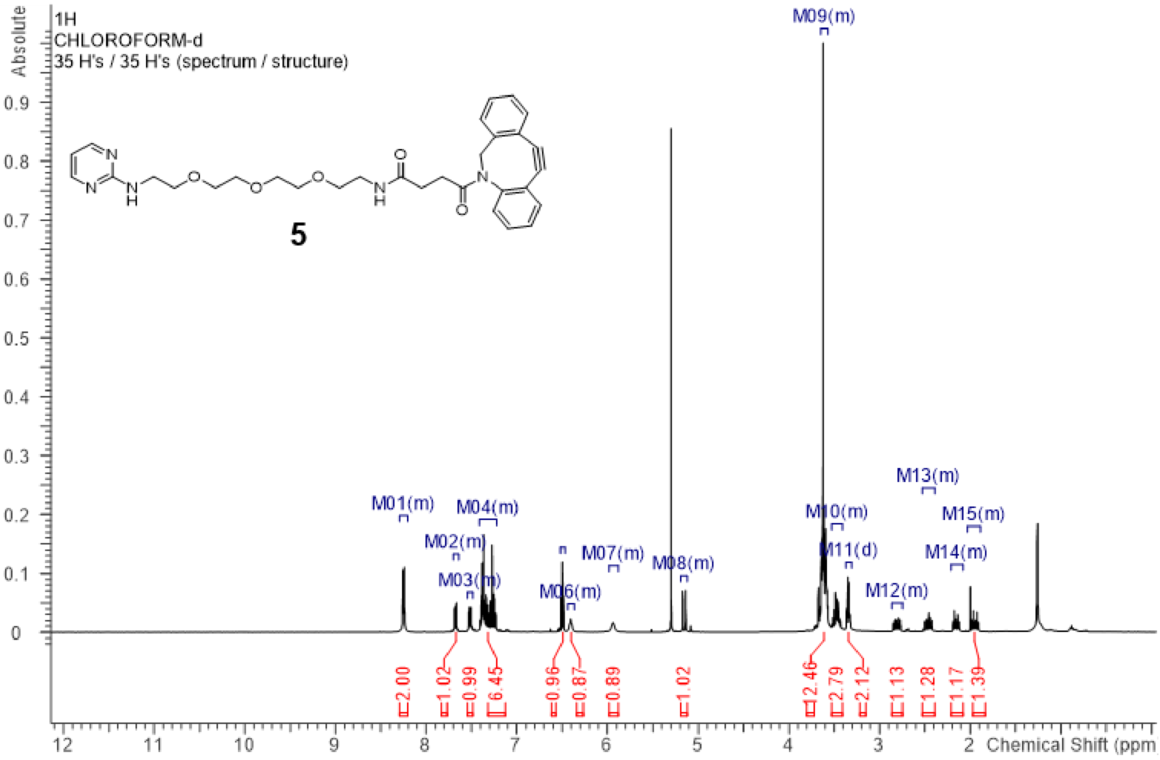

Figure S58. ^{1}H NMR spectrum of compound 5

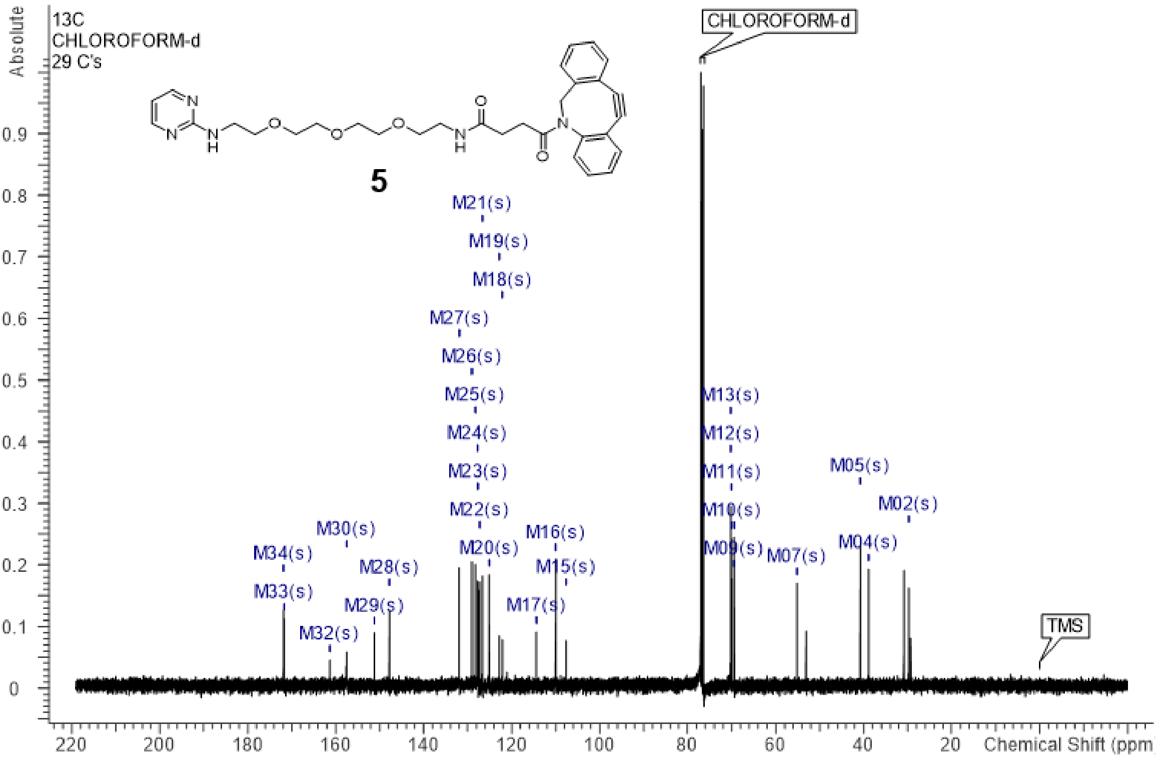

Figure S59. *^{13}C NMR spectrum of compound* ***5***

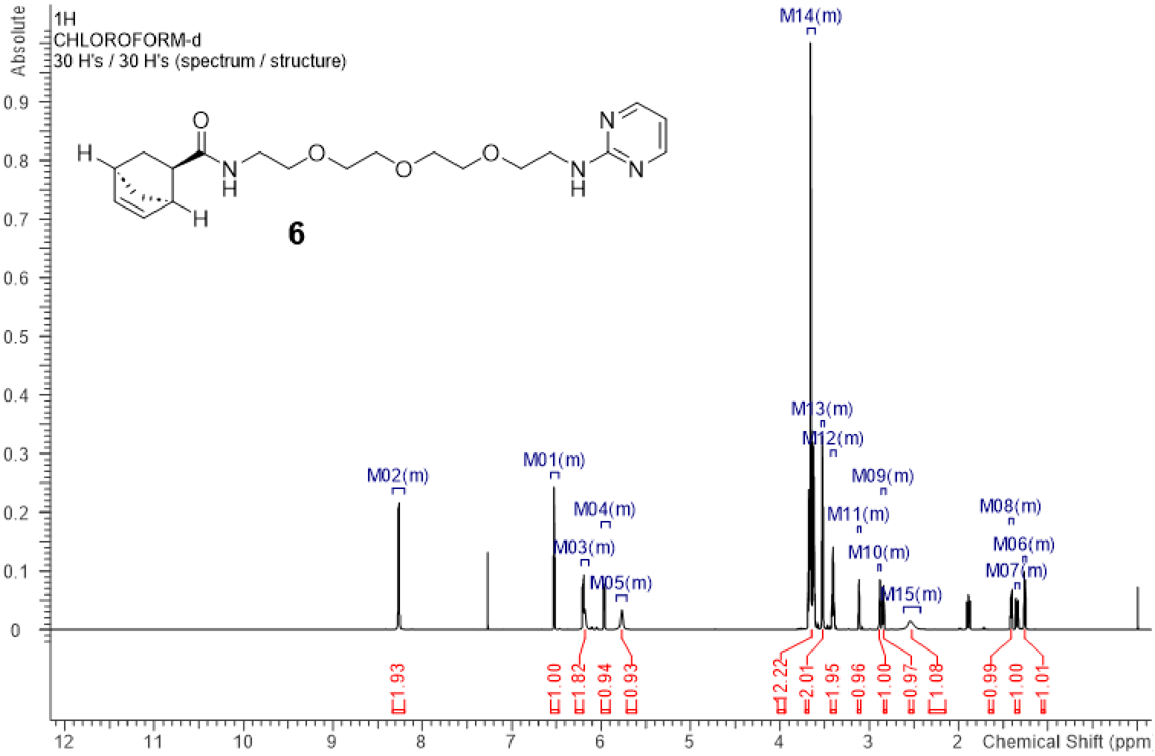

Figure S60. *^{1}H NMR spectrum of compound* ***6***

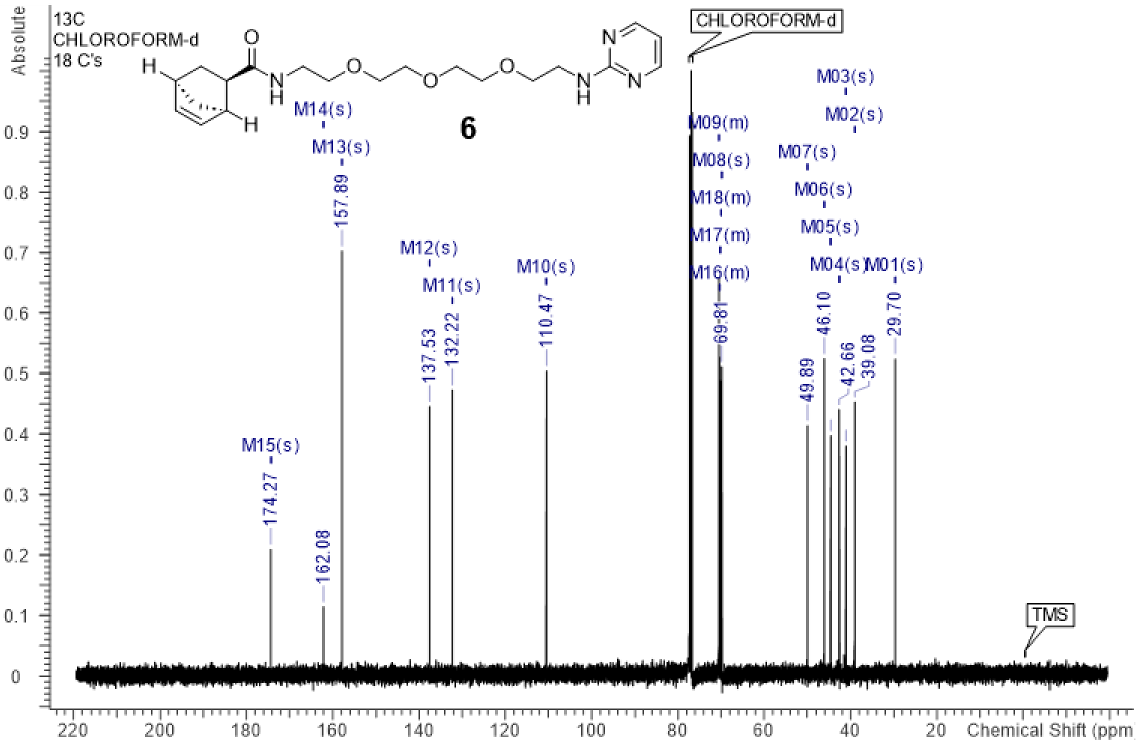

Figure ***S61.*** *^{13}C NMR spectrum of compound* ***6***

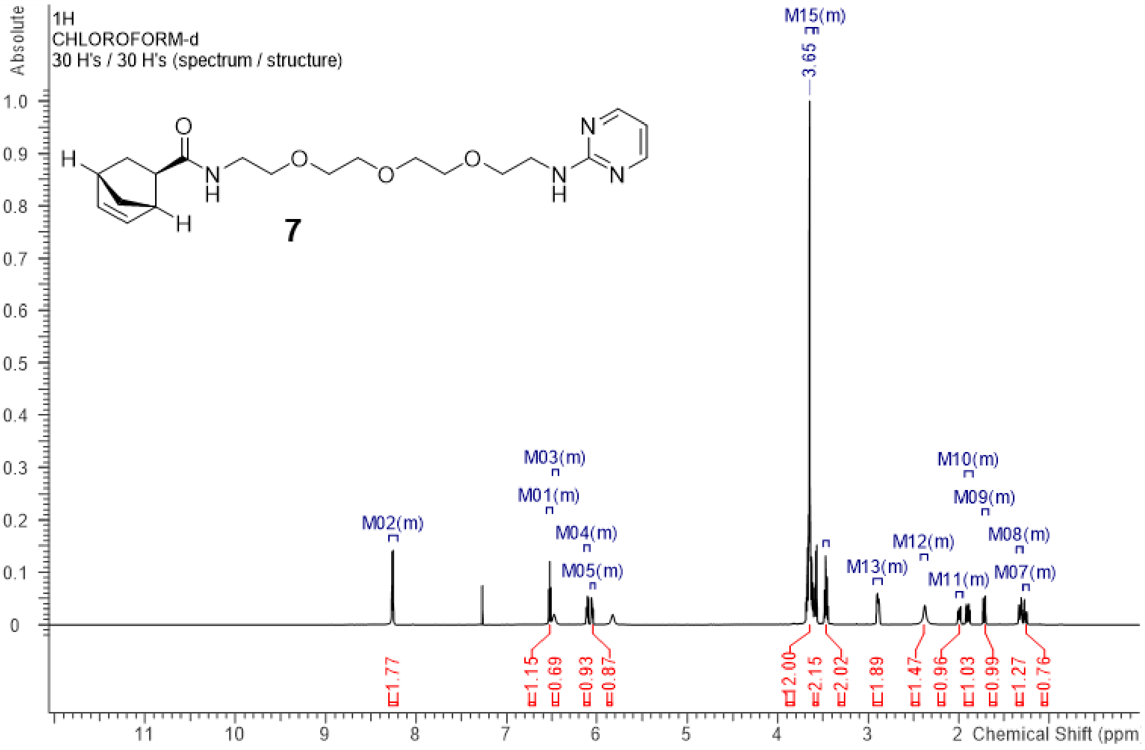

Figure ***S62.*** *^{1}H NMR spectrum of compound 7*

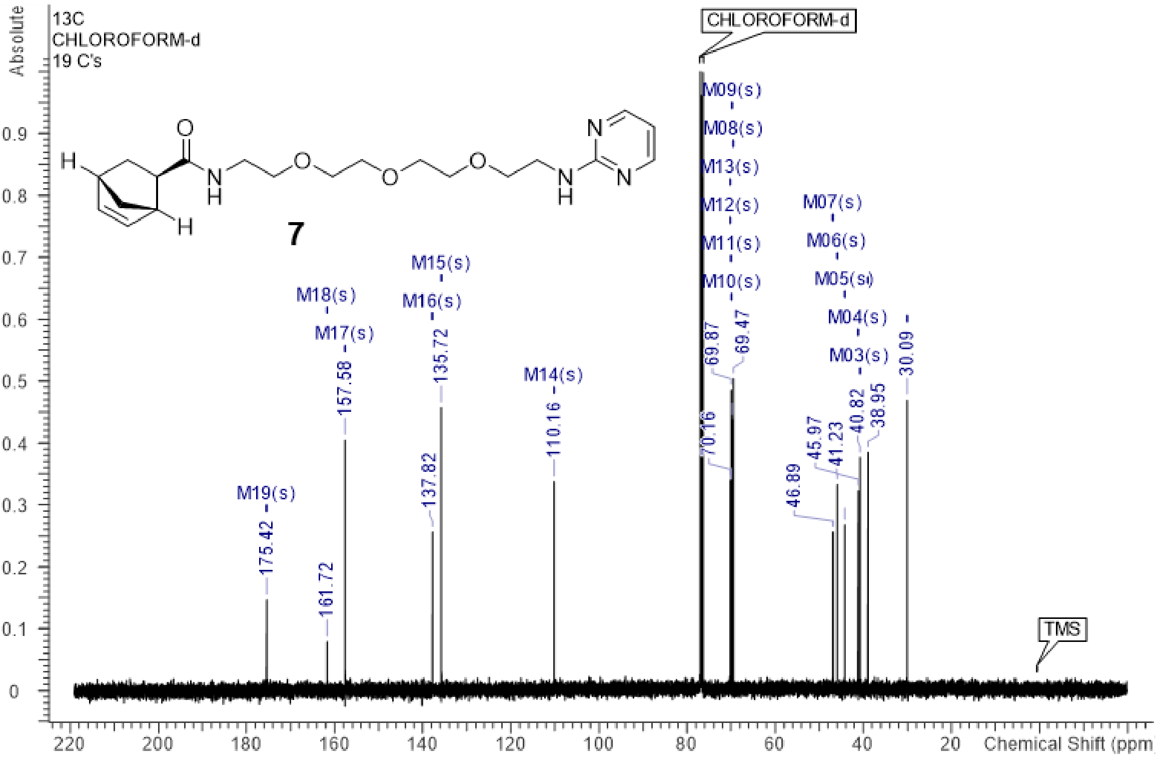

Figure S63. *^{13}C NMR spectrum of compound 7*

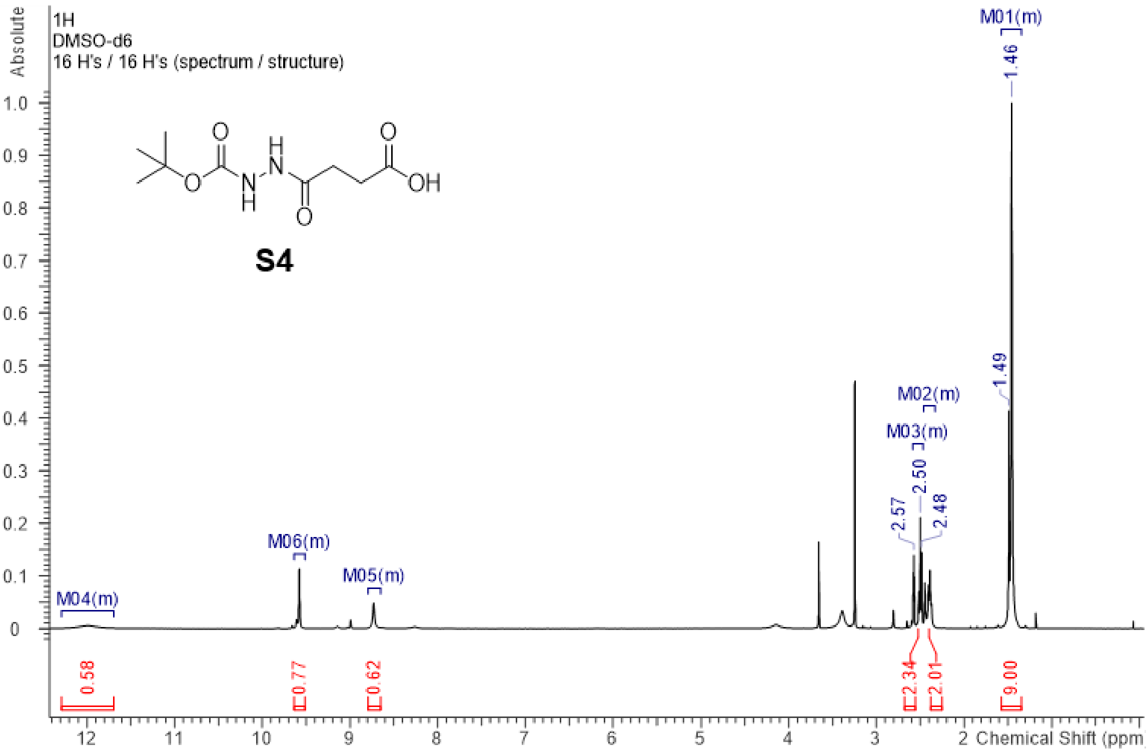

Figure S64. *^{1}H NMR spectrum of compound **S4***

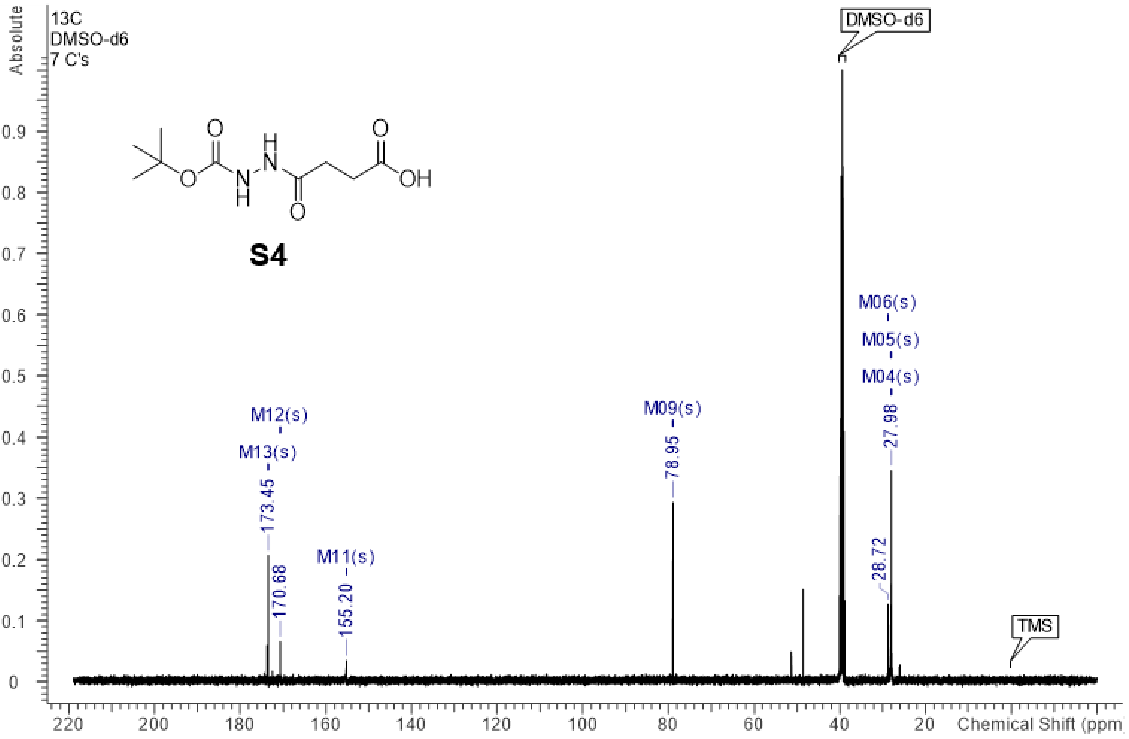

Figure S65. ^{13}C NMR spectrum of compound ***S4***

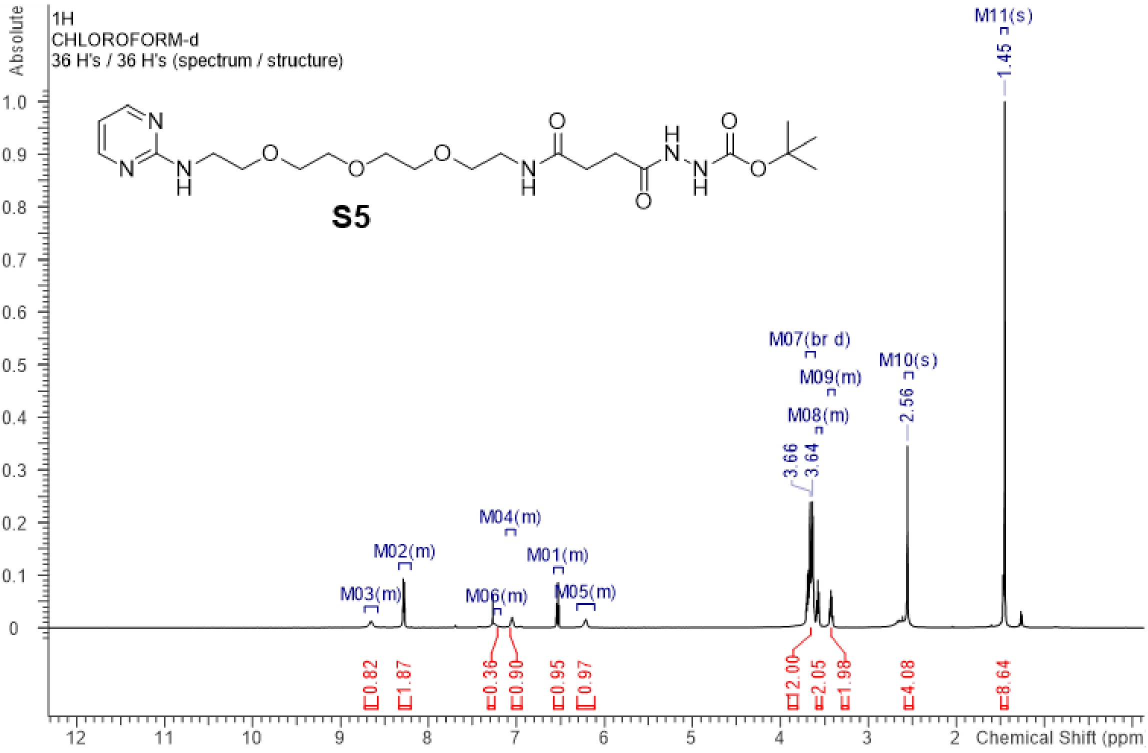

Figure S66. ^{1}H NMR spectrum of compound ***S5***

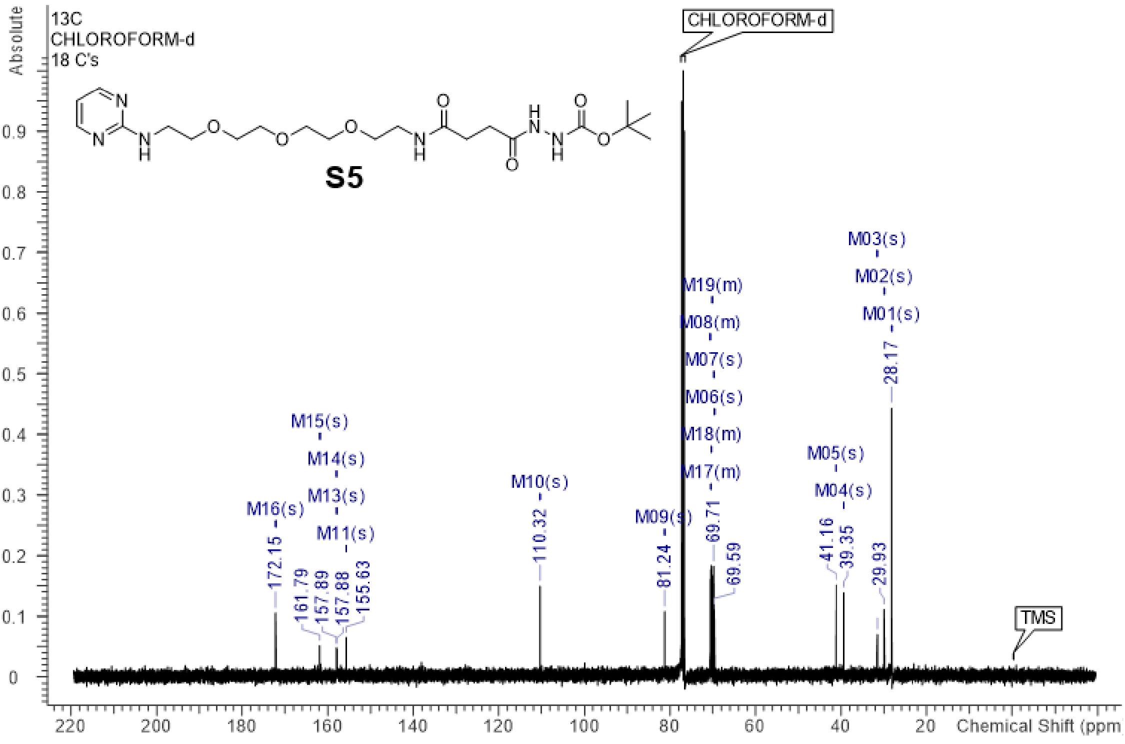

Figure S67. ^{13}C NMR spectrum of compound ***S5***

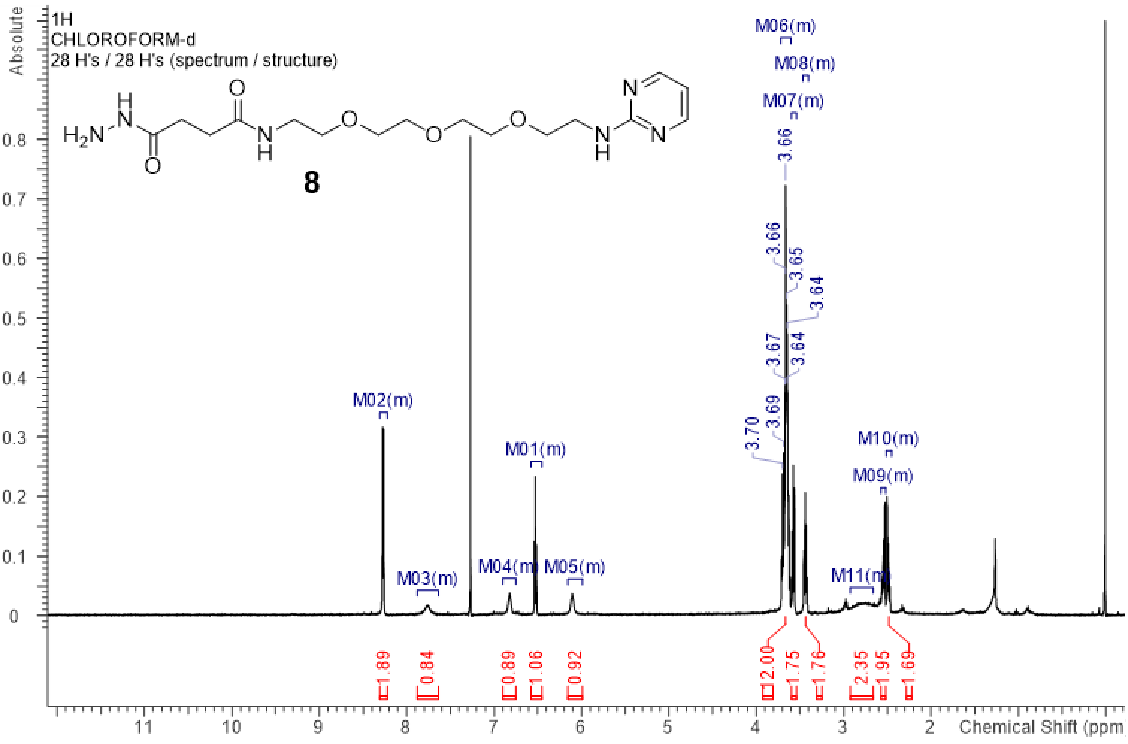

Figure S68. ^{1}H NMR spectrum of compound ***8***

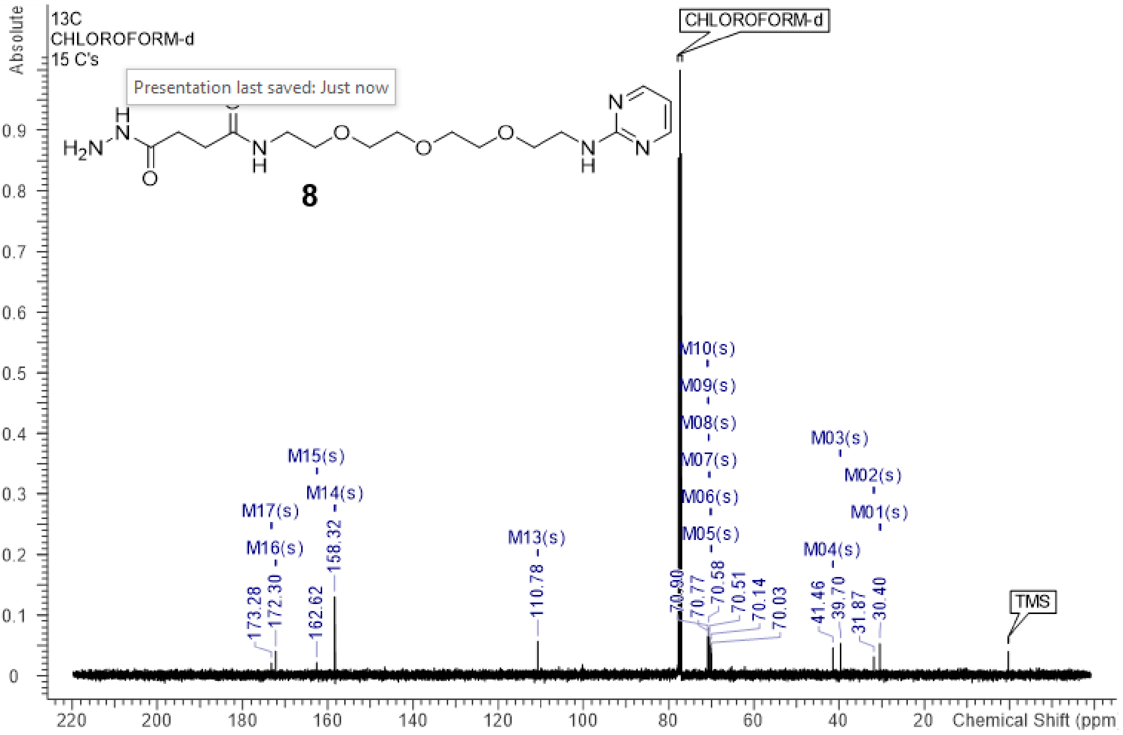

Figure S69. *^{13}C NMR spectrum of compound **8***

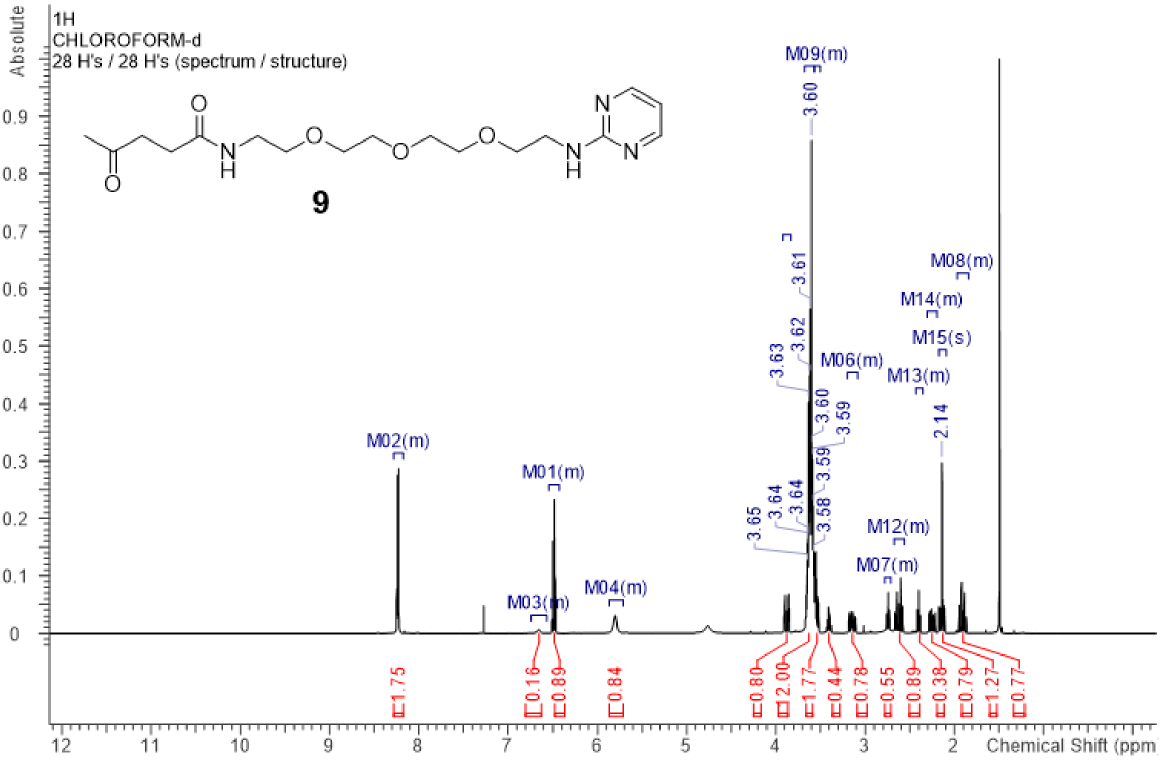

Figure S70. *^{1}H NMR spectrum of compound **9***

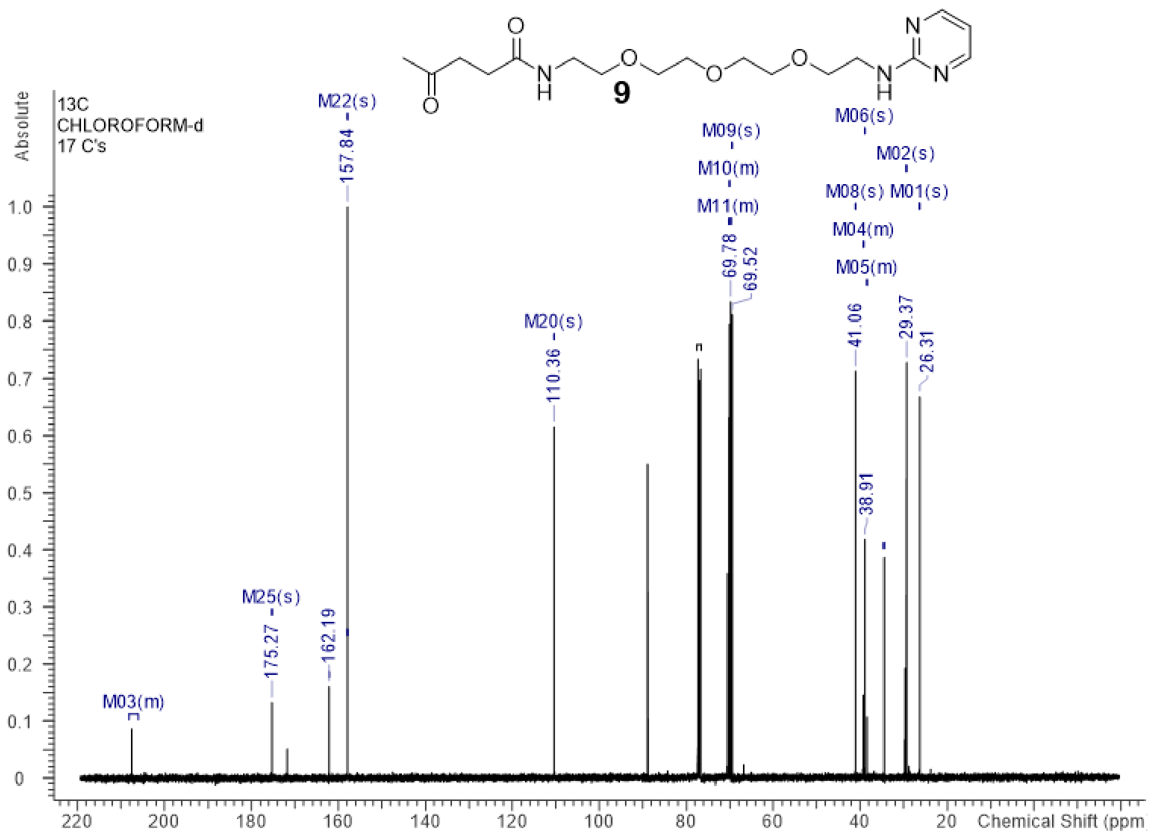

Figure S71. *^{13}C NMR spectrum of compound* ***9***

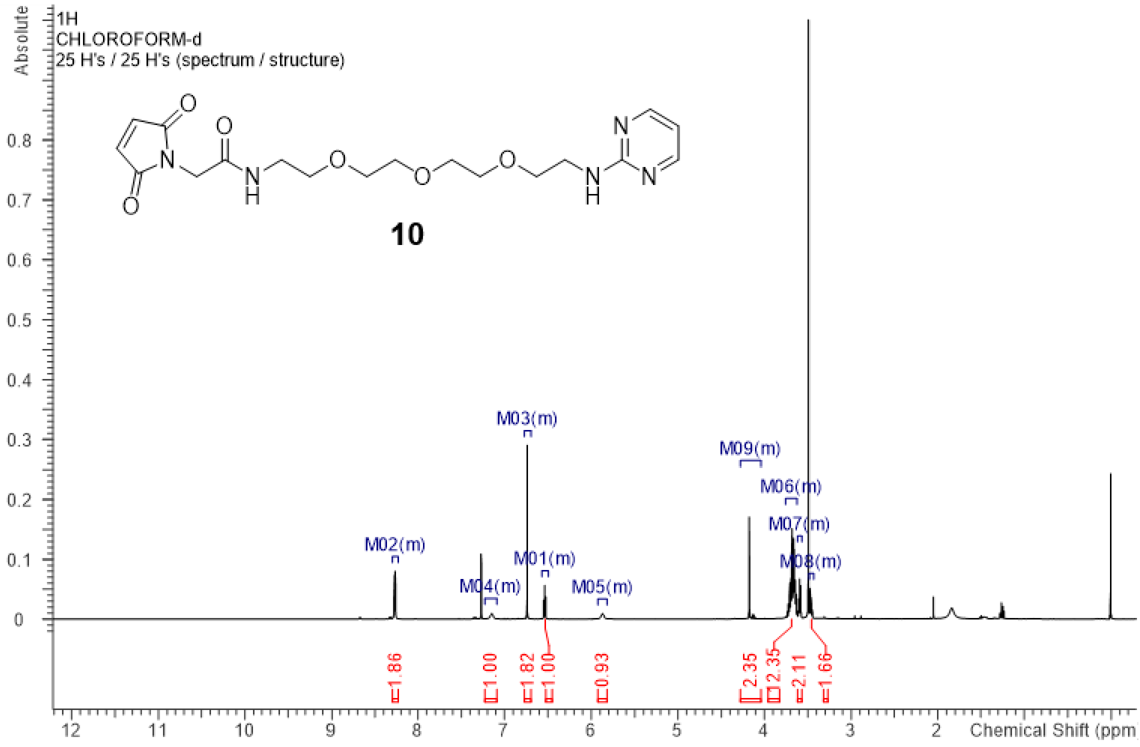

Figure S72. *^{1}H NMR spectrum of compound* ***10***

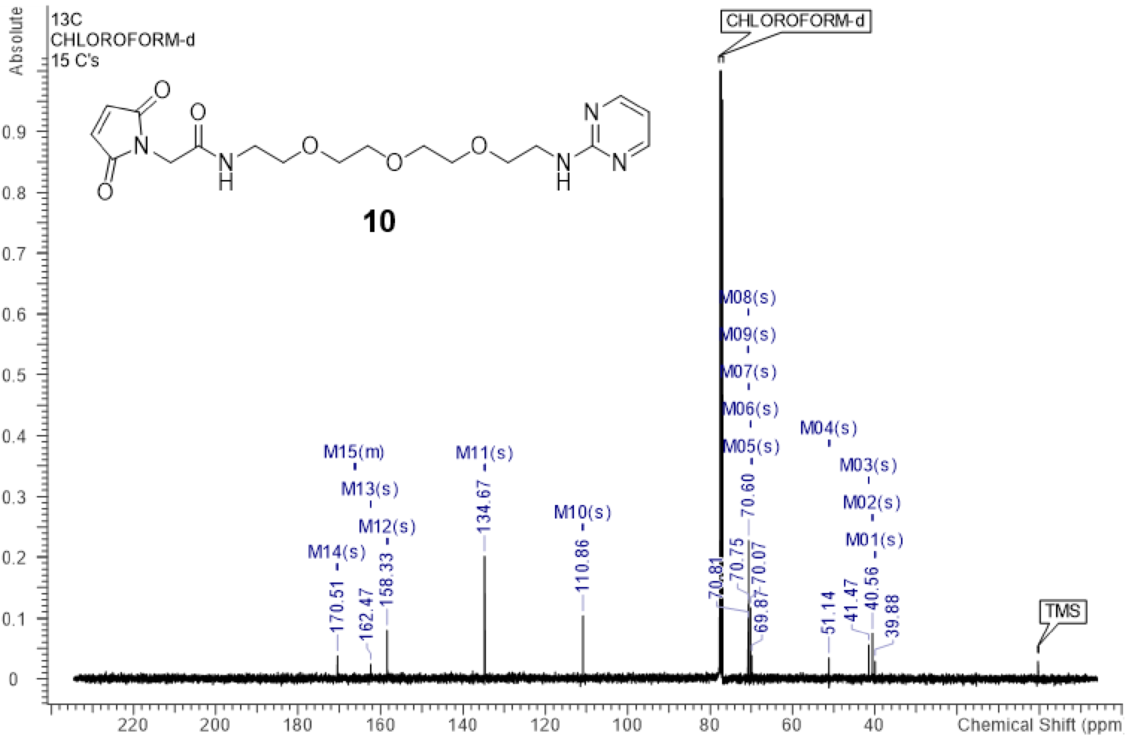

Figure S73. *^{13}C NMR spectrum of compound* ***10***

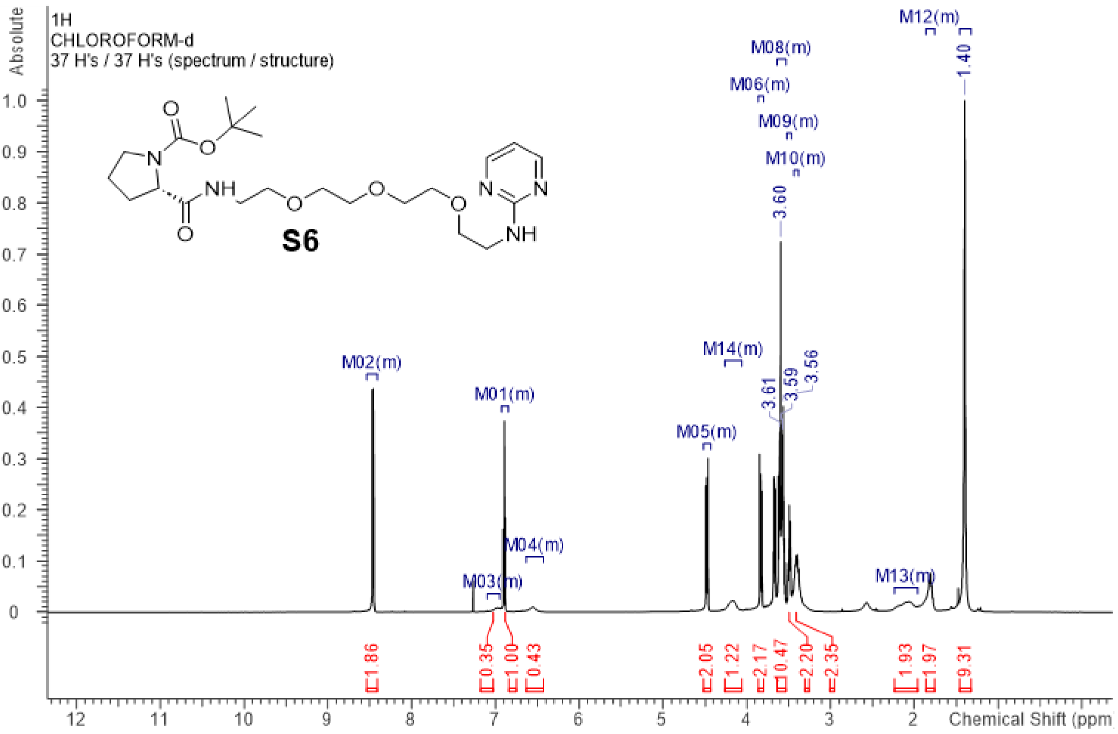

Figure S74. *^{1}H NMR spectrum of compound* ***S6***

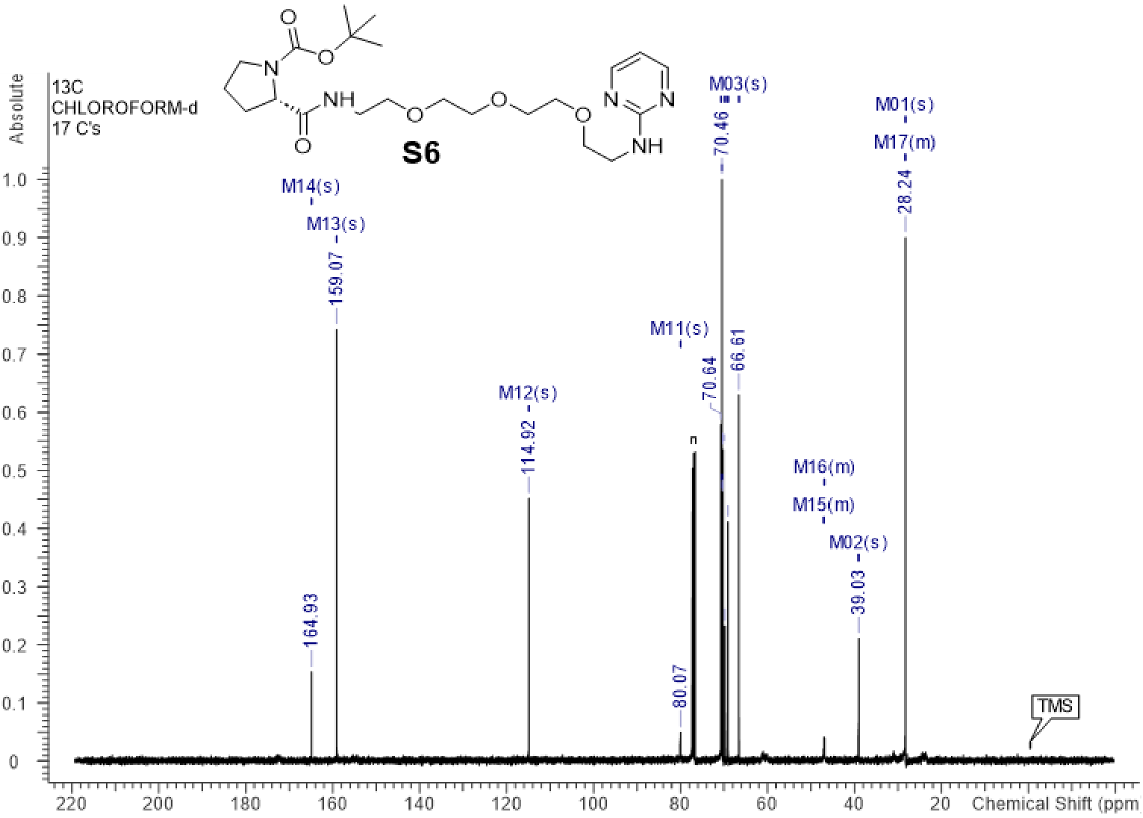

Figure S75. ^{13}C NMR spectrum of compound ***S6***

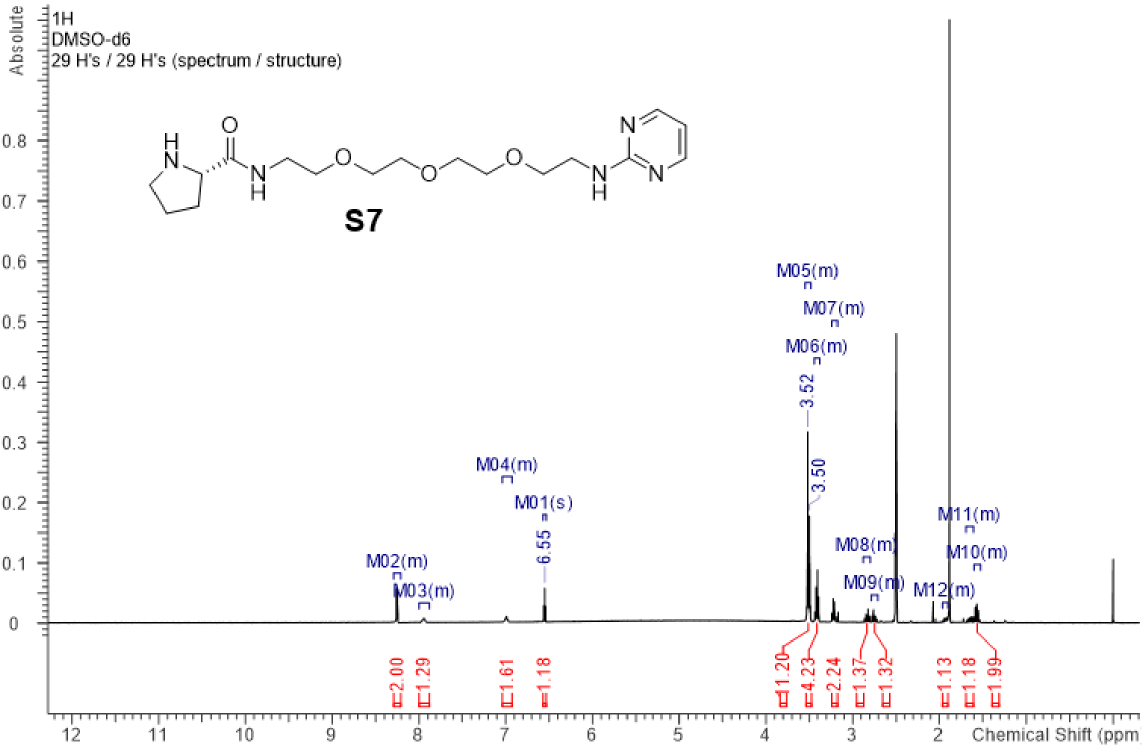

Figure S76. ^{1}H NMR spectrum of compound ***S7***

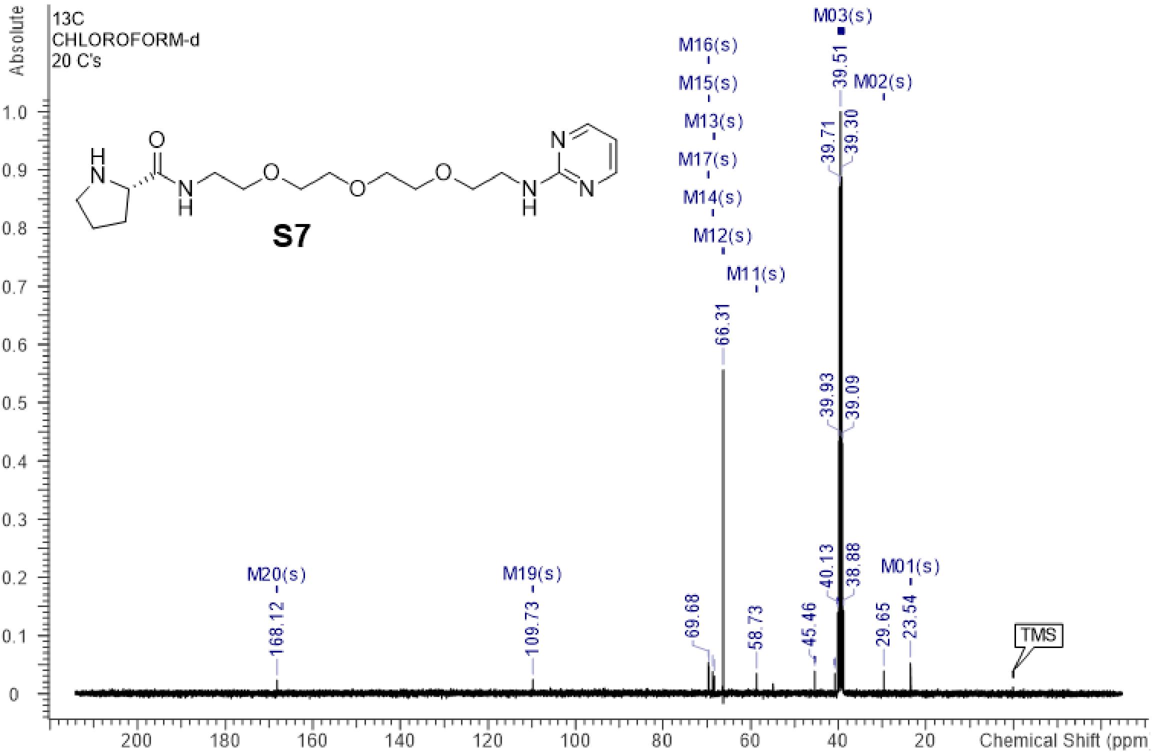

Figure S77. ^{13}C NMR spectrum of compound ***S7***

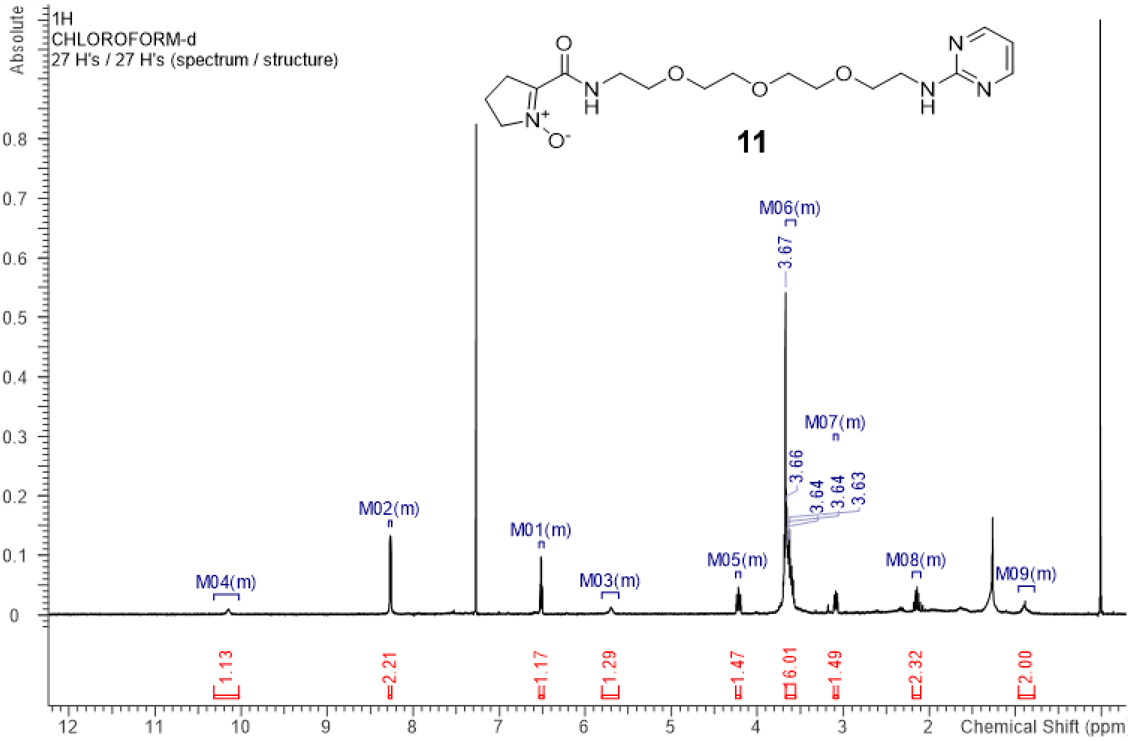

Figure S78. ^{1}H NMR spectrum of compound ***11***

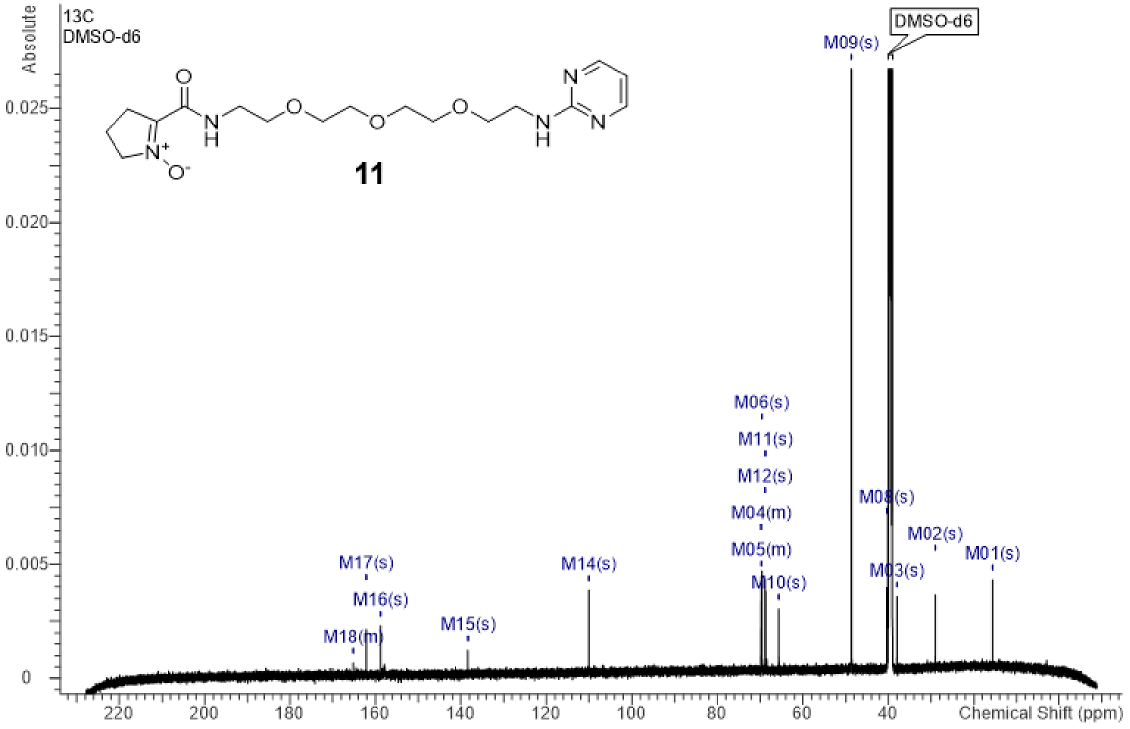

Figure S79. ^{13}C NMR spectrum of compound **11**

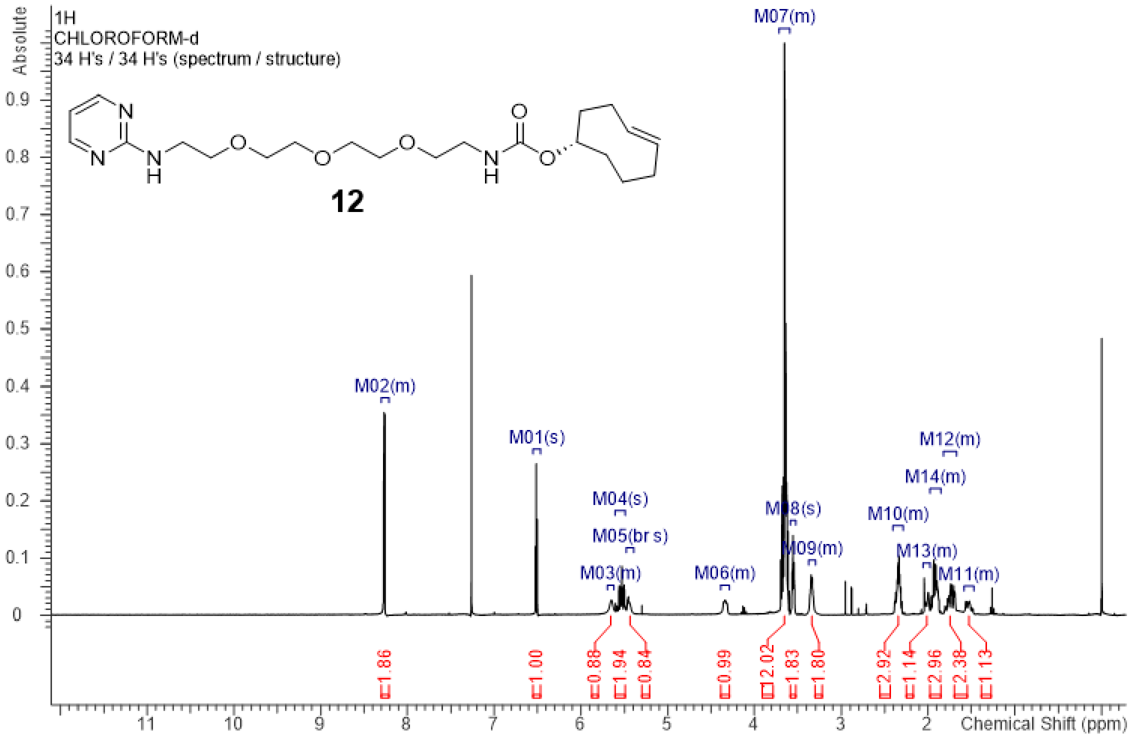

Figure S80. ^{1}H NMR spectrum of compound **12**

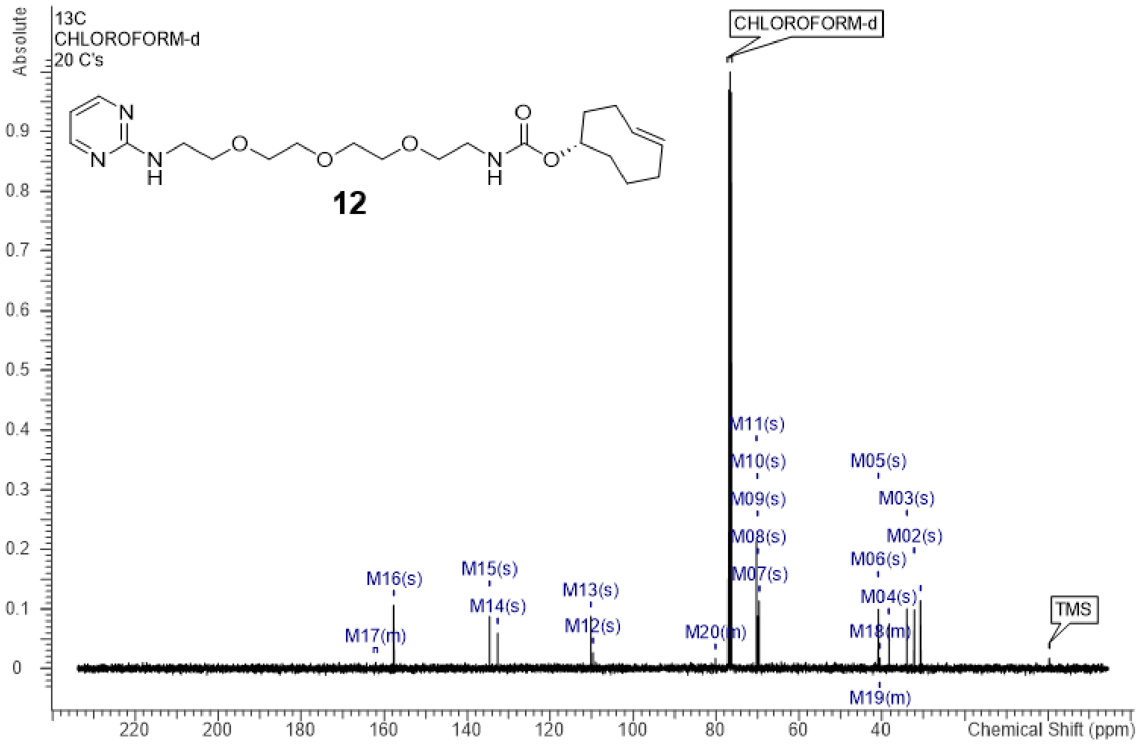

Figure S81. *^{13}C NMR spectrum of compound* ***12***

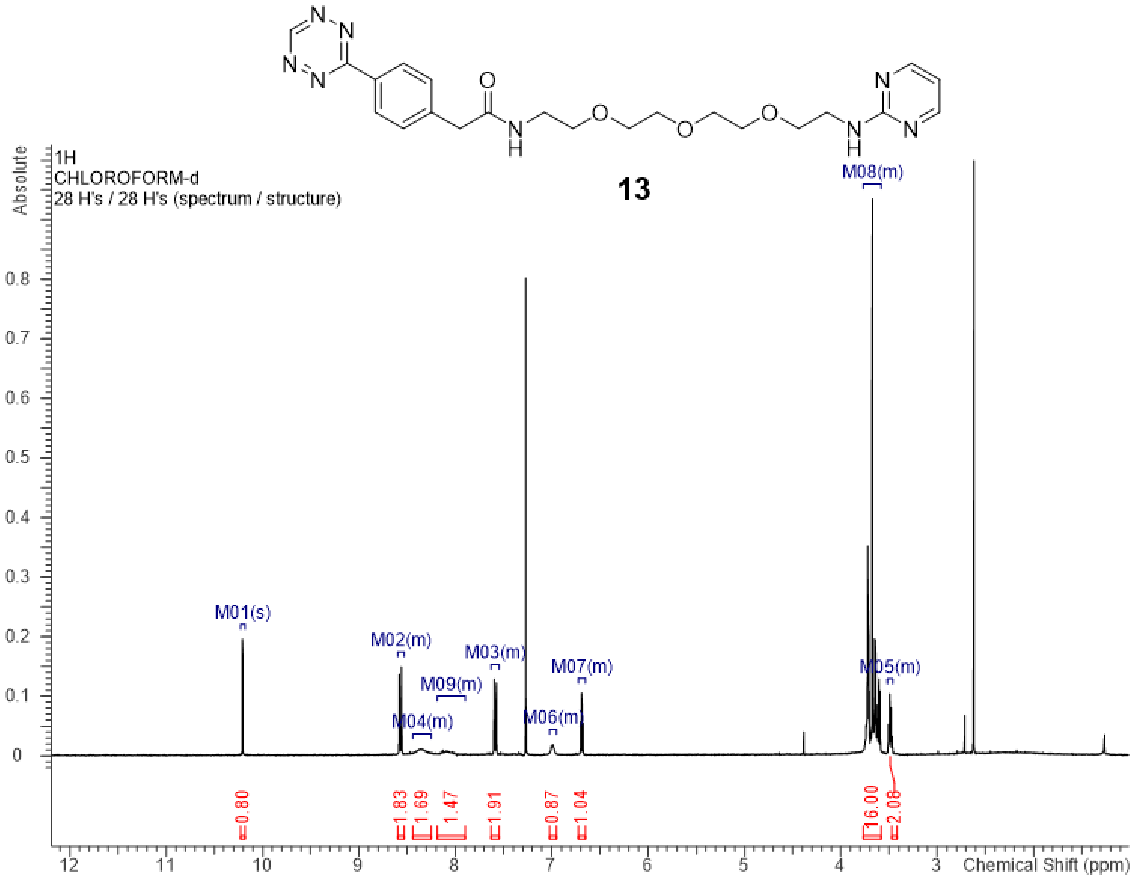

Figure S82. *^{1}H NMR spectrum of compound* ***13***

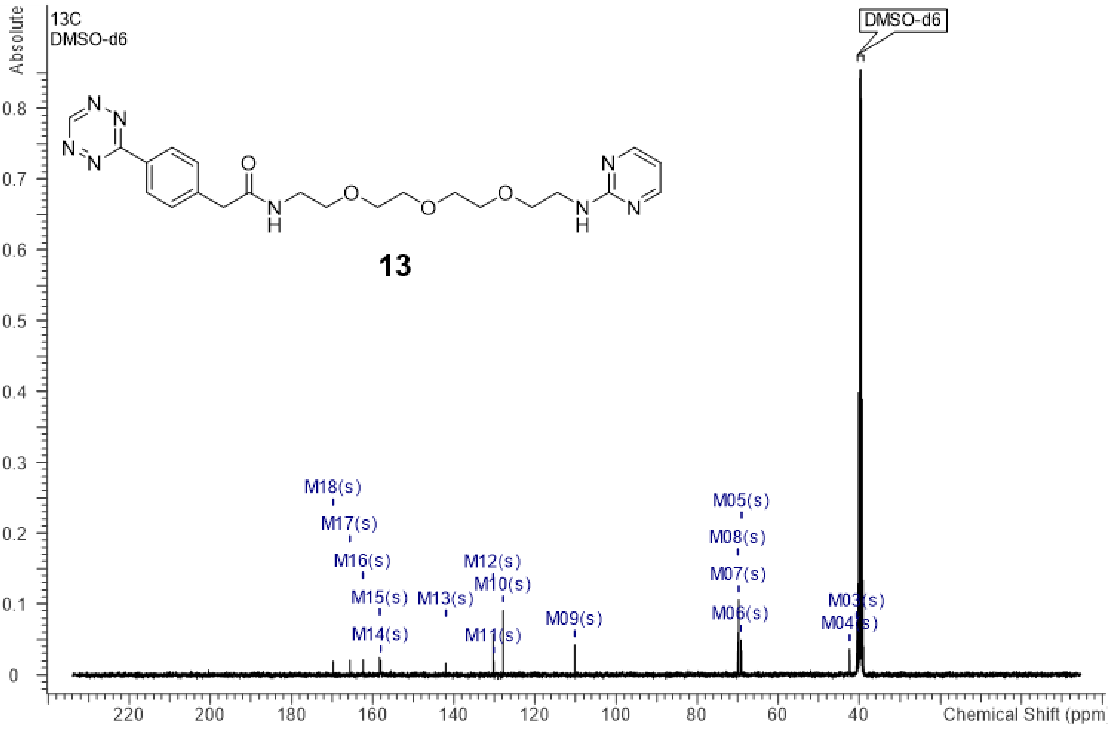

Figure S83. *^{13}C NMR spectrum of compound* ***13***

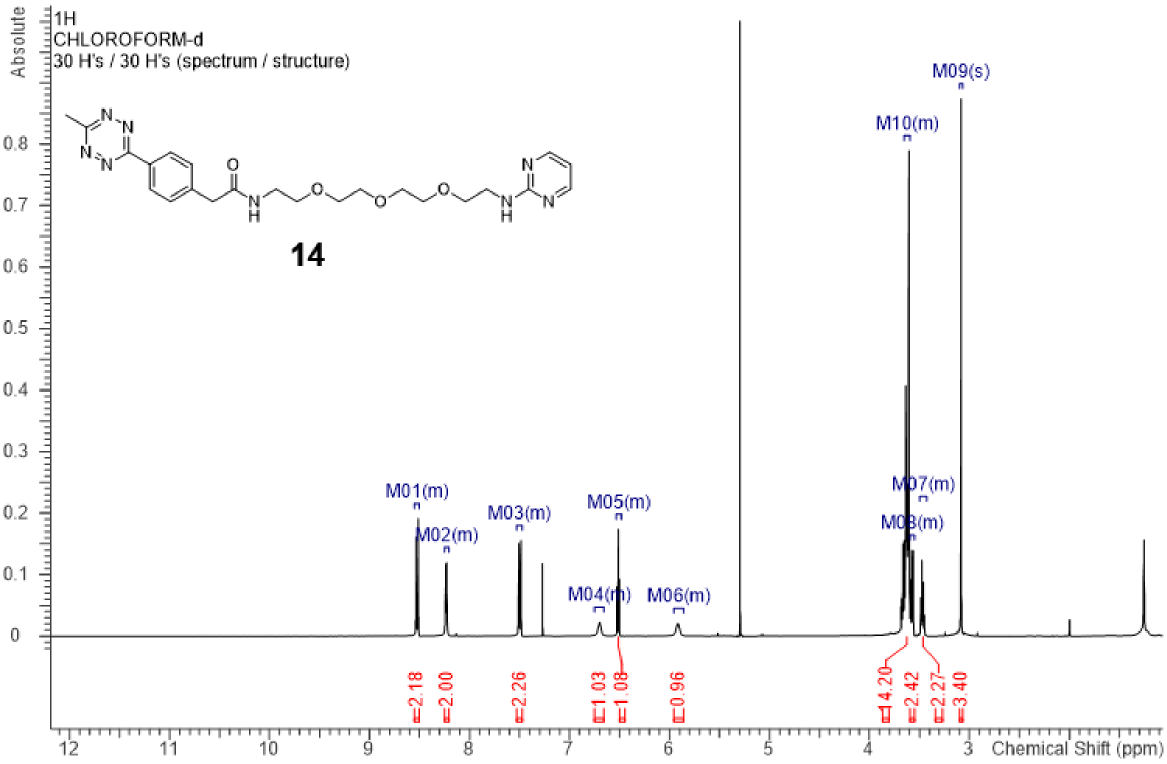

Figure S84. *^{1}H NMR spectrum of compound* ***14***

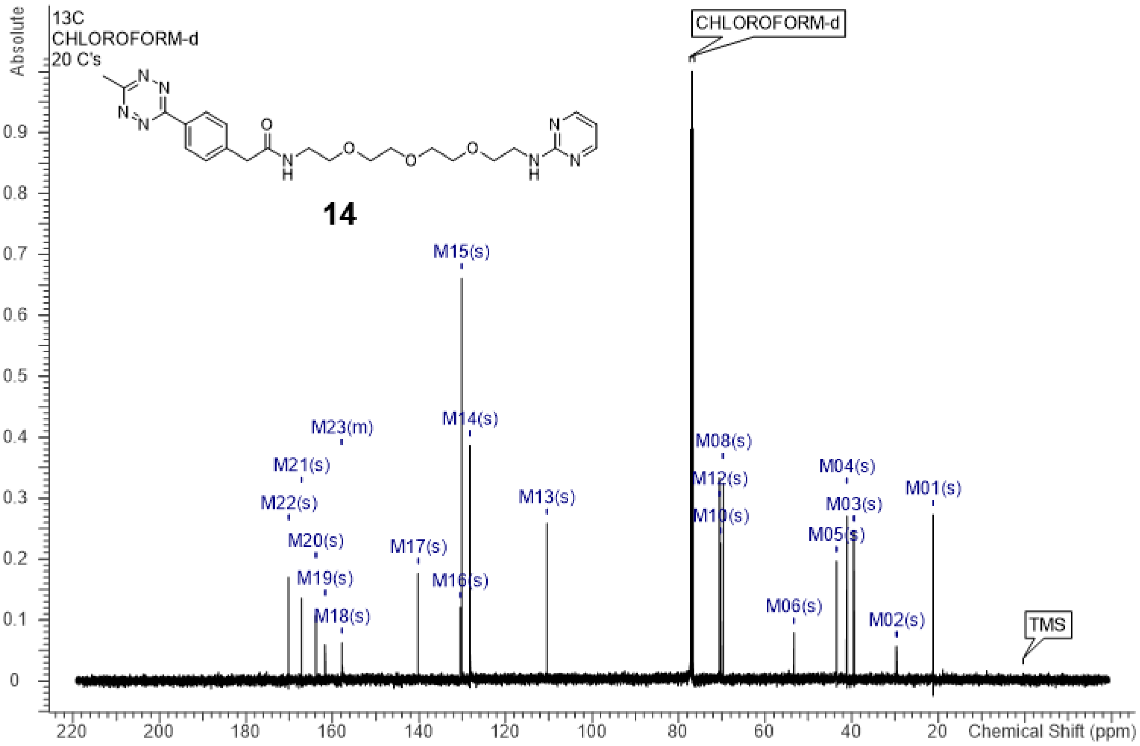

Figure S85. *^{13}C NMR spectrum of compound **14***

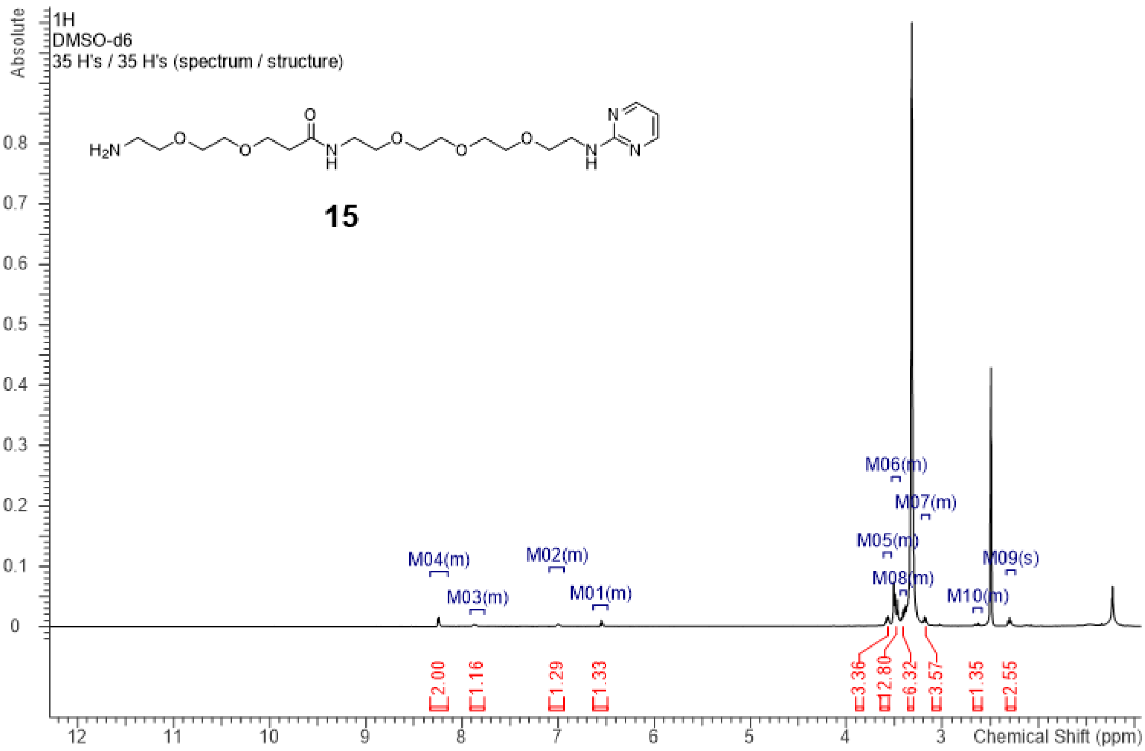

Figure S86. *^{1}H NMR spectrum of compound **15***

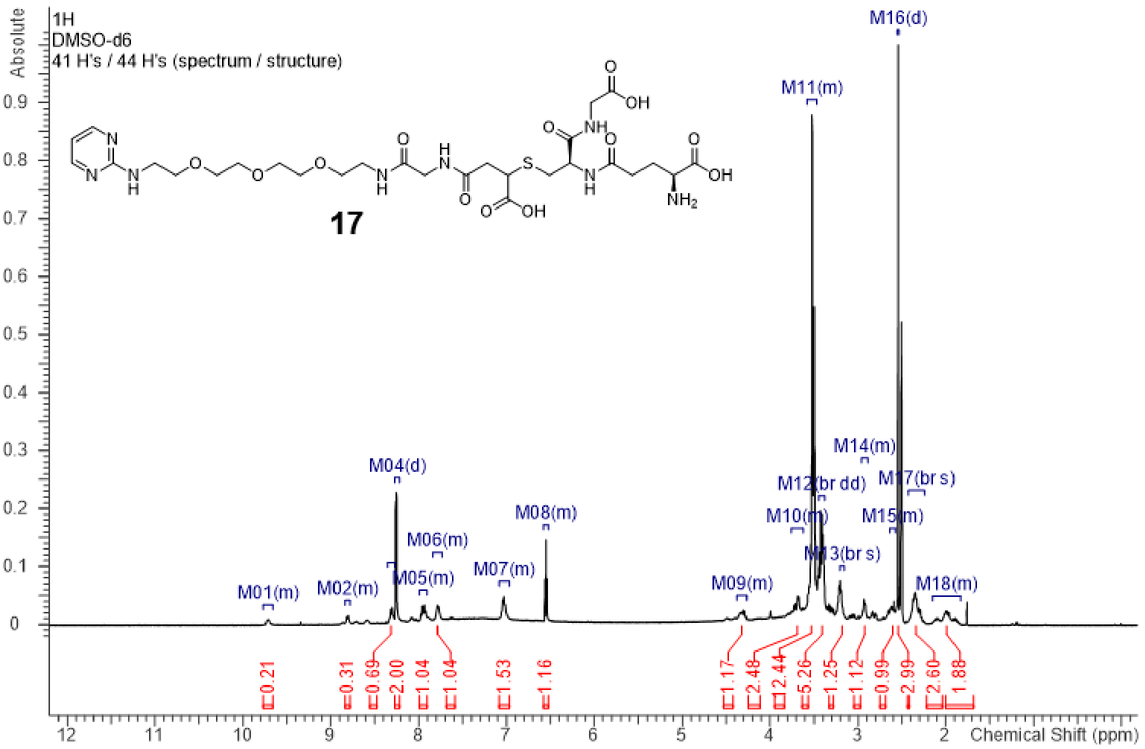

Figure S87. [1]*H NMR spectrum of compound* ***17***

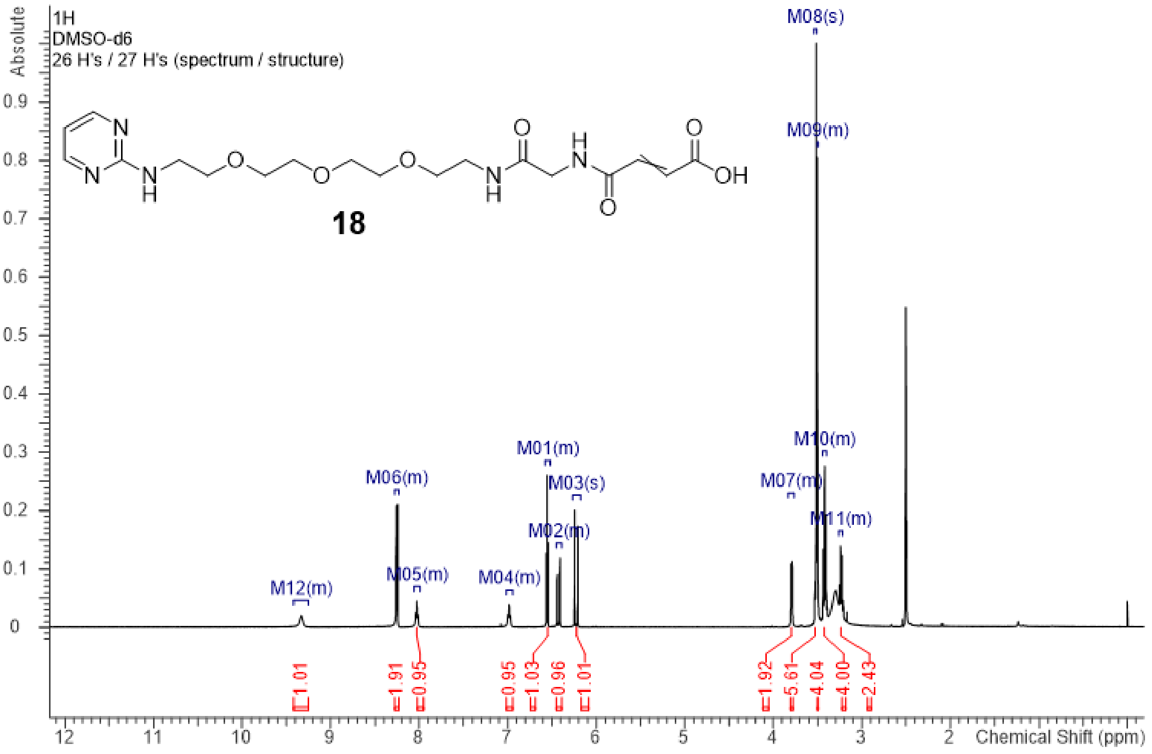

Figure S88. [1]*H NMR spectrum of compound* ***18***

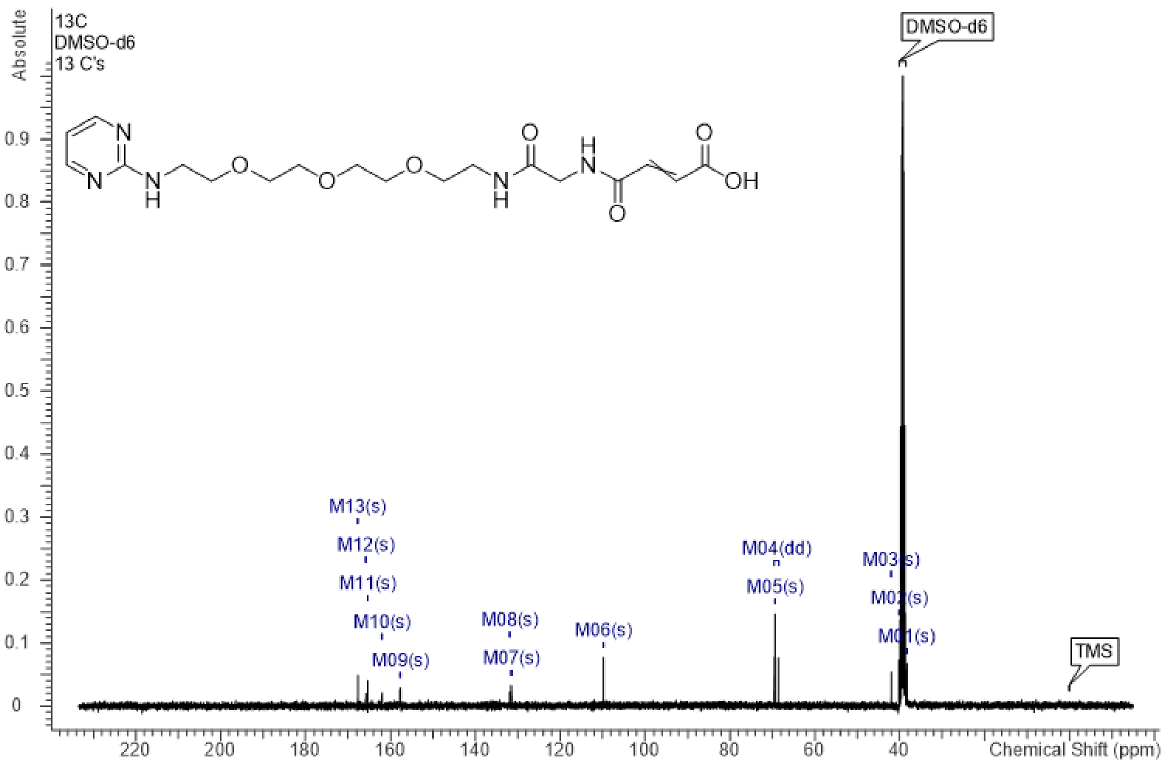

Figure S89. ^{13}C NMR spectrum of compound ***18***

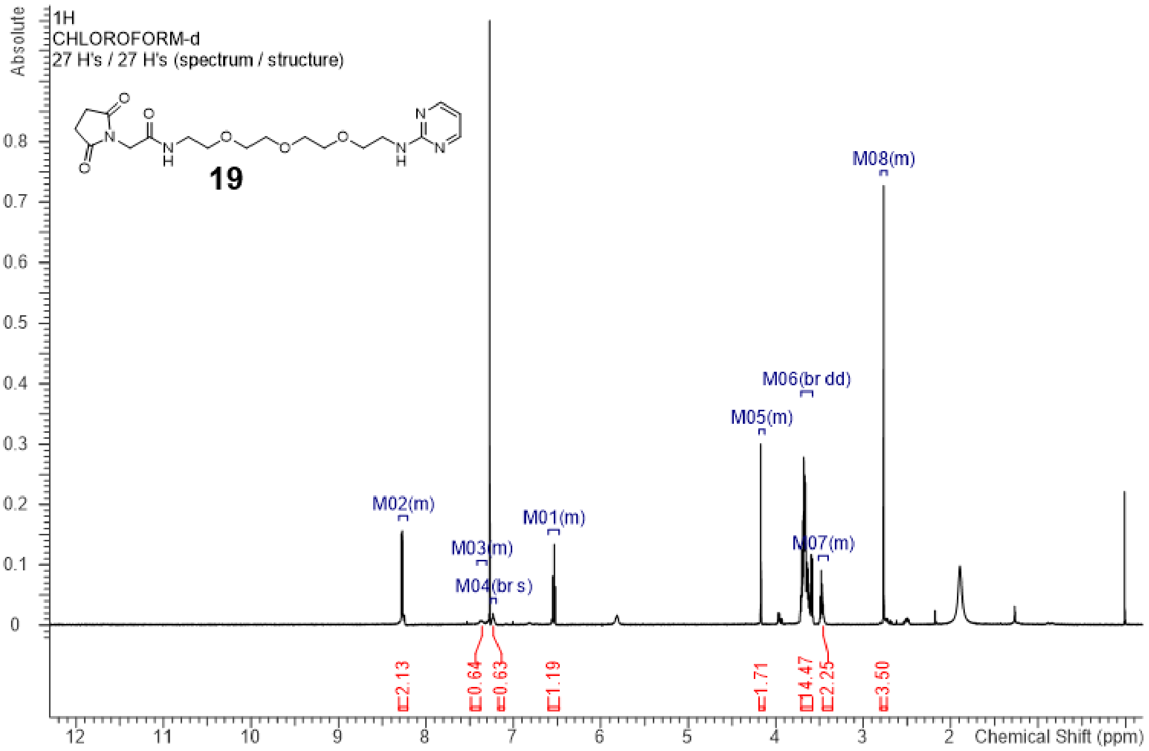

Figure S90. ^{1}H NMR spectrum of compound ***19***

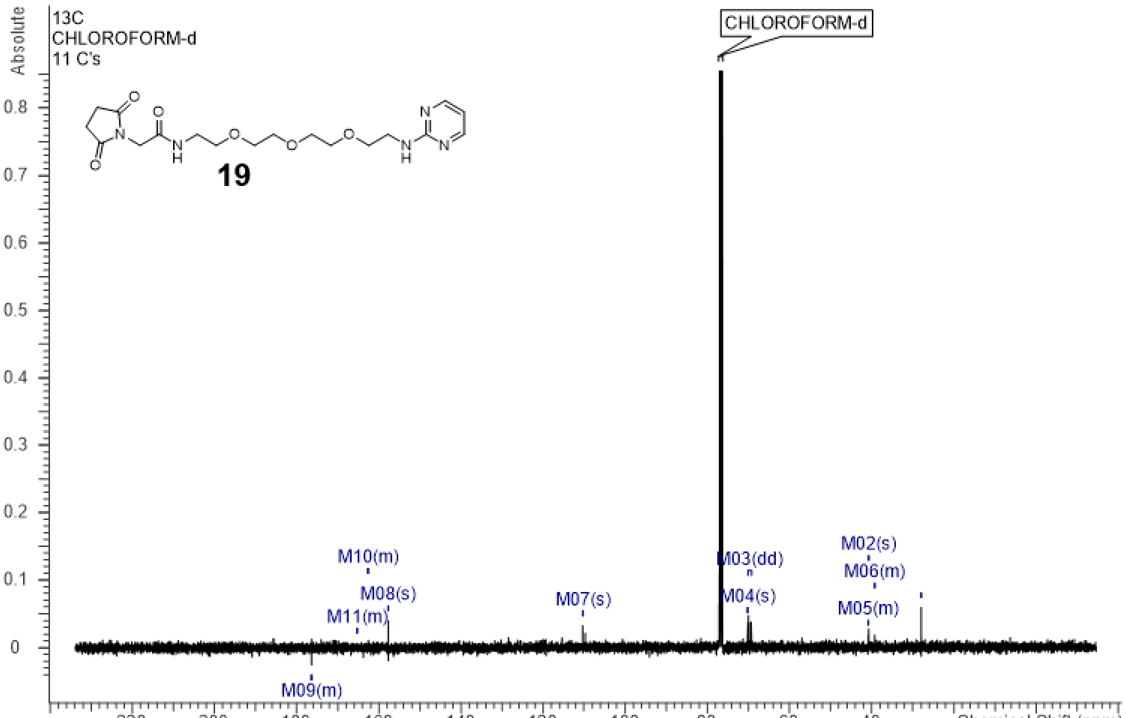

Figure S91. *^{13}C NMR spectrum of compound* ***19***

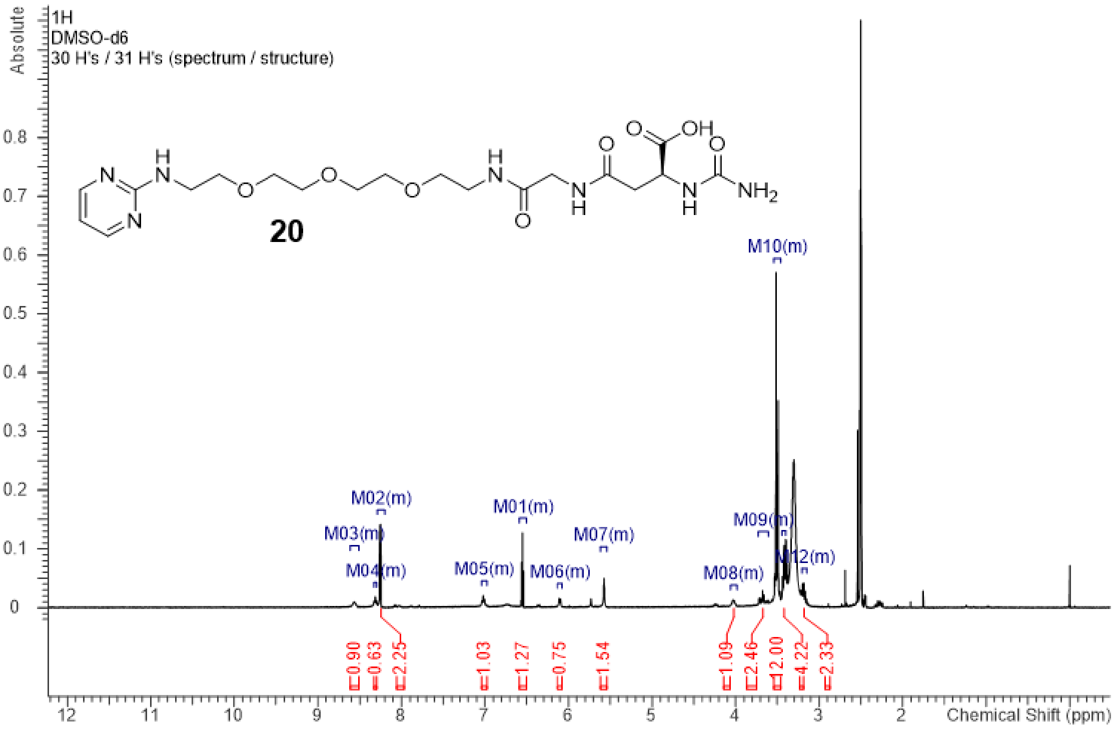

Figure S92. *^{1}H NMR spectrum of compound* ***20***

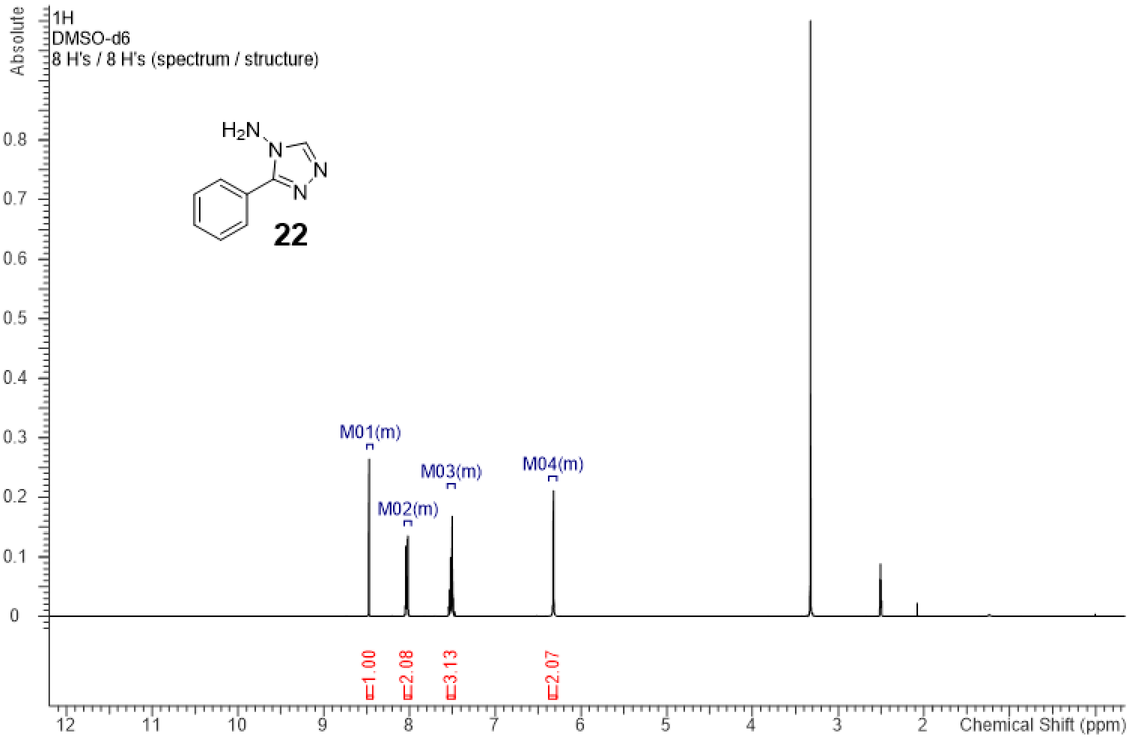

Figure S94. ^{1}H NMR spectrum of compound **22**

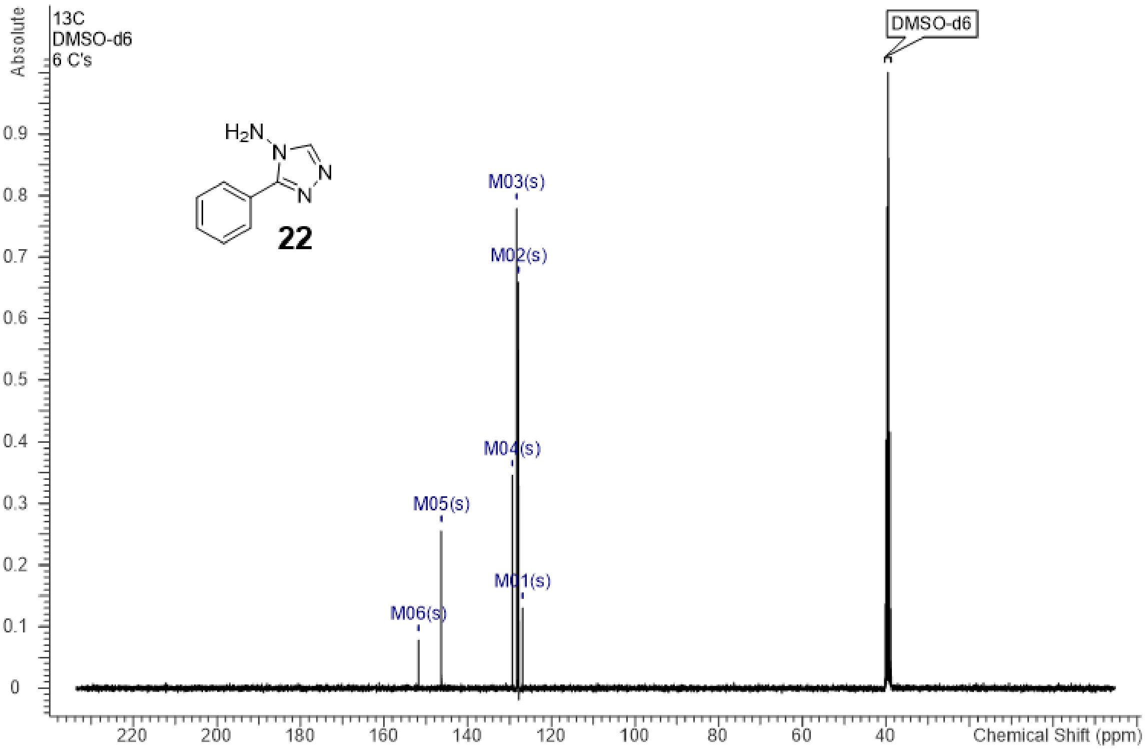

Figure S95. ^{13}C NMR spectrum of compound **22**

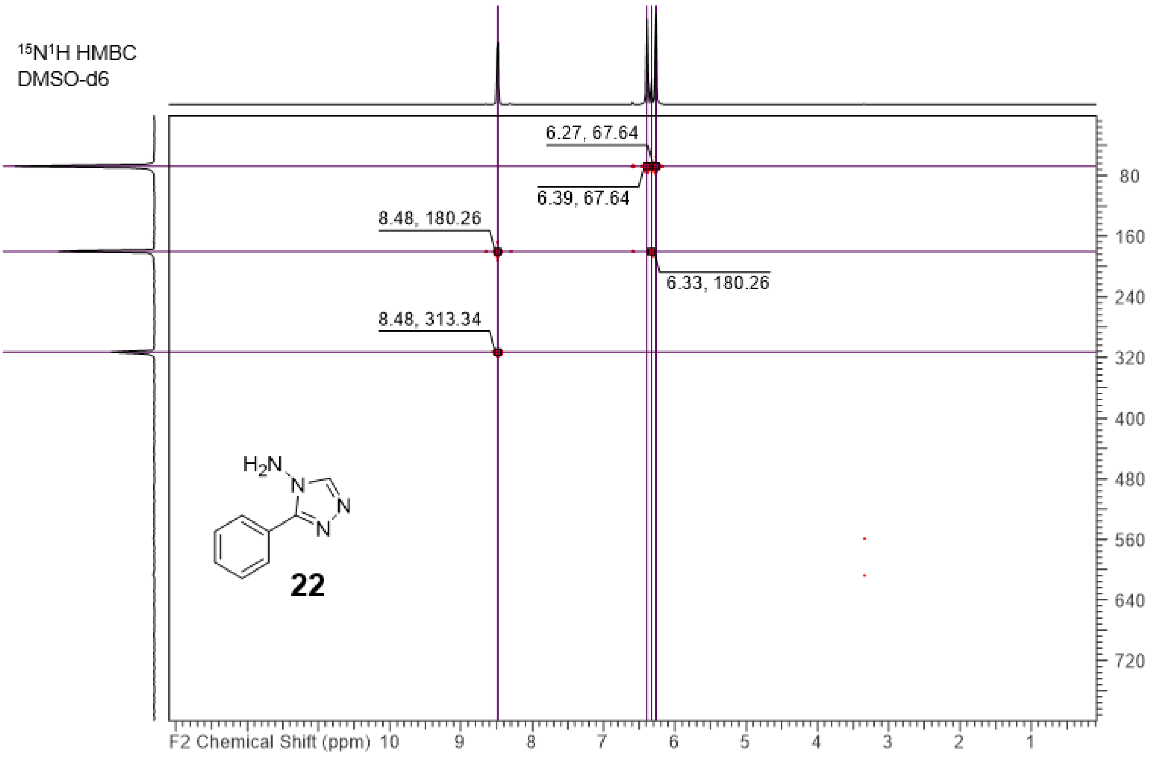

Figure S96. *$^{15}N^{1}H$ HMBC NMR spectrum of compound **22***

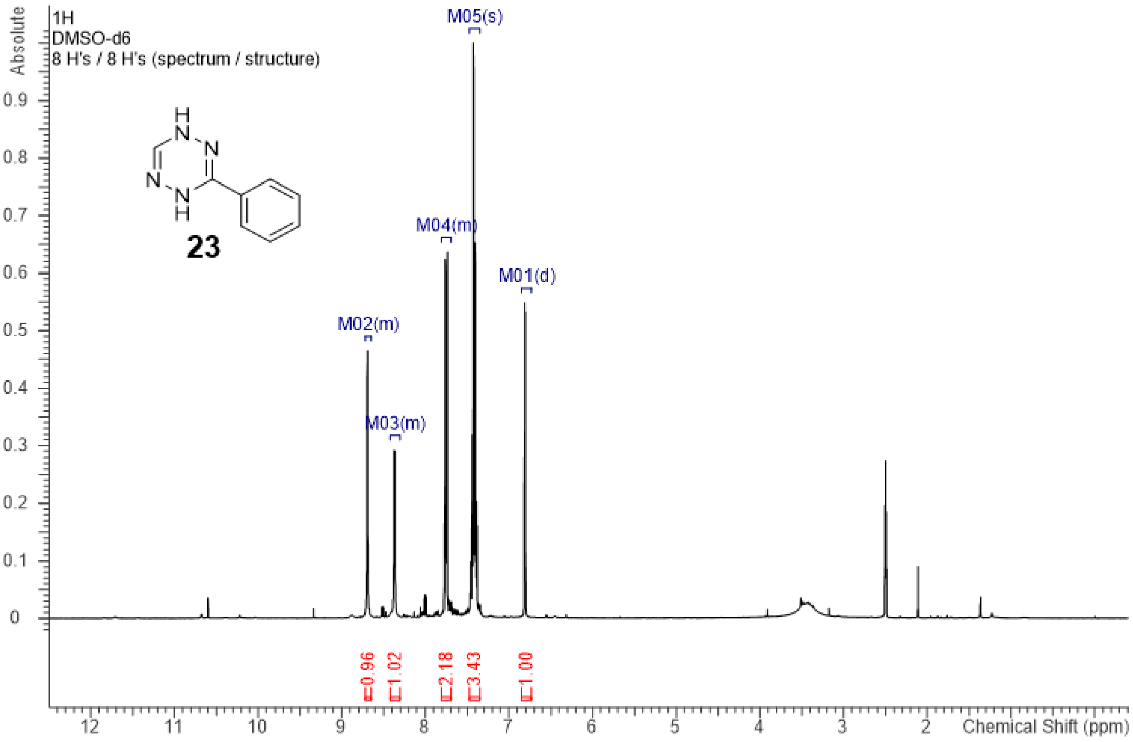

Figure S97. *^{1}H NMR spectrum of compound **23***

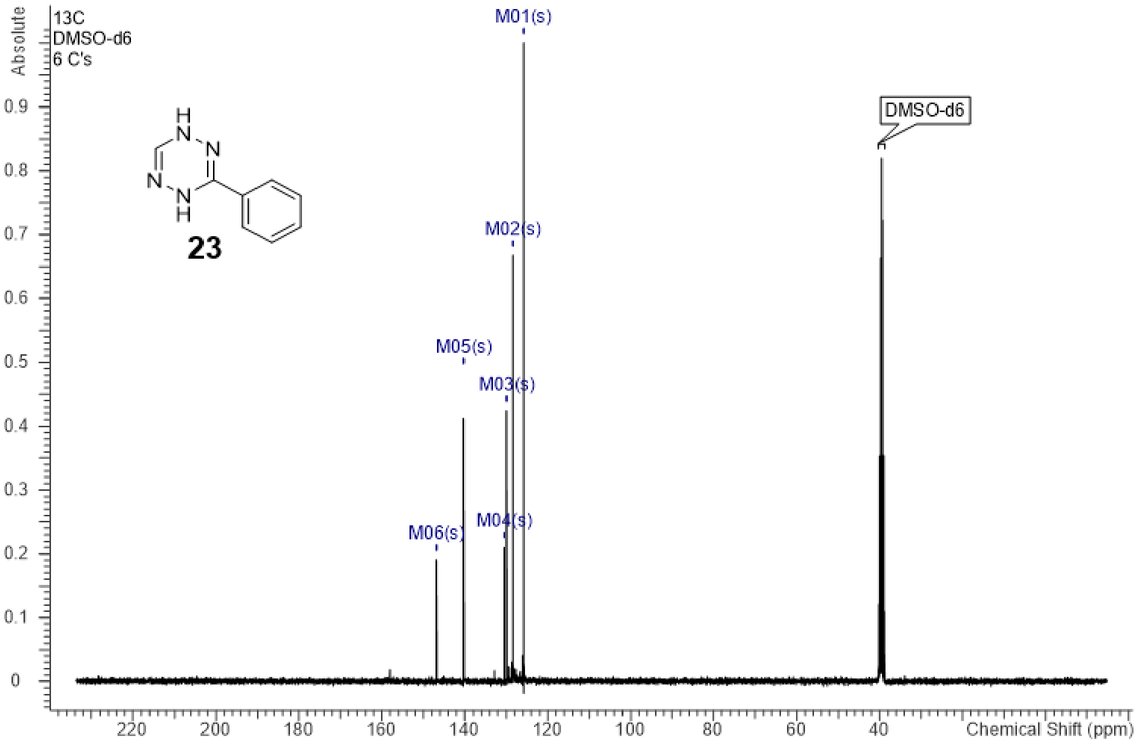

Figure S98. *^{13}C NMR spectrum of compound **23***

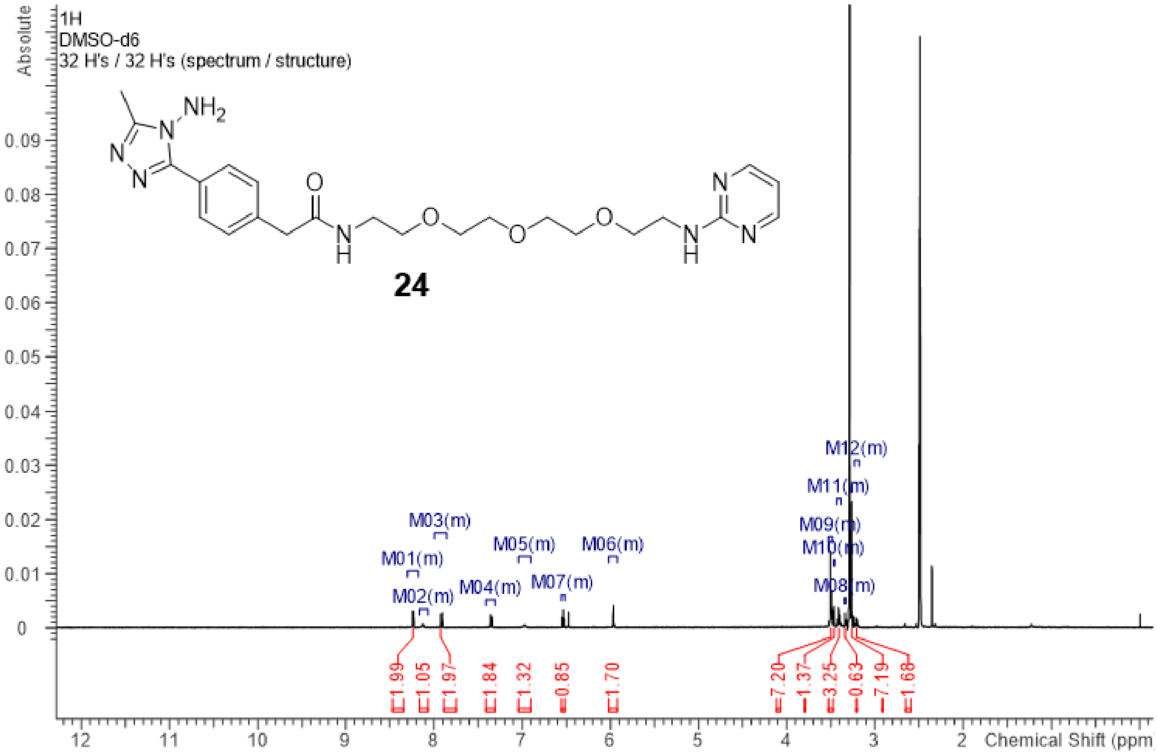

Figure S99. *^{1}H NMR spectrum of compound **24***

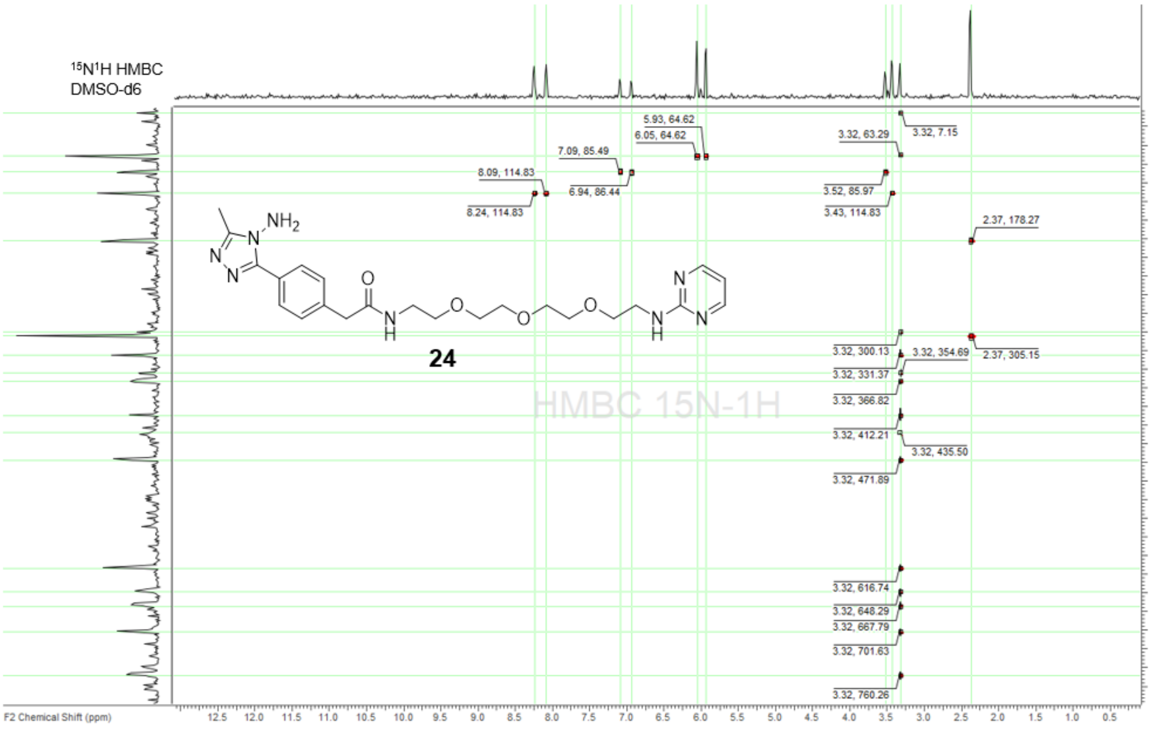

Figure S100. *$^{15}N^{1}H$ HMBC NMR spectrum of compound* ***24***

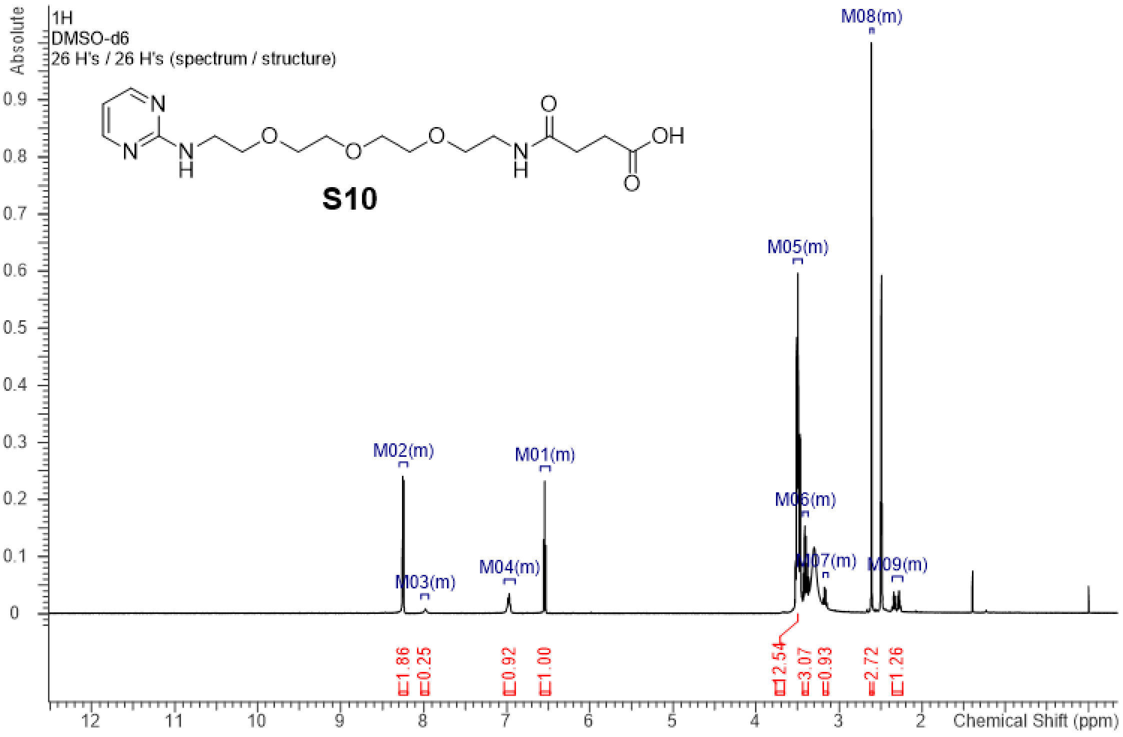

Figure S101. *^{1}H NMR spectrum of compound* ***S10***

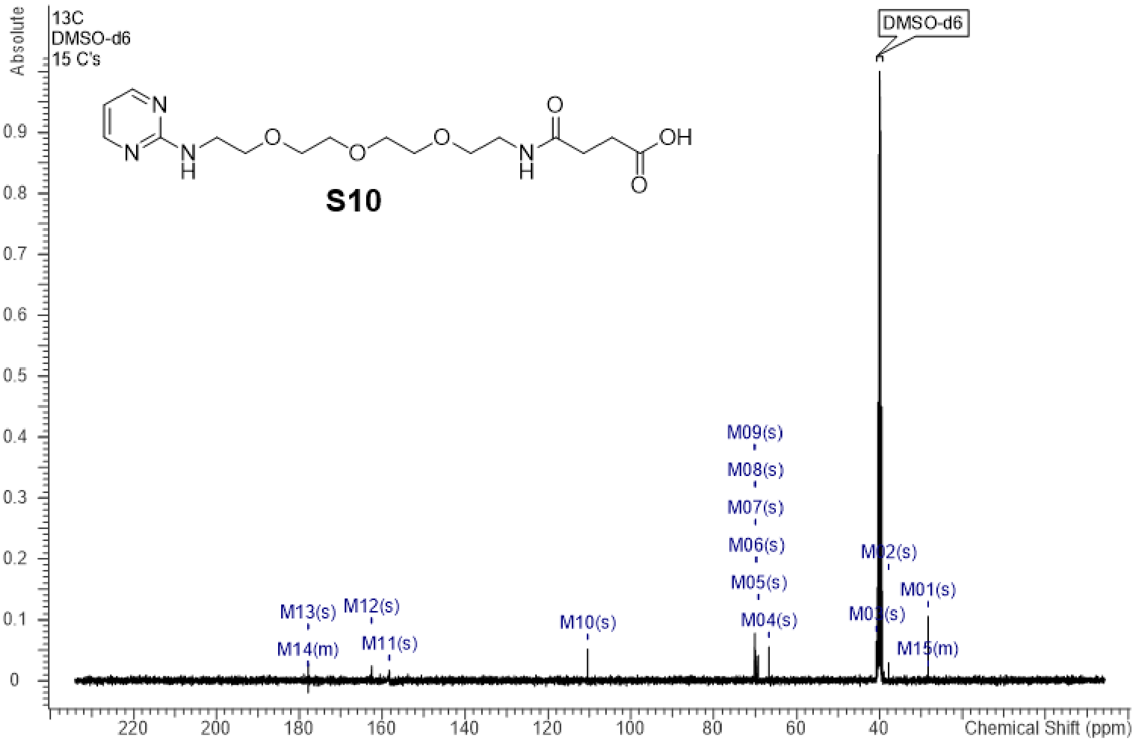

Figure S102. ^{13}*C NMR spectrum of compound* ***S10***

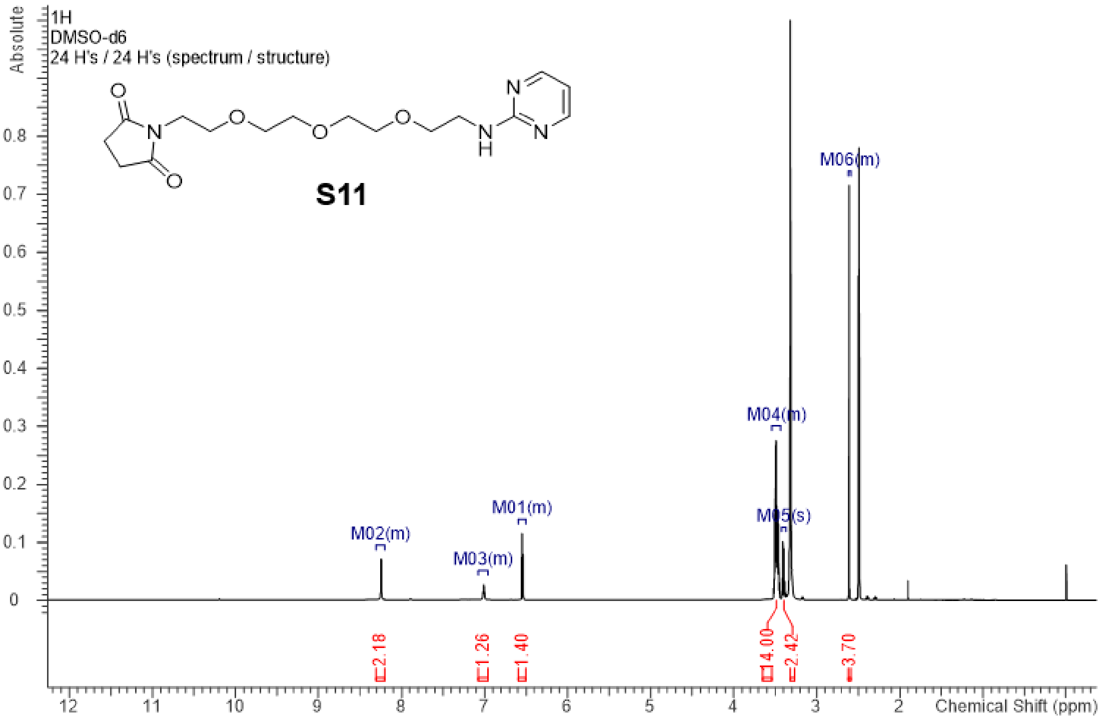

Figure S103. ^{1}*H NMR spectrum of compound* ***S11***

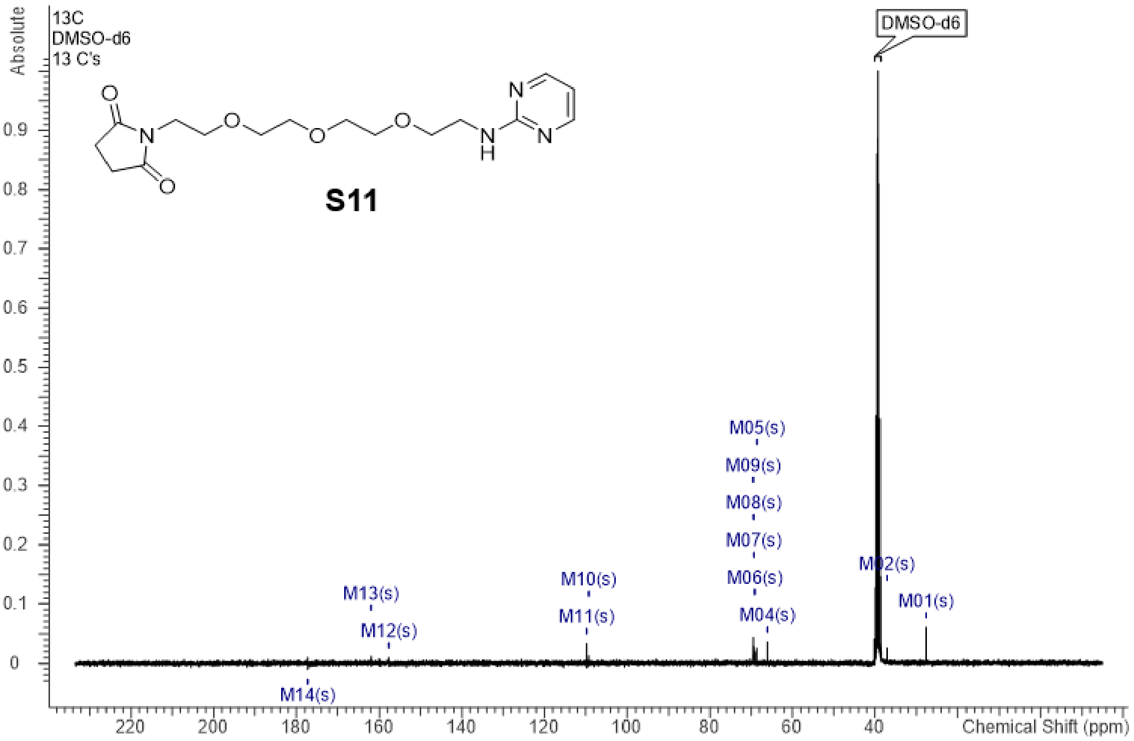

Figure S104. *^{13}C NMR spectrum of compound* ***S11***

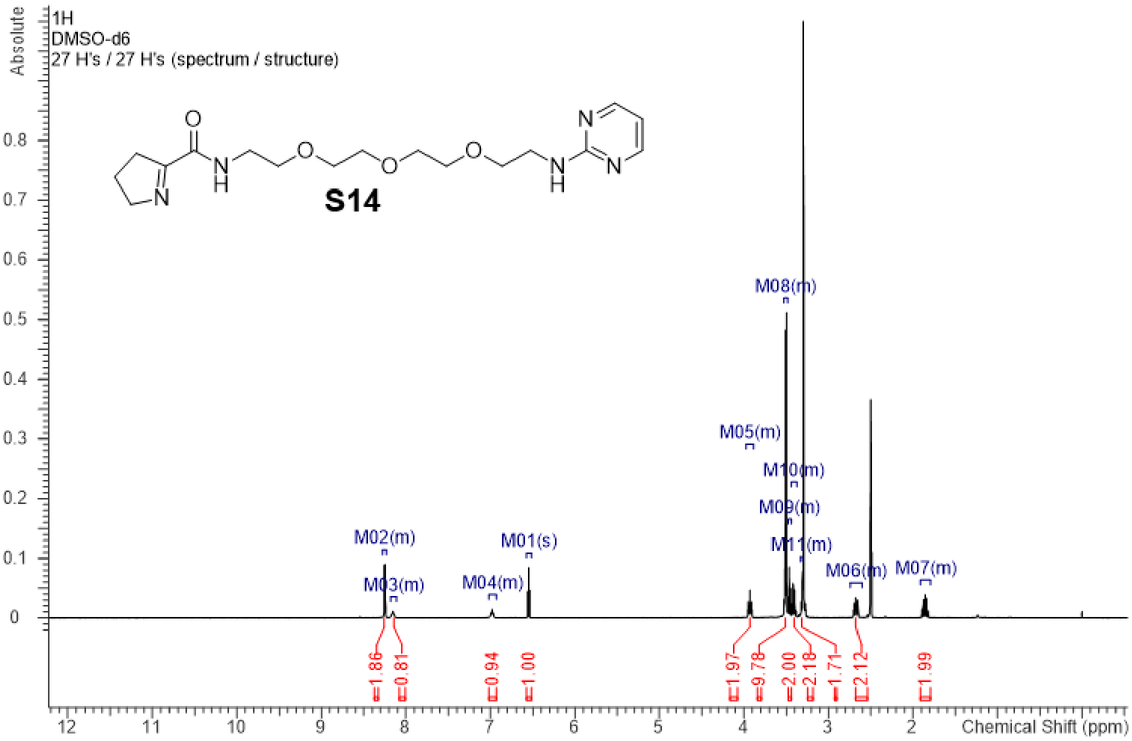

Figure S105. *^{1}H NMR spectrum of compound* ***S14***

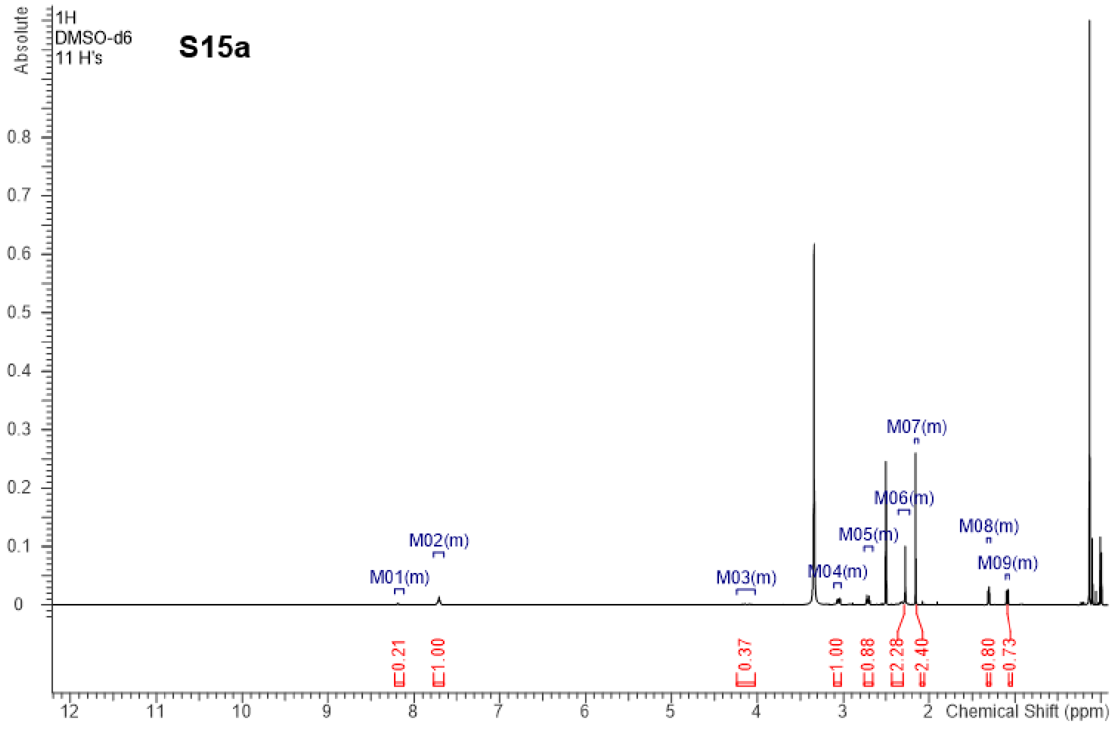

Figure S106. *¹H NMR spectrum of compound* ***S15a***

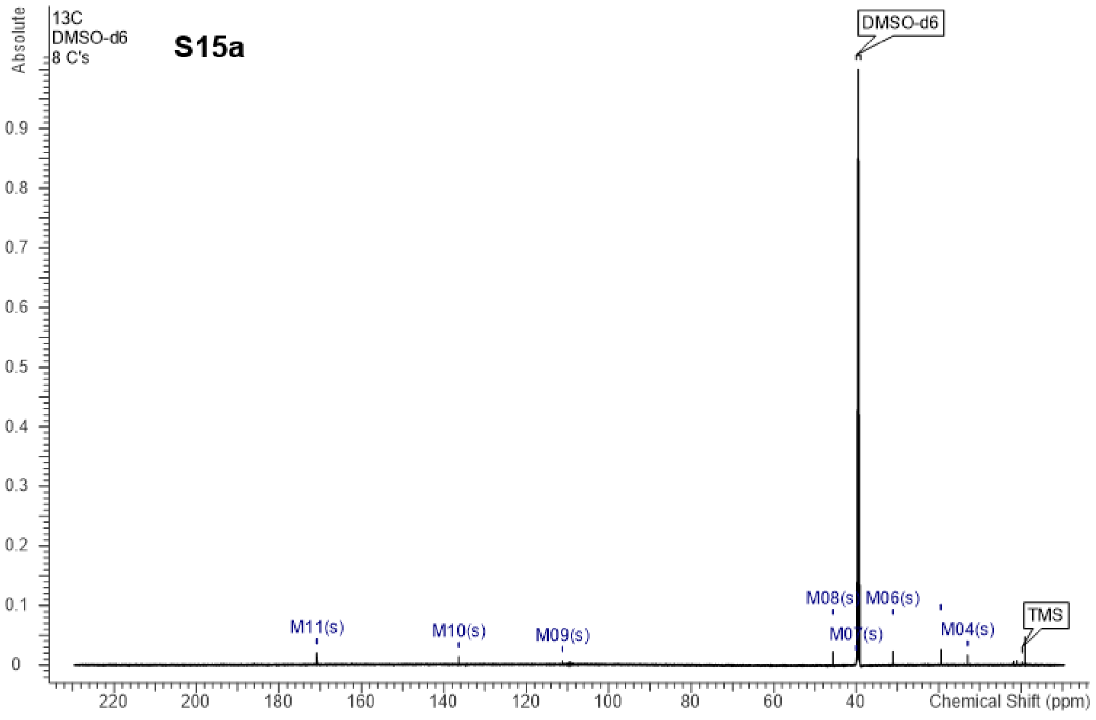

Figure S107. *¹³C NMR spectrum of compound* ***S15a***

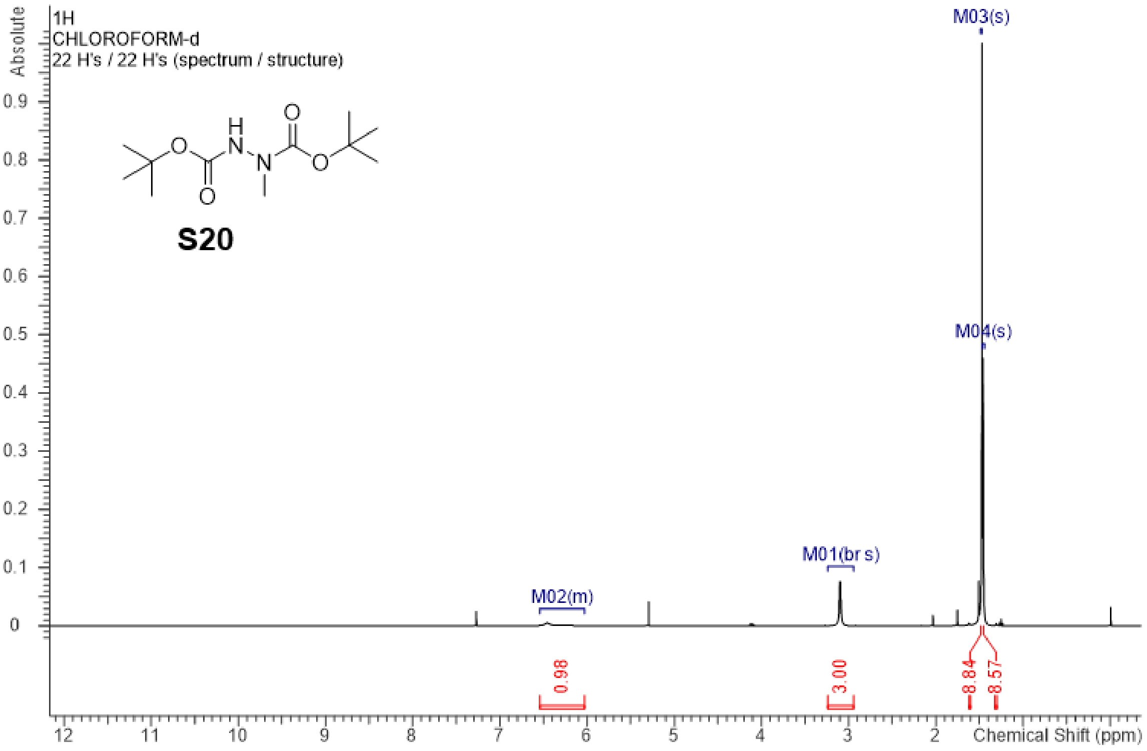

Figure S108. [1]*H NMR spectrum of compound* ***S20.***

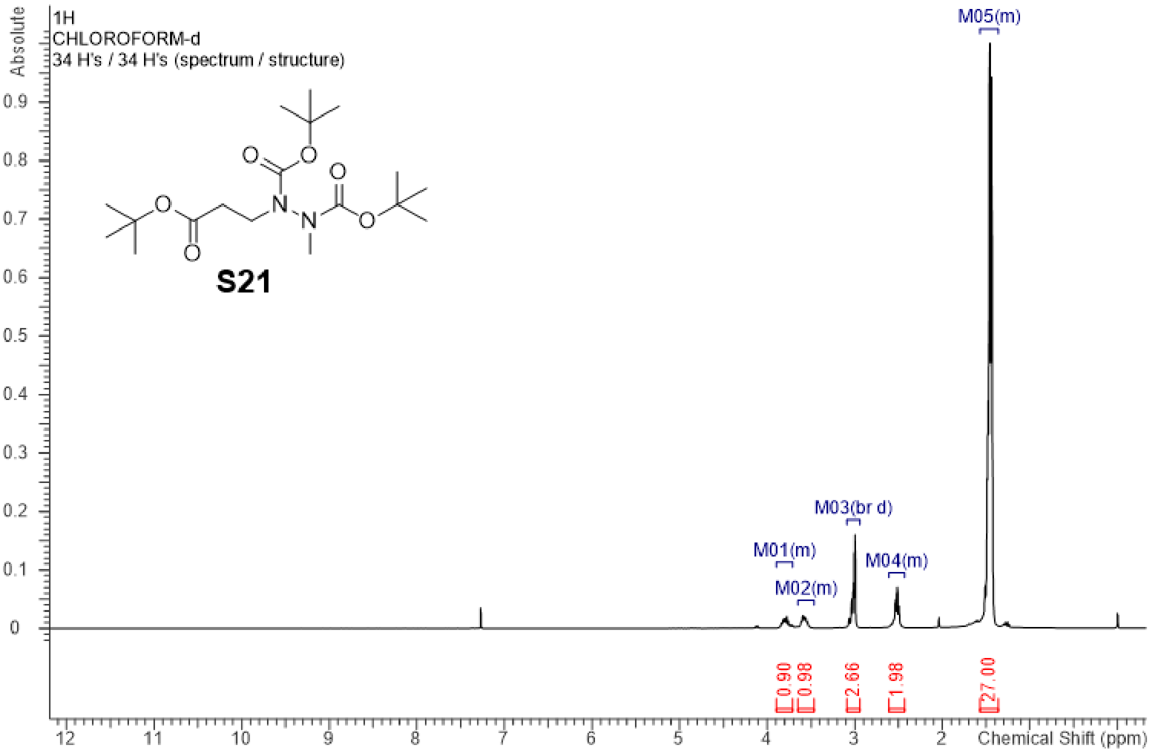

Figure S109. [1]*H NMR spectrum of compound* ***S21***

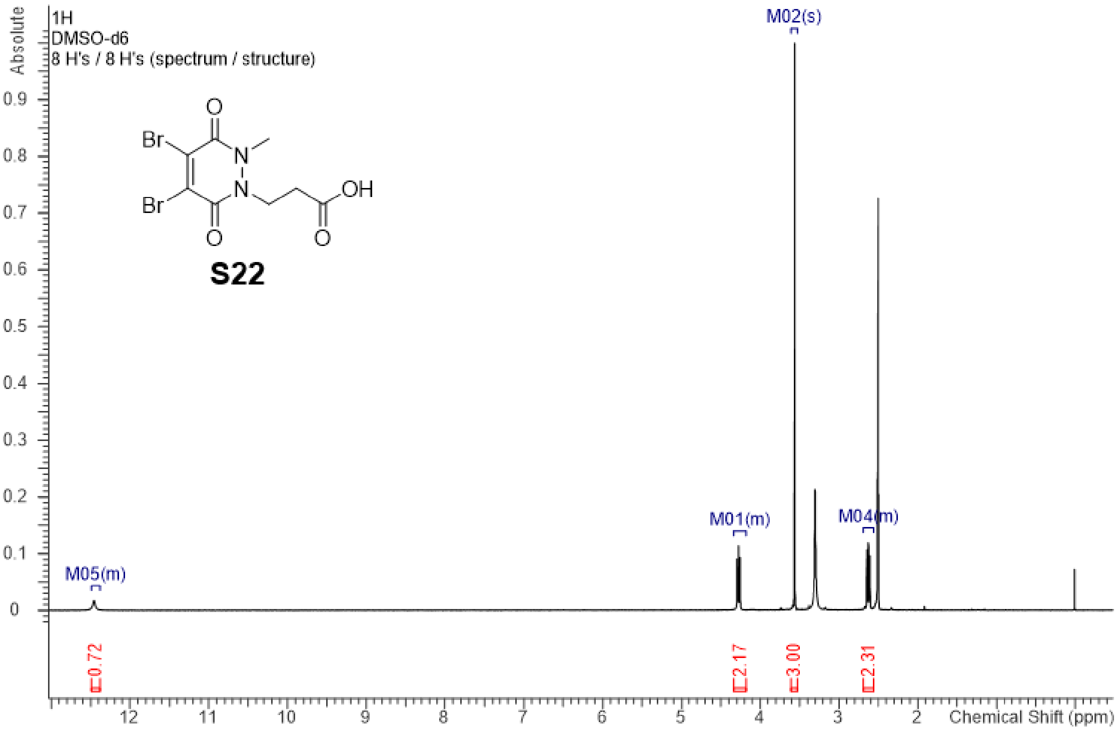

Figure S110. *¹H NMR spectrum of compound* ***S22***

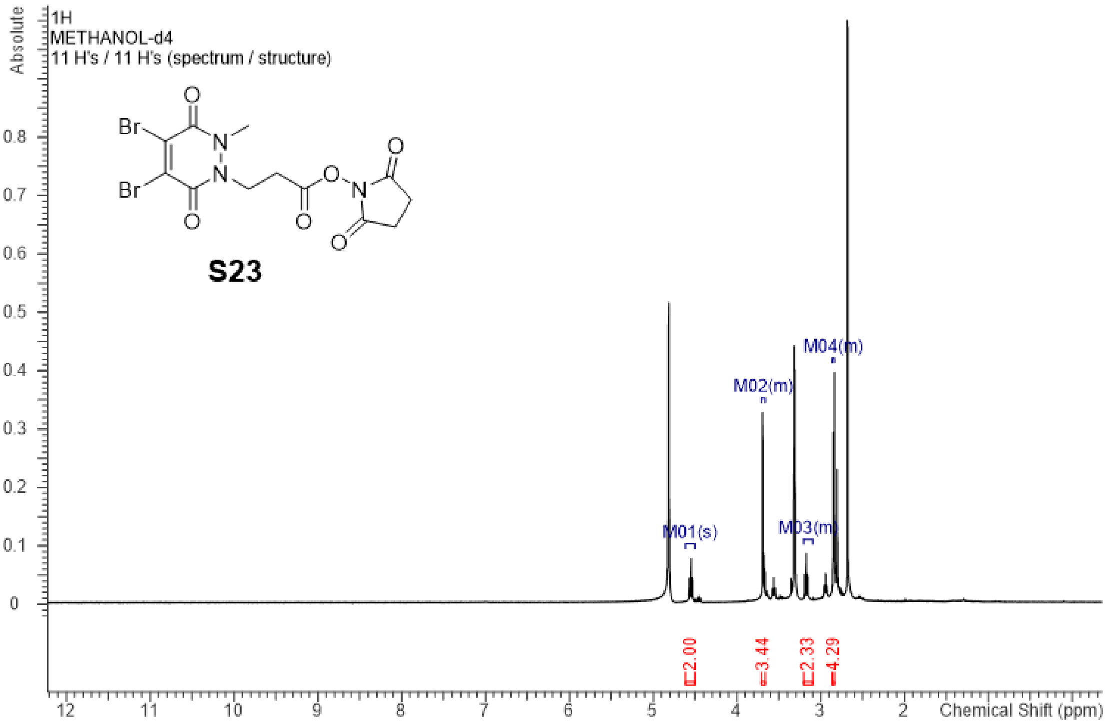

Figure S111. *¹H NMR spectrum of compound* ***S23***

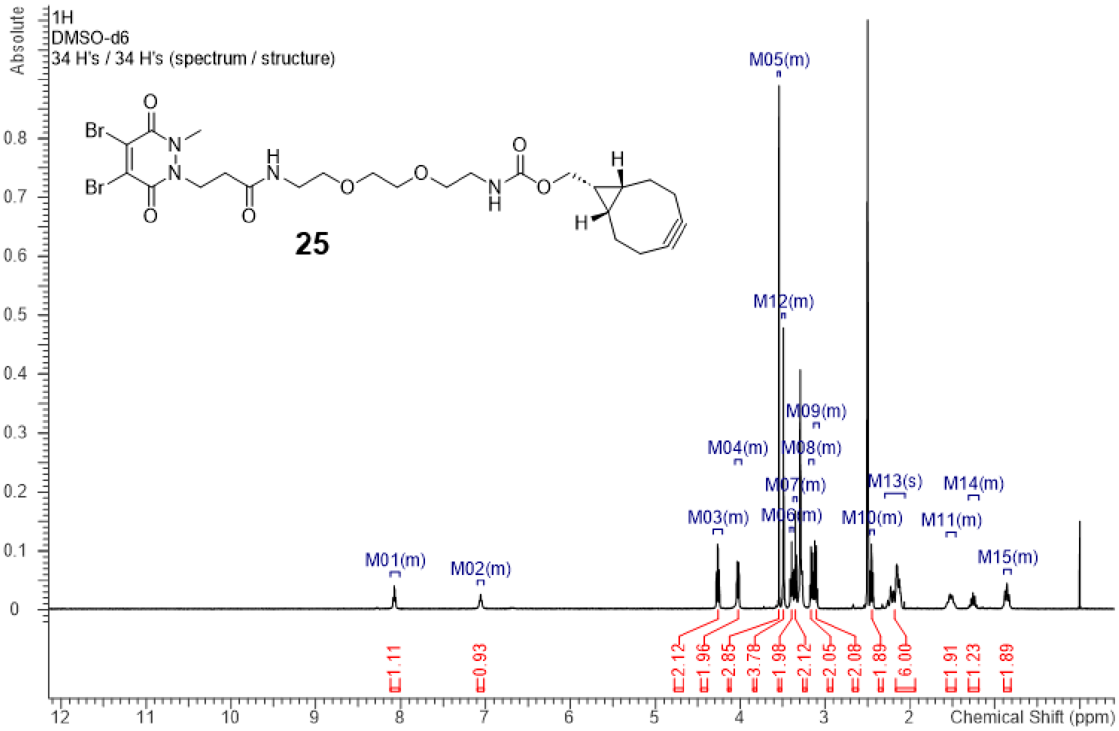

Figure S112. *^{1}H NMR spectrum of compound* ***25***

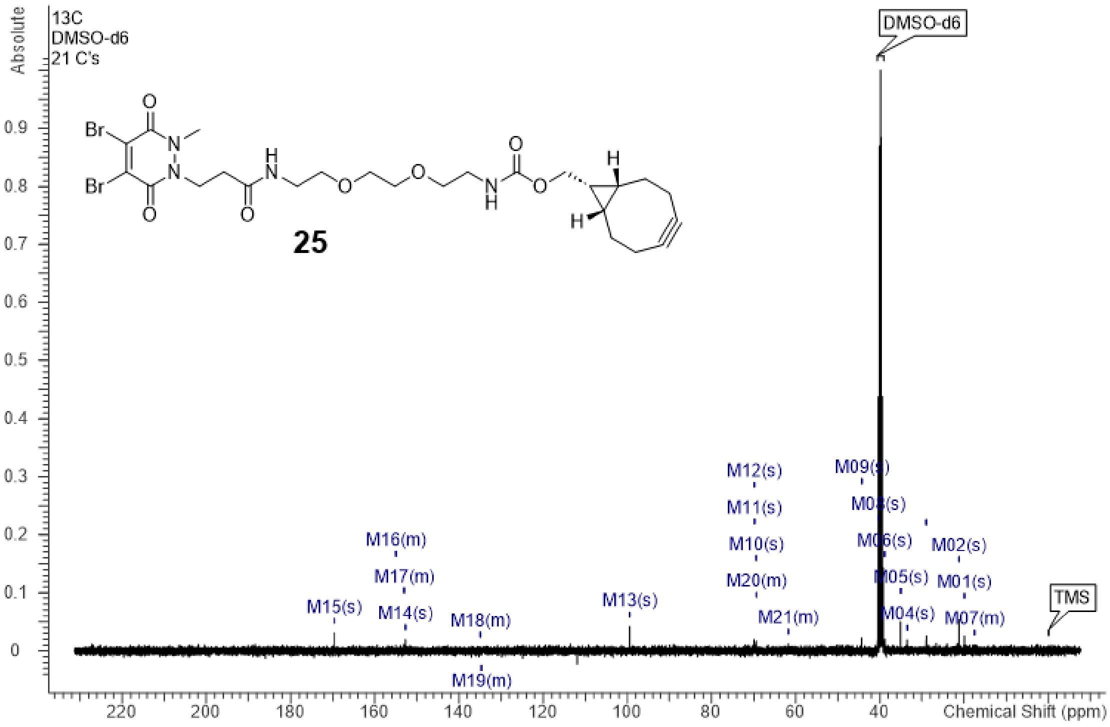

Figure S113. *^{13}C NMR spectrum of compound* ***25***

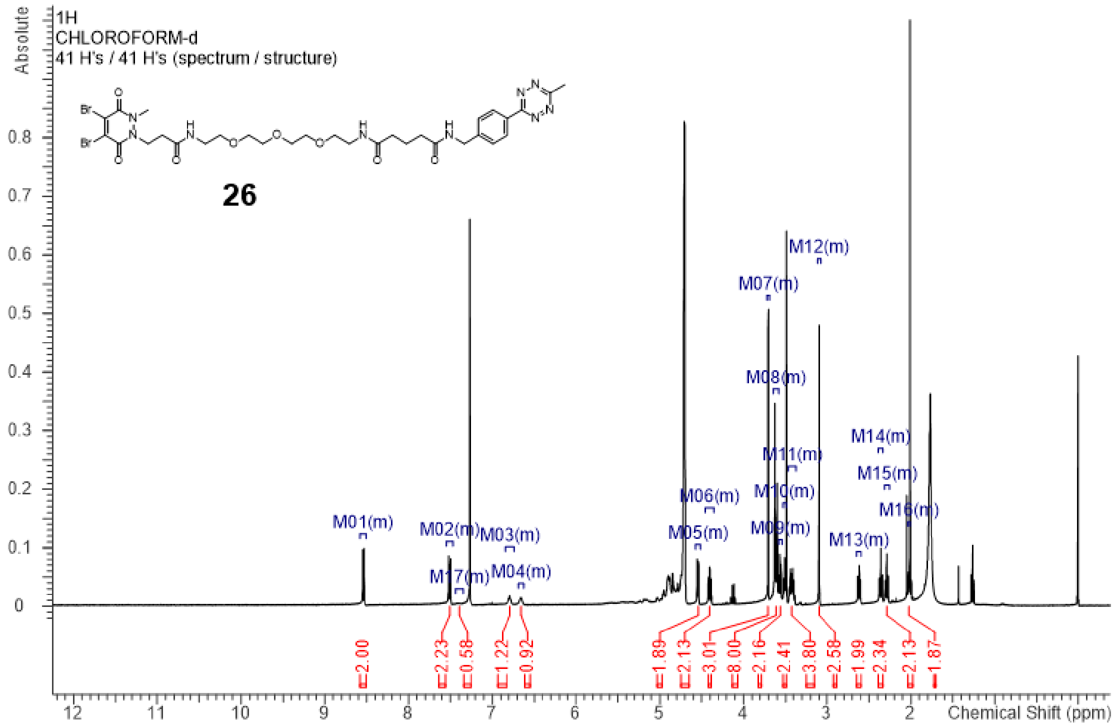

Figure S114. *¹H NMR spectrum of compound* ***26***

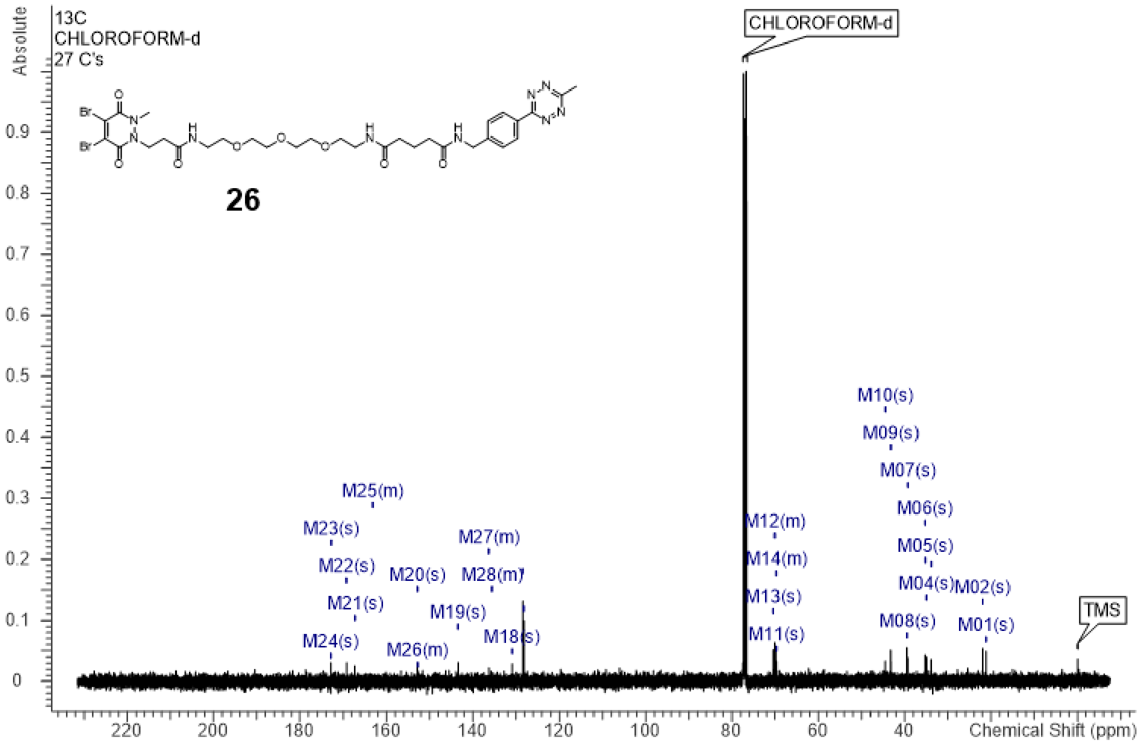

Figure S115. *¹³C NMR spectrum of compound* ***26***

Copies of protein analysis data

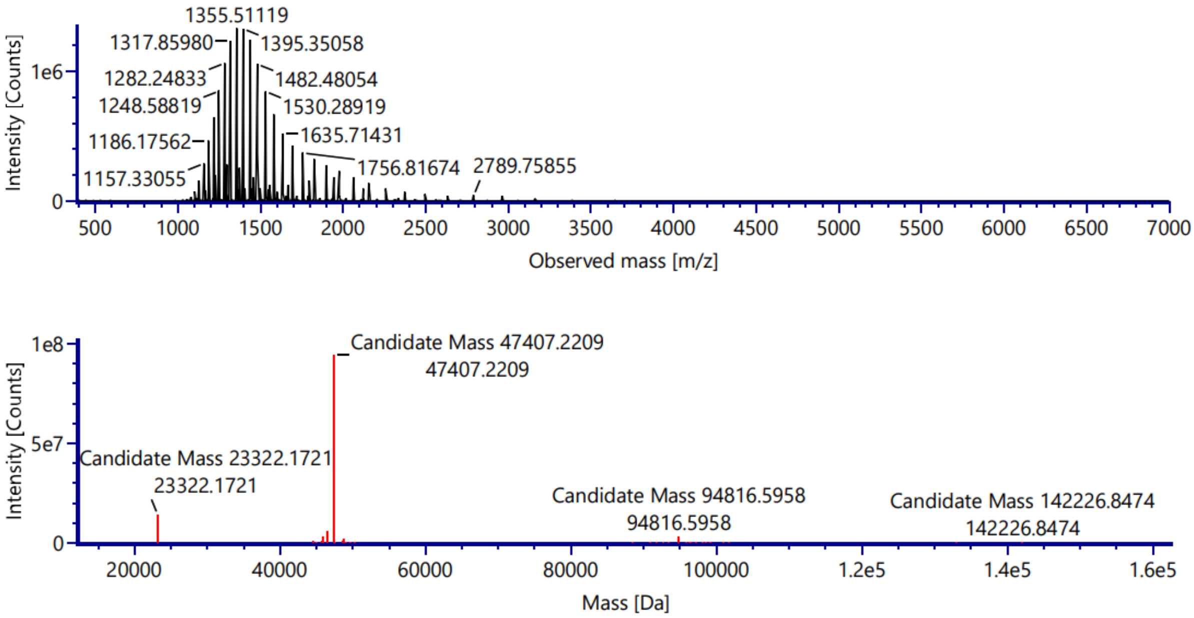

Figure S116. *LCMS spectrum of Fab*

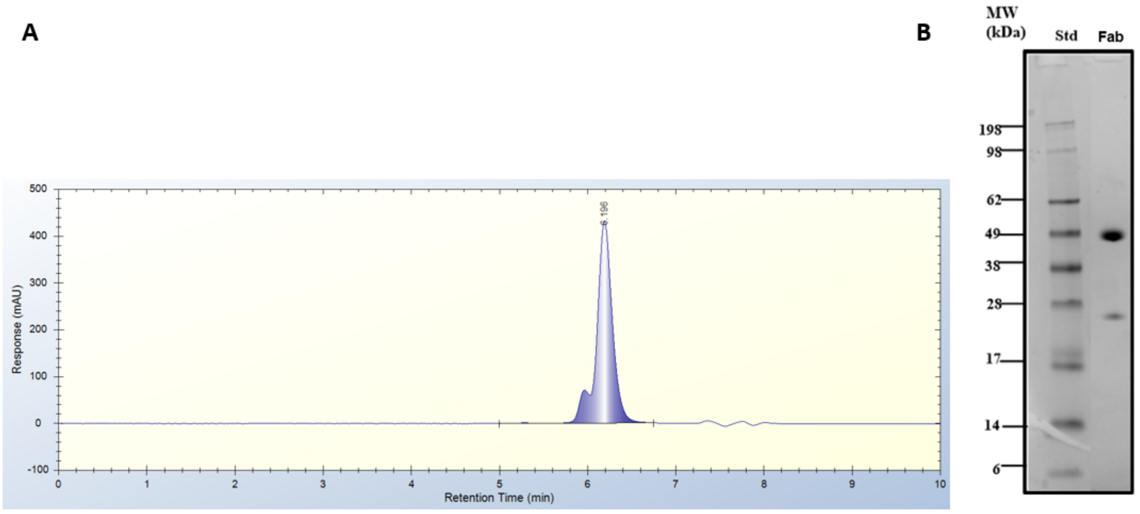

Figure S117. *(A) aSEC spectrum of Fab, major signal assigned to Fab, (B) SDS-PAGE of Fab, major signal at ~49 kDa assigned to Fab. Minor band assigned to free heavy chain or light chain (Fabs without formed interchain disulfide bonds likely to dissociate under conditions of SDS-PAGE).*

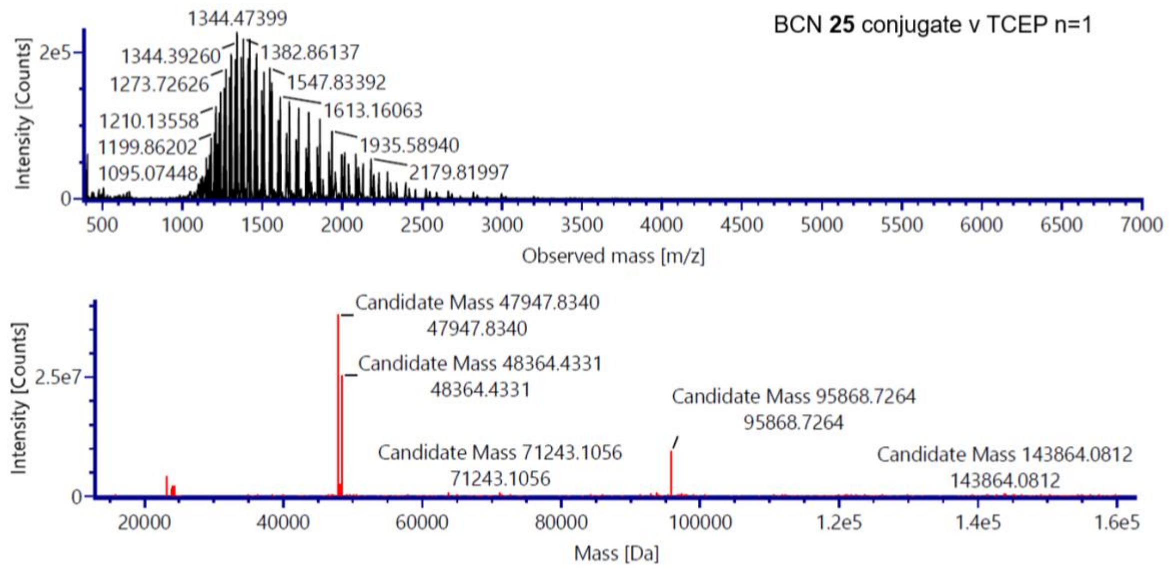

Figure S118. *LCMS spectrum of on protein stability of BCN **25** in TCEP. First replicate*

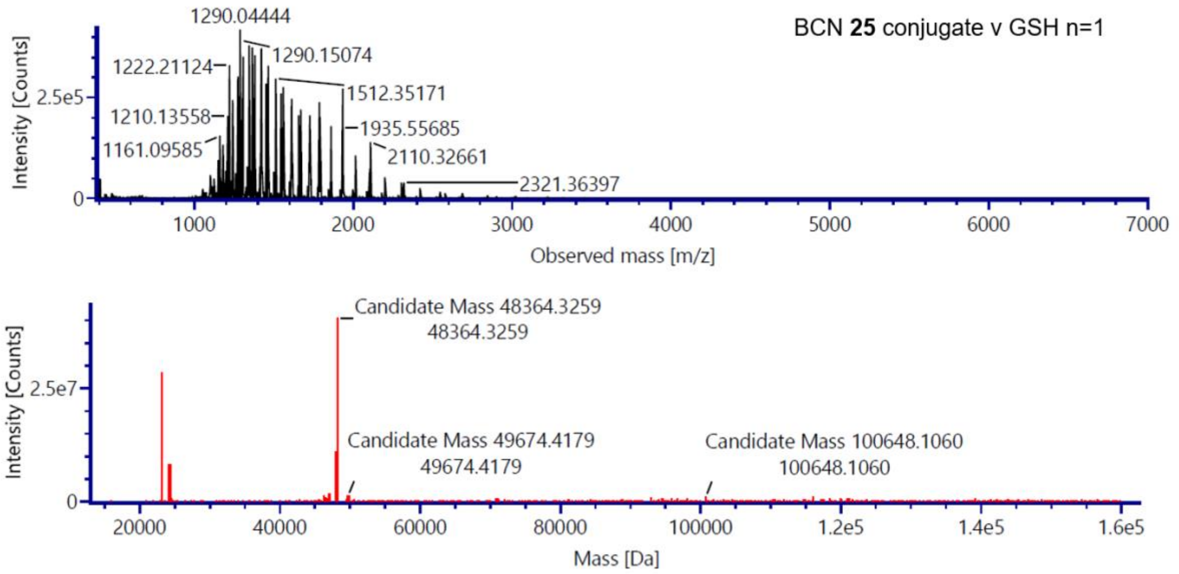

Figure S119. *LCMS spectrum of on protein stability of BCN **25** in GSH. First replicate*

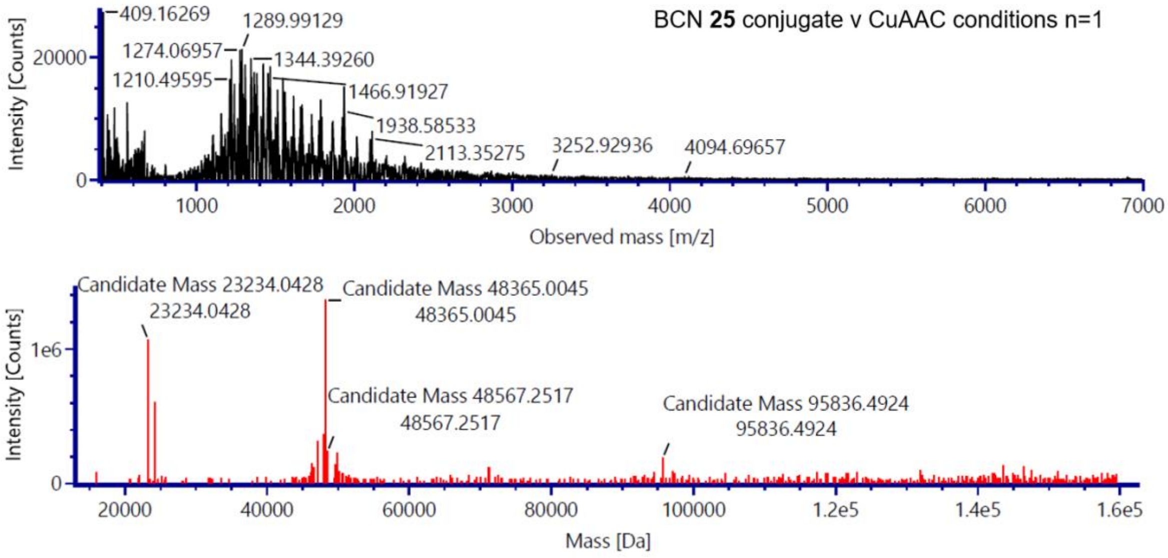

Figure S120. *LCMS spectrum of on protein stability of BCN **25** in CuAAC conditions. First replicate*

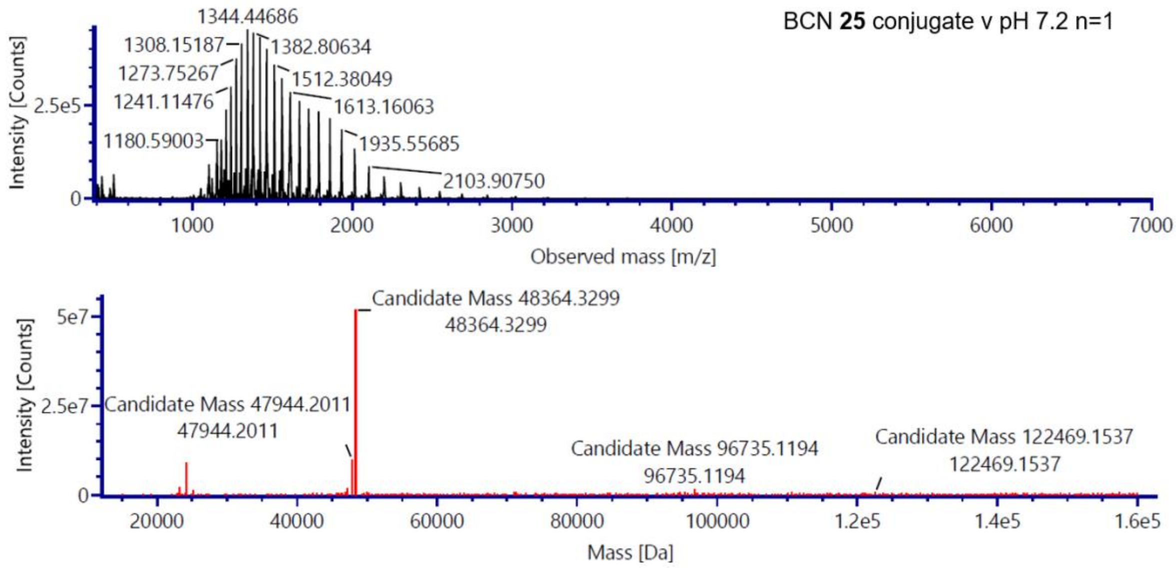

Figure S121. *LCMS spectrum of on protein stability of BCN **25** in pH 7.2. First replicate*

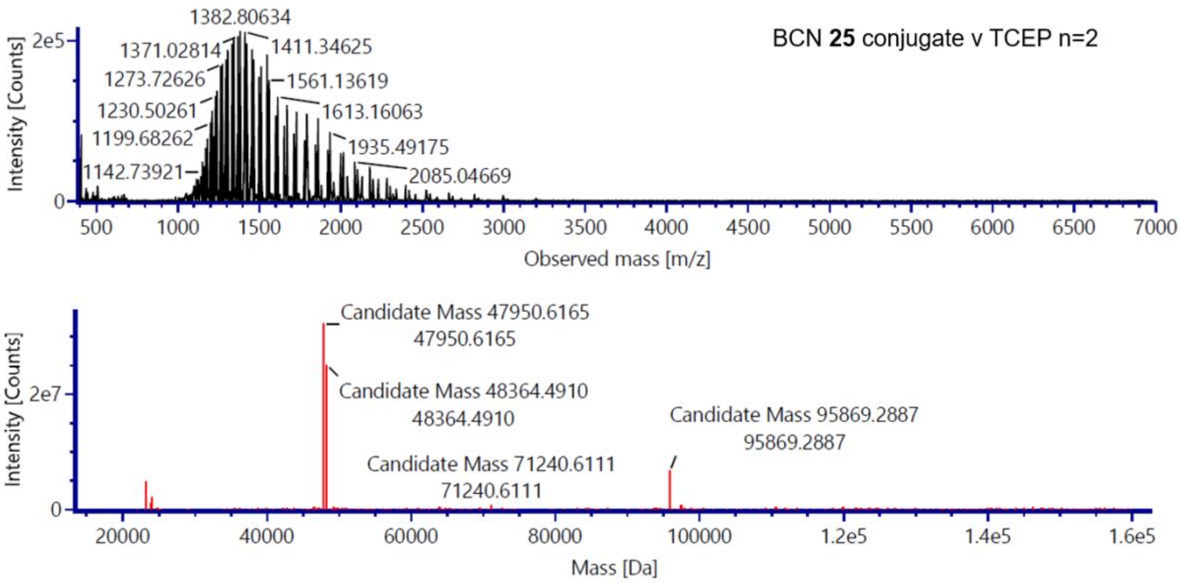

Figure S122. *LCMS spectrum of on protein stability of BCN* ***25*** *in TCEP. Second replicate*

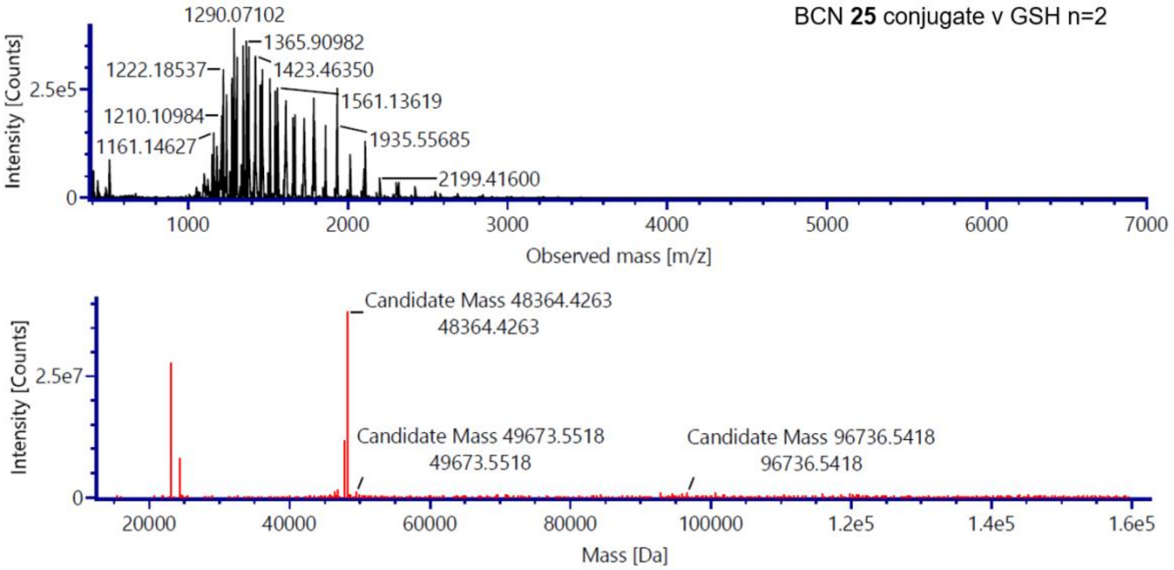

Figure S123. *LCMS spectrum of on protein stability of BCN* ***25*** *in TCEP. Second replicate*

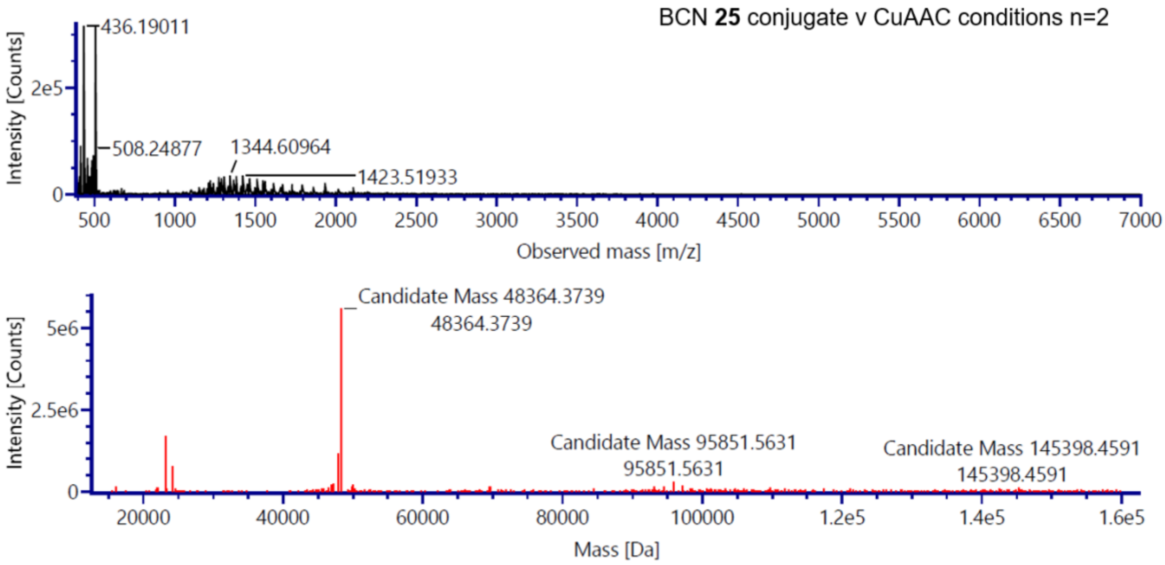

Figure S124. *LCMS spectrum of on protein stability of BCN **25** in CuAAC conditions. Second replicate*

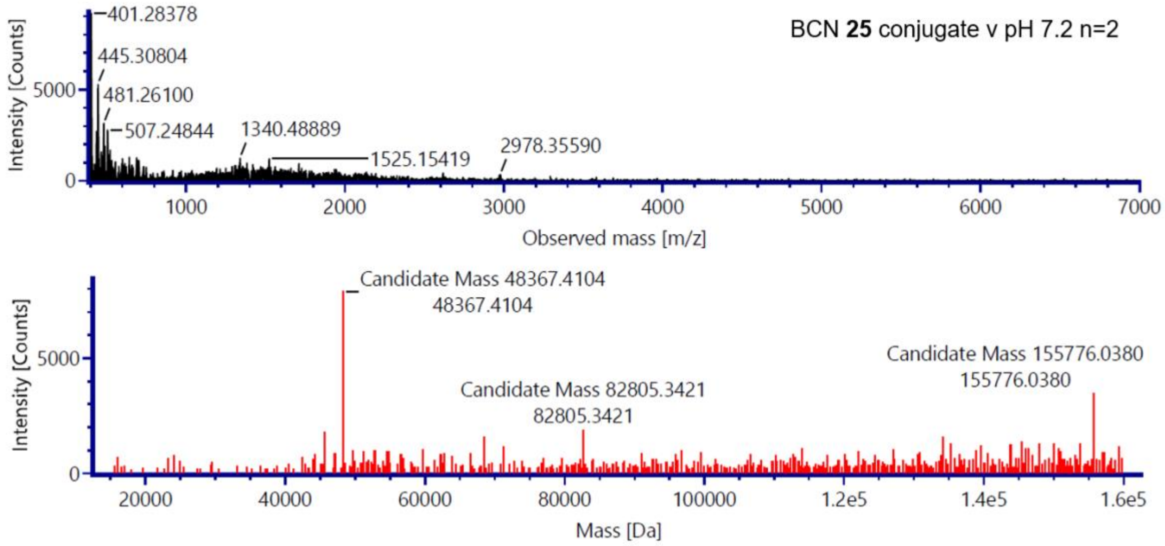

Figure S125. *LCMS spectrum of on protein stability of BCN **25** in pH 7.2. Second replicate*

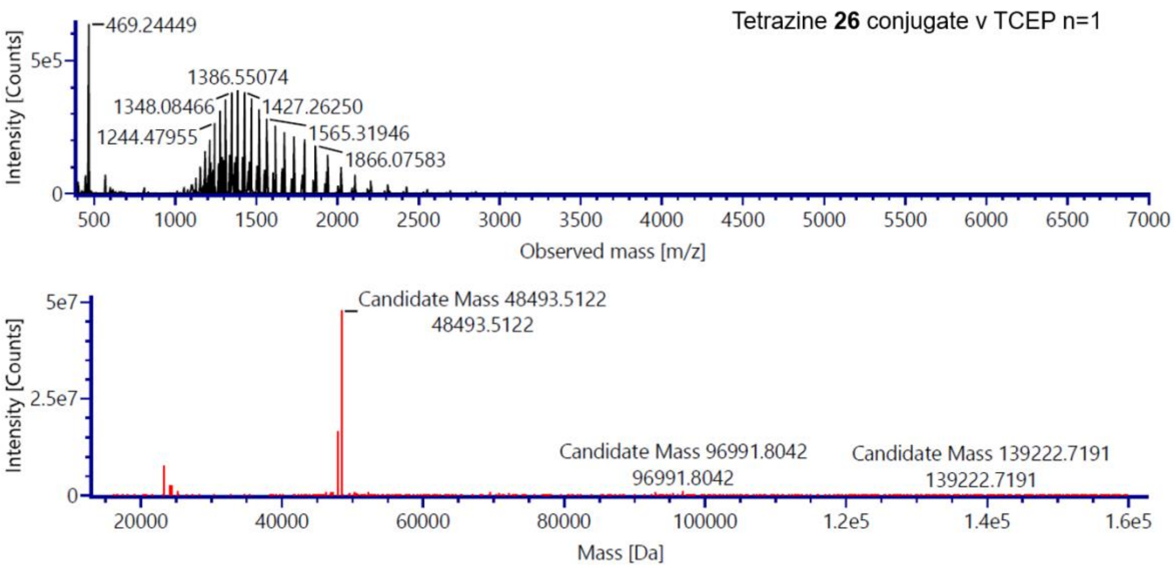

Figure S126. *LCMS spectrum of on protein stability of tetrazine **26** in TCEP. First replicate*

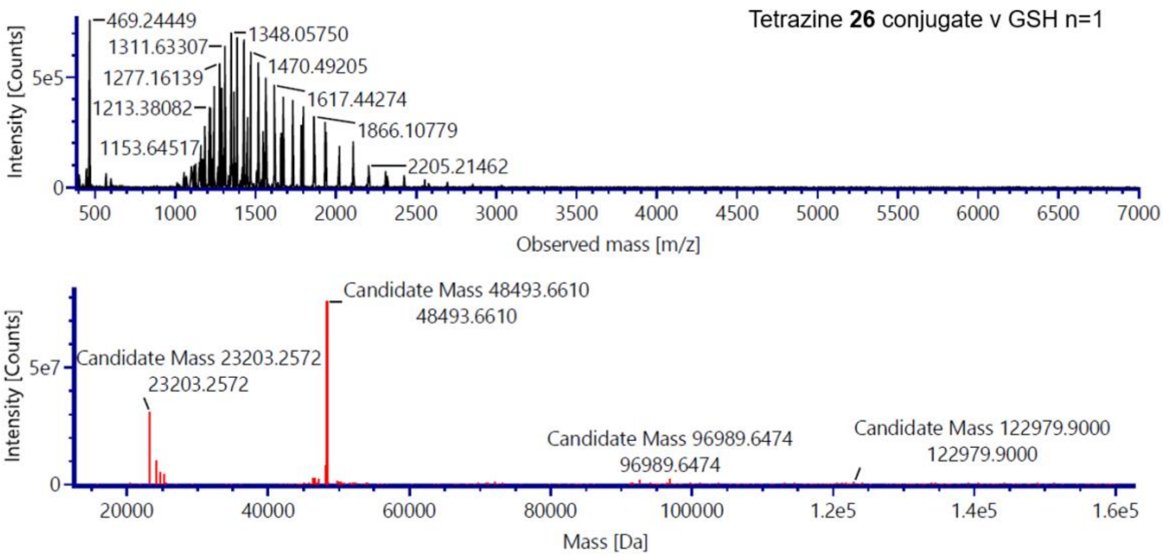

Figure S127. *LCMS spectrum of on protein stability of tetrazine **26** in GSH. First replicate*

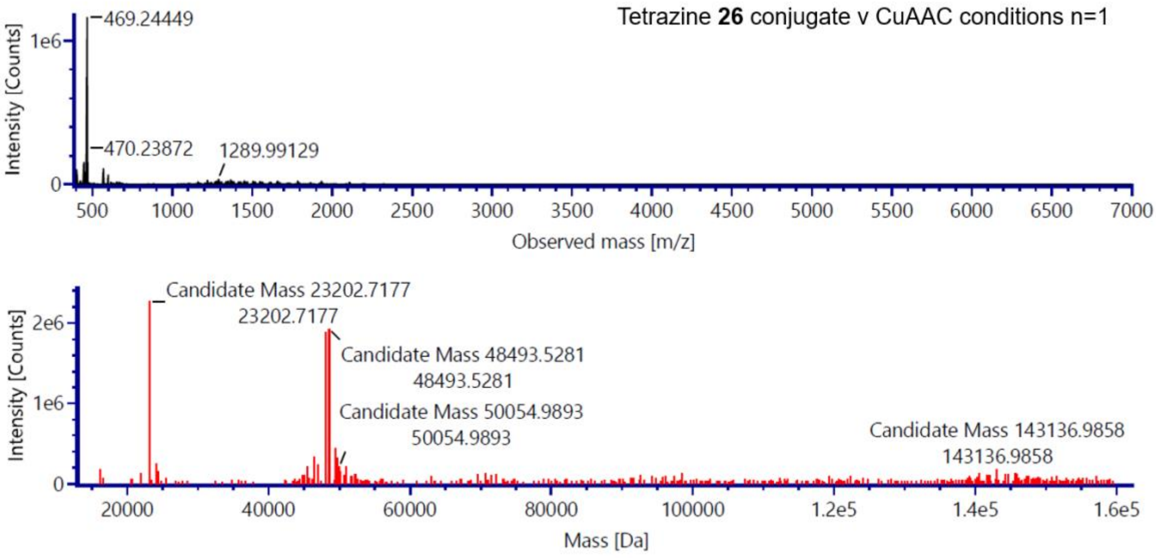

Figure S128. *LCMS spectrum of on protein stability of tetrazine* ***26*** *in CuAAC conditions. First replicate*

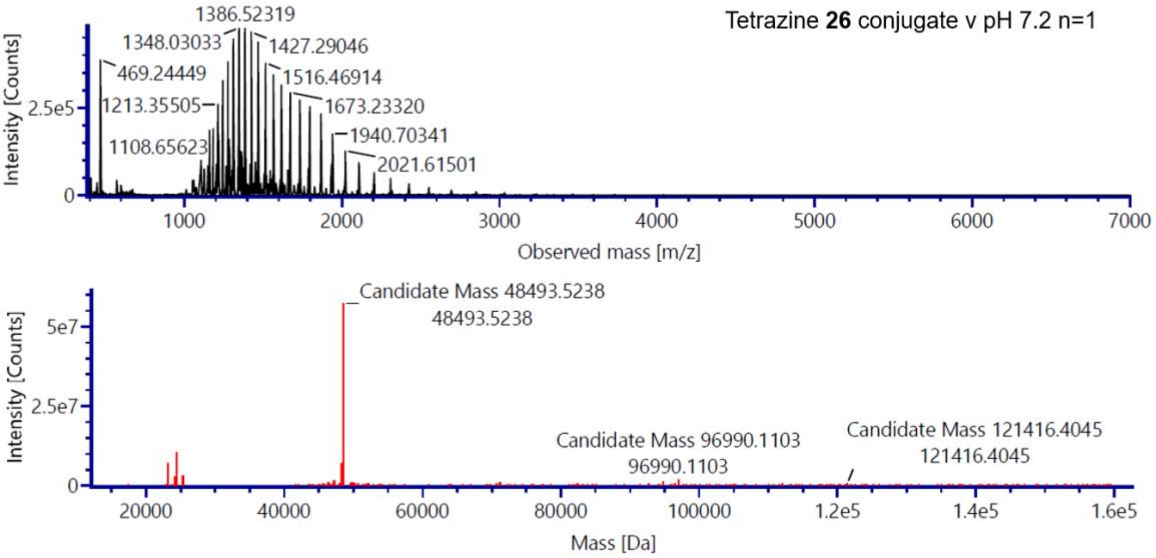

Figure S129. *LCMS spectrum of on protein stability of tetrazine* ***26*** *in pH 7.2. First replicate*

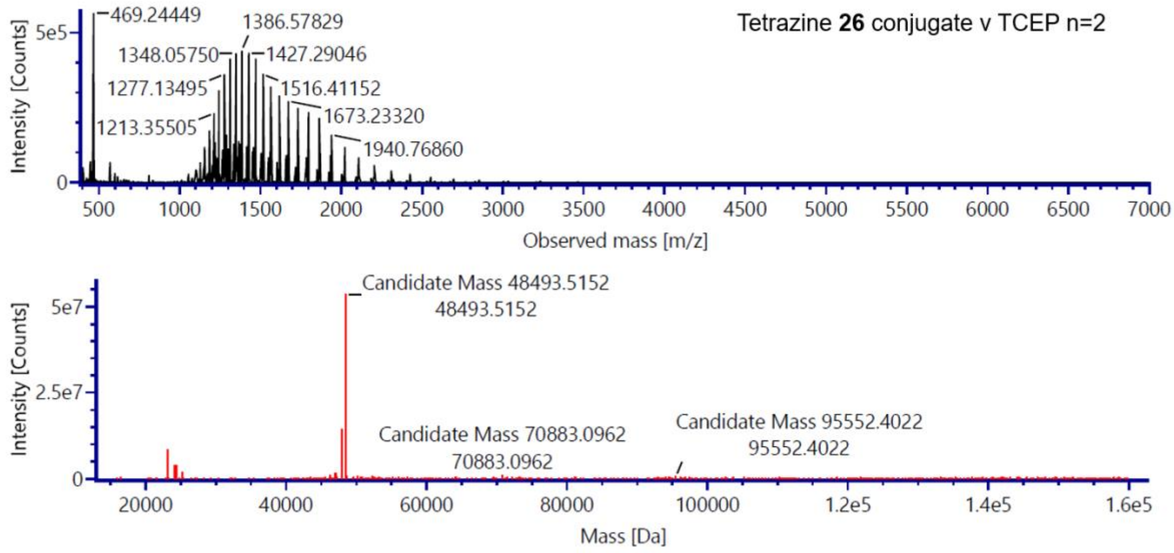

Figure S130. *LCMS spectrum of on protein stability of tetrazine **26** in TCEP. Second replicate*

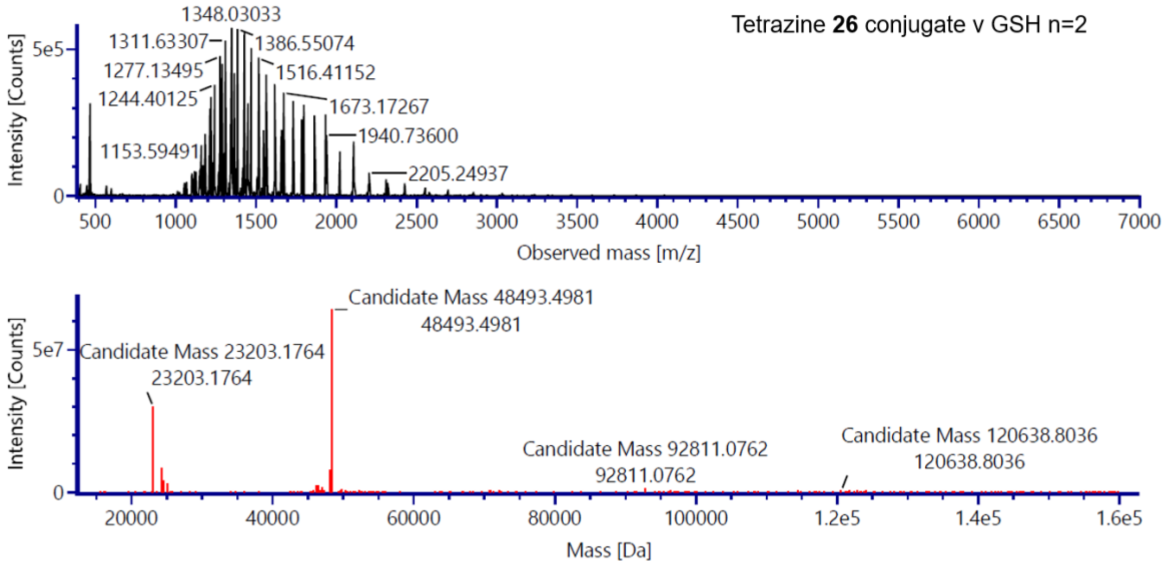

Figure S131. *LCMS spectrum of on protein stability of tetrazine **26** in GSH. Second replicate*

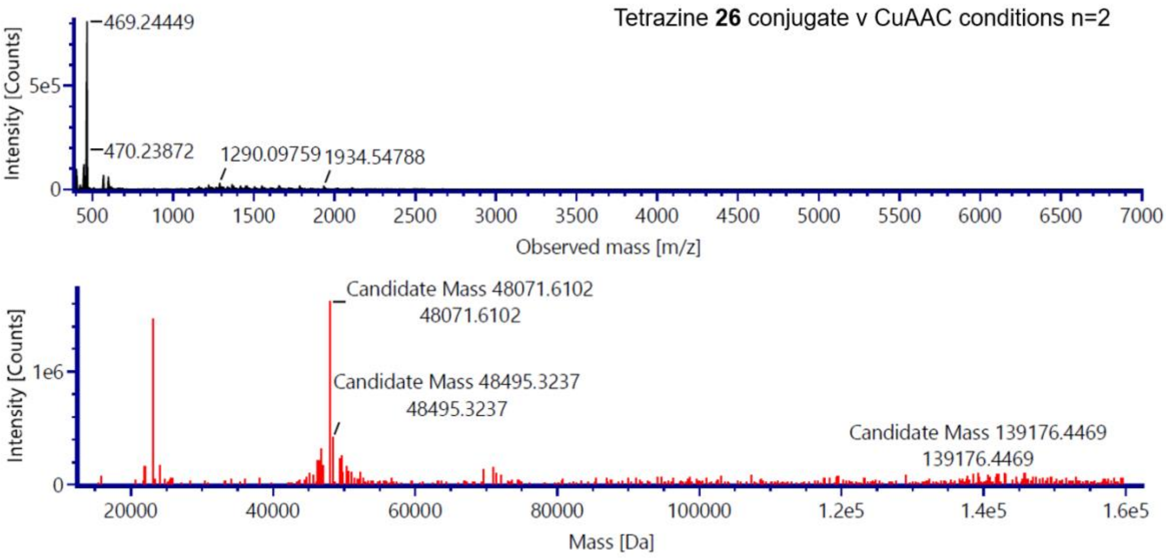

Figure S132. *LCMS spectrum of on protein stability of tetrazine* ***26*** *in CuAAC conditions. Second replicate*

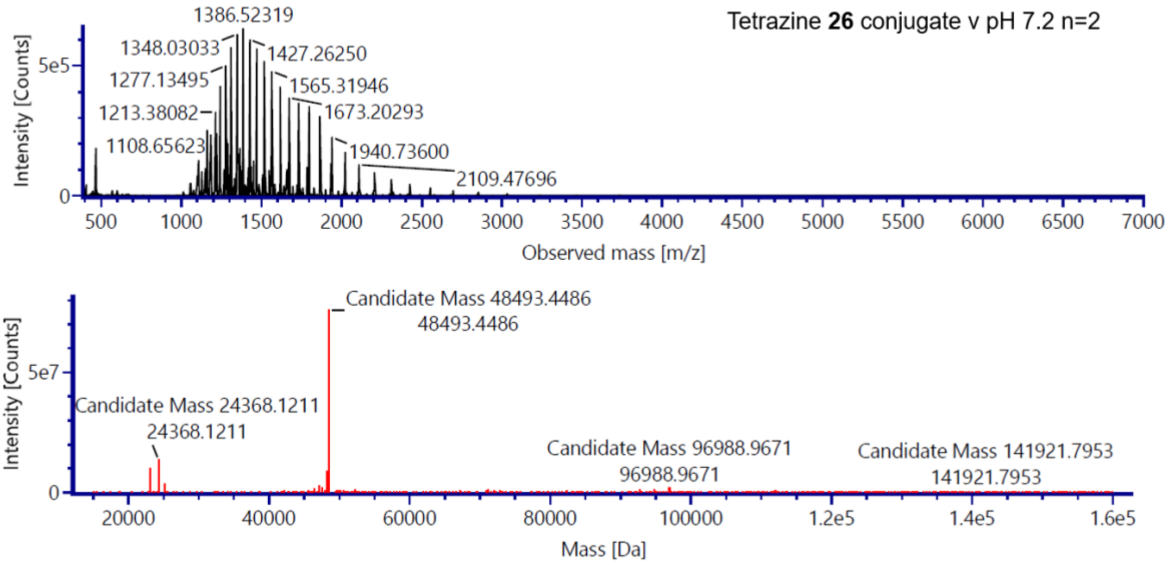

Figure S133. *LCMS spectrum of on protein stability of tetrazine* ***26*** *in pH 7.2. Second replicate*

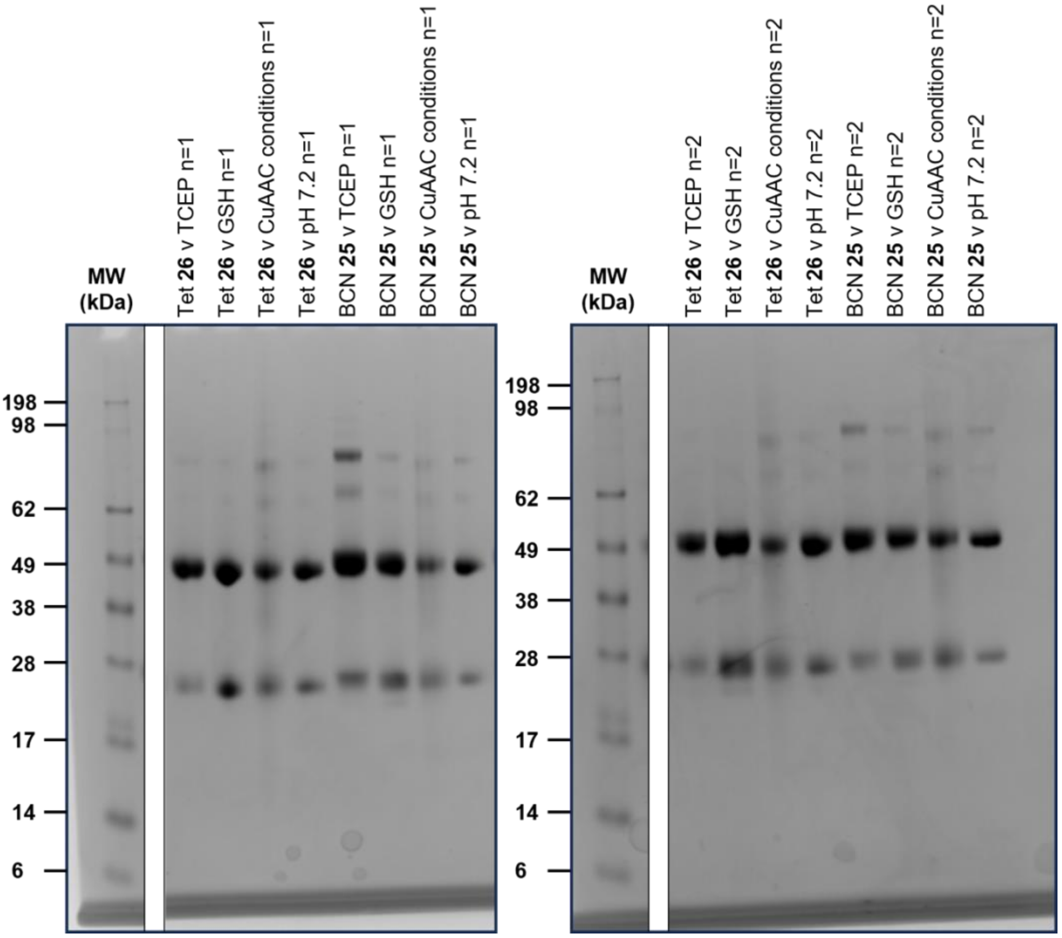

Figure S134. *Reduced SDS-PAGE analysis of the samples generated in the on protein stability experiment. Species at ~25 kDa corresponds to reduced unconjugated Fab, and species at ~ 49 kDa corresponds to Fab conjugate or capped Fab conjugate.*

References

[35] S. Glerup Pedersen, C. Gustafsen, P. Sondergaard Madsen, J. Pold Vilstrup, A. Quattropani, G. Whitlock, A. P. Glossop, S. Felbaek Nielsen , World Pat., *WO2023247754A1*, 2023.

[33a] A. Maruani, P. A. Szijj, C. Bahou, J. C. F. Nogueira, S. Caddick, J. R. Baker, V. Chudasama, *Bioconj. Chem.* **2020**, *31*, 520-529.

[36] Y. Fang, J. C. Judkins, S. J. Boyd, C. W. am Ende, K. Rohlfing, Z. Huang, Y. Xie, D. S. Johnson, J. M. Fox, *Tetrahedron* **2019**, *75*, 4307-4317.

[37] JMP Software, https://www.jmp.com/en_us/home.html, (accessed 07/24)

[23] J. Mason, H. Wilders, D. J. Fallon, R. P. Thomas, J. T. Bush, N. C. O. Tomkinson, F. Rianjongdee, *Digit. Discov.* **2023**, *2*, 1894-1899.

[38] GraphPad Prism, https://www.graphpad.com/, (accessed 09/24)

[39] M. W. Robinson, A. P. Hill, S. A. Readshaw, J. C. Hollerton, R. J. Upton, S. M. Lynn, S. C. Besley, B. J. Boughtflower, *Anal. Chem.* **2017**, *89*, 1772-1777.

[40] C. Bahou, D. A. Richards, A. Maruani, E. A. Love, F. Javaid, S. Caddick, J. R. Baker, V. Chudasama, *Org. Biomol. Chem.* **2018**, *16*, 1359-1366.

[41] I. Nikić, J. H. Kang, G. E. Girona, I. V. Aramburu, E. A. Lemke, *Nat. Protoc.* **2015**, *10*, 780-791.

[33b] A. E. Speers, G. C. Adam, B. F. Cravatt, *JACS.* **2003**, *125*, 4686-4687.

References

[35] S. Glerup Pedersen, C. Gustafsen, P. Sondergaard Madsen, J. Pold Vilstrup, A. Quattropani, G. Whitlock, A. P. Glossop, S. Felbaek Nielsen , World Pat., *WO2023247754A1*, 2023.

[33a] A. Maruani, P. A. Szijj, C. Bahou, J. C. F. Nogueira, S. Caddick, J. R. Baker, V. Chudasama, *Bioconj. Chem.* **2020**, *31*, 520-529.

[36] Y. Fang, J. C. Judkins, S. J. Boyd, C. W. am Ende, K. Rohlfing, Z. Huang, Y. Xie, D. S. Johnson, J. M. Fox, *Tetrahedron* **2019**, *75*, 4307-4317.

[37] JMP Software, https://www.jmp.com/en_us/home.html, (accessed 07/24)

[23] J. Mason, H. Wilders, D. J. Fallon, R. P. Thomas, J. T. Bush, N. C. O. Tomkinson, F. Rianjongdee, *Digit. Discov.* **2023**, *2*, 1894-1899.

[38] GraphPad Prism, https://www.graphpad.com/, (accessed 09/24)

[39] M. W. Robinson, A. P. Hill, S. A. Readshaw, J. C. Hollerton, R. J. Upton, S. M. Lynn, S. C. Besley, B. J. Boughtflower, *Anal. Chem.* **2017**, *89*, 1772-1777.

[40] C. Bahou, D. A. Richards, A. Maruani, E. A. Love, F. Javaid, S. Caddick, J. R. Baker, V. Chudasama, *Org. Biomol. Chem.* **2018**, *16*, 1359-1366.

[41] I. Nikić, J. H. Kang, G. E. Girona, I. V. Aramburu, E. A. Lemke, *Nat. Protoc.* **2015**, *10*, 780-791.

[33b] A. E. Speers, G. C. Adam, B. F. Cravatt, *JACS.* **2003**, *125*, 4686-4687.
